# Associations of diet, race, and other environmental factors with antimicrobial resistance genes in the gut bacterial communities of pregnant women and 3-month-old infants

**DOI:** 10.1128/msphere.00445-25

**Published:** 2025-11-24

**Authors:** Madeleine M. Russell, Andrea Sosa-Moreno, Lixin Zhang, Sarah S. Comstock

**Affiliations:** 1 Department of Food Science and Human Nutrition, Michigan State University 3078, East Lansing, Michigan, USA; 2 Department of Epidemiology, University of Michigan 1259, Ann Arbor, Michigan, USA; 3 Department of Epidemiology and Biostatistics, Michigan State University 3078, East Lansing, Michigan, USA; 4 Department of Microbiology and Molecular Genetics, Michigan State University 3078, East Lansing, Michigan, USA; University of Michigan Medical School, Ann Arbor, Michigan, USA

**Keywords:** antibiotic resistance, pregnancy, infancy, cohort, tetracyclines, human milk, mode of delivery

## Abstract

The emergence of pathogens resistant to antimicrobials has become a forefront concern for clinicians and patients alike. Antimicrobial resistance (AMR) is exacerbated by the misuse and overuse of antibiotics. Pregnant women and their infants are an important area of focus, as antibiotic use during this vulnerable period of development may generate reservoirs of AMR genes, which would contribute to future risk. Identifying the extent of antibiotic use and its association with ARG composition and persistence within this window is crucial. We sought to characterize the gut resistomes of 3-month-old infants (*n* = 212) and pregnant women in their third trimester (*n* = 99) to assess ARG burden in these populations. For a subset of women and their infants (*n* = 33 pairs), we explored overlap of ARG. Preliminary analyses demonstrated that pregnant women and infants had markedly different resistome communities and identified other environmental and demographic characteristics to be associated with univariate differences in infant ARG composition. When controlling for the race of the mother, infant diet, and infant antibiotic exposure since birth, delivery by cesarean section was associated with increased diversity of ARG relative to the diversity of ARG in the samples from vaginally born infants. Cesarean-born infants had increased richness of aminoglycoside ARG and increased diversity of beta-lactamase and tetracycline ARG relative to vaginally born infants. Furthermore, infants consuming any formula had increased overall richness and diversity of ARG in multivariate analyses. This study provides further insight into how diet and method of delivery are associated with resistome composition within the first 3 months of infant microbiome development.

**IMPORTANCE:**

Pregnancy and the first 3 months of life are vulnerable periods for antibiotic exposure and subsequent development of antimicrobial resistance (AMR). AMR is an increasingly worrisome problem for global public health. The full repertoire of AMR genes present in the gut collectively is referred to as the resistome. Herein, the associations between a variety of demographic and environmental factors, including race of the pregnant women, sex of the infant, mode of delivery, amount of breast milk consumed in infant diet, and antibiotic exposure during the first 3 months of life, with resistome composition are reported. Infants consuming any formula had a greater richness and diversity of ARG overall, and cesarean-born infants had greater diversity of ARG within their resistomes. These findings give insight into the early seeding of the infant resistome, which is crucial to understanding how the resistome develops throughout life.

## INTRODUCTION

Antimicrobial resistance (AMR) has risen in importance in recent years as a pressing global health concern. The estimated cost of AMR in the US is $55 billion annually, with a significant percentage of those costs going directly to preventing the further spread of AMR infections (1). Infants and pregnant women are of particular interest to study with regard to AMR. The malleability of the early gut microbiome and subsequent presence of AMR genes in infants’ microbiomes create an ideal target for interventions aimed at reducing early AMR reservoirs, also termed the resistome (2). The reservoir of antibiotic-resistant genes (ARGs) poses difficulties for treating infants with bacterial infections. This is especially true for pre-term infants whose immune system deficiencies make them extra vulnerable, putting them at enhanced risk when they acquire AMR infections (3). The vertical transmission of AMR genes from pregnant women to infant gut microbiome has been previously documented (4, 5). However, the extent of the impact of this transmission on the composition of the infant resistome is unclear, given the numerous colonization sources of the early microbiome (2, 6). Understanding how the infant resistome assembles is key to reducing the spread of AMR, and the pregnancy resistome likely plays an important role.

The development of AMR within the human gut microbiome is multifaceted, and the factors that contribute to the transmission of AMR genes between pregnant women and infants are still being identified. The key moderators of AMR presence within infant guts include genetics, mode of feeding for the infant, and mode of delivery (2, 7). Pregnant women’s and infant’s genetic predisposition influence bacterial diversity in the gut, which in turn influences the accumulation of AMR genes present (6). Pregnant women’s genetics can shape the probiotic properties of breast milk, which in turn can influence microbial composition within the infant gut (6). A previous study on the accumulation of AMR in adults has shown that increased consumption of fiber, decreased protein intake, and diverse palates are associated with decreased ARG burden (8). However, there has been little characterization of the role for early infant feeding patterns in the accumulation of ARG within a diverse cohort of healthy infants (9). Cessation of breastfeeding before the recommended 6 months of age has been associated with increased AMR (10), while formula feeding has been linked to increased presence of opportunistic pathogens and AMR genes (11). The predominant contributor to the presence of antimicrobial-resistant bacteria in the infant gut, up to at least 1 year of age, is currently thought to be mode of delivery (12, 13). Cesarean sections are associated with increased AMR and perturbation of the normal gut microbiota (9, 14). Identifying the factors that influence the presence of AMR genes will enable the development of methods of intervention aimed at preventing the spread of AMR.

Lack of adherence to prescription regimens and overuse of antibiotics during pregnancy and early infancy makes the infant gut microbiome a prime niche for the accumulation of AMR (15–17). AMR includes resistance to several antimicrobial agents, including antibiotics. The use of antimicrobials for pregnant women or infants during the perinatal period is associated with an increase in the presence of AMR genes in infants, persisting for up to 1 year of age (14, 18). Antibiotic treatment is typically associated with an increased abundance of specific bacterial species, as well as overall enrichment of ARG within the gut (19, 20). Additionally, the infancy resistome contains greater richness of AMR relative to adults’ regardless of antibiotic exposure (3, 12, 16, 17). Investigating the gut resistomes in a population of healthy infants, such as the cohort identified within, will help to elucidate the extent to which antibiotic exposure contributes to ARG composition.

Part of the concern with the transmission of ARG is the nature of horizontal gene transfer (HGT) between different bacterial species (6). Bacterial stress responses in the presence of antibiotics can cause changes in bacterial gene expression, which could potentially contribute to HGT. This is aided in part through mobile genetic elements (MGEs), which can essentially hop between bacteria and insert into new genomes (14). For these reasons, it is important to understand how various factors impact the spread of MGEs as well as other ARGs.

Characterizing the environmental factors that influence the development and dissemination of AMR genes in pregnant women and their infants could enhance understanding of AMR patterns. While several studies have reported ARG in infants and healthy adults, there is a lack of understanding of the overlap between infants and their birthing parents. The focus herein was to determine the environmental and behavioral factors that contribute to the development of the human gut resistome within a population of infants, pregnant women, and maternal-infant dyads. We sought to examine the contribution of different host demographics and environmental factors on the composition of the early resistome.

## MATERIALS AND METHODS

### Study population

**Fig 1:**
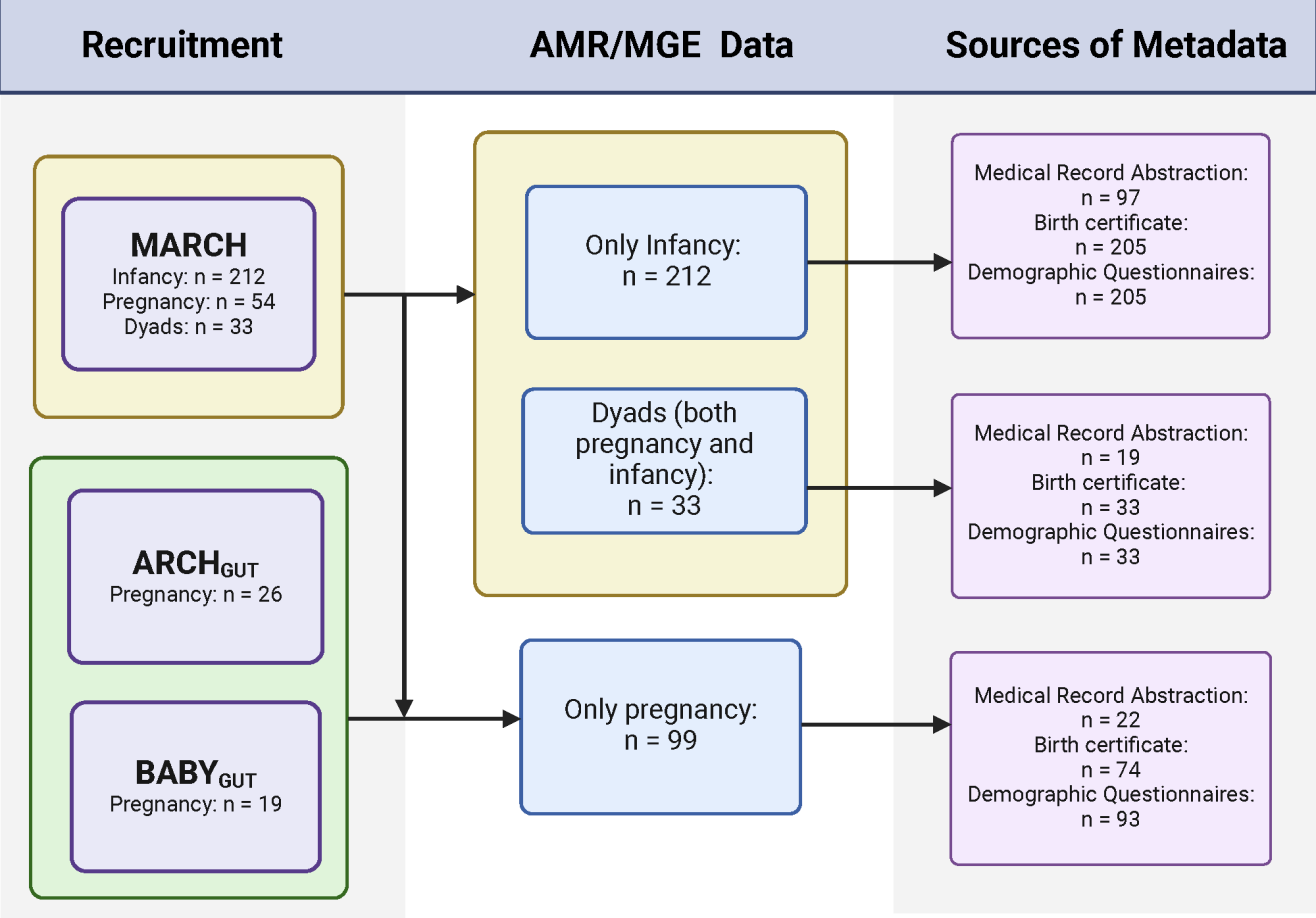
Flow chart of participants. Participants were recruited through MARCH, ARCH_GUT_, and BABY_GUT_ cohorts. The MARCH cohort provided the majority of pregnancy samples (*n* = 54), all of the provided infancy samples (*n* = 212), and all the dyads (*n* = 33) included in the study. Please note that the dyads sample size indicated in the MARCH recruitment box includes pregnancy and infancy samples that are also represented in the infancy and pregnancy counts in the box. Stool samples were collected from infants (*n* = 212) and pregnant women (*n* = 99) and were analyzed for AMR/MGE presence, abundance, richness, and diversity. Medical record abstraction (MRA), birth certificate (BC), and demographic questionnaires were obtained and matched to participants’ AMR/MGE data. BC data of participating infants was used to determine characteristics of infants who provided a stool sample and pregnant women’s demographics (*n* = 205), while MRA was available for (*n* = 97) infants who provided stool samples to characterize their mothers’ antibiotic use during pregnancy.

Participating pregnant women were recruited from the Michigan Archive for Research on Child Health (MARCH) cohort (21) or as part of two other cohorts (ARCH_GUT_ and BABY_GUT_) (22). ARCH_GUT_ and BABY_GUT_ have been described (22). The MARCH cohort is based in Michigan’s lower peninsula and is a long-term observational population-based pregnancy and birth cohort (21). For each cohort, pregnant women were recruited from participating OB/GYN offices early in their pregnancy and provided written consent under Michigan State University IRB protocols (project numbers 14-170M, 15-1240, 17-1352, 16-1429, and C07-1201). Inclusion criteria for all cohorts included healthy pregnant women over 18 years of age and who had a pre-pregnancy BMI over 18.5.The collection of available participant data is shown in Fig. 1. Medical record abstraction for antibiotic history was performed for a subset of the infants recruited in the larger cohorts (*n* = 267), and a proportion of this group (*n* = 97) also provided a stool sample. For infants who also provided a stool sample, birth certificate data (*n* = 205) collected at the time of birth were used to inform other demographics and covariates. Information regarding infant diet over the previous week (100% breast milk, 50–80%, and <20%)was extracted from a self-report questionnaire pregnant women filled out for themselves and their infants when the infants were approximately 3 months of age (*n* = 212). For multivariate analysis, infants were grouped into two categories: exclusively breastfed (BF, *n* = 85) or non-exclusively breastfed (FF, *n* = 107). For analyses of race, samples were pooled by maternal race as reported during pregnancy into white (*n* = 153) or non-white (*n* = 51) due to low sample size.

### Study design and AMR gene abundance

Fecal samples were collected from pregnant women (*n* = 99) during their third trimester of pregnancy and from infants (*n* = 212) at 3 months of age. Microbiome and resistome description of some of these infants at age 6 months is reported elsewhere (4, 23). Gut microbiome composition at pregnancy, 1 week, 3 months, 6 months, and 12 months for a subset of these participants has been previously published (21, 23, 24). Infant samples were collected from diapers and stored via Omnigene (DNA Genotek, Ontario Canda) and Para-Pak Clean Vial collection tubes (Meridian Biosciences, Cincinnati, OH, USA). Samples were collected at home and sent to the lab via mail, then aliquoted and stored at -80°C upon arrival to the lab. The Qiagen PowerSoil DNeasy Isolation kit (Qiagen, Carlsbad, CA, USA) was used for DNA isolation. Following DNA extraction, AMR genes were detected and quantified using Takara SmartChip Real-time quantitative PCR (qPCR). Primers used for detection of genes have been previously used and validated (4, 25–29). All qPCR reactions were run in triplicate using 480 SYBR Green (4). A PCR program of 2 min 53 s at 95°C, then 40 cycles of 95°C for 34 s and 64 s at 60°C was followed. A Ct cutoff of 30 was used to reduce potential false positives. Normalized abundance was calculated based on the number of genetic copies (GCs) for each gene based on Ct values. GCs were normalized using the 16S rRNA gene to determine normalized abundance.


(1) $$GC=10^{\left(\frac{30-CT}{3.33}\right)}$$

(2) $$\text{Normalized abundance}=\frac{GC(ARG)}{GC(16\text{S}\;rRNA)}$$

Based on a preliminary assessment of DNA pooled from 12 fecal samples from infants and pregnant women in the same cohort (4), 133 genes were included in this analysis. In addition to being analyzed separately, genes of interest were also grouped by ARG (*n* = 106) class (aminoglycoside, beta-lactamase, macrolide-lincosamide-streptogramin B [MLSB], multi-drug resistance [MDR], sulfonamide, tetracycline, and vancomycin), and MGEs (*n* = 27). Genes detected and their functional classification are reported in Table S1.

### Statistical analyses

Differences in pregnant women and infant ARG patterns were assessed. Alpha (within-sample) diversity was assessed using the vegan package in R (30) and was measured via richness (defined as the total number of ARG/MGE per sample), as well as Shannon and Inverse-Simpson indexes. Since the data were not normally distributed, Wilcoxon and Kruskal-Wallis non-parametric tests were used to test for significant differences between groups, and the Dunn test was used for post hoc testing with Bonferroni correction. Beta (between-community) diversity was assessed using Sorensen (community composition) and Bray-Curtis (community structure) dissimilarity metrics. Beta diversity was plotted via a principal coordinate plot (PCoA), and PERMANOVA (adonis R package) was performed to test for significant differences. PERMDISP (betadisper in vegan R package) was used to identify differences in sample dispersion. The Benjamini-Hochberg method was used to adjust for false positives (31). Using data from the related and unrelated dyads (*n* = 33), Sorensen and Bray-Curtis dissimilarity distances were visualized on Kernel Density Plots using the ggplot2 package in R (10). The boxplots represent the Bray-Curtis and Sorensen dissimilarity distances along PCoA 1 between groups. “Type” *P* values compared distances between samples of the same type (infancy/infancy; pregnancy/pregnancy) versus those of different types (infancy/pregnancy). “Family” *P* values compared distances between unrelated pregnancy/infancy samples with distances between related pregnancy/infancy samples (i.e., infants and their mothers). “Type and family” compared distances between unrelated samples of the same type (pregnancy/pregnancy; infancy/infancy) and related samples for infancy/pregnancy. An lmp function was used to determine if distances were statistically different. The lmp function, a modification of the lm function, uses permutation tests. Like the lm function, the lmp function can be used to carry out regression, one-way analysis of variance, and analysis of covariance. Univariate analysis of alpha diversity using a linear model was conducted on selected infant characteristics and followed by multivariate analysis using a multiple linear model (function lm in R version 4.2.0). Beta diversity was assessed using PERMANOVA (adonis2 function in vegan). Samples were clustered into a dendrogram based on the log of normalized gene abundance through hierarchical clustering based on Euclidean distance utilizing complete linkage (function hdist in R) (32, 33). Multinomial logistic regression based on clustering was performed using the multinom function in R (multinomial logistic regression model in nnet R package) (34).

## RESULTS

### Participant characteristics

Participant information is provided in Table 1. Among pregnant women who provided stool samples (*n* = 99), the predominant race was white (*n* = 78), followed by black (*n* = 11), Asian or Pacific Islander (*n* = 2), and mixed race/other (*n* = 2). Additionally, for pregnant women for whom medical record abstraction was available, the average pre-pregnancy BMI was 28.2. Pregnant women were grouped into BMI categories based on Centers for Disease Control and Prevention recommendations of normal (18.5 < BMI < 25; *n* = 40), overweight (25 ≤ BMI < 30; *n* = 28), and obese (BMI ≥ 30, *n* = 24). Average age was 30.9 years. Among infants who provided stool samples (*n* = 212), sex was equally represented (females *n* = 105, males *n* = 100), and the primary mode of delivery was via vaginal birth (*n* = 134), then cesarean section (*n* = 71). Infants were grouped into feeding categories based on percentage of human milk consumed in diet, with 100% breast milk (*n* = 86) being the most prevalent, followed by diets consisting of <20% breast milk (*n* = 80), and finally infants’ diets consisting of 50–80% breast milk (*n* = 26).

**TABLE 1:** Characteristics of participants who provided stool samples

Characteristic	Description	Result
Pregnant women (*n* = 99)		
Race, *n* (%)^*a*^	Asian or Pacific Islander	2 (3.9)
	Black	11 (19.5)
	White	78 (74.6)
	Other	2 (3.9)
Smoking status, *n* (%)*^b^*	Non-smoking	42 (89.5)
	Smoking	6 (10.5)
Pre-pregnancy BMI category, *n* (%)^*c*^	Normal	40 (43)
	Overweight	28 (30)
	Obese	24 (26)
Pre-pregnancy BMI, mean (SD)^*c*^		28.2 (7.55)
Mean maternal age, years (SD)^*d*^		30.9 (5.35)
Infants (*n* = 212)		
Sex, *n* (%)	Female	105 (51.2)
	Male	100 (48.8)
Mode of delivery, *n* (%)	Cesarean	71 (34.6)
	Vaginal	134 (65.3)
Plurality, *n* (%)	Singleton	196 (95.6)
	Twins	9 (4.4)
Percentage of diet consisting of breast milk, *n* (%)	100%	86 (44.8)
	50–80%	26 (13.5)
	<20%	80 (41.7)
Antibiotics given during first 3 months of life, *n* (%)	Yes	28 (14.7)
	No	163 (85.3)
Mean weight, g (SD)		3,304 (596.73)

^*a*^ Missing data (*n* = 6).

^*b*^ Missing data (*n* = 51).

^*c*^ Missing data (*n* = 7).

^*d*^ Missing data (*n* = 26).

Since many participants were not part of a dyad pair, maternal characteristics of all participating infants (*n* = 212) are reported in Table S2. Most infants from whom stool was collected had mothers who identified as white (*n* = 153), then black (*n* = 40), Asian (*n* = 3), and mixed racial identity/other (*n* = 8). This distribution is representative of the population of Michigan (35). Infant’s mothers pre-pregnancy body mass index (BMI) average BMI was 28.7 (Table S2) and were grouped into BMI categories of normal (*n* = 84), overweight (*n* = 48), and obese (*n* = 68). The mean age of infant’s mothers was 30.85 (range 20–52) years, and the average estimated weeks gestation was 38.37.

### ARG and MGE in infants and pregnant women

Of the 133 screened genes, 131 were found at least once in a pregnancy sample, and all of them were found at least once in an infant sample. On average, 53 ARG were found in pregnancy samples, while 40 ARG were found in infancy samples. The number of ARG and MGE found in infants and pregnant women, as well as the overall study population, is provided in Table S3. ARGs contributing to the MDR ARG class had the highest diversity in genes detected per sample on average (11 genes/sample), followed by genes in the macrolide-lincosamide-streptogramin B ARG class (MLSB; 7 genes/sample), then aminoglycoside (7 genes/sample), tetracycline (6 genes/sample), and beta-lactamase (5 genes/sample) ARG classes. There were low ARG averages per sample of sulfonamide (two genes/sample), fluoroquinolones (one gene/sample), and vancomycin (one gene/sample) ARG classes. The genes detected most overall within the entire study population are listed in ascending order in Table 2. A transposase, *tnpA*, was present in 87% of infants’ samples and 97% of pregnant women’s samples, while *tetQ*, a tetracycline ARG, was found in all pregnant women’s samples. Two of the three most prevalent genes were also related to tetracycline resistance (*tetM* and *tetW*). *mefA*, which targets MLSB, was the second most detected ARG, having been detected in 90% of overall samples, specifically within 89% of infant samples and 92% of pregnant women’s samples.

**TABLE 2:** Most prevalent ARG found in gut bacterial communities of pregnant women and 3-month-old infants, in ascending order of detection within all samples

Gene	Target	Present in infancy and pregnancy samples (*n* = 311)	Present in infancy samples (*n* = 212)	Present in pregnancy samples (*n* = 99)
tetQ	Tetracycline	273 (88%)	174 (82%)	99 (100%)
acrF	MDR	275 (88%)	190 (90%)	85 (86%)
tolC	MDR	275 (88%)	189 (89%)	86 (87%)
tnpA	MGE	280 (90%)	184 (87%)	96 (97%)
tetM	Tetracycline	280 (90%)	187 (88%)	93 (94%)
mefA	MLSB	280 (90%)	189 (89%)	91 (92%)
tetW	Tetracycline	283 (91%)	185 (87%)	98 (99%)

### Comparing the pregnancy and infancy resistome

**Fig 2:**
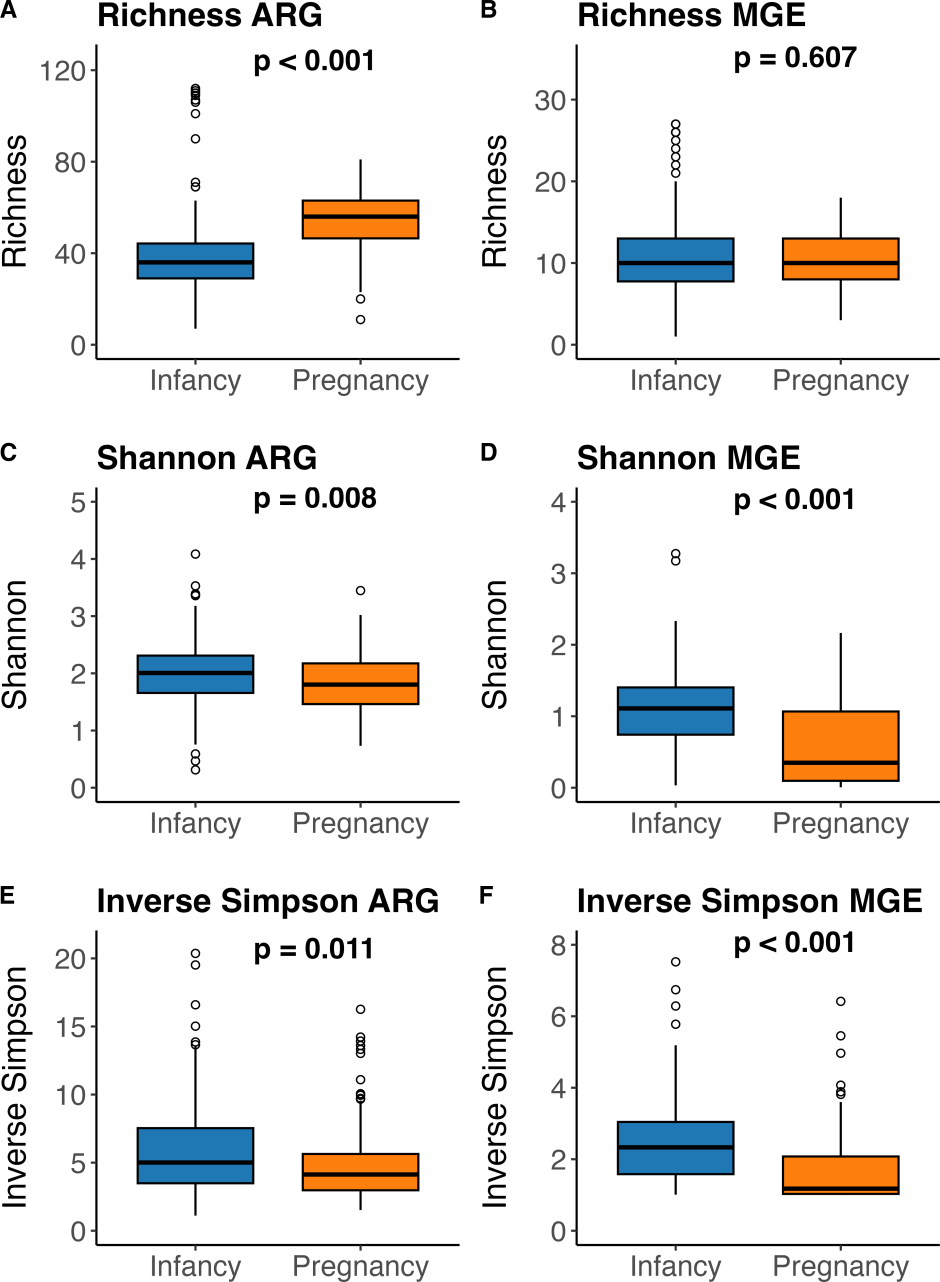
Box and whisker plot of alpha diversity metrics of richness, Shannon, and Inverse Simpson for the gut resistomes of infants and pregnant women. Infancy samples are blue, while pregnancy samples are orange. The horizontal line in the center of the box and whisker plot represents the median, while the upper and lower limits of the box represent the interquartile range (IQR). The whiskers are determined by Q1/Q3 ± 1.5 × IQR and anything falling outside of the whiskers represents an outlier. The gut bacterial communities of pregnant women had greater richness of ARG (**A**) but not MGE (**B**). Infants had greater diversity of ARG (**C and E**) and MGE (**D and F**).

Pregnant women’s resistomes were significantly enriched in normalized abundance of aminoglycoside, MLSB, and tetracycline ARG. Infant samples were significantly enriched in normalized abundance of MGE, MDR, and vancomycin ARG (Fig. S1). However, the overall detection of normalized abundance of vancomycin ARG in pregnancy and infancy samples was minimal and thus is not visible in Fig. S1. Pregnant women had greater richness of ARG (Fig. 2A) but not MGE (Fig. 2B) compared with infants. Furthermore, infants had greater Shannon and Inverse Simpson diversity of ARG and MGE (Fig. 2C, D, E and F).

**Fig 3:**
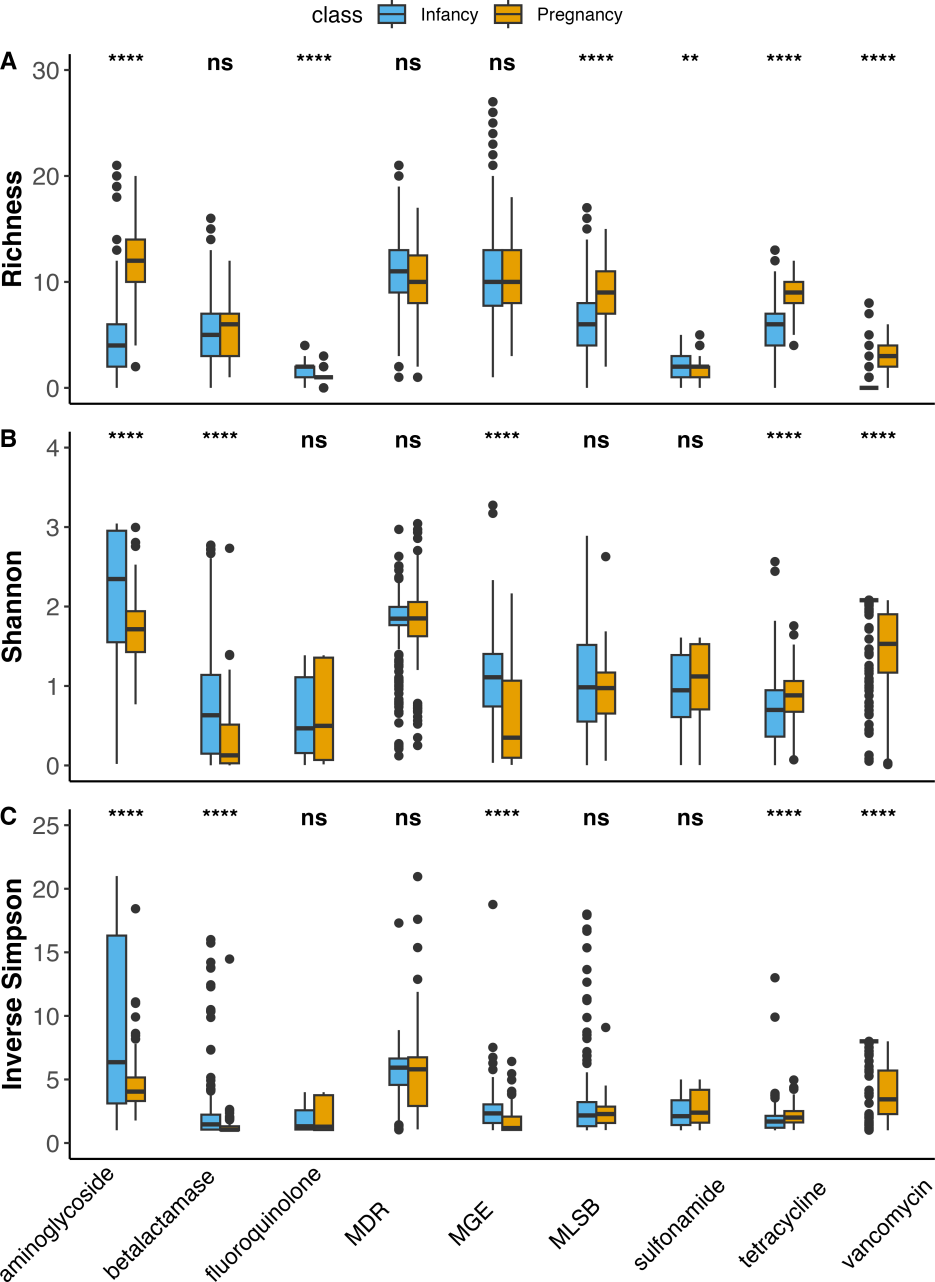
Box and whisker plot of alpha diversity, including richness (**A**), Shannon diversity (**B**), and Inverse Simpson (**C**), by ARG class for infants and pregnant women. The horizontal line in the center of the box and whisker plot represents the median, while the upper and lower limits of the box represent the interquartile range (IQR). The whiskers are determined by Q1/Q3 ± 1.5 × IQR and anything falling outside of the whiskers represents an outlier. Statistical significance is indicated by NS for non-significant (*P* > 0.05). **P < 0.01, and ****P < 0.0001. Pregnancy samples had a greater richness (**A**) of aminoglycoside, MLSB, tetracycline, and vancomycin ARG, whereas infants had greater fluoroquinolone and sulfonamide ARG. Infancy samples also had greater Shannon diversity (**B**) of aminoglycoside, beta-lactamase, MGE, and vancomycin ARG, whereas pregnancy samples had greater tetracycline ARG. Infancy samples did have increased Inverse Simpson diversity (**C**) of aminoglycoside, beta-lactamase, and vancomycin ARG. Pregnancy samples had increased Inverse Simpson diversity of tetracycline ARG.

There were differences in alpha diversity metrics by ARG class (Fig. 3). Pregnancy resistomes had a greater richness of aminoglycoside, MLSB, tetracycline, and vancomycin ARG (richness: *P* < 0.05), as well as an increased diversity of tetracycline (Inverse Simpson, Shannon: *P* < 0.05) (Table S4). Infants had a greater richness of fluoroquinolones and sulfonamides (richness: *P* < 0.05), as well as an increased diversity (Inverse Simpson, Shannon: *P* < 0.05) of aminoglycoside, beta-lactamase, and vancomycin ARG. Overall, pregnant women and infants were found to have distinctive ARG and MGE compositions.

**Fig 4:**
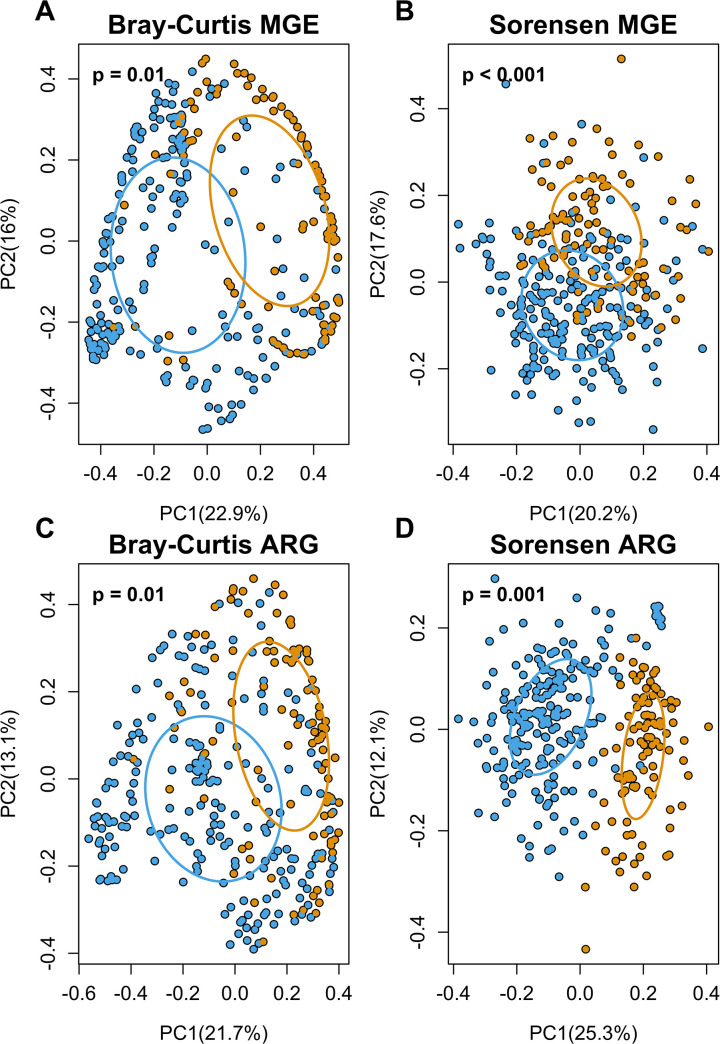
The gut resistomes of infants and pregnant women have distinctive ARG/MGE composition and structure. PCoA of Bray-Curtis dissimilarity for MGE (**A**) and ARG (**C**). Sorensen dissimilarity for MGE (**B**) and ARG (**D**). Orange dots represent pregnant women, while blue dots represent infants. Each dot represents an individual infant or pregnant woman, while ellipses are based on the centroids of each group. The greater the distance between samples, the greater the dissimilarity. There were community structural differences based on PCoA Bray-Curtis dissimilarity for MGE (**A**) (PERMDISP: *P* value < 0.0001), and compositional differences based on Sorensen dissimilarity (**B**) (PERMDISP: *P* value = 0.80). For ARG, there were also resistome structural differences for both Bray-Curtis (**C**) (PERMDISP: *P* value < 0.0001) and compositional differences Sorensen (**D**) (PERMDISP: *P* value < 0.0001).

Beta diversity of ARG and MGE of the gut bacteria in pregnancy and infancy also differed. The community structure of MGE (Fig. 4A) and composition (Fig. 4B) differed significantly between infants and pregnant women’s resistomes. Similarly, community structure of ARG (Fig. 4C) and composition (Fig. 4D) differed significantly between pregnant women and infants.

**Fig 5:**
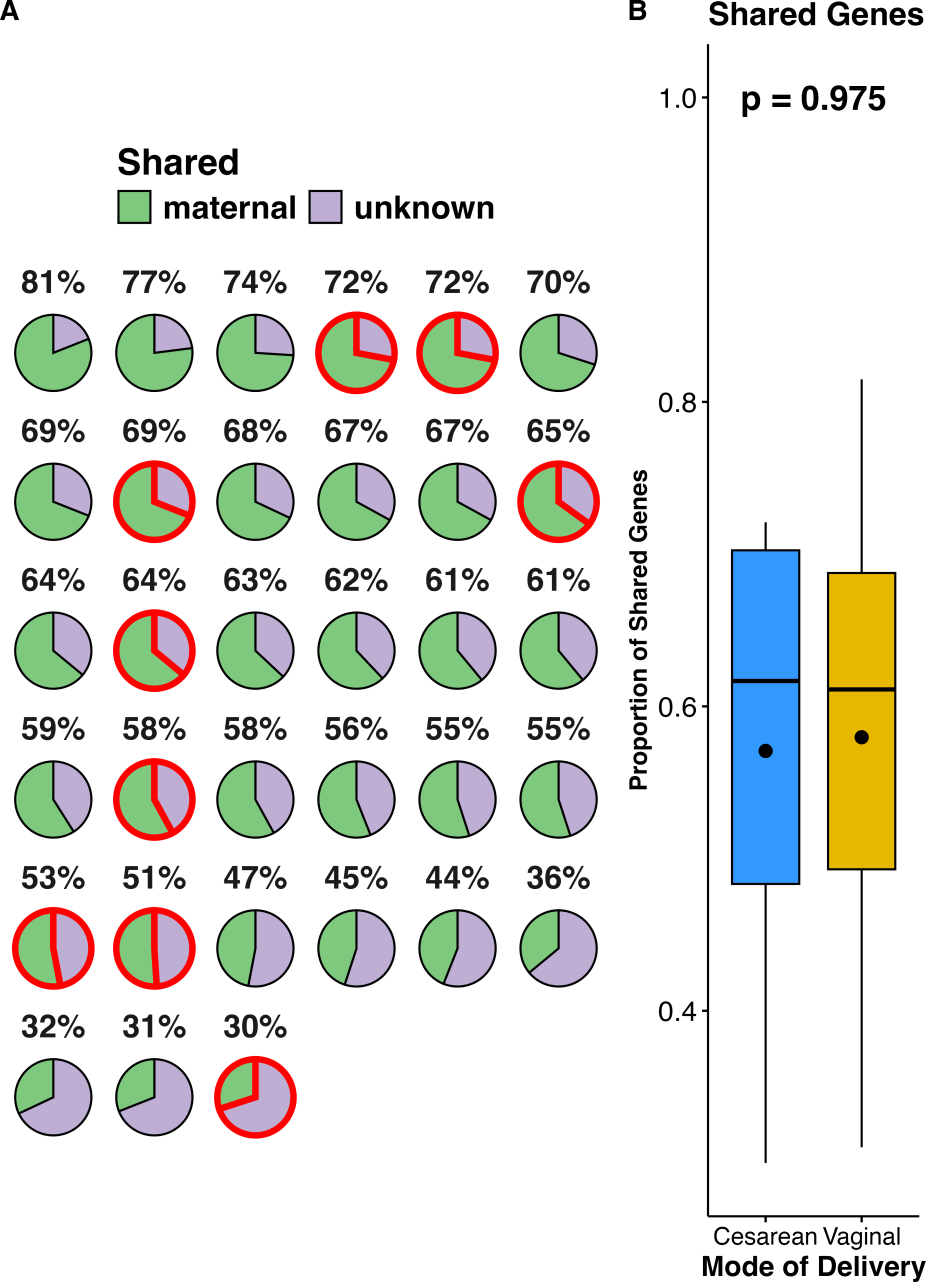
Pie charts (**A**) of ARG shared in the gut resistomes of dyads (infants and pregnant women). Each pie represents the resistome of one of the infants (*n* = 33). Maternal (green) represents the percentage of genes shared with the pregnant women, while unknown (lavender) represents ARG not found in the infant’s mother’s sample. Red outlines indicate infants born via cesarean section (*n* = 9). The shared proportion of genes with their mother’s (**B**) for cesarean (blue, *n* = 9) versus vaginally born infants (gold, *n* = 24) was not significantly different (Wilcoxon *P* value = 0.975). The horizontal line in the center of the box and whisker plot represents the median, while the upper and lower limits of the box represent the interquartile range (IQR). The whiskers are determined by Q1/Q3 ± 1.5 × IQR and anything falling outside of the whiskers represents an outlier. The black dot in the center of each of the boxplots is the mean.

Matched pregnant women and infants showed similar trends in alpha and beta diversity as un-paired samples. There were (*n* = 33) dyads that were available for analysis. On average, dyad pairs shared approximately 59% (range: 30–81%) of their ARG (Fig. 5A). The proportion of shared genes between mother and infant was similar for vaginally versus cesarean-born infants (Fig. 5B). Matched infants and pregnant women had similar differences in alpha diversity (Fig. S2) and beta diversity (Fig. S3) as unpaired pregnant women and infants. However, in paired samples, the resistomes of infants had a greater richness of ARG than pregnant women (Fig. S2A), which was inverse to what was observed in unpaired samples (Fig. 2A). ARG and MGE PCoA axis 1 distances patterns (Fig. S4 through S7) showed that infants’ resistomes were more like those of other infants than those of their own mother’s during pregnancy. Specific genes were present in nearly all dyads (Table S5), including *tetW* (protection and tetracycline)*, mefA* (efflux and MLSB), and *tnpA* (transposase and MGE), all of which were present in over 90% of dyad samples.

### Infant’s ARG composition and structure are associated with infant and maternal characteristics

**Fig 6:**
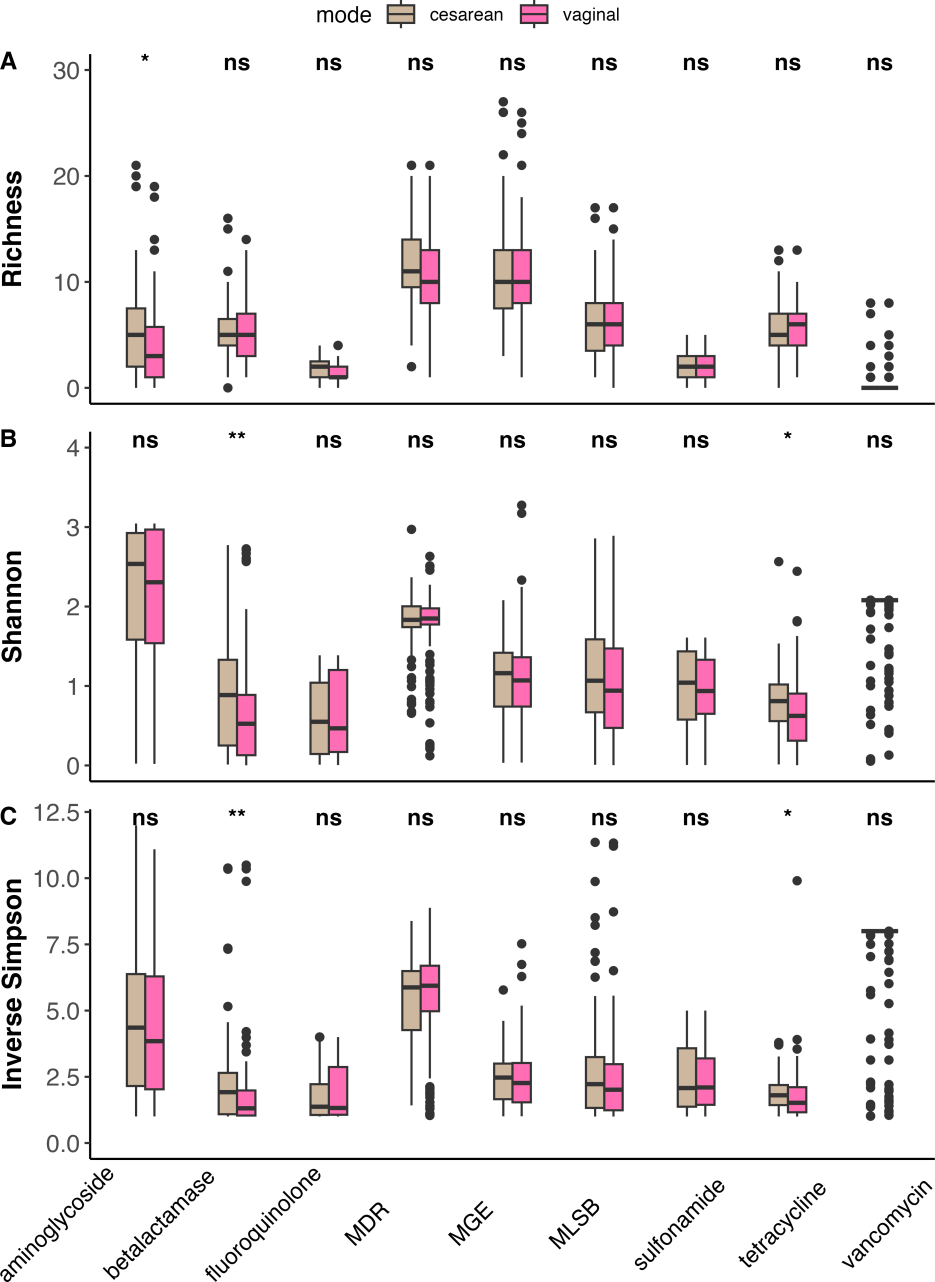
Box and whisker plot of richness (**A**), Shannon diversity (**B**), and Inverse Simpson (**C**) by ARG class based on method of delivery for infants. Cesarean-born infants (*n* = 71) are in brown, and vaginally born infants (*n* = 134) are in pink. The horizontal line in the center of the box and whisker plot represents the median, while the upper and lower limits of the box represent the interquartile range (IQR). The whiskers are determined by Q1/Q3 ± 1.5 × IQR and anything falling outside of the whiskers represents an outlier. Statistical significance is indicated by NS for non-significant (*P* ≥ 0.05). *P < 0.05, and **P < 0.01. Cesarean-born infants had a greater richness of aminoglycoside ARG (**A**) and a greater diversity for beta-lactamase (**B and C**) and tetracycline (**B and C**) ARG than infants born vaginally.

Infant bacterial ARG was compared based on their characteristics and their mother’s demographics extracted from birth certificate and sample questionnaire data (*n* = 202 infants). Univariate analysis revealed associations between resistome (ARG or MGE) composition and several factors, most notably infant’s mother’s race (Fig. S8 and S9, Table S6) and mode of delivery (Fig. 6). Infants with non-white mothers had a greater richness of ARG (Fig. S8A) and MGE (Fig. S8D) as well as greater diversity of MGE (Fig. S8E) than infants with white mothers. There were also differences in beta diversity between infants with white and non-white mothers (Fig. S9) based on community structure and composition of MGE and ARG as calculated by Bray-Curtis (Fig. S9A and C) and Sorensen dissimilarity (Fig. S9B and D). For the method of delivery (Fig. 6), cesarean-born infants had a greater richness of aminoglycoside ARG (Fig. 6A, richness: *P* < 0.05) and a greater diversity of beta-lactamase and tetracycline ARG (Shannon [Fig. 6B], Inverse Simpson [Fig. 6C]: *P* < 0.05) than infants born vaginally. There were significant differences in the resistome communities of infants born vaginally versus via cesarean section (Table S6) by community structure and composition of ARG and MGE.

Alpha diversity in the infancy resistome was associated with the infant’s mother’s pre-pregnancy BMI, and if an infant’s mother had ever smoked (Table S6). There were significant differences in the richness of aminoglycoside (richness: *P* < 0.05) and MDR (richness: *P* < 0.05) ARG present in obese, overweight, and normal BMI women’s infants resistomes (Fig. S10). Upon post hoc analysis, the infants born to individuals who had obesity prior to becoming pregnant had significantly higher richness of aminoglycoside ARG (Dunn test: *P* < 0.05) than infants born to individuals of normal weight prior to pregnancy and higher richness of MDR (Dunn test: *P* < 0.05) ARG than infants born to individuals who were overweight prior to pregnancy. Beta diversity of ARG classes did not differ by pregnant women’s pre-pregnancy BMI. The richness of ARG and MGE (richness: *P* < 0.001) and the diversity of MGE (Shannon, Inverse Simpson: *P* < 0.001) in infants whose mothers had previously smoked (*n* = 21) was greater than those whose mothers had never smoked (*n* = 179). Those whose mothers had smoked had increased richness of beta-lactamase, fluoroquinolone, MDR, MGE, MLSB, sulfonamide, and tetracycline ARG (data not shown). Community structure and composition also differed between these groups (Table S6).

Infants whose mothers had intrapartum antibiotic prophylaxis (IAP) (*n* = 41) for Group B *Streptococcus* (GBS) had increased diversity of aminoglycoside ARG (Inverse Simpson: *P* < 0.005) (Fig. S11). Those whose mothers did not have IAP (*n* = 151) had increased diversity of fluoroquinolone ARG (Inverse Simpson: *P* < 0.005). Due to limited data availability, further analysis of antibiotic exposure during pregnancy was not continued. Infants who had taken antibiotics themselves anytime since birth (Fig. S12) up to 3 months of age (*n* = 28) versus those who had not (*n* = 163) did not have significantly different gut resistomes overall but did have increased diversity of beta-lactamase ARG (Inverse Simpson: *P* < 0.05). Those who had not had antibiotics since birth had an increased diversity of fluoroquinolone ARG (Inverse Simpson: *P* < 0.05).

### Infant diet as a driver of variance for ARG and MGE richness and diversity

**Fig 7:**
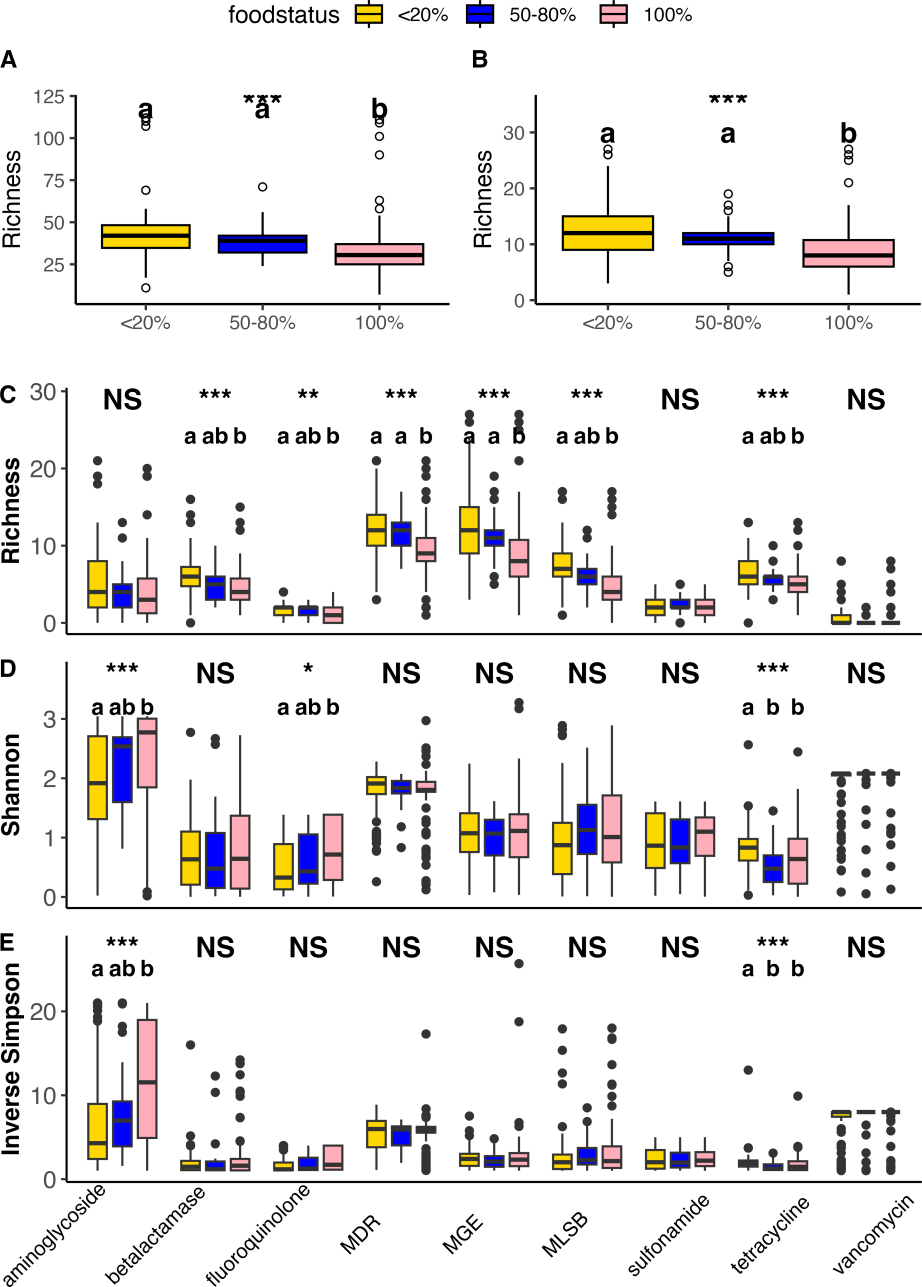
Box and whisker plot of richness of overall ARG (**A**) and MGE (**B**), and by ARG class for richness (**C**) Shannon (**D**), and Inverse Simpson (**E**), for infant stool samples. Samples are grouped by percent of infant diet consisting of breast milk. Those consuming a diet of <20% breast milk are in gold (*n* = 80), those consuming a diet of 50–80% breast milk are in blue (*n* = 26), and those consuming a diet of 100% breast milk are in pink (*n* = 86). The horizontal line in the center of the box and whisker plot represents the median, while the upper and lower limits of the box represent the interquartile range (IQR). The whiskers are determined by Q1/Q3 ± 1.5 × IQR and anything falling outside of the whiskers represents an outlier. Statistical significance is indicated by NS for non-significant (*P* ≥ 0.05). *P < 0.05, **P < 0.01, and ***P < 0.001. For classes with significant Kruskal-Wallis *P* values, Dunn tests were performed, and results are indicated with letters above the boxplots. Boxplots with different letters indicate significant differences, while boxplots with shared letters indicate no significant differences. Infants consuming <20% breast milk as part of total diet had a greater richness of ARG (**A**) (Dunn test: *P* < 0.0001) and (**B**) MGE (Dunn test: *P* value < 0.0001) than infants consuming 100% breast milk. Infants consuming between 50% and 80% of total diet as breast milk had greater richness (**A**) of ARG (Dunn test: *P* value = 0.002) and (**B**) MGE (Dunn test: *P* value = 0.001) than those consuming 100% breast milk. Overall, there was not a difference in richness of ARG and MGE between infants consuming a <20% and 50–80% breast milk diet, and there was no overall difference in diversity between groups. Infants consuming <20% breast milk as part of their diet had greater richness (**C**) of beta-lactamase, fluoroquinolone, MDR, MGE, MLSB, and tetracycline than infants consuming a 100% breast milk diet (Dunn test: *P* value < 0.05). Additionally, infants consuming a 50–80% breast milk diet had a greater richness (**C**) of MDR and MGE than infants consuming 100% breast milk (Dunn test: *P* < 0.05). Infants consuming a 100% breast milk diet had an increased diversity (**D and E**) of aminoglycoside (Dunn test: *P* value < 0.001) and fluoroquinolone (Dunn test: *P* value = 0.04) ARG relative to 20% breast milk diet infants, who had a greater diversity (**D and E**) of tetracycline ARG relative to infants consuming 50–80% or 100% breast milk (Dunn test: *P* value < 0.001).

Infants were separated into diet categories based on percentage of human milk as part of total dietary intake (consuming breast milk as less than <20% of total diet, breast milk as 50–80% of the diet, or 100% breast milk diet). The richness of ARG and MGE type was significantly different between breastfeeding groups (Fig. 7; Table S6, Fig. S13, richness: *P* < 0.0001) and by ARG class (Fig. 7). Infants consuming <20% breast milk had an increased richness of overall ARG and MGE (Fig. 7A and B), and beta-lactamase, fluoroquinolone, MDR, MLSB, and tetracycline ARG relative to the 100% breastfed group (Dunn test: *P* < 0.05) (Fig. 7C). There were also differences in richness of ARG by class (Fig. 7C) between the 50–80% breastfed infants and the 100% breast milk infants, with the former having increased richness of ARG and MDR by class (Dunn test: *P* < 0.05). There was increased diversity (Fig. 7D and E) of aminoglycoside and fluoroquinolone ARG in the 100% breast milk diet infants relative to the <20% group. Furthermore, there were differences in the beta diversity of the gut resistomes of ARG and MGE within infant’s resistome communities for the different dietary groups (Table S6).

### Multivariate analysis and hierarchical clustering

Alpha diversity was assessed using multivariate models (Table S7 and S9) for associations between mode of delivery, race of infant’s mother, antibiotic exposure of the infant, and feeding patterns of the infant (exclusively breastfed vs non-exclusively breastfed), and resistome composition. No variables were significant for predicting richness (Chao). Race and feeding pattern were only significant in predicting alpha diversity within univariate linear regression models. Mode of delivery was significant for predicting increased diversity (Shannon and Inverse Simpson) across both univariate and multivariate models of ARG. Cesarean-born infants were found to have a greater diversity (Shannon and Inverse Simpson) of ARG than vaginally born infants (Table S8).

The association between beta diversity and mode of delivery, antibiotic exposure, race of the mother, and feeding pattern was also assessed in multivariable models (Table 3). Mode of delivery and feeding pattern were significantly associated with community ARG structure and composition, whereas race was only associated with community structure of ARG.

**TABLE 3:** Multivariate analysis assessing the association between beta diversity of ARG and infant and maternal characteristics

Beta diversity model	Variable	*P* value	*R* ^2^	*F*-Statistic
Sorensen	Overall	0.0001^*a*^	0.06	3.18
	Mode of delivery	0.01^*a*^	0.01	2.16
	Antibiotic exposure	0.27	0.01	1.21
	Race	0.06	0.01	1.73
	Feeding pattern	0.0001	0.03	5.85
Bray-Curtis	Overall	0.0001^*a*^	0.06	2.88
	Mode of delivery	0.0001^*a*^	0.03	5.20
	Antibiotic exposure	0.29	0.01	1.13
	Race	0.04^*a*^	0.01	1.87
	Feeding pattern	0.0009^*a*^	0.02	3.18

^*a*^ significant* P*-values.

To determine if infant resistomes cluster based on normalized gene abundance (ARG and MGE), hierarchical clustering was performed (Fig. S14) on 3-month-old infants (*n* = 212) and three distinct clusters were identified (Fig. S15). Multinomial logistic regression, based on the clustering groups identified via hierarchical clustering analysis, was used to determine if mode of delivery, infant antibiotic exposure within the first 3 months of age, and whether the infants had been exclusively versus non-exclusively breastfed contributed to the clustering. Mode of delivery and feeding pattern were identified as significant predictors within this multinomial analysis. For mode of delivery, the relative risk ratio (RR) for being born vaginally vs. cesarean section was 0.44 for being in cluster 2 versus cluster 1. For feeding pattern, the relative RR for being FF versus BF was 0.269 for being in cluster 2 versus cluster 1 (Table S9). This meant that infants born vaginally were less likely to be in cluster 2, and infants that were FF were also less likely to be in cluster 2 than those who were exclusively breastfed.

## DISCUSSION

There is currently an incomplete understanding of how different risk factors contribute to the development of the resistome within the first few months of life, a critical period for the establishment of the microbiome which influences subsequent health outcomes. Several studies have shown that early-life exposures impact the resistome (12, 14), including our prior work describing resistomes of 6-month-old infants from the ARCH_GUT_ cohort (4, 22). Herein, the resistomes of 3-month-old infants and pregnant women are described, and participant characteristics associated with the resistome composition are presented. Pregnant women were found to have a greater richness of ARG, while infants were found to have a greater diversity of ARG based on Shannon and Inverse Simpson diversity indices within their resistomes. Several factors were associated with the composition of ARG within the gut bacterial communities of this diverse cohort of 3-month-old infants, including race of infant’s mother, mode of delivery, and percent of breast milk consumed as part of the infant diet. Mode of delivery was significant across univariate and multivariate models of alpha and beta diversity, with cesarean-born infants having a greater diversity of ARG relative to vaginally born infants. In our multivariate model for 3-month-old infants, cesarean-born infants had higher ARG alpha diversity (Shannon and Inverse Simpson indices) compared to vaginally born infants. Furthermore, the race of infant mother, mode of delivery, and infant feeding all remained associatd with compositional differences in multivariate beta diversity analyses. Additionally, any formula consumption by the infant was associated with increased richness and diversity of ARG, in multivariate analysis. Our results demonstrate strong associations between demographic and environmental factors with AMR and indicate potential areas for future intervention, such as mode of delivery and infant diet.

Infants and pregnant women were found to have markedly different resistome communities. The gut bacterial communities of pregnant women were observed to have greater richness of ARG present, but infants had a greater diversity of observed MGE and ARG (Fig. 2). Given the exposure of pregnant women throughout their lifetime to a wide range of antibiotics, it is expected that they would have a greater diversity and abundance of ARG present, which coincides with our findings. Based on alpha diversity analysis, the gut bacterial communities of pregnant women had a greater richness of aminoglycoside, MLSB, tetracycline, and vancomycin ARG (richness), and an increased diversity of genes conferring resistance to tetracycline (Table S4). The presence of vancomycin resistance in pregnant women is especially concerning given that vancomycin is administered as a last resort for GBS (36). Conversely, infants had an increased richness of AMR conferring resistance to fluoroquinolones and sulfonamides, and an increased diversity of genes which confer resistance to aminoglycosides, beta-lactamase, and vancomycin (Table S4; Fig. 3). The increased diversity of genes conferring beta-lactam resistance in infants is troubling given that beta-lactams are one of the common treatments for GBS and thus are key to preventing transmission of GBS from pregnant women to infants during delivery (2, 4, 37). Several factors could be contributing to the increased diversity of the infant resistome, including the early composition and diversity of the infant gut microbiome itself (19, 38). One main contributor to ARG in the early infant resistome is *Escherichia coli*, and that specific bacterial taxa has been identified as a carrier for many ARG (9, 39, 40). Due to this inherent resistance, many ARGs are not an immediate concern (38, 41). However, in populations of infants, who have increased ARG loads and underdeveloped immune systems, it is necessary to understand how diversity of ARG can lead to resistant infections and proliferation of ARG (38). Further examination of the relationship between the pregnancy and infancy gut microbiome and ARG composition in the context of clinical outcomes is needed to determine how resistome composition contributes to resistant infections.

An exploratory component of this research was investigating the extent of shared ARG within dyads of pregnant women and their infants. Previous studies have provided evidence for vertical transmission of ARG (4, 10, 17, 42–44); however, further research is needed to elucidate the proportion of ARG being passed from pregnant women to their infants during birth and early life. We observed that within the dyads of our study, infants had greater richness and diversity of ARG and greater diversity of MGE than their mothers and identified several genes that were present in nearly all dyads. Infant resistome communities were more like other infants (Table S4 through S7) than their mothers, indicating that factors outside of vertical transmission contribute to ARG development within the first 3 months of age (4). However, other studies have detected strain level transfer events and have identified family-specific or shared ARG (5), indicating that familial acquisition of ARG is occurring. Within the dyads of our study, tetracycline ARGs were prevalent in ~90% of the dyad pairs (Table S5). A larger cohort of dyads is necessary to determine the extent of similarity between mother and infant resistome communities.

While examining environmental factors contributing to ARG in infant resistomes, mode of delivery was found to be the most heavily associated, with differences in ARG composition being detected within univariate and multivariate analyses and for predicting sample clustering. Mode of delivery has been established as a major influencer of the composition of the gut microbiome for at least the first year of life (2, 45). We observed significant differences in the composition of the resistome between vaginally born infants and cesarean-born infants at 3 months of age (Fig. 6; Table S6). Cesarean-born infants were observed to have a greater richness of aminoglycoside ARG and greater diversity of beta-lactamase and tetracycline ARG relative to vaginally born infants, which coincides with previous identification of increased ARG in cesarean-born infants (46). Furthermore, the mode of delivery remained significant in the multivariate model (Table S7) for predicting increased diversity of ARG for cesarean-born infants compared to those born vaginally. Although the influence of mode of delivery on resistance appears to decrease over time (4, 14, 40), it is clearly important in the initial seeding of the early infant resistome, as observed within our results (47).

We identified clear differences in resistome composition between infants born to white women and non-white women. We observed that infants born to non-white pregnant women had increased richness of ARG and MGE as well as increased diversity of MGE and distinct resistome communities (Fig. S8 and S9). Previous studies have reported differences in the microbiome in ethnic and racial groups; however, it is difficult to tease apart the nuances of what differences in AMR gene composition are truly due to racial differences versus a multitude of other factors, including socioeconomic status, diet, location, and more (48, 49). Incorporation of other social determinants of health should be utilized within future multivariate analysis of AMR to determine drivers of resistome composition between racial groups.

Part of the interest in investigating the impact of diet composition on ARG is the modifiable nature of the diet. Specifically, assessing the differences between formula and breast-fed infants gives key insights into the role of diet as a protective (or risk) factor for AMR. We chose to evaluate infants consuming a mixed formula and breast milk diet by grouping infants into a third category (percent of total diet consisting of breast milk being between 50% and 80%) to determine if resistome composition was more similar between formula-fed or breastfed infants and those consuming a mixed diet (50). Infants consuming less than 20% breast milk as part of their total diet had a greater overall richness of ARG, but infants consuming a 100% breast milk diet had greater diversity of specific classes of ARG (Fig. 7). For multivariate analysis, infants were pooled into two categories: exclusively breastfed versus any formula, and the latter was found to be predictive of increased Chao and Inverse Simpson diversity (Table S7). As observed in the multinomial logistic regression analysis, having any formula was predictive of AMR/MGE clustering identified, with formula-fed infants more likely to be found in cluster 1 than cluster 2 (Table S9). The increased variety of ARG in formula-fed infant resistomes could be due in part to the microbial development of formula-fed infants, who typically develop diverse gut microbial communities like those of adult faster than breastfed infants (11, 51, 52). Suggestions for lowering ARG in the resistomes of infants include altering the pregnancy and lactation diets of women, including increasing fiber and decreasing fat intake to decrease AMR transmission via breast milk (53–55). Within our previous work, we have identified the introduction of solid foods as a potential area of ARG intervention (4). The timing and type of foods introduced at weaning is an important future direction for this line of research, such as introducing more phylogenetically diverse fiber sources into formula-fed infants' diets earlier to lower ARG burden (8, 11). Understanding how infant diets direct gut ARG composition will help to elucidate the mechanisms by which ARG reservoirs are created, and in turn, how to mitigate the formation of such reservoirs of resistance.

An original aim of this study was to analyze how treatment with specific classes of antibiotics contributes to the richness of ARG. Other studies have identified the role of select antibiotics in creating ARG reservoirs (56). Over 20% of the infants in our study were born to pregnant women given antibiotics for GBS during delivery (Table S2), and 14% of infants received antibiotics themselves during the first 3 months of life (Table 1). These values may be an underrepresentation of the actual number of individuals receiving antibiotics, as other reports suggest that up to 70% of infants receive antibiotics within the first year of life and up to 30% of women are exposed to antibiotics during delivery for GBS (2, 37, 57, 58). Tetracycline genes were the most prevalent overall, with *tetW* and *tetM* being found in nearly all samples and *tetQ* being found in all pregnant women’s samples (Table 2). Previous studies have reported vertical transmission of tetracycline resistance genes from pregnant women to the child resulting from that pregnancy (59, 60). Additionally, the abundance of the tetracycline gene *tetQ* has been shown to be lower in breast-fed infants compared to formula-fed infants (1). Within our study, administration of antibiotics for GBS impacted the diversity of ARG within the infant gut (Fig. S11), with infants born to pregnant women treated for GBS having increased diversity of aminoglycoside ARG, and those who had not an increased diversity of fluoroquinolone ARG. There was limited data of antibiotic type and class provided to participants based on available medical record abstraction. Due to this, it was difficult to assess how individual antibiotics given to women during pregnancy or to infants during the first 3 months of life influence ARG accumulation in the infant gut.

The methodology employed within this study has several strengths, including the utilization of highly parallel quantitative real-time PCR, which has been shown to detect ARG/MGE with high sensitivity (4, 26–29). Additionally, the utilization of the Takara SmartChip allowed for more robust identification of ARG and has been found to be precise in detecting rare ARG (4). A potential limitation of the study design was the decision to use RT-PCR instead of whole-genome or shotgun sequencing, which would have allowed detection of novel ARG. Other proposed methodologies, such as utilizing Hi-C technology, could provide further insights in linking strain-specific bacteria to ARG (61). Another limitation of using PCR is that all detected ARGs were previously identified, which prevents the discovery of additional ARGs that were omitted from the qPCR panel. Furthermore, insufficient data collection of antibiotic use and exposure limited our ability to detect differences in the role that antibiotic exposure plays in AMR. By only assessing fecal samples, we limit the scope of the claims we can make regarding the mode of transmission of ARG, as breast milk, environmental, and mode of delivery via vaginal canal are all potential methods of ARG spread from pregnant women to infant (10, 45, 52, 54, 62). Finally, as our results were observational, we can only draw associations, and future research is necessary to determine the cause and the taxonomic identities of bacteria harboring the ARGs.

### Conclusions

In this prospective, longitudinal, cohort study, pregnant women in their third trimester and infants at 3 months of age had key differences in resistome composition. Consumption of any formula in the infant diet and delivery via cesarean section were identified as factors associated with increased richness and diversity of ARG. Future directions include assessing maternal dietary patterns during pregnancy in relation to her gut resistome composition. Additionally, comparisons between maternal and infant gut resistome development over time could be strengthened with longitudinal analysis across multiple time points. Furthermore, additional studies are needed to assess the impact of specific prenatally administered antibiotics on the AMR reservoir in young infants.

## SUPPLEMENTAL MATERIAL

Supplemental Figures and Tables Part 1 msphere.00445-25-s0001.pdf Figures S1 through S10 and Tables S2 through S6.

Supplemental Figures and Tables Part 2 msphere.00445-25-s0002.pdf Figures S11 through S15 and Tables S7 through S9.

Data File msphere.00445-25-s0003.csv Data used in the analyses presented in the paper.

Code msphere.00445-25-s0004.pdf R code used to analyze data.

Data Directory msphere.00445-25-s0005.docx Information to understand the Data File in the supplemental material.

Table S1 msphere.00445-25-s0006.pdf Genes assayed and their functional classification.

ASM does not own the copyrights to Supplemental Material that may be linked to, or accessed through, an article. The authors have granted ASM a non-exclusive, world-wide license to publish the Supplemental Material files. Please contact the corresponding author directly for reuse.

## References

[1] Dadgostar P. 2019. Antimicrobial resistance: implications and costs. Infect Drug Resist 12:3903–3910. doi:10.2147/IDR.S234610 31908502 PMC6929930

[2] Samarra A, Esteban-Torres M, Cabrera-Rubio R, Bernabeu M, Arboleya S, Gueimonde M, Collado MC. 2023. Maternal-infant antibiotic resistance genes transference: what do we know? Gut Microbes 15. doi:10.1080/19490976.2023.2194797 PMC10078139 37020319

[3] Gasparrini AJ, Crofts TS, Gibson MK, Tarr PI, Warner BB, Dantas G. 2016. Antibiotic perturbation of the preterm infant gut microbiome and resistome. Gut Microbes 7:443–449. doi:10.1080/19490976.2016.1218584 27472377 PMC5154371

[4] Sosa-Moreno A, Comstock SS, Sugino KY, Ma TF, Paneth N, Davis Y, Olivero R, Schein R, Maurer J, Zhang L. 2020. Perinatal risk factors for fecal antibiotic resistance gene patterns in pregnant women and their infants. PLoS One 15:e0234751. doi:10.1371/journal.pone.0234751 32555719 PMC7302573

[5] Yassour M, Jason E, Hogstrom LJ, Arthur TD, Tripathi S, Siljander H, Selvenius J, Oikarinen S, Hyöty H, Virtanen SM, Ilonen J, Ferretti P, et al.. 2018. Strain-level analysis of mother-to-child bacterial transmission during the first few months of life. Cell Host Microbe 24:146–154. doi:10.1016/j.chom.2018.06.007 30001517 PMC6091882

[6] Van Daele E, Knol J, Belzer C. 2019. Microbial transmission from mother to child: improving infant intestinal microbiota development by identifying the obstacles. Crit Rev Microbiol 45:613–648. doi:10.1080/1040841X.2019.1680601 31859540

[7] Crits-Christoph A, Hallowell HA, Koutouvalis K, Suez J. 2022. Good microbes, bad genes? The dissemination of antimicrobial resistance in the human microbiome. Gut Microbes 14:2055944. doi:10.1080/19490976.2022.2055944 35332832 PMC8959533

[8] Oliver A, Xue Z, Villanueva YT, Durbin-Johnson B, Alkan Z, Taft DH, Liu J, Korf I, Laugero KD, Stephensen CB, Mills DA, Kable ME, Lemay DG. 2022. Association of diet and antimicrobial resistance in healthy U.S. adults. mBio 13:e0010122. doi:10.1128/mbio.00101-22 35536006 PMC9239165

[9] Lebeaux RM, Coker MO, Dade EF, Palys TJ, Morrison HG, Ross BD, Baker ER, Karagas MR, Madan JC, Hoen AG. 2021. The infant gut resistome is associated with E. coli and early-life exposures. BMC Microbiol 21:201. doi:10.1186/s12866-021-02129-x 34215179 PMC8252198

[10] Pärnänen K, Karkman A, Hultman J, Lyra C, Bengtsson-Palme J, Larsson DGJ, Rautava S, Isolauri E, Salminen S, Kumar H, Satokari R, Virta M. 2018. Maternal gut and breast milk microbiota affect infant gut antibiotic resistome and mobile genetic elements. Nat Commun 9:3891. doi:10.1038/s41467-018-06393-w 30250208 PMC6155145

[11] Pärnänen KMM, Hultman J, Markkanen M, Satokari R, Rautava S, Lamendella R, Wright J, McLimans CJ, Kelleher SL, Virta MP. 2022. Early-life formula feeding is associated with infant gut microbiota alterations and an increased antibiotic resistance load. Am J Clin Nutr 115:407–421. doi:10.1093/ajcn/nqab353 34677583 PMC8827105

[12] Lebeaux RM, Madan JC, Nguyen QP, Coker MO, Dade EF, Moroishi Y, Palys TJ, Ross BD, Pettigrew MM, Morrison HG, Karagas MR, Hoen AG. 2021. Impact of antibiotics to off-target infant gut microbiota and resistance genes in cohort studies. medRxiv. doi:10.1101/2021.11.02.21265394 PMC9659678 35568730

[13] Shao Y, Forster SC, Tsaliki E, Vervier K, Strang A, Simpson N, Kumar N, Stares MD, Rodger A, Brocklehurst P, Field N, Lawley TD. 2019. Stunted microbiota and opportunistic pathogen colonization in caesarean-section birth. Nature 574:117–121. doi:10.1038/s41586-019-1560-1 31534227 PMC6894937

[14] Busi SB, de Nies L, Habier J, Wampach L, Fritz JV, Heintz-Buschart A, May P, Halder R, de Beaufort C, Wilmes P. 2021. Persistence of birth mode-dependent effects on gut microbiome composition, immune system stimulation and antimicrobial resistance during the first year of life. ISME Commun 1:8. doi:10.1038/s43705-021-00003-5 36717704 PMC9723731

[15] Ravi A, Avershina E, Foley SL, Ludvigsen J, Storrø O, Øien T, Johnsen R, McCartney AL, L’Abée-Lund TM, Rudi K. 2015. The commensal infant gut meta-mobilome as a potential reservoir for persistent multidrug resistance integrons. Sci Rep 5:15317. doi:10.1038/srep15317 26507767 PMC4623605

[16] von Wintersdorff CJ, Wolffs PF, Savelkoul PH, Nijsen RR, Lau S, Gerhold K, Hamelmann E, Penders J. 2016. The gut resistome is highly dynamic during the first months of life. Future Microbiol 11:501–510. doi:10.2217/fmb.15.154 27064174

[17] Li W, Tapiainen T, Brinkac L, Lorenzi HA, Moncera K, Tejesvi MV, Salo J, Nelson KE. 2021. Vertical transmission of gut microbiome and antimicrobial resistance genes in infants exposed to antibiotics at birth. J Infect Dis 224:1236–1246. doi:10.1093/infdis/jiaa155 32239170 PMC8514186

[18] Tapiainen T, Koivusaari P, Brinkac L, Lorenzi HA, Salo J, Renko M, Pruikkonen H, Pokka T, Li W, Nelson K, Pirttilä AM, Tejesvi MV. 2019. Impact of intrapartum and postnatal antibiotics on the gut microbiome and emergence of antimicrobial resistance in infants. Sci Rep 9:10635. doi:10.1038/s41598-019-46964-5 31337807 PMC6650395

[19] Gibson MK, Wang B, Ahmadi S, Burnham C-AD, Tarr PI, Warner BB, Dantas G. 2016. Developmental dynamics of the preterm infant gut microbiota and antibiotic resistome. Nat Microbiol 1:16024. doi:10.1038/nmicrobiol.2016.24 27572443 PMC5031140

[20] Yassour M, Vatanen T, Siljander H, Hämäläinen A-M, Härkönen T, Ryhänen SJ, Franzosa EA, Vlamakis H, Huttenhower C, Gevers D, Lander ES, Xavier RJ. 2016. Natural history of the infant gut microbiome and impact of antibiotic treatments on strain-level diversity and stability behalf of the DIABIMMUNE study group HHS public access. Sci Transl Med 9:343–381. doi:10.1126/scitranslmed.aad0917 PMC5032909 27306663

[21] Ma T, Bu S, Paneth N, Kerver JM, Comstock SS. 2022. Vitamin D supplementation in exclusively breastfed infants is associated with alterations in the fecal microbiome. Nutrients 14:202. doi:10.3390/nu14010202 35011077 PMC8747039

[22] Haddad EN, Comstock SS. 2021. Archive for research in child health (ARCH) and baby gut: study protocol for a remote, prospective, longitudinal pregnancy and birth cohort to address microbiota development and child health. Methods Protoc 4:52. doi:10.3390/mps4030052 34449678 PMC8395764

[23] Haddad EN, Sugino KY, Kerver JM, Paneth N, Comstock SS. 2021. The infant gut microbiota at 12 months of age is associated with human milk exposure but not with maternal pre-pregnancy body mass index or infant BMI-for-age z-scores. Current Research in Physiology 4:94–102. doi:10.1016/j.crphys.2021.03.004 34136830 PMC8205433

[24] Sugino KY, Ma T, Kerver JM, Paneth N, Comstock SS. 2021. Human milk feeding patterns at 6 months of age are a major determinant of fecal bacterial diversity in infants. J Hum Lact 37:703–713. doi:10.1177/0890334420957571 32926654

[25] Stedtfeld RD, Guo X, Stedtfeld TM, Sheng H, Williams MR, Hauschild K, Gunturu S, Tift L, Wang F, Howe A, Chai B, Yin D, Cole JR, Tiedje JM, Hashsham SA. 2018. Primer set 2.0 for highly parallel qPCR array targeting antibiotic resistance genes and mobile genetic elements. FEMS Microbiol Ecol 94:fiy130. doi:10.1093/femsec/fiy130 30052926 PMC7250373

[26] Su JQ, Wei B, Ou-Yang WY, Huang FY, Zhao Y, Xu HJ, Zhu YG. 2015. Antibiotic resistome and its association with bacterial communities during sewage sludge composting. Environ Sci Technol 49:7356–7363. doi:10.1021/acs.est.5b01012 26018772

[27] Looft T, Johnson TA, Allen HK, Bayles DO, Alt DP, Stedtfeld RD, Sul WJ, Stedtfeld TM, Chai B, Cole JR, Hashsham SA, Tiedje JM, Stanton TB. 2012. In-feed antibiotic effects on the swine intestinal microbiome. Proc Natl Acad Sci USA 109:1691–1696. doi:10.1073/pnas.1120238109 22307632 PMC3277147

[28] Guo X, Stedtfeld RD, Hedman H, Eisenberg JNS, Trueba G, Yin D, Tiedje JM, Zhang L. 2018. Antibiotic resistome associated with small-scale poultry production in rural ecuador. Environ Sci Technol 52:8165–8172. doi:10.1021/acs.est.8b01667 29944836

[29] Zhu YG, Zhao Y, Li B, Huang CL, Zhang SY, Yu S, Chen YS, Zhang T, Gillings MR, Su JQ. 2017. Continental-scale pollution of estuaries with antibiotic resistance genes. Nat Microbiol 2:16270. doi:10.1038/nmicrobiol.2016.270 28134918

[30] Oksanen J, Blanchet FG, Friendly M, Kindt R, Legendre P, Mcglinn D, Minchin PR, O’hara RB, Simpson GL, Solymos P, Henry M, et al.. 2020. Package “vegan” title community ecology package. Version 2.5-7. http://CRAN.R-project.org/package=vegan

[31] Benjamini Y, Hochberg Y. 1995. Controlling the false discovery rate: a practical and powerful approach to multiple testing. Journal of the Royal Statistical Society Series B 57:289–300. doi:10.1111/j.2517-6161.1995.tb02031.x

[32] Zhang L, Forst CV, Gordon A, Gussin G, Geber AB, Fernandez PJ, Ding T, Lashua L, Wang M, Balmaseda A, Bonneau R, Zhang B, Ghedin E. 2020. Characterization of antibiotic resistance and host-microbiome interactions in the human upper respiratory tract during influenza infection. Microbiome 8:39. doi:10.1186/s40168-020-00803-2 32178738 PMC7076942

[33] Desagulier G. 2019. Validating clusters in hierarchical cluster analysis. Available from: https://corpling.hypotheses.org/2675

[34] Multinomial logistic regression | r data analysis examples. 2021 UCLA: Statistical Consulting Group. Available from: https://stats.oarc.ucla.edu/r/dae/multinomial-logistic-regression

[35] Michigan: 2020 Census. 2020. United States Census Bureau. Available from: https://www.census.gov/library/stories/state-by-state/michigan.html

[36] Hasperhoven GF, Al-Nasiry S, Bekker V, Villamor E, Kramer BWW. 2020. Universal screening versus risk-based protocols for antibiotic prophylaxis during childbirth to prevent early-onset group B streptococcal disease: a systematic review and meta-analysis. BJOG 127:680–691. doi:10.1111/1471-0528.16085 31913562 PMC7187465

[37] Stephens K, Charnock-Jones DS, Smith GCS. 2023. Group B Streptococcus and the risk of perinatal morbidity and mortality following term labor. Am J Obstet Gynecol 228:S1305–S1312. doi:10.1016/j.ajog.2022.07.051 37164497

[38] Theophilus RJ, Taft DH. 2023. Antimicrobial resistance genes (ARGs), the gut microbiome, and infant nutrition. Nutrients 15:3177. doi:10.3390/nu15143177 37513595 PMC10383493

[39] Gasparrini AJ, Wang B, Sun X, Kennedy EA, Hernandez-Leyva A, Ndao IM, Tarr PI, Warner BB, Dantas G. 2019. Persistent metagenomic signatures of early-life hospitalization and antibiotic treatment in the infant gut microbiota and resistome. Nat Microbiol 4:2285–2297. doi:10.1038/s41564-019-0550-2 31501537 PMC6879825

[40] Li X, Stokholm J, Brejnrod A, Vestergaard GA, Russel J, Trivedi U, Thorsen J, Gupta S, Hjelmsø MH, Shah SA, Rasmussen MA, Bisgaard H, Sørensen SJ. 2021. The infant gut resistome associates with E. coli, environmental exposures, gut microbiome maturity, and asthma-associated bacterial composition. Cell Host Microbe 29:975–987. doi:10.1016/j.chom.2021.03.017 33887206

[41] Zhang A-N, Gaston JM, Dai CL, Zhao S, Poyet M, Groussin M, Yin X, Li L-G, van Loosdrecht MCM, Topp E, Gillings MR, Hanage WP, Tiedje JM, Moniz K, Alm EJ, Zhang T. 2021. An omics-based framework for assessing the health risk of antimicrobial resistance genes. Nat Commun 12:4765. doi:10.1038/s41467-021-25096-3 34362925 PMC8346589

[42] Seale J, Millar M. 2014. Perinatal vertical transmission of antibiotic-resistant bacteria: a systematic review and proposed research strategy. BJOG 121:923–928. doi:10.1111/1471-0528.12746 24674346

[43] Gosalbes MJ, Vallès Y, Jiménez-Hernández N, Balle C, Riva P, Miravet-Verde S, de Vries LE, Llop S, Agersø Y, Sørensen SJ, Ballester F, Francino MP. 2016. High frequencies of antibiotic resistance genes in infants’ meconium and early fecal samples. J Dev Orig Health Dis 7:35–44. doi:10.1017/S2040174415001506 26353938

[44] Patangia DV, Ryan CA, Dempsey E, Stanton C, Ross RP. 2022. Vertical transfer of antibiotics and antibiotic resistant strains across the mother/baby axis. Trends Microbiol 30:47–56. doi:10.1016/j.tim.2021.05.006 34172345

[45] Selma-Royo M, García-Mantrana I, Calatayud M, Parra-Llorca A, Martínez-Costa C, Collado MC. 2020. Maternal microbiota, cortisol concentration, and post-partum weight recovery are dependent on mode of delivery. Nutrients 12:1779. doi:10.3390/nu12061779 32549282 PMC7353435

[46] Dohou AM, Buda VO, Yemoa LA, Anagonou S, Van Bambeke F, Van Hees T, Dossou FM, Dalleur O. 2022. Antibiotic usage in patients having undergone caesarean section: a three-level study in Benin. Antibiotics (Basel) 11:617. doi:10.3390/antibiotics11050617 35625261 PMC9137971

[47] Thänert R, Sawhney SS, Schwartz DJ, Dantas G. 2022. The resistance within: antibiotic disruption of the gut microbiome and resistome dynamics in infancy. Cell Host Microbe 30:675–683. doi:10.1016/j.chom.2022.03.013 35550670 PMC9173668

[48] Brooks AW, Priya S, Blekhman R, Bordenstein SR. 2018. Gut microbiota diversity across ethnicities in the United States. PLoS Biol 16:e2006842. doi:10.1371/journal.pbio.2006842 30513082 PMC6279019

[49] Borrello K, Lim U, Park SY, Monroe KR, Maskarinec G, Boushey CJ, Wilkens LR, Randolph TW, Le Marchand L, Hullar MA, Lampe JW. 2022. Dietary intake mediates ethnic differences in gut microbial composition. Nutrients 14:660. doi:10.3390/nu14030660 35277019 PMC8840192

[50] Madan JC, Hoen AG, Lundgren SN, Farzan SF, Cottingham KL, Morrison HG, Sogin ML, Li H, Moore JH, Karagas MR. 2016. Association of cesarean delivery and formula supplementation with the intestinal microbiome of 6-week-old infants. JAMA Pediatr 170:212–219. doi:10.1001/jamapediatrics.2015.3732 26752321 PMC4783194

[51] Stewart CJ, Ajami NJ, O’Brien JL, Hutchinson DS, Smith DP, Wong MC, Ross MC, Lloyd RE, Doddapaneni H, Metcalf GA, et al.. 2018. Temporal development of the gut microbiome in early childhood from the TEDDY study. Nature 562:583–588. doi:10.1038/s41586-018-0617-x 30356187 PMC6415775

[52] Ma J, Li Z, Zhang W, Zhang C, Zhang Y, Mei H, Zhuo N, Wang H, Wang L, Wu D. 2020. Comparison of gut microbiota in exclusively breast-fed and formula-fed babies: a study of 91 term infants. Sci Rep 10. doi:10.1038/s41598-020-72635-x PMC7519658 32978424

[53] Chu DM, Antony KM, Ma J, Prince AL, Showalter L, Moller M, Aagaard KM. 2016. The early infant gut microbiome varies in association with a maternal high-fat diet. Genome Med 8:77. doi:10.1186/s13073-016-0330-z 27503374 PMC4977686

[54] Lundgren SN, Madan JC, Emond JA, Morrison HG, Christensen BC, Karagas MR, Hoen AG. 2018. Maternal diet during pregnancy is related with the infant stool microbiome in a delivery mode-dependent manner. Microbiome 6:109. doi:10.1186/s40168-018-0490-8 29973274 PMC6033232

[55] Savage JH, Lee-Sarwar KA, Sordillo JE, Lange NE, Zhou Y, O’Connor GT, Sandel M, Bacharier LB, Zeiger R, Sodergren E, Weinstock GM, Gold DR, Weiss ST, Litonjua AA. 2018. Diet during pregnancy and infancy and the infant intestinal microbiome. J Pediatr 203:47–54. doi:10.1016/j.jpeds.2018.07.066 30173873 PMC6371799

[56] Rahman SF, Olm MR, Morowitz MJ, Banfield JF. 2018. Machine learning leveraging genomes from metagenomes identifies influential antibiotic resistance genes in the infant gut microbiome. mSystems 3:e00123-17. doi:10.1128/mSystems.00123-17 PMC5758725 29359195

[57] Santillan DA, Hubb AJ, Nishimura TE, Rosenfeld-O’Tool SR, Schroeder KJ, Conklin JM, Karras AE, Gumusoglu SB, Brandt DS, Miller E, Hunter SK, Santillan MK. 2022. Group B Streptococcus screening and treatment adherence in pregnancy: a retrospective cohort study and opportunities for improvement. AJPM Focus 1:100028. doi:10.1016/j.focus.2022.100028 37791233 PMC10546507

[58] Van Dyke MK, Phares CR, Lynfield R, Thomas AR, Arnold KE, Craig AS, Mohle-Boetani J, Gershman K, Schaffner W, Petit S, Zansky SM, Morin CA, et al.. 2009. Evaluation of universal antenatal screening for group B Streptococcus. N Engl J Med 360:2626–2636. doi:10.1056/NEJMoa0806820 19535801

[59] de Vries LE, Vallès Y, Agersø Y, Vaishampayan PA, García-Montaner A, Kuehl JV, Christensen H, Barlow M, Francino MP. 2011. The gut as reservoir of antibiotic resistance: microbial diversity of tetracycline resistance in mother and infant. PLoS One 6:e21644. doi:10.1371/journal.pone.0021644 21738748 PMC3125294

[60] Shoemaker NB, Vlamakis H, Hayes K, Salyers AA. 2001. Evidence for extensive resistance gene transfer among Bacteroides spp. and among Bacteroides and other genera in the human colon. Appl Environ Microbiol 67:561–568. doi:10.1128/AEM.67.2.561-568.2001 11157217 PMC92621

[61] Kent AG, Vill AC, Shi Q, Satlin MJ, Brito IL. 2020. Widespread transfer of mobile antibiotic resistance genes within individual gut microbiomes revealed through bacterial Hi-C. Nat Commun 11:4379. doi:10.1038/s41467-020-18164-7 32873785 PMC7463002

[62] Behari P, Englund J, Alcasid G, Garcia-Houchins S, Weber SG. 2004. Transmission of methicillin-resistant Staphylococcus aureus to preterm infants through breast milk. Infect Control Hosp Epidemiol 25:778–780. doi:10.1086/502476 15484804

