# Supplementary material for: Associations of diet, race, and other environmental factors with antimicrobial resistance genes in the gut bacterial communities of pregnant women and 3-month-old infants
Source: mSphere. 2025 Nov 24;10(12):e00445-25. doi: 10.1128/msphere.00445-25 (PMC12724134; doi:10.1128/msphere.00445-25)
Supplement: Supplemental Figures and Tables Part 1 — Figures S1 through S10 and Tables S2 through S6. [file msphere.00445-25-s0001.pdf]

Supplemental Figures and Tables Part 1.

Table S2. Characteristics of Infant's (n= 212) mothers.

Maternal Characteristics		
**Race, n(%)[a]**	Asian	3(1.4%)
	Black	40(19.5%)
	White	153(74.6%)
	Other	8(3.9%)
**Smoking Status, n(%)[b]**	Never smoked	179(89.5%)
	Ever smoked	21(10.5%)
**Pre-pregnancy BMI Category, n(%)[b]**	Normal	84(42%)
	Overweight	48(24%)
	Obese	68(34%)
**Pre-pregnancy BMI, mean(SD)[b]**	28.7(8.54)	
**Estimated weeks gestation, mean(SD)[a]**	38.37(1.92)	
**Antibiotics for Group B Strep during pregnancy, n(%)[c]**	Yes	41(21.3%)
	No	151(76%)
**Maternal Age, years mean(SD)**	30.85(5.43)	

a = missing data (n=8)

b= missing data (n=12)

c= missing data (n=20)

Table S3. ARG and MGE recorded in study participants.

	ARG, mean (min-max)	MGE, mean(min-max)
Overall	44 (7-112)	11(1-27)
Pregnant women	53 (11-81)	11(3-18)
Infant	40 (7-112)	11(1-27)

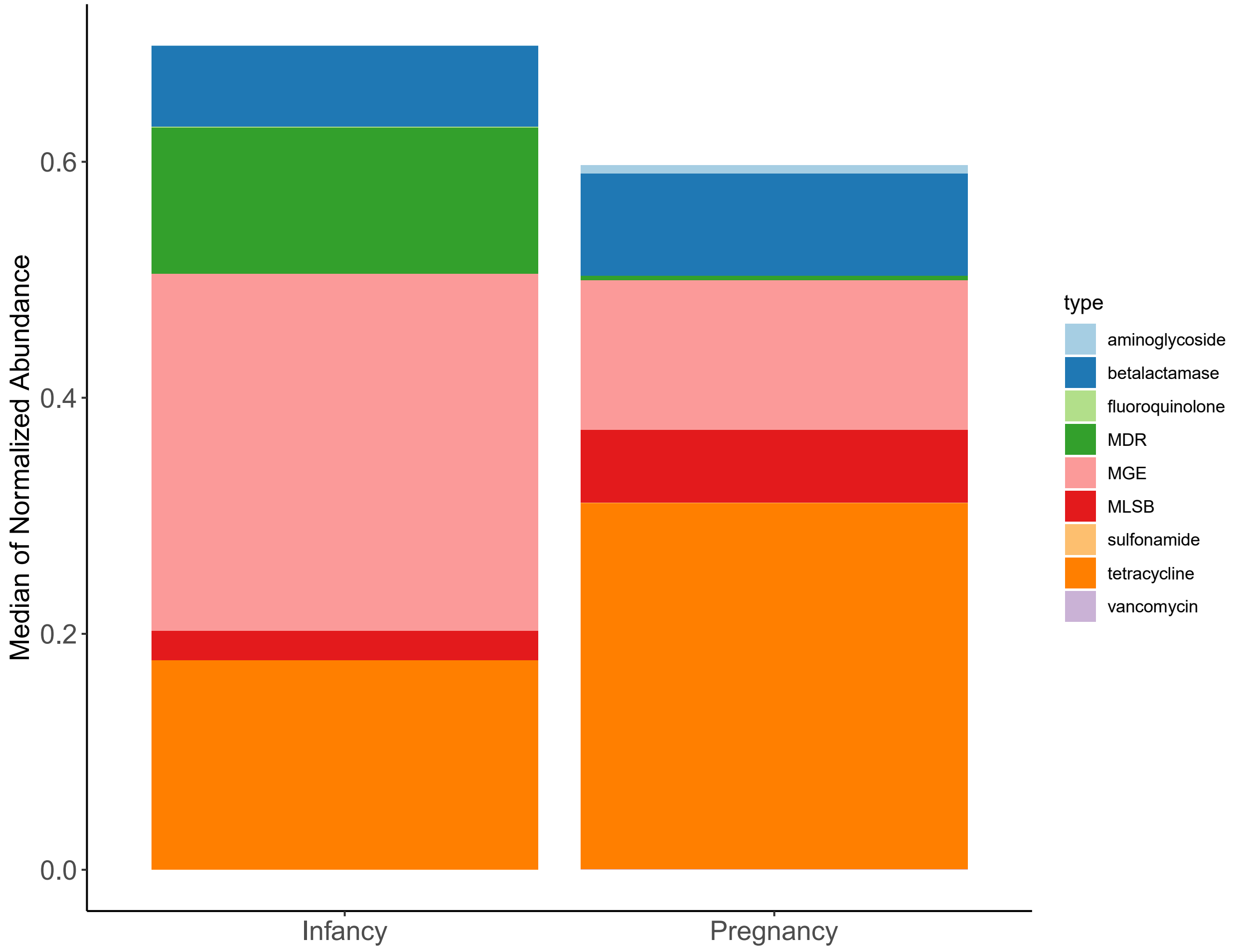

Figure S1. Median of normalized abundance of ARG in pregnant women and infant's samples by ARG class. Colors separate individual ARG class and are based on genes identified in DNA isolates from infant (n=212) and pregnant women (n=99) stool samples. There were significant differences in median of normalized abundance between pregnant women and infants between aminoglycoside (p-value < 0.0001), fluroquinolone (p-value < 0.0001), MDR (p-value <

0.0001), MGE (p-value = 0.001), MLSB (p-value = 0.006), tetracycline (p-value < 0.0001), sulfonamide (p-value < 0.0001), and vancomycin (p-value = 0.0004).

Table S4. The alpha diversity of the resistomes of infants and of pregnant women.

Alpha Diversity Metric	ARG Class	Pregnancy mean±SD	Infancy mean±SD	P-value*
Richness	ARG	53.26 ± 13.13	40.10 ± 19.19	< 0.0001
	Aminoglycoside	11.70 ± 3.49	4.92 ± 4.54	< 0.0001
	MLSB	9.23 ± 2.64	6.25 ± 3.50	< 0.0001
	Tetracycline	8.86 ± 1.65	5.86 ± 2.24	< 0.0001
	Vancomycin	2.90 ± 1.68	0.66 ± 1.79	< 0.0001
	Fluroquinolone	0.92 ± 0.68	1.55 ± 1.03	< 0.0001
	Sulfonamide	1.62 ± 1.23	2.10 ± 1.36	< 0.0001
Shannon Diversity	ARG	1.83 ± 0.52	1.99 ± 0.56	0.008
	Aminoglycoside	1.71 ± 0.42	2.13 ± 0.88	< 0.0001
	Beta-lactamase	0.31 ± 0.41	0.76 ± 0.73	< 0.0001
	Tetracycline	0.88 ± 0.30	0.70 ± 0.44	< 0.0001
	Vancomycin	1.42 ± 0.55	1.91 ± 0.44	< 0.0001
Inverse Simpson	ARG	4.63 ± 3.16	5.93 ± 3.62	0.01
	Aminoglycoside	4.63 ± 2.33	9.22 ± 7.10	< 0.0001
	Beta-lactamase	1.39 ± 1.38	2.40 ± 2.73	< 0.0001
	Tetracycline	2.12 ± 0.71	1.86 ± 1.14	< 0.0001
	Vancomycin	3.98 ± 2.21	7.07 ± 2.11	< 0.0001

***** = P-values represent Wilcoxon differences between infancy and pregnancy.

Note: only those alpha diversity metrics that differed significantly are included in the table.

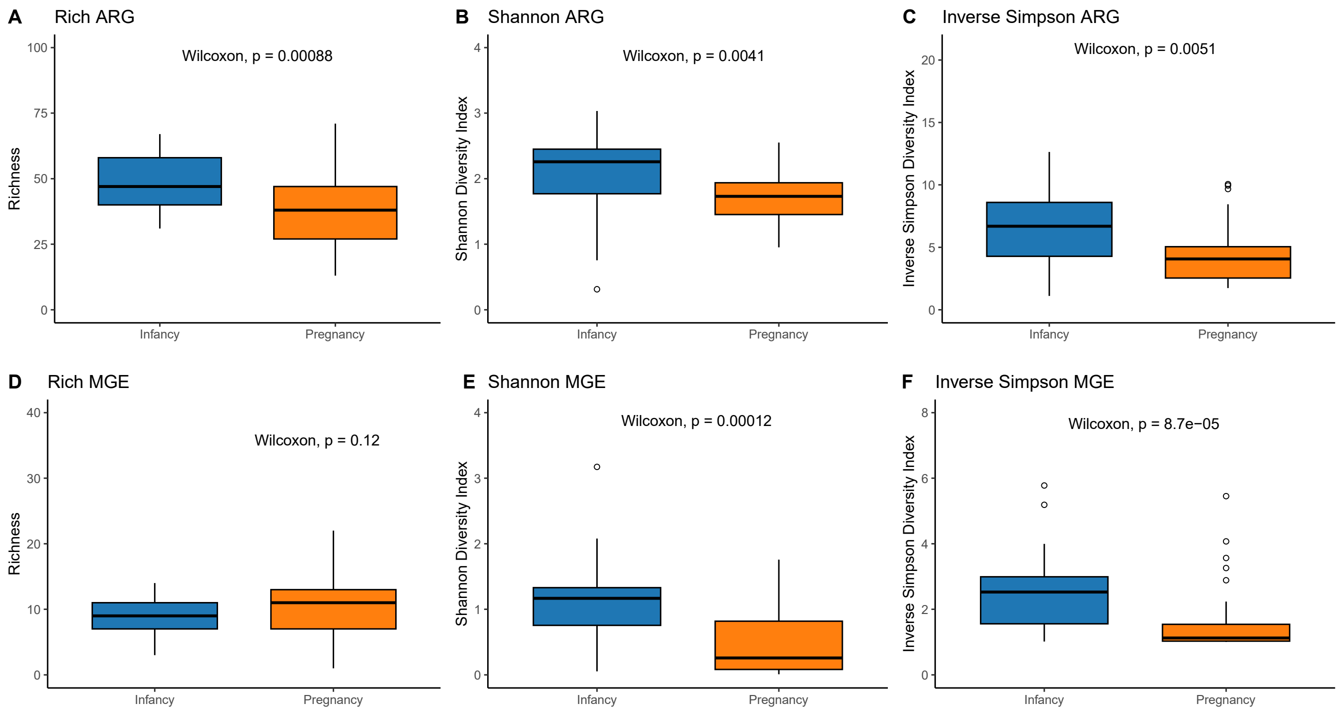

Figure S2. Box and whisker plot of alpha diversity of dyads Richness, Shannon, and Inverse Simpson of matched infants (n=33) and pregnant women (n=33). The horizontal line in the center of the box and whisker plot represents the median, while the upper and lower limit of the box represent the interquartile range (IQR). The whiskers are determined by Q1/Q3 +/- 1.5 * IQR and anything falling outside of the whiskers represents an outlier. The gut bacterial communities of infants had greater richness of ARG (A) but not MGE (D). Infants had greater diversity of ARG (B, C) and MGE (E, F).

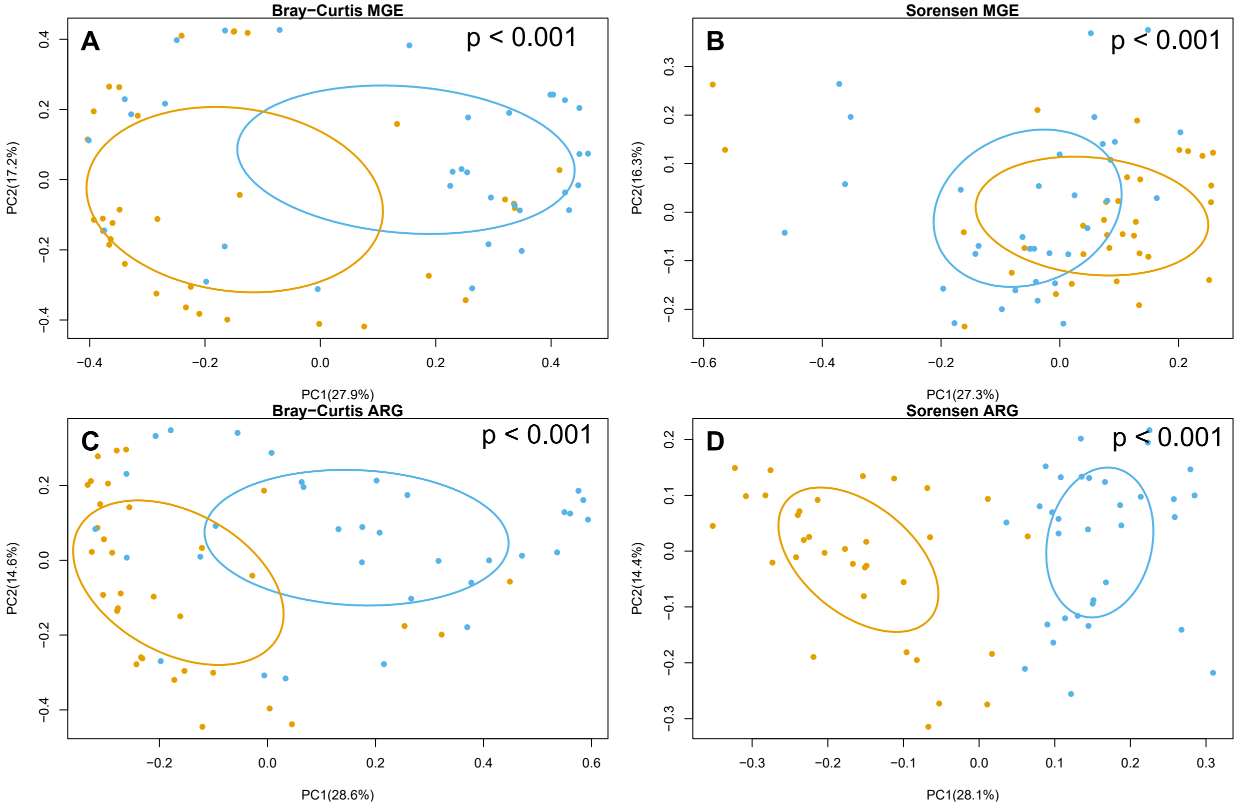

Figure S3. Dyads of matched infants and pregnant women had distinctive resistome communities. Red dots represent pregnant women, while black dots represent infants. Each dot represents an individual infant or pregnant women, while ellipses are based on centroids of each group. Axis percentage accounts for percentage of variation explained by that given axis. The further apart each dot, the greater the dissimilarity. There were community structural differences based on PCoA Bray-Curtis dissimilarity for MGE (A) (PERMDISP: p-value = 0.01), and compositional differences based on Sorensen dissimilarity (B) (PERMDISP: p-value < 0.0001). For ARG, there were also resistome structural differences between matched dyad samples, for Bray-Curtis (C) (PERMDISP: 0.94) and compositional differences based on Sorensen (D) (PERMDISP: 0.02).

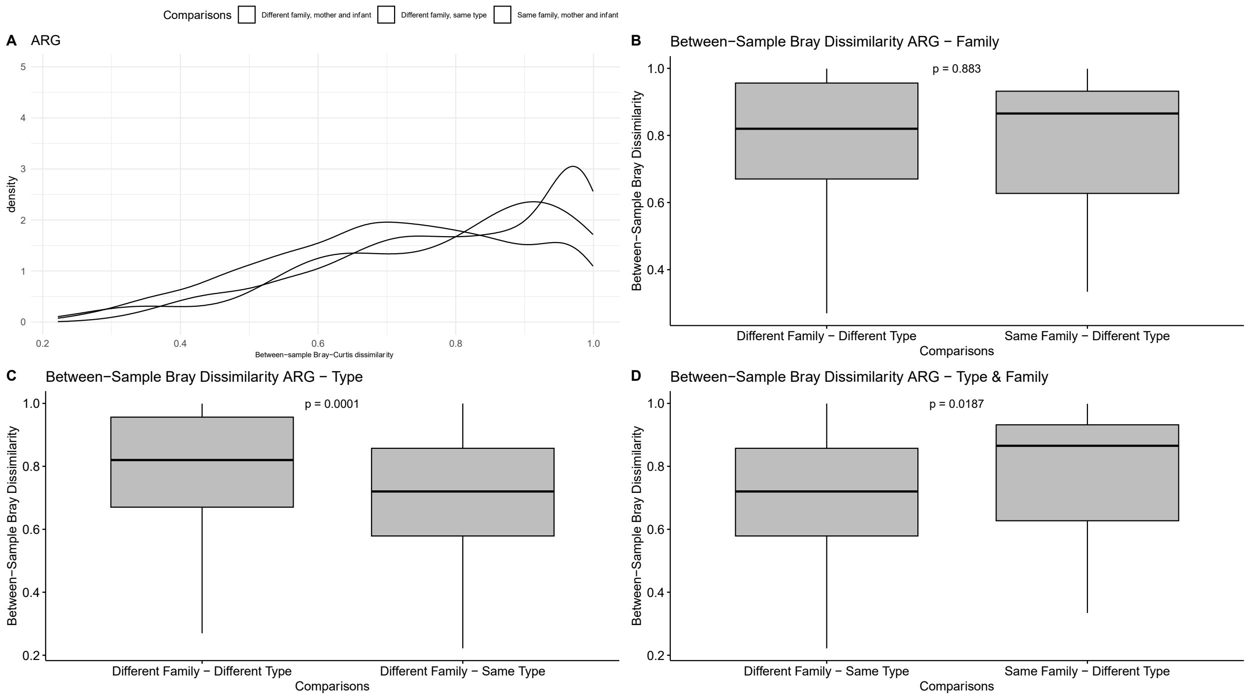

Figure S4. The ARG community structure of infant resistomes were more like that of other infants than their own mothers. Only matched pairs were included in this analysis. Kernel density plots based on Bray-Curtis (A) dissimilarity of ARG. The Bray-Curtis dissimilarity distances between groups were compared. The horizontal line in the center of the box and whisker plot represents the median, while the upper and lower limit of the box represent the interquartile range (IQR). The whiskers are determined by Q1/Q3 +/- 1.5 * IQR and anything falling outside of the whiskers represents an outlier. ‘Family’ (B): Bray-Curtis distances between related and unrelated dyads were similar though those of related dyads tended to be smaller than those of unrelated dyads. This indicates that the infancy ARG resistomes of related dyads are like those of unrelated dyads. ‘Type’ (C): Bray-Curtis distances between infant samples or between pregnancy samples were shorter than Sorensen distances between infant samples and pregnancy samples indicating that ARG resistome composition is more similar within sample types compared to between sample types. ‘Type & family’ (D): Bray-Curtis distances between related dyads were larger than Bray-Curtis distances between unrelated pregnancy or unrelated infancy samples indicating that the infant ARG resistome composition is more like other infant’s than that of their own mothers during pregnancy.

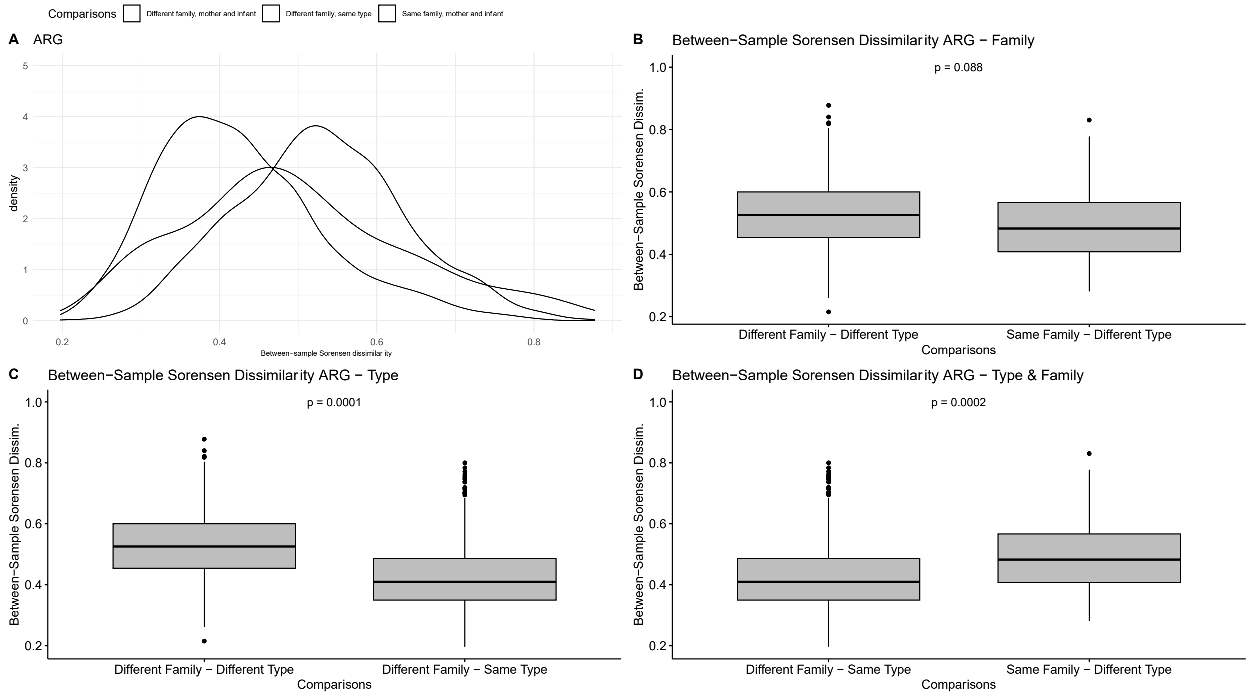

Figure S5. The ARG composition of resistomes of infant samples were more like that of other infants than that of their own mothers during pregnancy. Only matched pairs of pregnancy and infancy samples were included in this analysis. Kernel density plots based on Sorensen dissimilarity of ARG (A). The Sorensen dissimilarity distances between groups were compared. The horizontal line in the center of the box and whisker plot represents the median, while the upper and lower limit of the box represent the interquartile range (IQR). The whiskers are determined by Q1/Q3 +/- 1.5 * IQR and anything falling outside of the whiskers represents an outlier. 'Family' (B): Sorensen distances between related and unrelated dyads were similar. This indicates that the infancy ARG resistomes are related dyads are like those of unrelated dyads. 'Type' (C): Sorensen distances between infant samples or between pregnancy samples were shorter than Sorensen distances between infant samples and pregnancy samples indicating that ARG resistome composition is more similar within sample types compared to between sample types. 'Type & family' (D): Sorensen distances between related dyads were larger than Sorensen distances between unrelated pregnancy or unrelated infancy samples indicating that the infant ARG resistome composition is more like other infant's than that of their own mothers during pregnancy.

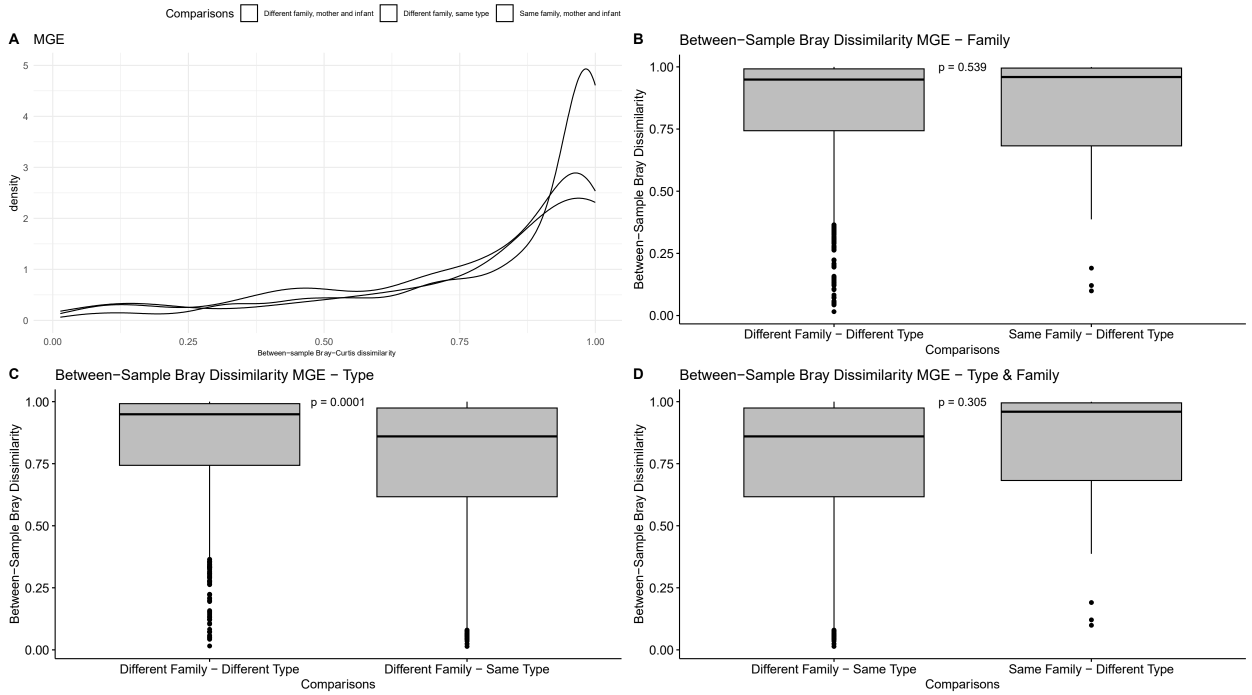

Figure S6. The MGE community structure of infant resistomes were more like that of other infants than their own mothers. Only matched pairs were included in this analysis. Kernel density plots based on Bray-Curtis (A) dissimilarity of MGE. The Bray-Curtis dissimilarity distances between groups were compared. The horizontal line in the center of the box and whisker plot represents the median, while the upper and lower limit of the box represent the interquartile range (IQR). The whiskers are determined by Q1/Q3 +/- 1.5 * IQR and anything falling outside of the whiskers represents an outlier. 'Family' (B): Bray-Curtis distances between related and unrelated dyads were similar. This indicates that the infancy MGE resistomes of related dyads are like those of unrelated dyads. 'Type' (C): Bray-Curtis distances between infant samples or between pregnancy samples were shorter than Sorensen distances between infant samples and pregnancy samples indicating that MGE resistome composition is more similar within sample types compared to between sample types. 'Type & family' (D): Bray-Curtis distances between related dyads were similar to Bray-Curtis distances between unrelated pregnancy or unrelated infancy samples, indicating there was no difference in community structure between comparisons of infants to their own mothers and infants to each other.

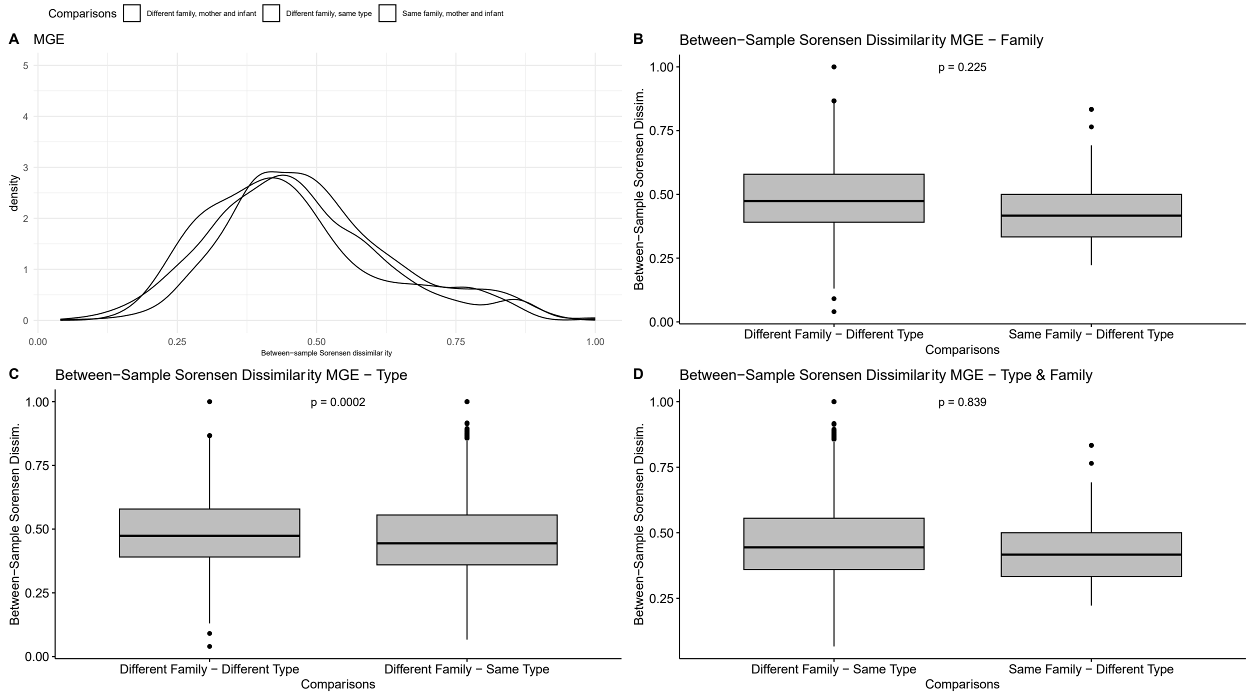

Figure S7. The MGE composition of resistomes of infant samples was similar to other infants. Only matched pairs of pregnancy and infancy samples were included in this analysis. Kernel density plots based on Sorensen dissimilarity of MGE (A). The Sorensen dissimilarity distances between groups were compared. The horizontal line in the center of the box and whisker plot represents the median, while the upper and lower limit of the box represent the interquartile range (IQR). The whiskers are determined by Q1/Q3 +/- 1.5 * IQR and anything falling outside of the whiskers represents an outlier. 'Family' (B): Sorensen distances between related and unrelated dyads were similar. This indicates that the infancy MGE resistomes are related dyads are like those of unrelated dyads. 'Type' (C): Sorensen distances between infant samples or between pregnancy samples were shorter than Sorensen distances between infant samples and pregnancy samples indicating that MGE resistome composition is more similar within sample types compared to between sample types. 'Type & family' (D): Sorensen distances between related dyads were similar to Sorensen distances between unrelated pregnancy or unrelated infancy samples, indicating there was no difference in community composition between comparisons of infants to their own mothers and infants to each other.

Table S5. Most prevalent ARG found in matched pregnant women and infant stool samples.

Present in dyads (n=66)	Gene Name	Function	Antibiotic Target
58(88%)	*acrF*	efflux	MDR
59(89%)	*tetM*	protection	Tetracycline
61(92%)	*tetW*	protection	Tetracycline
61(92%)	*mefA*	efflux	MLSB
63(95%)	*tnpA*	Transposase	MGE

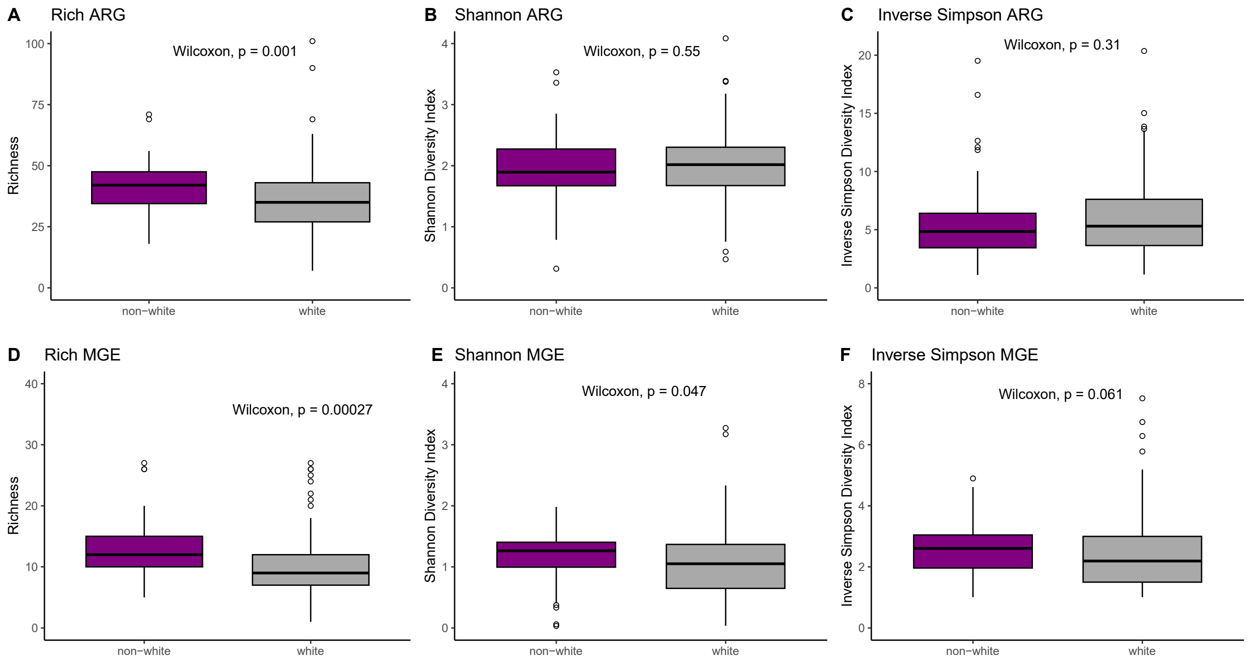

Figure S8. Box and whisker plot of Richness, Shannon, and Inverse Simpson for three-month-old infants with white (grey, n =153) and non-white mothers (purple, n = 51). The horizontal line in the center of the box and whisker plot represents the median, while the upper and lower limit of the box represent the interquartile range (IQR). The whiskers are determined by Q1/Q3

+/- 1.5 * IQR and anything falling outside of the whiskers represents an outlier. Infants with non-white mothers had a greater richness of ARG and MGE (A, B), as well as greater diversity of MGE (E).

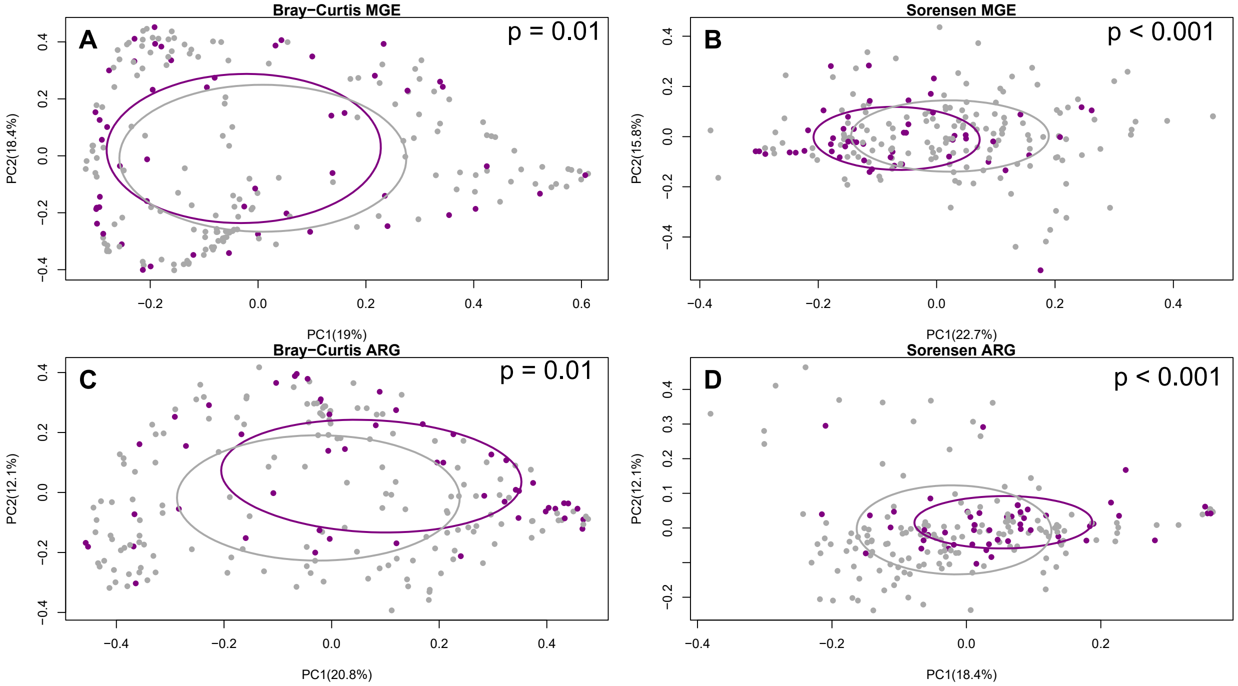

Figure S9. Infants with white mothers (grey, n=153) and non-white mothers (purple, n=51) had distinctive resistome communities. PCoA of Bray-Curtis dissimilarity for MGE (A) (PERMDISP: p < 0.0001) ARG (C) (PERMDISP: p = 0.94) and Sorensen dissimilarity for MGE (B) (PERMDISP: p < 0.0001) and ARG (D) (PERMDISP: p = 0.02). Grey dots represent infants with white mothers, while purple dots represent infants with non-white mothers. Each dot represents an individual infant, while ellipses are based on centroids of each group. Axis percentage accounts for percentage of variation explained by that given axis. The further apart each dot, the greater the dissimilarity.

Table S6. Univariate analysis assessing the association between infant and maternal characteristics and infant's resistome.

Variable of Interest	Alpha/Beta Diversity	Index	P-Value ARG	P-Value MGE
Race	Alpha	Richness	0.001*	<0.0001*

		Shannon	0.047*	0.55
		Inverse Simpson	0.31	0.06
	Beta	Bray-Curtis (PERMANOVA)	0.01*	0.01*
		Bray-Curtis (PERMDISP)	0.94	0.34
		Sorensen (PERMANOVA)	0.0008*	0.0004*
		Sorensen (PERMDISP)	0.021*	0.0001*
Method of delivery	Alpha	Richness	0.50	0.70
		Shannon	0.17	0.52
		Inverse Simpson	0.044*	0.62
	Beta	Bray-Curtis (PERMANOVA)	0.0001*	0.0034*
		Bray-Curtis (PERMDISP)	0.73	0.34
		Sorensen (PERMANOVA)	0.005*	0.69
		Sorensen (PERMDISP)	0.90	0.66
Infant Sex	Alpha	Richness	0.83	0.88
		Shannon	0.29	0.56
		Inverse Simpson	0.07	0.46
	Beta	Bray-Curtis (PERMANOVA)	0.007*	0.24
		Bray-Curtis (PERMDISP)	0.9	0.10
		Sorensen (PERMANOVA)	0.005*	0.91
		Sorensen (PERMDISP)	0.90	0.56
Smoking	Alpha	Richness	0.0008*	<0.0001*
		Shannon	0.31	0.02*
		Inverse Simpson	0.93	0.01*
	Beta	Bray-Curtis (PERMANOVA)	0.06	0.06

		Bray-Curtis (PERMDISP)	0.26	0.71
		Sorensen (PERMANOVA)	0.002*	<0.0001*
		Sorensen (PERMDISP)	<0.0001*	<0.0001*
Pregnant women's BMI Category	Alpha	Richness	0.05	0.21
		Shannon	0.40	0.93
		Inverse Simpson	0.17	0.78
	Beta	Bray-Curtis (PERMANOVA)	0.28	0.26
		Bray-Curtis (PERMDISP)	0.75	0.15
		Sorensen (PERMANOVA)	0.13	0.60
		Sorensen (PERMDISP)	0.22	0.10
Food Status (3 groups)	Alpha	Richness	<0.0001*	<0.0001*
		Shannon	0.38	0.66
		Inverse Simpson	0.66	0.56
	Beta	Bray-Curtis (PERMANOVA)	<0.0001*	<0.0001*
		Bray-Curtis (PERMDISP)	0.1532	<0.0001*
Antibiotics since birth	Alpha	Richness	0.63	0.49
		Shannon	0.76	0.61
		Inverse Simpson	0.97	0.45
	Beta	Bray-Curtis (PERMANOVA)	0.19	0.06
		Bray-Curtis (PERMDISP)	0.76	0.22
		Sorensen (PERMANOVA)	0.35	<0.0001*
		Sorensen (PERMDISP)	0.93	0.96

* p-value < 0.05

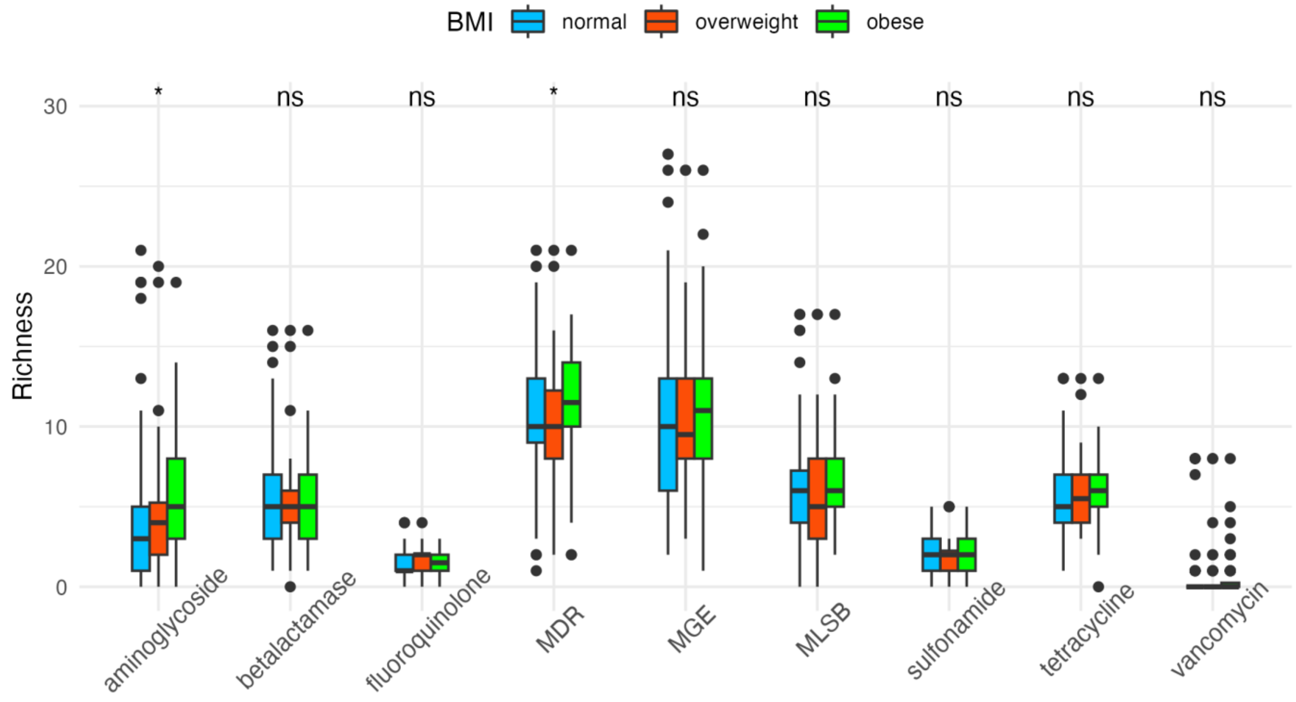

Figure S10. Box and whisker plot of Richness by ARG class by infant's mother's pre-pregnancy BMI category. Infants born to women with a pre-pregnancy with a BMI within a normal range (18.5-24.9) are in blue (n=84), infants born to women with a pre-pregnancy in the overweight range (25-29.9) are in red (n=48) and those infants born to women with obesity (>30) prior to becoming pregnant are in green (n =68). The horizontal line in the center of the box and whisker plot represents the median, while the upper and lower limit of the box represent the interquartile range (IQR). The whiskers are determined by Q1/Q3 +/- 1.5 * IQR and anything falling outside of the whiskers represents an outlier. Statistical significance is indicated by '*' for significance or NS for non-significant ($p > 0.05$). * $p < 0.05$, ** $p < 0.01$, *** $p <0.001$, **** $p <0.0001$. Post-hoc analysis revealed that obese women's infants had significantly higher presence of aminoglycoside ARG (Dunn Test: $p = 0.009$) than normal weight women and higher MDR (Dunn Test: $p = 0.0457$) than overweight women. Beta diversity did not differ by pregnant women's pre-pregnancy BMI.
