# Supplementary material for: Associations of diet, race, and other environmental factors with antimicrobial resistance genes in the gut bacterial communities of pregnant women and 3-month-old infants
Source: mSphere. 2025 Nov 24;10(12):e00445-25. doi: 10.1128/msphere.00445-25 (PMC12724134; doi:10.1128/msphere.00445-25)
Supplement: Supplemental Figures and Tables Part 2 — Figures S11 through S15 and Tables S7 through S9. [file msphere.00445-25-s0002.pdf]

Supplemental Figures and Tables Part 2.

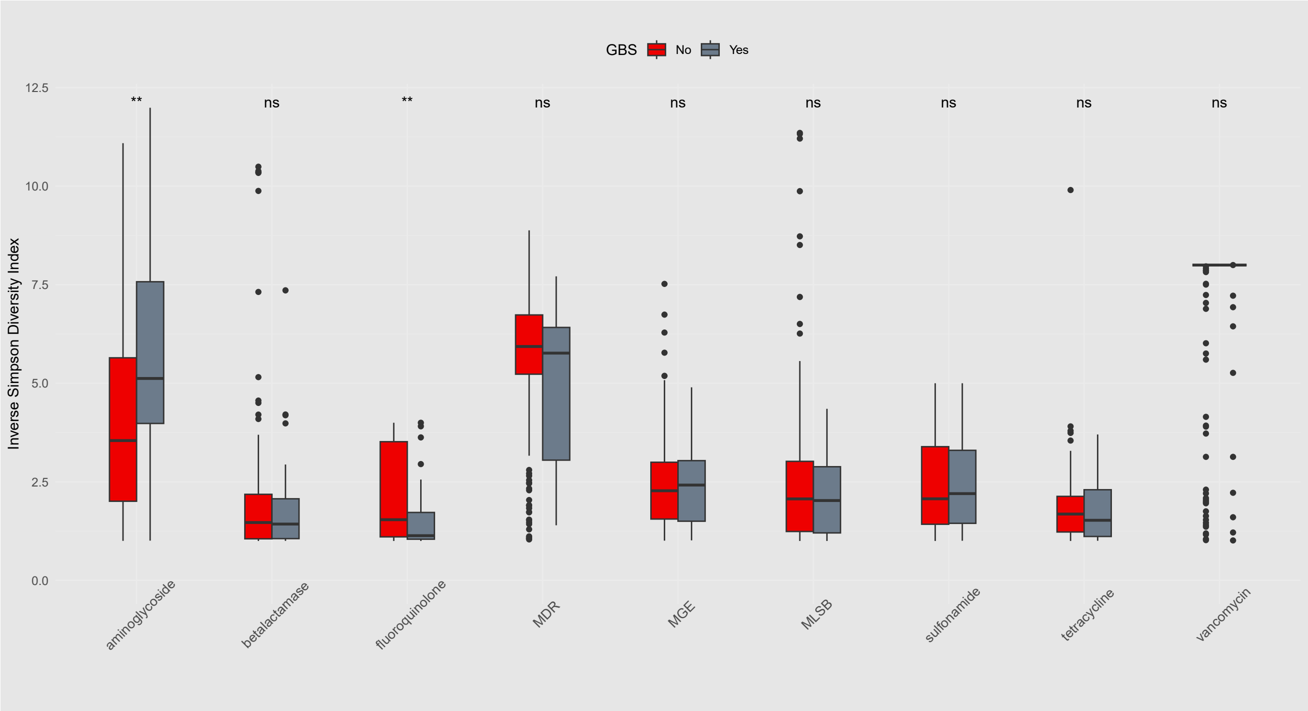

Figure S11. Box and whisker plot of Inverse Simpson for infants whose mother's had been treated for GBS during pregnancy (Yes, grey) versus those whose mother's had not (No, red) by ARG class. The horizontal line in the center of the box and whisker plot represents the median, while the upper and lower limit of the box represent the interquartile range (IQR). The whiskers are determined by Q1/Q3 +/- 1.5 * IQR and anything falling outside of the whiskers represents an outlier. Statistical significance is indicated by '*' for significance or NS for non-significant ($p > 0.05$). * $p < 0.05$, ** $p < 0.01$, *** $p < 0.001$, **** $p < 0.0001$. Infants whose mothers were given IAP for GBS ($n = 41$) had a higher diversity of aminoglycoside ARG. Infants whose mother's had not been given IAP ($n = 151$) for GBS had a higher diversity of fluroquinolone ARG.

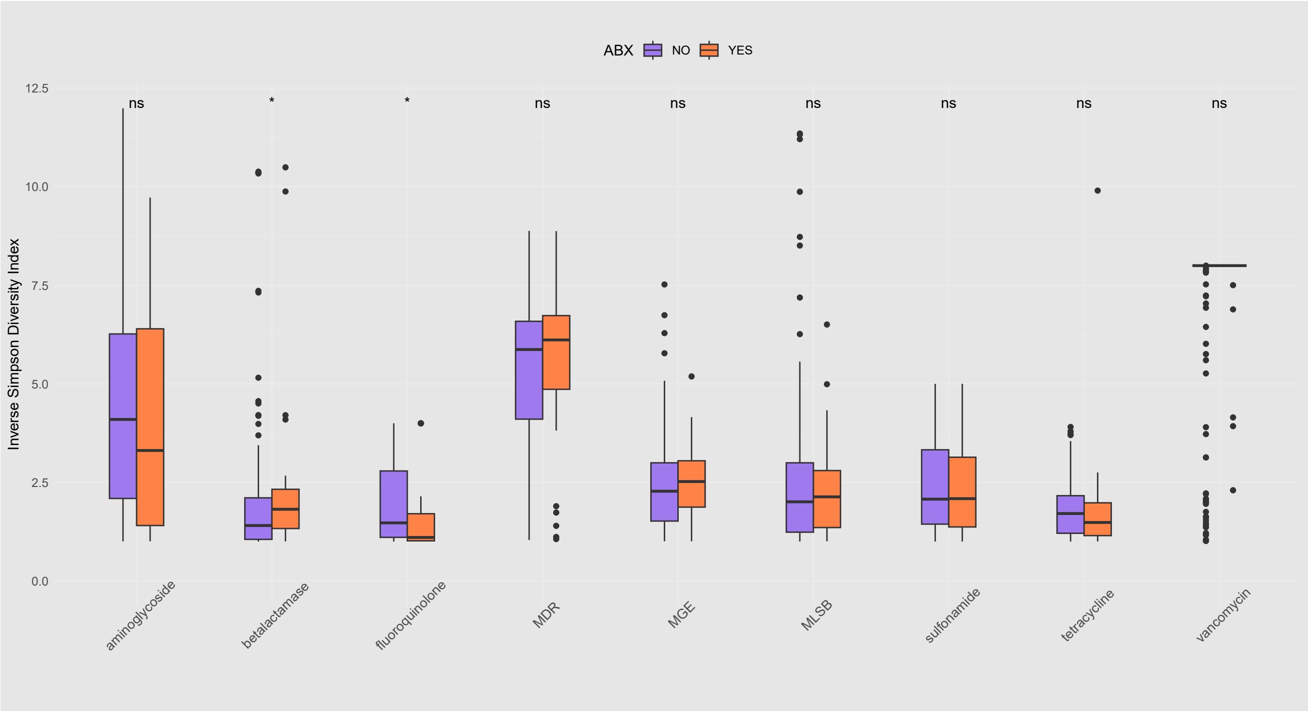

Figure S12. Box and whisker plot of Inverse Simpson for infants who had been given antibiotics since birth (Yes, orange) versus those who had not (No, purple). The horizontal line in the center of the box and whisker plot represents the median, while the upper and lower limit of the box represent the interquartile range (IQR). The whiskers are determined by Q1/Q3 +/- 1.5 * IQR and anything falling outside of the whiskers represents an outlier. Statistical significance is indicated by '*' for significance or NS for non-significant ($p > 0.05$). * $p < 0.05$, ** $p < 0.01$, *** $p < 0.001$, **** $p < 0.0001$. Infants who had antibiotics since birth (n = 28) had a greater diversity of beta-lactamase ARG, while infants who had not had antibiotics (n=163) since birth had a greater diversity of fluoroquinolone ARG.

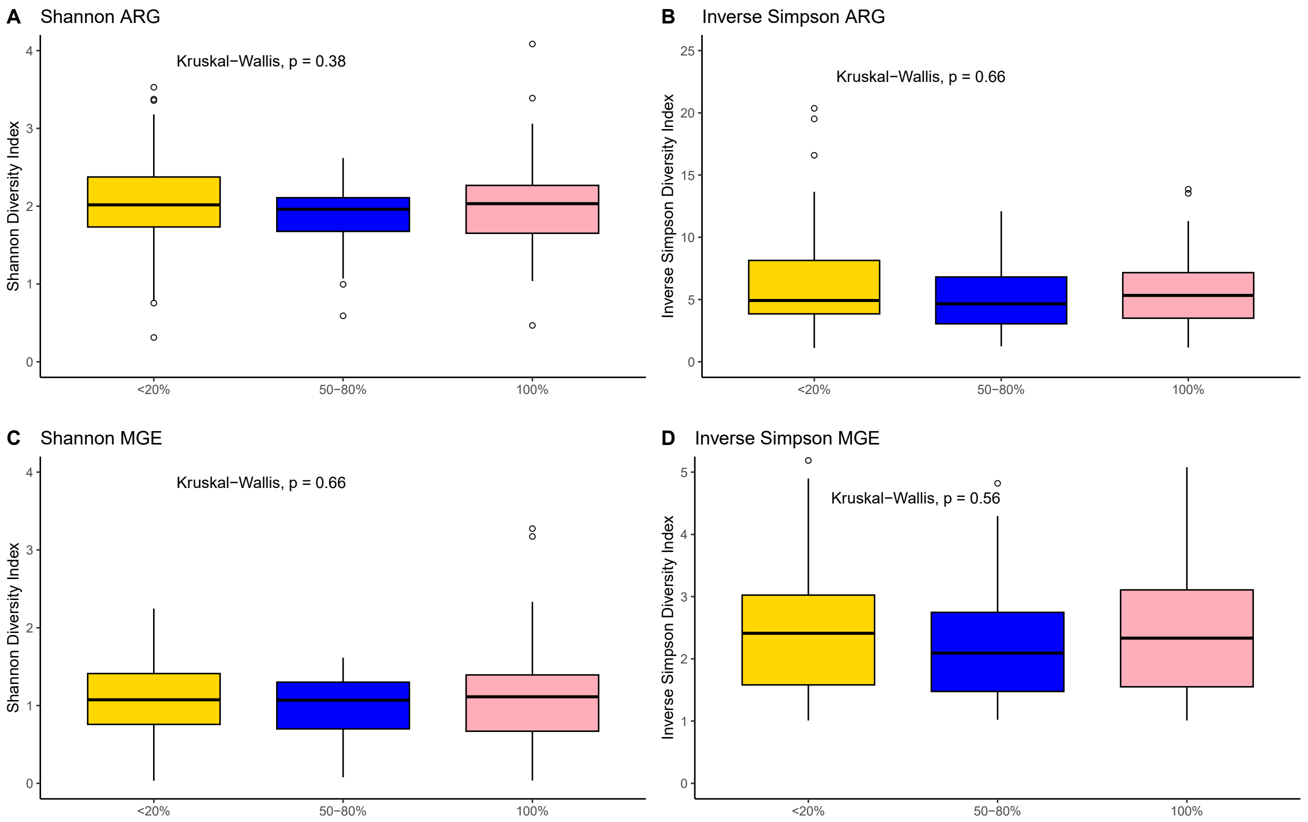

Figure S13. Box and whisker plot of Shannon and Inverse Simpson Indices of alpha diversity for ARG and MGE for infants grouped by percent of diet consisting of human breastmilk. Those consuming a diet of <20% breastmilk are in gold (n=20), those consuming a diet of 50-80% breastmilk are in blue (n=26), and those consuming a diet of 100% breastmilk are in pink (n=86). The horizontal line in the center of the box and whisker plot represents the median, while the upper and lower limit of the box represent the interquartile range (IQR). The whiskers are determined by Q1/Q3 +/- 1.5 * IQR and anything falling outside of the whiskers represents an outlier. There was no difference in overall diversity of ARG between any of the groups.

Table S7. Multivariate analysis assessing the association between Alpha Diversity of ARG and infant and maternal characteristics.

		**Model 1[a]**				**Model 2[b]**			
		Regression Coeff	Std Error	P-value	Adjusted R-squared	Regression Coeff	Std Error	P-value	Adjusted R-squared
**Chao**	Overall					1204.37	261.0	<0.0001*	0.01
	Mode of delivery	-314.9	176.3	0.08	0.01	-251.0	179.0	0.16	
	Antibiotic Exposure	-33.1	238.3	0.89	-0.01	8.30	238.0	0.97	
	Race	-353.3	192.5	0.07	0.01	-249.0	204.0	0.23	
	Feeding pattern	350.3	167.4	0.04*	0.02	235.0	180.0	0.19	
**Shannon**	Overall					2.10	0.12	<0.0001*	0.002
	Mode of delivery	-0.17	0.08	0.04*	0.02	-0.17	0.08	0.04 *	
	Antibiotic Exposure	0.03	0.11	0.82	-0.005	0.010	0.11	0.93	
	Race	0.01	0.09	0.9	-0.01	0.02	0.10	0.85	
	Feeding pattern	0.01	0.08	0.89	-0.01	-0.02	0.0	0.86	
**Inverse Simpson**	Overall					6.34	0.73	<0.0001*	0.02
	Mode of delivery	-1.37	0.48	0.005 *	0.04	-1.33	0.50	0.009*	
	Antibiotic Exposure	-0.57	3.80	0.88	-0.01	-0.25	0.66	0.71	
	Race	-7.54	3.06	0.01 *	0.03	0.30	0.57	0.60	

	Feeding pattern	8.94	2.63	0.0008*	0.05	0.33	0.50	0.50	

* p-value < 0.05

a = linear regression analysis of univariate characteristics using lm function in R

b = multivariate linear regression analysis of mode of delivery, antibiotic exposure, race, and infant feeding pattern

Table S8. Chao, Shannon, and Inverse Simpson scores for selected significant characteristics for infants.

	**Overall**	**Feeding pattern[a]**		**Mode of Delivery**		**Race**	
	**Mean, SD**		**Mean, SD**		**Mean, SD**		**Mean, SD**
**Chao**	984, 1162						
		FF	1139, 1193	Cesarean	-	White	-
		BF	789, 1098	Vaginal	-	Non-white	-
**Shannon**	1.99, 0.53						
		FF	-	Cesarean	2.10, 0.56	White	
		BF	-	Vaginal	1.93, 0.51	Non-white	
**Inverse Simpson**	5.84, 3.23						
		FF	6.06, 3.63	Cesarean	6.74, 3.74	White	5.85, 3.08
		BF	5.56, 2.65	Vaginal	5.38, 2.85	Non-white	5.80, 3.69

a = FF: non-exclusively breastfed, predominately formula fed infants; BF: exclusively breastfed infants

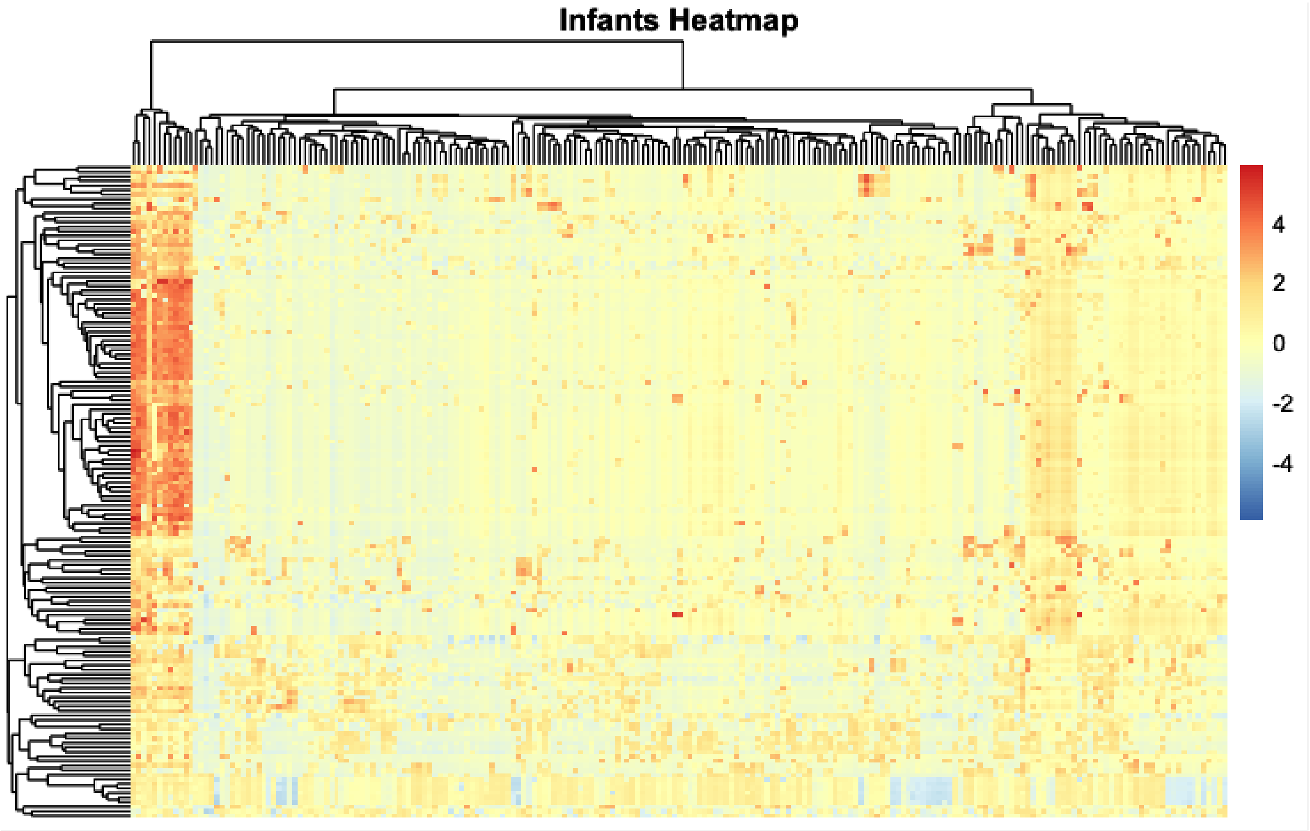

Figure S14. Hierarchal clustering heatmap of infant samples (top) and normalized gene abundance data (down) with complete linkage and Euclidean distances. Log normalized. Top dendrogram is based on infant samples (n=212) while left dendrogram shows inter-relatedness of genes based on normalized abundance.

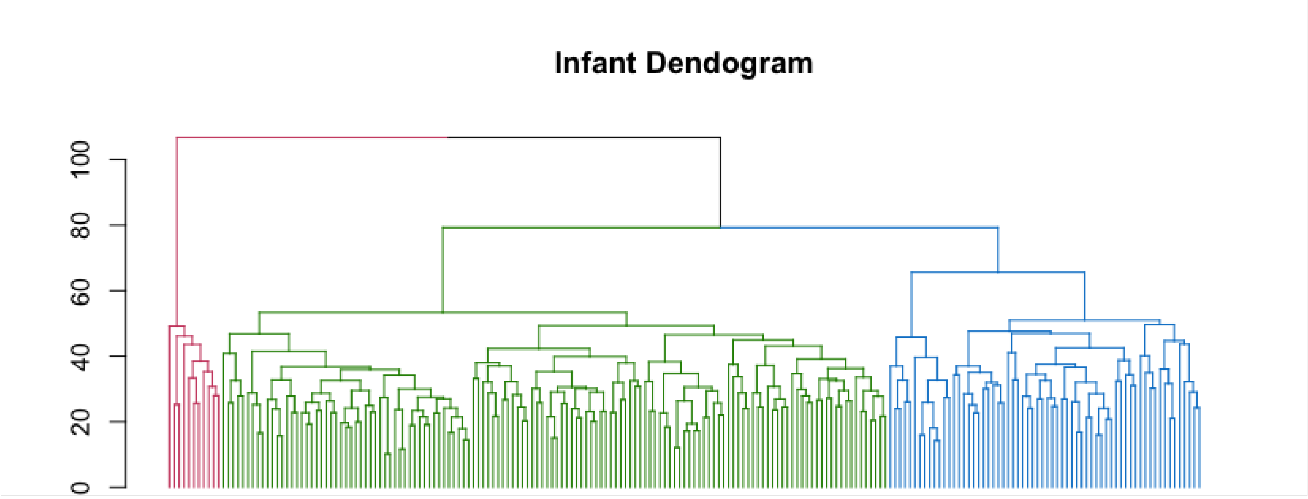

Figure S15. Dendrogram based on normalized abundance of ARG (including MGE) of infant samples (n=197) using Euclidean distances and complete linkages. Samples were clustered into three distinct clusters, as shown in red (cluster 3, n=10), green (cluster 1, n=140), and blue (cluster 2, n=47).

Table S9. Multinomial logistic regression results based on clustering patterns from hierarchal clustering analysis. Within this analysis, infants in cluster 2 (n= 47) and cluster 3 (n=10) were compared to infants in cluster 1 (n=140). Comparisons were made for mode of delivery of the infant, had infants had antibiotic exposure within the first three months of life, maternal race, and whether infant had been exclusively vs. non-exclusively breastfed (feeding pattern). Reference groups were cesarean born infants, infants having not had antibiotic exposure, non-white infants, and infants having been exclusively breastfed.

	Cluster 2				Cluster 3		
**Variable**	**Adjusted RR**	**95% Confidence Interval**	**P-value**		**Adjusted RR**	**95% Confidence Interval**	**P-value**
**Mode of delivery**	0.44	0.21, 0.92	0.03*		0.36	0.10, 1.39	0.14
**Antibiotic use**	1.11	0.41, 2.95	0.84		1.56	0.29, 8.31	0.60

**Race**	0.65	0.27, 1.56	0.33		0.56	0.12, 2.65	0.46
**Feeding Pattern**	0.27	0.12, 0.59	0.001*		0.41	0.10, 1.71	0.22

* p-value < 0.05
