# Supplementary material for: Associations of diet, race, and other environmental factors with antimicrobial resistance genes in the gut bacterial communities of pregnant women and 3-month-old infants
Source: mSphere. 2025 Nov 24;10(12):e00445-25. doi: 10.1128/msphere.00445-25 (PMC12724134; doi:10.1128/msphere.00445-25)
Supplement: Code — R code used to analyze data. [file msphere.00445-25-s0004.pdf]

R Code used to analyze data

Russell et al

```
###AMR 3mos Data/Analysis###############

#####Nov 28th 2022############

# Install (only needs to be run once)

# install.packages(c(

#   "haven",

#   "MASS",

#   "reshape2",

#   "vegan",

#   "lubridate",

#   "tidyr",

#   "car",

#   "dunn.test",

#   "ggplot2",

#   "openxlsx",

#   "dplyr",

#   "tidyverse"

# ))


# Load packages

library(haven)

library(MASS)

library(reshape2)

library(vegan)

library(lubridate)
```

```
library(tidyr)

library(car)

library(dunn.test)

library(ggplot2)

library(openxlsx)

library(dplyr)

library(tidyverse)



#remove.packages("tidyverse")

#install.packages("tidyverse")


#set working directory

setwd("/Users/madeleinerussell/Library/Mobile Documents/com~apple~CloudDocs/Desktop/Desktop - Madeleine's MacBook - 1/Comstock Lab/AbxResistance/ABXR_Ranalysis/AMR_Data")

getwd()

#can skip to line 350 unless you need to remake files


####

#####reading in tables####

metadata<-read.xlsx("20221031_AMR_MetaData.xlsx")

metadata$ID<-gsub('P8',"",metadata$ID)


setting<-read.xlsx("20221031_recruit_site_MR.xlsx")

names(setting)[names(setting) == 'SAMPLEID'] <- 'ID'

setting$ID<-gsub('P8',"",setting$ID)
```

```
setting$ID

Metadata_correctlocation<-inner_join(metadata, setting, by="ID")
Metadata_correctlocation$RECRUIT_SITE
unique(Metadata_correctlocation$RECRUIT_SITE)
#recruitsite<-as.table(Metadata_correctlocation$RECRUIT_SITE)
metadata<-Metadata_correctlocation

#write.xlsx(Metadata_correctlocation, "/Users/madeleinerussell/Desktop/Comstock Lab/AbxResistance/ABXR_Ranalysis/AMR_Data/2022OCT31_AMR_MetaData.xlsx", quote=FALSE, rowNames=FALSE)

#Creating the table into a more accessible variable
metadata$location<-gsub(".*Spectrum.*", "Spectrum",
                  gsub(".*Sinai Grace.*", "Sinai Grace",
                    gsub("*.Sinai-Grace.*", "Sinai Grace",
                      gsub(".*Munson.*", "Munson",
                        gsub(".*Briarwood.*", "UM",
                          gsub(".*UM.*", "UM",
                            gsub(".*Hutzel.*", "Hutzel", metadata$RECRUIT_SITE)))))))

table(metadata$location)
#write.xlsx(metadata, "/Users/madeleinerussell/Desktop/Comstock Lab/AbxResistance/ABXR_Ranalysis/AMR_Data/2023FEB03_AMR_MetaData.xlsx", quote=FALSE, rowNames=FALSE)
```

```
###At this point, the correct location should be applied

#####timepoint order #####


abundance<-read.xlsx("AMR_abundances.xlsx")

abundance.x<-abundance[,c("ID","Timepoint","pair","26","201","202","206","336","338","340","342","359","366","370","372","376","1522","1528","1546","1547","1548","1551","1552","1553","1556","1557","1558","1559","1565","1567","6","14","49","104","174","402","404","406","410","412","417","425","428","429","431","432","438","1503","1540","1541","1545","9","11","64","81","89","234","245","246","298","331","355","1300","1302","1303","1305","1504","1509","1536","1549","1572","1573","42","46","106","107","108","121","153","162","236","362","1108","1118","1123","1505","1512","1544","54","180","181","185","191","196","200","294","506","507","1507","1513","1539","91","137","138","209","227","229","283","285","801","804","806","809","812","815","817","819","1511","1519","133","177","208","280","363","211","213","214","215","306","309","316","318","328","1200","1201","1577","603","610","910","911","239","700","704","1500","1520","53")]

abundance.x<-abundance.x[order(abundance.x$pair),]

abundance<-abundance.x

#abundance$Study_ID<-smartchip.combined$added_study_ID


#create a variable at the end

abundance$class<-as.factor(ifelse(abundance$Timepoint== '1wk', 'Infant',
                   ifelse(abundance$Timepoint== '1mos', 'Infant',
                      ifelse(abundance$Timepoint== '3mos', 'Infant',
                         ifelse(abundance$Timepoint== '6mos', 'Infant',
                            ifelse(abundance$Timepoint== '12mos', 'Infant',
                               ifelse(abundance$Timepoint== '24mos', 'Infant', 'Mother')))))))


###presence

presence<-read.xlsx("AMR_presence.xlsx")
```

```
presence.x<-
presence[,c("Assay_IDnum","Timepoint","pair","26","201","202","206","336","338","340","342","
359","366","370","372","376","1522","1528","1546","1547","1548","1551","1552","1553","1556","1
557","1558","1559","1565","1567","6","14","49","104","174","402","404","406","410","412","417","4
25","428","429","431","432","438","1503","1540","1541","1545","9","11","64","81","89","234","245","
246","298","331","355","1300","1302","1303","1305","1504","1509","1536","1549","1572","1573","
42","46","106","107","108","121","153","162","236","362","1108","1118","1123","1505","1512","15
44","54","180","181","185","191","196","200","294","506","507","1507","1513","1539","91","137","1
38","209","227","229","283","285","801","804","806","809","812","815","817","819","1511","1519","
133","177","208","280","363","211","213","214","215","306","309","316","318","328","1200","1201"
,"1577","603","610","910","911","239","700","704","1500","1520","53")]

#order by monm and baby pair

presence.x<-presence.x[order(presence.x$pair),]

presence<-presence.x


presence$class<-as.factor(ifelse(presence$Timepoint== '1wk', 'Infant',
                         ifelse(presence$Timepoint== '1mos', 'Infant',
                           ifelse(presence$Timepoint== '3mos', 'Infant',
                             ifelse(presence$Timepoint== '6mos', 'Infant',
                               ifelse(presence$Timepoint== '12mos', 'Infant',
                                 ifelse(presence$Timepoint== '24mos', 'Infant', 'Mother')))))))



# write.xlsx(abundance, "/Users/madeleinerussell/Desktop/Comstock
Lab/AbxResistance/ABXR_Ranalysis/AMR_Data/AMR_abundances_location_class.xlsx",
quote=FALSE, rowNames=FALSE)

# write.xlsx(presence, "/Users/madeleinerussell/Desktop/Comstock
Lab/AbxResistance/ABXR_Ranalysis/AMR_Data/AMR_presence_location_class.xlsx",
quote=FALSE, rowNames=FALSE)
```

```
#####If you want to finish making them this is where####

#####need to fix ID list

abun3mos<-abundance[grep("3mos", abundance$Timepoint), ]
abun3mos$match<-abun3mos$ID


#creating new data frame
df1<-abun3mos %>%
  select(ID, pair, match)

#fixing messy IDs so they can match better
df1$match
df1$match<-gsub('.*8756 Ella.*',"35FE8756",df1$match)
df1$match<-gsub('.*8756Char.*',"25FE8756",df1$match)
df1$match<-gsub('.*8756 Char.*',"25FE8756",df1$match)
df1$match<-gsub('.*8493A.*',"25FE8493",df1$match)
df1$match<-gsub('.*8493 A.*',"25FE8493",df1$match)
df1$match<-gsub('8493Slias',"35FE8493",df1$match)
df1$match<-gsub('25FE8119Kalv',"35FE8119",df1$match)
df1$match<-gsub('25FE8119Kaid',"25FE8119",df1$match)
df1$match<-gsub('.*25FE8945.*',"25FE8945",df1$match)
df1$match<-gsub('.*25FE7124.*',"25FE7124",df1$match)
df1$match<-gsub('.*45FE8684*',"35FE8684",df1$match)
df1$match<-gsub('.*MM005_74B3M.*', "25FE8116", df1$match)
```

```
df1$match<-gsub('.*MM001_69B3M.*', "25FE8663", df1$match)

df1$match<-gsub('.*MM003_71B3M.*', "25FE8873", df1$match)

df1$match<-gsub('.*MM004_72B3M.*', "25FE8269", df1$match)

df1$match<-gsub('.*MM007_77B3M.*', "25FE8424", df1$match)

df1$match<-gsub('.*MM008_80B3M.*', "25FE8986", df1$match)

df1$match<-gsub('.*MM017_82B3M.*', "25FE8323", df1$match)

df1$match<-gsub('.*88B3mos.*', "25FE8614", df1$match)


df1$match<-gsub("Omni", "",
        gsub("omni", "",
          gsub("PP", "",
            gsub("pp", "", df1$match))))


df1$match


#remove gut kit number from #25FE8518


####you need to double check duplicates & then also do cross walk. yay!
head(df1$match)
un<-as.list(unique(df1$match))
#seeing which IDs are duplicated
df2<-abun3mos[duplicated(df1$match)|duplicated(df1$match, fromLast=TRUE),]
df3<-df2[order(df2$match),]


df1 <- df1 %>% distinct(match, .keep_all = TRUE)
#remove pair
```

```
df4<-df1[,-c(2)]

df4 <- df4 %>% distinct(match, .keep_all = TRUE)


abun3mos.list<-inner_join(df4, abun3mos, by="ID")

#only have to do this if older file, shouldn't have to now

abun3mos<-abun3mos.list[,-c(149:152)]

#watch 25FE8518, has same gut kit # as 25FE8712




##made this file 2023JAN31 because I needed the cross walk data

crossmeta<-read.xlsx("2023_3mos_Crosswalk_4_MAdi.xlsx")

names(abun3mos)[names(abun3mos) == 'match.x'] <- 'match'

abun3mos$match


crossmeta$match<-gsub("Omni", "",

             gsub("omni", "",

               gsub("PP", "",

                 gsub("pp", "", crossmeta$Specimen.ID))))


crossmeta2<-crossmeta %>% distinct(match, .keep_all = TRUE)

crossmeta2$match<-gsub('.*35FE8684.*',"35FE8684",crossmeta2$match)

crossmeta2$match<-gsub('.*25FE8684.*',"25FE8684",crossmeta2$match)

crossmeta2$match<-gsub('.*25FE8945A.*',"25FE8945",crossmeta2$match)


crossmeta2$match

abun3mos$match
```

```
abun3mos <- abun3mos %>% mutate(match = str_squish(match))
abun3mos2<-left_join(abun3mos, crossmeta2, by = 'match')
abun3mos3 <- abun3mos2 %>% distinct(match, .keep_all = TRUE)
df5<-abun3mos3[,c(1:2)]
#abun3mos3$match

#check list
#un<-as.list(unique(abun3mos2$match))

#match the 212 to presence data
names(presence)[names(presence) == 'Assay_IDnum'] <- 'ID'
pres3mos<-inner_join(presence, df5, by='ID')
pres3mos<-pres3mos %>% distinct(match, .keep_all = TRUE)



#match 99 moms
abunmoms<-abundance[grep("M", abundance$Timepoint), ]
abunmoms$match<-gsub("Omni", "",
        gsub("omni", "",
          gsub("PP", "",
            gsub("pp", "", abunmoms$ID))))
abunmoms <- abunmoms %>% distinct(pair, .keep_all = TRUE)
abun3mos2<-left_join(abunmoms, crossmeta2, by = 'pair')
```

```
presence.momsonly<-presence[grep("M",presence$Timepoint),]

df6<-abunmoms[,c(1,148)]
presmoms<-inner_join(presence, df6, by='ID')

pres.test<-rbind(pres3mos, presmoms)
pres.test <- pres.test %>% distinct(match, .keep_all = TRUE)

#just 3 months info
# write.xlsx(abun3mos3, "/Users/madeleinerussell/Desktop/Comstock Lab/AbxResistance/ABXR_Ranalysis/AMR_Data/2023APR17_abun3mosdata.xlsx", quote=FALSE, rowNames=FALSE)
# write.xlsx(pres3mos, "/Users/madeleinerussell/Desktop/Comstock Lab/AbxResistance/ABXR_Ranalysis/AMR_Data/2023APR17_pres3mosdata.xlsx", quote=FALSE, rowNames=FALSE)

###How to add in abundance data

#read in files --> depends on the samples of interest
abundance.match<-read.xlsx("2023APR17_abun3mosdata.xlsx")
head(abundance.match)
presence.match<-read.xlsx("2023APR17_pres3mosdata.xlsx")
head(abunmoms)

#check to make sure same column names so can rbind moms and babies
#move match to the end so same order
abundance.3mos<-abundance.match %>% relocate(match, .after = last_col())
```

```
#get rid of MM_kit_ID because same as pairs and removed it in the mom column

abun.3mos<-abundance.3mos[,-c(151)]

names(abun.3mos)[names(abun.3mos) == 'pair.x'] <- 'pair'


#abunmoms$class<-"Mother"


abun.moms<-abunmoms[,-c(148:150)]

head(abun.moms)

abun.3mos<-abundance.match[,-c(149:152)]

head(abun.3mos)

abun.test<-rbind(abun.3mos, abun.moms)


abun.test<-abun.test %>% relocate(match, .after = last_col())

pres.test<-pres.test %>% relocate(match, .after = last_col())



# write.xlsx(abun.test, "/Users/madeleinerussell/Desktop/Comstock Lab/AbxResistance/ABXR_Ranalysis/AMR_Data/2023APR18_abundance3mos_plusmoms_MR.xlsx", quote=FALSE, rowNames=FALSE)

# write.xlsx(pres.test, "/Users/madeleinerussell/Desktop/Comstock Lab/AbxResistance/ABXR_Ranalysis/AMR_Data/2023APR18_presence3mos_plusmoms_MR.xlsx", quote=FALSE, rowNames=FALSE)


####crossing data -- this small part is just for babies with matched pairs #####

cross<-read.xlsx("2023APR18_mom_IDlist_MR.xlsx")

#cross<-read.xlsx("test.xlsx")

cross$match
```

```
library(stringr)

library(dplyr)



abundance.match<-read.xlsx("2023APR17_abun3mosdata.xlsx")

abundance.3moscross<-abundance.match[,-c(149:152)]

abundance.3moscross$match

head(abundance.3moscross)


#this takes away trailing and leading white space because some of the IDs were weird

testdata <- abundance.3moscross %>% mutate(match = str_squish(match))

testdata$match


abun3matched<-inner_join(testdata, cross, by="match")

abun3matchedbabies<- abun3matched %>% distinct(match, .keep_all = TRUE)

write.xlsx(abun3matchedbabies, "/Users/madeleinerussell/Desktop/Comstock
Lab/AbxResistance/ABXR_Ranalysis/AMR_Data/2023APR18_AMR_3mos_matchedbbys.xls
x", quote=FALSE, rowNames=FALSE)

abun3matchedbabies2<-abun3matchedbabies[,-c(4, 149:151)]

names(abun3matchedbabies2)[names(abun3matchedbabies2) == 'pair.y'] <- 'pair'


#make mom file


df6<-abun3matchedbabies2[,c(148:149)]

df6$pair

abunmom.matched<-inner_join(abunmoms, df6, by="pair")
```

```
head(abun3matchedbabies2)

# MARCHID Specimen.ID MM_ID    match March_id

abunmom.matched2<-abunmom.matched[,-c(148:150)]


names(abun3matchedbabies2)[names(abun3matchedbabies2) == 'Timepoint.x'] <-
'Timepoint'


abun.test<-rbind(abunmom.matched2, abun3matchedbabies2)

write.xlsx(abun.test, "/Users/madeleinerussell/Desktop/Comstock
Lab/AbxResistance/ABXR_Ranalysis/AMR_Data/2023APR18_AMR_3mos_matchedmoms_b
bys.xlsx", quote=FALSE, rowNames=FALSE)


#fix presence

df6<-abun.test[,c(1,3,148:149)]

presence$class

pres.matched<-inner_join(presence, df6, by='ID')

write.xlsx(pres.matched, "/Users/madeleinerussell/Desktop/Comstock
Lab/AbxResistance/ABXR_Ranalysis/AMR_Data/2023APR18_pres_3mos_matchedmoms_b
bys.xlsx", quote=FALSE, rowNames=FALSE)



####crossing data-- with meta data, all infants#####

#moms

crossmeta<-read.xlsx("2023_3mos_Crosswalk_4_MAdi.xlsx")

names(crossmeta)[names(crossmeta) == 'MM_Kit_ID'] <- 'pair'

#abundancemoms<-binary[grep("M", binary$Timepoint), ]

abundancemoms<-abundance[grep("M", abundance$Timepoint), ]
```

```
abunmoms.cross<-left_join(abundancemoms, crossmeta, by="pair")


abunmoms.cross2 <- abunmoms.cross %>% distinct(pair, .keep_all = TRUE)

abunmoms<-abunmoms.cross2


#add in BC data


abundance.match<-read.xlsx("2023APR17_abun3mosdata.xlsx")

abundance.3moscross<-abundance.match[,-c(150:152)]

names(abundance.3moscross)[names(abundance.3moscross) == 'pair.x'] <- 'pair'

abundance.3mos<-abundance.3moscross %>% relocate(match, .after = last_col())
#moved match to the end

abundance.3moscross <- abundance.3mos %>% mutate(match = str_squish(match)) #get
rid of white space

abundance.3moscross$match


head(abundance.3moscross)

birthcert<-read.xlsx("2022JAN26_data4Madi_set2_bc_info.xlsx")

names(birthcert)[names(birthcert) == 'ID'] <- 'MARCHID'

birthcert_abun<-inner_join(abundance.3moscross, birthcert, by="MARCHID")

BC_3mos_abun <- birthcert_abun %>% distinct(match, .keep_all = TRUE)

#n = 205

write.xlsx(BC_3mos_abun, "/Users/madeleinerussell/Desktop/Comstock
Lab/AbxResistance/ABXR_Ranalysis/AMR_Data/2023APR21_AMR_3mosBCData_plusabun
.xlsx", quote=FALSE, rowNames=FALSE)


#birthcert for moms
```

```
birthcert_abun_moms<-inner_join(abunmoms, birthcert, by="MARCHID")

BC_3mos_abun_moms <- birthcert_abun_moms %>% distinct(pair, .keep_all = TRUE)

#n=48


#add in meta data


#n=97

metadata<-read.xlsx("2023FEB03_AMR_MetaData.xlsx")

names(metadata)[names(metadata) == 'ID'] <- 'MARCHID'

metadata3mos<-inner_join(abundance.3moscross, metadata, by="MARCHID")

#n = 97


metadata3mos_moms<-inner_join(BC_3mos_abun_moms, metadata, by="MARCHID")

#n= 22


un<-as.list(unique(BC_3mos_abun$match))


metadata$MARCHID

bcdata$MARCHID

all.data<-inner_join(metadata, bcdata, by="MARCHID")

all.data <- all.data %>% distinct(MARCHID, .keep_all = TRUE)



write.xlsx(all.data, "/Users/madeleinerussell/Desktop/Comstock
Lab/AbxResistance/ABXR_Ranalysis/AMR_Data/2023APR17_AMR_3mosMetaData.xlsx",
quote=FALSE, rowNames=FALSE)
```

```
write.xlsx(metadata, "/Users/madeleinerussell/Desktop/Comstock Lab/AbxResistance/ABXR_Ranalysis/AMR_Data/2023APR17_AMR_3mosMetaData_only.xlsx", quote=FALSE, rowNames=FALSE)



#metadata.checkfordups<-rbind(metadata3mos, metadatamoms)


table(metadata3mos$location)



####since we have the same ID at multiple time points, easiest to split this up
##I did this above, basically ignore this
# abundance3mos<-abundance[grep("3mos|M",abundance$Timepoint),]
# abundancemoms<-abundance[grep("M",abundance$Timepoint),]
#
# data.moms<-abundancemoms[,c(1:3)]
# write.xlsx(data.moms, "/Users/madeleinerussell/Desktop/Comstock Lab/AbxResistance/ABXR_Ranalysis/AMR_Data/2023APR04_mom_IDlist_MR.xlsx", quote=FALSE, rowNames=FALSE)
#
#
# table(abundance$Timepoint)




######Checking for pp vs. omni-- not necessary if not doing pp vs. omni analysis########
#####Duplicates
```

```
# abundance3mos<-read.xlsx("2023APR18_abundance3mos_plusmoms_MR.xlsx")

# presence3mos<-read.xlsx("2023APR18_presence3mos_plusmoms_MR.xlsx")

#

# presence<-presence3mos

# abundance<-abundance3mos

# abundance3mos.only<-abundance[grep("3mos",abundance$Timepoint),]


head(df1$match)

un<-as.list(unique(df1$match))

#seeing which IDs are duplicated

df2<-abun3mos[duplicated(df1$match)|duplicated(df1$match, fromLast=TRUE),]
#selecting for only duplicated IDs

df3<-df2[order(df2$match),]


head(df3)

abundance3mos.only<-df3


# #create a column for tube type

# df1$tubetype<-df1$ID

# df1$tubetype

# df1$tubetype<-gsub(".*omni.*", "omni",

#                       gsub(".*Omni.*", "omni", df1$tubetype))

# df1$tubetype<-as.factor(ifelse(df1$tubetype== 'omni', 'omni', 'pp'))
```

```
# head(df1$tubetype)

df1a<-df1[grep("pp",df1$tubetype),] #150

df1b<-df1[grep("omni",df1$tubetype),] #101


#creating a column for duplicate


#create a column for tube type

abundance3mos.only$tubetype<-abundance3mos.only$ID

abundance3mos.only$tubetype<-gsub(".*omni.*", "omni",

                    gsub(".*Omni.*", "omni", abundance3mos.only$tubetype))

abundance3mos.only$tubetype<-as.factor(ifelse(abundance3mos.only$tubetype==
'omni', 'omni', 'pp'))

head(abundance3mos.only$tubetype)


# #creating a column for duplicates

abundance3mos.only$dup<-gsub("Omni", "",

                 gsub("omni", "",

                    gsub("PP", "",

                       gsub("pp", "", abundance3mos.only$ID))))


abundance3mos.only$tubetype

#abundance3mos.only<-
abundance3mos.only[grep("pp",abundance3mos.only$tubetype),]


###look for duplicates & testing for them

head(abundance3mos.only$dup)

un<-as.list(unique(abundance3mos.only$dup)) #only 16 with both pp and omni
```

```
df2a<-
abundance3mos.only[duplicated(abundance3mos.only$dup)|duplicated(abundance3mos
.only$dup, fromLast=TRUE),]

df3a<-df2a[order(df2a$ID),]

df4 <- df3a[-c(9, 10), ] # these individuals were both OG, only one was used


#performing t-test

abundance2<-df4[,4:146]

abundance2[is.na(abundance2)]<- 0


#alpha

OTU<-abundance2[,1:143]

#make numeric

OTU<- mutate_all(OTU[1:143], function(x) as.numeric(as.character(x)))


#alpha diversity

OTU<-abundance2[1:143]

#make numeric

OTU<- mutate_all(OTU, function(x) as.numeric(as.character(x)))


#Alpha table

Alpha<-function(OTU,Names="ID",Groups="tubetype"){

  Shannon<-diversity(OTU,index="shannon")

  Invsimpson<-diversity(OTU,index="invsimpson")

  OTU.Subsample.Alpha<-data.frame(Names,Groups,Shannon,Invsimpson)

  return(OTU.Subsample.Alpha)

}
```

```
Data.Alpha<-Alpha(abundance2[,c(1:143)]) #MGE

Data.Alpha

shapiro.test(Data.Alpha$Shannon)

#p-value = 0.5253

shapiro.test(Data.Alpha$Invsimpson)

#p-value = 0.092

shan<-Data.Alpha$Shannon

head(shan)

length(shan)


levels(df4$tubetype)

a<-df4$tubetype

length(a) #14


#levels(df3$tubetype)

boxplot(shan~a,main="Shannon Index of Tube Type - Infants",ylab="Shannon Index")

t.test(shan~a, paired = TRUE, alternative = "two.sided")


# Paired t-test

#

# data:  shan by a

# t = 0.69944, df = 6, p-value = 0.5105

# alternative hypothesis: true mean difference is not equal to 0

# 95 percent confidence interval:

#   -0.3375981  0.6078509

# sample estimates:
```

```
#   mean difference
# 0.1351264


inv<-Data.Alpha$Invsimpson
boxplot(inv~a,main="Inverse Simpson Index of Tube Type - Infants",ylab="Inverse Simpson Index")
t.test(inv~a, paired = TRUE, alternative = "two.sided")
# > inv<-Data.Alpha$Invsimpson
# > t.test(inv~a, paired = TRUE, alternative = "two.sided")
#
# Paired t-test
#
# data:  inv by a
# t = 0.23156, df = 6, p-value = 0.8246
# alternative hypothesis: true mean difference is not equal to 0
# 95 percent confidence interval:
#   -1.394584  1.686120
# sample estimates:
#   mean difference
# 0.1457679



t.test(df4$`26`~a, paired = TRUE, alternative = "two.sided")
boxplot(df4$`26`~a,main="Gene Test -- Tube Type Infants",ylab="Normalized Abundance")


#testing a specific gene
```

```
# Paired t-test
#
# data:  df4$`26` by a
# t = -0.79901, df = 6, p-value = 0.4547
# alternative hypothesis: true mean difference is not equal to 0
# 95 percent confidence interval:
#   -0.012486838  0.006339365
# sample estimates:
#   mean difference
# -0.003073736


#make matching presence to detect richness


#match the 212 to presence data
abundance2<-df4[,4:146]
df6<-df4[,c(1,148:150)]


names(presence)[names(presence) == 'Assay_IDnum'] <- 'ID'


pres.tubetype<-inner_join(presence, df6, by='ID')
presence2<- pres.tubetype[,4:146]
presence2


pres.tubetype$RichARG<-rowSums(presence2[,1:143])
RichARG<-pres.tubetype$RichARG
```

```
t.test(RichARG~a, paired = TRUE, alternative = "two.sided")

# Paired t-test
t.test(RichARG~a, paired = TRUE, alternative = "two.sided")
boxplot(RichARG~a,main="Richness of Tube Type -- Infants",ylab="Richness")

# Paired t-test
#
# data:  RichARG by a
# t = -1.0486, df = 6, p-value = 0.3348
# alternative hypothesis: true mean difference is not equal to 0
# 95 percent confidence interval:
#   -23.81086   9.52515
# sample estimates:
#   mean difference
# -7.142857

#####checking omni vs pp in mom###

#create a column for tube type
abundancemoms<-abundance[grep("M",abundance$Timepoint),]

abundancemoms$tubetype<-abundancemoms$ID
```

```
abundancemoms$tubetype<-gsub(".*omni.*", "omni",
                gsub(".*Omni.*", "omni",
                  gsub(".*OG.*", "omni", abundancemoms$tubetype)))
head(abundancemoms$tubetype)
abundancemoms$tubetype<-gsub(".*M.*", "pp", abundancemoms$tubetype)
head(abundancemoms$tubetype)

#creating a column for duplicates
abundancemoms$dup<-gsub("Omni", "",
              gsub("omni", "",
                gsub("OG","",
                  gsub("PP", "",
                    gsub("pp", "", abundancemoms$ID)))))
abundancemoms$dup<-gsub('.*-',"",abundancemoms$dup)
abundancemoms$dup<-gsub('_',"",abundancemoms$dup)
abundancemoms$dup<-gsub('MM014|MM008|MM017',"",abundancemoms$dup)
abundancemoms$dup<-gsub('M',"",abundancemoms$dup)
head(abundancemoms$dup)
abundancemoms$dup<-as.numeric(abundancemoms$dup)

abundancemoms2<-abundancemoms[grep("pp",abundancemoms$tubetype),] #85
abundancemoms2<-abundancemoms[grep("omni",abundancemoms$tubetype),] #50


#look for duplicates
un<-as.list(unique(abundancemoms$dup))
```

```
df2b<-
abundancemoms[duplicated(abundancemoms$dup)|duplicated(abundancemoms$dup,
fromLast=TRUE),]

df3b<-df2b[order(df2b$dup),]

df4b<-df3b[-grep("2012-23_M|2021-23_M|104|104M",df3b$ID),] # both were PP


abundance2<-df4b[,4:146]

abundance2[is.na(abundance2)]<- 0


#alpha of MGERA

OTU<-abundance2[1:143]

#make numeric

OTU<- mutate_all(OTU[1:143], function(x) as.numeric(as.character(x)))


#OTU<-presence[,-c(1:3)]


#Alpha table

Alpha<-function(OTU,Names="ID",Groups="tubetype"){

  Shannon<-diversity(OTU,index="shannon")

  Invsimpson<-diversity(OTU,index="invsimpson")

  OTU.Subsample.Alpha<-data.frame(Names,Groups,Shannon,Invsimpson)

  return(OTU.Subsample.Alpha)

}

Data.Alpha<-Alpha(OTU) #MGE

Data.Alpha

shapiro.test(Data.Alpha$Shannon)

#p-value = 0.0002059
```

```
shapiro.test(Data.Alpha$Invsimpson)

#p-value = 4.504e-10


shan<-Data.Alpha$Shannon

head(shan)

length(shan)


levels(df4b$tubetype)

a<-df4b$tubetype

length(a) #14


#levels(df3$tubetype)

boxplot(shan~a,main="Shannon Index of Tube Type Pregnant Women",ylab="Shannon Index")

wilcox.test(shan ~ a, data = df4b, paired = TRUE)

#0.06692



# Wilcoxon signed rank exact test

#

# data:  shan by a

# V = 190, p-value = 0.06692

# alternative hypothesis: true location shift is not equal to 0



inv<-Data.Alpha$Invsimpson
```

```
wilcox.test(inv ~ a, data = df4b, paired = TRUE)

# Paired t-test
# Wilcoxon signed rank exact test
#
# data:  inv by a
# V = 226, p-value = 0.228
# alternative hypothesis: true location shift is not equal to 0



#match the 212 to presence data
abundance2<-df4b[,4:146]
df6<-df4b[,c(1,148:149)]

names(presence)[names(presence) == 'Assay_IDnum'] <- 'ID'

pres.tubetype<-inner_join(presence, df6, by='ID')
presence2<- pres.tubetype[,4:146]
presence2

pres.tubetype$RichARG<-rowSums(presence2[,1:143])
RichARG<-pres.tubetype$RichARG
shapiro.test(pres.tubetype$RichARG)
t.test(RichARG~a, paired = TRUE, alternative = "two.sided")

# Paired t-test
```

```
#
# data:  RichARG by a
# t = 1.2866, df = 33, p-value = 0.2072
# alternative hypothesis: true mean difference is not equal to 0
# 95 percent confidence interval:
#   -2.633226 11.692050
# sample estimates:
#   mean difference
# 4.529412


moms.nodup <- abundancemoms %>% distinct(dup, .keep_all = TRUE)
#df4 <- moms.nodup[, -c(159, 160)]


#####Removing Dupes####


abundance3mos.only$ID
abundance3mos.only$class
table(abundance3mos.only$class)


###removing duplicates from abundance


abundance3mos.nodup <- abundance3mos.only %>% distinct(dup, .keep_all = TRUE)
abundancemoms$dup<-as.numeric(abundancemoms$dup)
#abundance3mos.nodup2 <- abundance3mos.nodup %>% distinct(pair, .keep_all = TRUE)
```

```
abundance3mos.nodup2<-abundance3mos.nodup[-
grep("25FE8518|MM001_69B3M|MM003_71B3M|MM004_72B3M|MM007_77B3M|35FE805
6",abundance3mos.nodup$ID),]


write.xlsx(abundance3mos.nodup2, "/Users/madeleinerussell/Desktop/Comstock
Lab/AbxResistance/ABXR_Ranalysis/AMR_Data/2023JAN26_abundance3mos_nodup_MR.
xlsx", quote=FALSE, rowNames=FALSE)

write.xlsx(abundance.nodups.only, "/Users/madeleinerussell/Desktop/Comstock
Lab/AbxResistance/ABXR_Ranalysis/AMR_Data/2022DEC05_abundance3mos_plusmoms
_MR.xlsx", quote=FALSE, rowNames=FALSE)

#####presence
presence.3mosonly<-presence[grep("3mos",presence$Timepoint),]
presence.3mosonly$ID<-presence.3mosonly$Assay_IDnum
presence.3mosonly$tubetype<-presence.3mosonly$ID
presence.3mosonly$tubetype<-gsub(".*omni.*", "omni",
                         gsub(".*Omni.*", "omni", presence.3mosonly$ID))

presence.3mosonly$dup<-gsub("Omni", "",
                      gsub("omni", "",
                         gsub("PP", "",
                            gsub("pp", "", presence.3mosonly$ID))))

#checking to make sure lengths of dups is the same
head(presence.3mosonly$dup)
un<-as.list(unique(presence.3mosonly$dup))
```

```
df2<-
abundance3mos.only[duplicated(presence.3mosonly$dup)|duplicated(presence.3mosonl
y$dup, fromLast=TRUE),]

df3<-df2[order(df2$ID),]

#df4 <- df3[-c(9, 10), ]


presence.3mos.nodup <- presence.3mosonly %>% distinct(dup, .keep_all = TRUE)

presence.3mos.nodup2<-presence.3mos.nodup[-
grep("25FE8518|MM001_69B3M|MM003_71B3M|MM004_72B3M|MM007_77B3M|35FE805
6",presence.3mos.nodup$ID),]


write.xlsx(presence.3mos.nodup2, "/Users/madeleinerussell/Desktop/Comstock
Lab/AbxResistance/ABXR_Ranalysis/AMR_Data/2023JAN26_presence3mos_nodup_MR.xls
x", quote=FALSE, rowNames=FALSE)


#presence moms

presence.momsonly<-presence[grep("M",presence$Timepoint),]

presence.momsonly$ID<-presence.momsonly$Assay_IDnum

presence.momsonly$tubetype<-presence.momsonly$ID


#sep by tube type

presence.momsonly$tubetype<-gsub(".*omni.*", "omni",

                    gsub(".*Omni.*", "omni", presence.momsonly$ID))


presence.momsonly$dup<-gsub("Omni", "",

                 gsub("omni", "",

                    gsub("OG","",

                       gsub("PP", "",
```

```
                    gsub("pp", "", presence.momsonly$ID)))))
presence.momsonly$dup<-gsub('.*-',"",presence.momsonly$dup)
presence.momsonly$dup<-gsub('_',"",presence.momsonly$dup)
presence.momsonly$dup<-gsub('M',"",presence.momsonly$dup)
head(presence.momsonly$dup)
presence.momsonly$dup<-as.numeric(presence.momsonly$dup)

#put into a form that you can analyze duplicates

head(presence.momsonly$dup)
un<-as.list(unique(presence.momsonly$dup))
df2<-
presence.momsonly[duplicated(presence.momsonly$dup)|duplicated(presence.momsonl
y$dup, fromLast=TRUE),]
df3<-df2[order(df2$ID),]

presence.momsonly <- presence.momsonly %>% distinct(dup, .keep_all = TRUE)

presence.nodups.only<-rbind(presence.3mos.nodup2, presence.momsonly)

table(presence.nodups.only$Timepoint)
##3mos= 243
##M= 99
```

```
write.xlsx(presence.nodups.only, "/Users/madeleinerussell/Desktop/Comstock Lab/AbxResistance/ABXR_Ranalysis/AMR_Data/2022DEC05_presence3mos_plusmoms_MR.xlsx", quote=FALSE, rowNames=FALSE)


#####Abundance/Presence Data#####can start here########

#####Add in Abundances

#i had to make a "match column" for my original IDs because they were messy -- DOUBLE CHECK YOUR OWN CODE


abundance3mos<-read.xlsx("2023APR18_abundance3mos_plusmoms_MR.xlsx")

presence3mos<-read.xlsx("2023APR18_presence3mos_plusmoms_MR.xlsx")


presence<-presence3mos

abundance<-abundance3mos

#make it so just abundance data, not IDs etc

abundance2<-abundance[,4:146]

head(abundance2)


#set NAs to 0

abundance2[is.na(abundance2)]<- 0


abundance$SumMGE<-rowSums(abundance2[,1:27])

abundance$Sumamino<-rowSums(abundance2[,28:48])

abundance$SumMDR<-rowSums(abundance2[,49:69])

abundance$Sumbeta<-rowSums(abundance2[,70:85])

abundance$Sumtetra<-rowSums(abundance2[,86:98])
```

```r
abundance$SumMLSB<-rowSums(abundance2[,99:116])

abundance$Sumsulfo<-rowSums(abundance2[,117:121])

abundance$Sumvanco<-rowSums(abundance2[,122:129])

abundance$Sumfluoro<-rowSums(abundance2[,130:133])

abundance$Sumother<-rowSums(abundance2[,134:143])

abundance$SumARG<-rowSums(abundance2[,28:143])



# Install (only needs to run once)

# install.packages(c(

#   "gridExtra",

#   "vegan",

#   "Hmisc",

#   "plyr",

#   "plotrix",

#   "tidyverse",

#   "ggpubr"

# ))


# Load libraries

library(gridExtra)

library(vegan)

library(Hmisc)

library(plyr)

library(plotrix)

library(tidyverse)
```

```
library(ggpubr)

#performing wilcox tests
wilcox.test(abundance$Sumamino~abundance$Timepoint, data=abundance)
#p-value = 3.927e-11 **
summary(abundance$Sumamino)
wilcox.test(abundance$SumMDR~abundance$Timepoint, data=abundance)
#p-value < 2.2e-16
summary(abundance$SumMDR)
wilcox.test(abundance$Sumbeta~abundance$Timepoint, data=abundance)
#p-value =0.4964
wilcox.test(abundance$Sumtetra~abundance$Timepoint, data=abundance)
#p-value = 1.088e-05
wilcox.test(abundance$SumMLSB~abundance$Timepoint, data=abundance)
#p-value = 0.005909
wilcox.test(abundance$Sumsulfo~abundance$Timepoint, data=abundance)
#p-value = 8.532e-07
wilcox.test(abundance$Sumvanco~abundance$Timepoint, data=abundance)
#p-value = 0.0004223
wilcox.test(abundance$Sumfluoro~abundance$Timepoint, data=abundance)
#p-value = 3.464e-06
wilcox.test(abundance$Sumother~abundance$Timepoint, data=abundance)
#p-value = 5.293e-10
wilcox.test(abundance$SumMGE~abundance$Timepoint, data=abundance)
#p-value = 0.001178
```

```
#re-name to infancy and pregnancy

abundance$class2<-as.factor(ifelse(abundance$class== 'Mother','Pregnancy','Infancy'))

abundance$class


abundance$class2




#boxplots

abundance3mos<-abundance

# abundance3mos$class2<-abundance3mos$class

wilcox.test(abundance3mos$SumMGE~abundance3mos$class2, data=abundance3mos)

#Result: p value = 0.001178

a<- ggboxplot(data=abundance3mos, x="class2", y="SumMGE", color="black", fill="class2",
palette="d3", ylab="Sum of relative abundance", xlab="", outlier.colour = "black",
outlier.shape = 1, main="MGE 3mos")+ theme_classic() + guides(fill= "none") +
coord_cartesian(ylim = c(0, 4))+ stat_compare_means(label.x=1.6, label.y=3)

a


boxplot(abundance3mos$SumMGE~abundance3mos$class2, main="Sum of
abundances",ylab="Relative Abundance")


dev.off()


wilcox.test(abundance3mos$SumARG~abundance3mos$class2, data=abundance3mos)

#p-value = 0.5528

#arg
```

```
b<- ggboxplot(data=abundance3mos, x="class2", y="SumARG", color="black", fill="class2", palette="d3", ylab="Sum of relative abundance", xlab="", outlier.colour = "black", outlier.shape = 1, main="ARG 3mos")+ theme_classic() + guides(fill="none") + coord_cartesian(ylim = c(0, 15))+ stat_compare_means(label.x=1.6, label.y=11.25)

b


#combine these

figure <- ggarrange(a, b,labels = c("A", "B", ncol = 2, nrow = 1))

figure

#ggexport(figure, filename = "Sum of abundances boxplotAll 3mos.pdf")

#2023APR28_SumRelAbun_MGE_ARG_3mos_MR


#the each in boxplot = length of boxplot/9 (so 5688/9=632 or number of individuals)

boxplot<-c(abundance3mos$SumMGE,abundance3mos$Sumamino,abundance3mos$SumMDR, abundance3mos$Sumbeta, abundance3mos$Sumtetra,  abundance3mos$SumMLSB, abundance3mos$Sumsulfo, abundance3mos$Sumvanco, abundance3mos$Sumfluoro)

length(boxplot)

class<-rep(abundance3mos$class2,9)


type<-rep(c("MGE","aminoglycoside","MDR","betalactamase","tetracycline","MLSB","sulfonamide","vancomycin","fluoroquinolone"),each=311)

boxplot2<-data.frame(boxplot,type,class)

ab<-ggplot(boxplot2, aes(x = type, y = boxplot, fill=class)) +ylim(0,13)+ geom_boxplot(width=0.4,position=position_dodge(0.4)) + theme_minimal() + theme(axis.text.x = element_text(angle = 90)) + labs(title = "", y = "Sum of relative abundance by antibiotic class 3 mos", x = "") + stat_compare_means(aes(group = class),label="p.signif") + scale_fill_manual(values=c("#E69F00", "#56B4E9", "#E69F00"))+ theme(legend.position="top",)
```

```
ab

#ggsave("2023OCT08_SumAbudancesClassAll3mos_MR.tiff", units="in", width=7.5, height=5, dpi=300)

#ggsave("SumAbudancesClassAll3mos.tiff", units="in", width=7.5, height=5, dpi=300)
#2023APR28_SumAbudancesClassAll3mos_abxclass_MR

#stacked bar plot--#SumRelativeAbundanceMothervsChild
ID<-rep(abundance3mos$ID,9)

#reorder_size <- function(x) {
# factor(x, levels = names(sort(table(x), decreasing = TRUE)))}

sumabundance3mos<-aggregate(boxplot~type+class, boxplot2,median)
stackplot<-ggplot(data = sumabundance3mos, aes(x = class, y=boxplot)) + geom_bar(stat="identity", aes(fill=type))+theme_classic() + scale_fill_brewer(palette="Paired")+ labs(title = "", y = "Median of Normalized Abundance", x = "")+theme (axis.text = element_text(size=14), axis.title=element_text(size=14))
stackplot
#ggsave("2024MAY21_stackplot_NA_class_SF1.tiff", units="in", width=7.5, height=5, dpi=300)

#2023APR28_Stackplot_SumRAall_abxclass_MR

#moms= more amino, more MLSB, more tetra
#infants= more MGE, MDR, vanco
```

```
#####Shan and inv simpson####

presence<-presence3mos

presence2<- presence[,4:146]

presence2


abundance<-abundance3mos

abundance2<- abundance[,4:146]

abundance2[is.na(abundance2)]<- 0


abundance$class


#re-name to infancy and pregnancy

abundance$class2<-as.factor(ifelse(abundance$class== 'Mother','Pregnancy','Infancy'))

abundance$class2

abundance$class<-abundance$class2

abundance$class

indices <- abundance[,c("class","pair")]

head(indices)


#Sum of all antibiotic resistance genes found per class

#attaches the ID to the actual data set

#here she is attaching each indices to the row sums of presence/absence

#in doing so she is creating a relative abundance

indices$RichMGE<-rowSums(presence2[,1:27])
```

```
summary(indices$RichMGE)

indices$Richamino <- rowSums(presence2[,28:48])

summary(indices$Richamino)

indices$RichMDR<-rowSums(presence2[,49:69])

summary(indices$RichMDR)

indices$Richbeta<-rowSums(presence2[,70:85])

summary(indices$Richbeta)

indices$Richtetra<-rowSums(presence2[,86:98])

summary(indices$Richtetra)

indices$RichMLSB<-rowSums(presence2[,99:116])

summary(indices$RichMLSB)

indices$Richsulfo<-rowSums(presence2[,117:121])

summary(indices$Richsulfo)

indices$Richvanco<-rowSums(presence2[,122:129])

summary(indices$Richvanco)

indices$Richfluoro<-rowSums(presence2[,130:133])

summary(indices$Richfluoro)

indices$Richother<-rowSums(presence2[,134:143])

summary(indices$Richother)

indices$RichARG<-rowSums(presence2[,28:143])

summary(indices$RichARG)


table(indices$RichMGE)


mean(indices$RichARG)

#44.28939
```

```
sd(indices$RichARG)

#18.51695

indices$class

rich_Inf <- subset(indices, (class == "Infancy")) # subset

rich_Preg <- subset(indices, (class == "Pregnancy")) # subset


mean(rich_Inf$Richsulfo)

# [1] 40.09906 ARG 4.919811 amino 6.245283 MLSB 5.86 tetra #0.6603774 vanco 1.556604 fluro 2.099057 sulfo

sd(rich_Inf$Richsulfo)

#19.18986 ARG # 4.544481 amino 3.497626 MLSB 2.24 tetra 1.791961 vanco 1.031083 fluro 1.364617 sulfo

summary(rich_Inf$Richtetra)

# > summary(rich_Inf$RichARG)

# Min. 1st Qu.  Median    Mean 3rd Qu.    Max.

# 7.00   29.00   36.00   40.10   44.25  112.00


mean(rich_Preg$Richsulfo)


#53.26263 11.69697 amino 9.232323 MLSB 8.858586 tetra 2.89899 vanco 0.9191919 fluro 1.626263 sulfo

sd(rich_Preg$Richsulfo)

#13.13088 3.494762 amino 2.641228 MLSB 1.647572 tetra 1.681198 vacno

summary(rich_Preg$RichARG)

# summary(rich_Preg$RichARG)

# Min. 1st Qu.  Median    Mean 3rd Qu.    Max.

# 11.00   46.50   56.00   53.26   63.00   81.00
```

```
#stacked box plot

boxplot_rich<-c(indices$RichMGE,indices$Richamino,indices$RichMDR,
indices$Richbeta, indices$Richtetra,  indices$RichMLSB, indices$Richsulfo,
indices$Richvanco, indices$Richfluoro)

class<-rep(indices$class,9)

type<-
rep(c("MGE","aminoglycoside","MDR","betalactamase","tetracycline","MLSB","sulfonamide","
vancomycin","fluoroquinolone"),each=311)

length(type)


boxplot2<-data.frame(boxplot_rich,type,class)

boxplot2$type


levels(class)


#mothers greater amount of amino, MLSB, tetra, vanco

#infants greater amount of fluro, sulfo


rich_class<-ggplot(boxplot2, aes(x = type, y = boxplot, fill=class)) +

 ylim(0,30)+ geom_boxplot(width=0.4,position=position_dodge(0.4)) +

 theme_minimal() +

 theme(axis.text.x = element_text(size = 10, angle = 45)) +

 labs(title = "", y = "Richness", x = "") +

 stat_compare_means(aes(group = class),label="p.signif") +

 scale_fill_manual(values=c("#56B4E9", "#E69F00"))+ theme(legend.position="top",)
```

```
rich_class


#ggsave("2023MAY21_infancy_pregnancy_rich_abx_class_Figure_3a.tiff", units="in", width=7.5, height=5, dpi=300)

#2023APR28_RichnessClassAll3mos_abxclass_MR


#png("2023OCT08_RichnessClassAll3mos_abxclass_MR", res=300, height=7, width=7.5, units="in")


wilcox.test(RichARG~class, data=indices)

#p-value = < 2.2e-16

#amino, fluro, MGE, MLSP, sulf,. tetra, vanco

wilcox.test(RichMGE~class, data=indices)

#p-value = 0.6074


#Richness


b<-ggboxplot(data=indices, x="class", y="RichARG", color="black", fill="class",palette="d3", ylab="Richness", xlab="", outlier.colour = "black", outlier.shape = 1, main="Richness ARG")+
  theme_classic() +
  guides(fill=FALSE) +
  coord_cartesian(ylim = c(0, 125))+
  stat_compare_means(label.x=1.3, label.y=95)
b
```

```
a<-ggboxplot(data=indices, x="class", y="RichMGE", color="black", fill="class",palette="d3", ylab="Richness", xlab="", outlier.colour = "black", outlier.shape = 1, main="Richness MGE")+
  theme_classic() +
  guides(fill=FALSE) +
  coord_cartesian(ylim = c(0, 35)) +
  stat_compare_means(label.x=1.7, label.y=33)
a

#combine these
figure <- ggarrange(a, b,labels = c("A", "B", ncol = 2, nrow = 1))
figure
#ggexport(figure, filename = "RichMGE_ARG_All.pdf")

#ggsave("2023NOV30_RichMGE_ARG_All_MR.tiff", units="in", width=7.5, height=5, dpi=300)

#SHANNON INDEX

#shannon diversity index as default = Relative abundance
indices$ShannonMGERA<-diversity(abundance2[,1:27])
indices$ShannonaminoRA <- diversity(abundance2[,28:48])
indices$ShannonMDRRA<-diversity(abundance2[,49:69])
indices$ShannonbetaRA<-diversity(abundance2[,70:85])
indices$ShannontetraRA<-diversity(abundance2[,86:98])
indices$ShannonMLSBRA<-diversity(abundance2[,99:116])
indices$ShannonsulfoRA<-diversity(abundance2[,117:121])
```

```
indices$ShannonvancoRA<-diversity(abundance2[,122:129])

indices$ShannonfluoroRA<-diversity(abundance2[,130:133])

indices$ShannonotherRA<-diversity(abundance2[,134:143])

indices$ShannonARGRA<-diversity(abundance2[,28:143])



mean(indices$ShannonARGRA)

#1.938523

sd(indices$ShannonARGRA)

#0.5511772


shan_Inf <- subset(indices, (class == "Infancy")) # subset

shan_Preg <- subset(indices, (class == "Pregnancy")) # subset



mean(shan_Inf$ShannonvancoRA)

#1.98759 ARG 2.126075 amino 0.7753794 beta 0.7018619 tetra 1.90998 vanco

sd(shan_Inf$ShannonvancoRA)

#0.5581943 #  0.8785273 amino 0.7265166 beta 0.4389797 vanco


mean(shan_Preg$ShannonvancoRA)

#1.83345 ARG 1.71034 amino 0.3111938 beta 0.879 tetra 1.424014 vanco

sd(shan_Preg$ShannonvancoRA)

#0.5232344 0.421041 amino 0.4170454 beta 0.3026777 tetra 0.5497213 vanco


##shan tests
```

```
wilcox.test(ShannonARGRA~class, data=indices)

#p-value= 7.43e-12

wilcox.test(ShannonARGRA~class, data=indices)

#p-value = 0.00752

#shan-values: amino, beta, MGE, tetra, vanco



#shannon

d<-ggboxplot(data=indices, x="class", y="ShannonARGRA", color="black", fill="class",palette="d3", ylab="Shannon Diversity Index", xlab="", outlier.colour = "black", outlier.shape = 1, main="Shannon ARG")+ theme_classic() + guides(fill=FALSE) + coord_cartesian(ylim = c(0, 5.0))+ stat_compare_means(label.x=1.3, label.y=4.8)

d


c<-ggboxplot(data=indices, x="class", y="ShannonMGERA", color="black", fill="class",palette="d3", ylab="Shannon Diversity Index", xlab="", outlier.colour = "black", outlier.shape = 1, main="Shannon MGE")+ theme_classic() + guides(fill=FALSE) + coord_cartesian(ylim = c(0, 4.0))+ stat_compare_means(label.x=1.3, label.y=3.8)

c



#wilcoxin--MGE

#wilcoxon--p=0.021


#stacked box plot

boxplot_shan<-c(indices$ShannonMGERA,indices$ShannonaminoRA,indices$ShannonMDRRA, indices$ShannonbetaRA, indices$ShannontetraRA,  indices$ShannonMLSBRA, indices$ShannonsulfoRA, indices$ShannonvancoRA, indices$ShannonfluoroRA)
```

```
class<-rep(indices$class,9)

type<-
rep(c("MGE","aminoglycoside","MDR","betalactamase","tetracycline","MLSB","sulfonamide","
vancomycin","fluoroquinolone"),each=311)

length(type)

#infants have higher amino, beta, MGE

#moms have higher tetra and vanco


boxplot3<-data.frame(boxplot_shan,type,class)


shan_class<-ggplot(boxplot2, aes(x = type, y = boxplot, fill=class)) +ylim(0,4)+
geom_boxplot(width=0.4,position=position_dodge(0.4)) + theme_minimal() +
theme(axis.text.x = element_text(size= 10, angle = 45)) + labs(title = "", y = "Shannon
Diversity Index", x = "") + stat_compare_means(aes(group = class),label="p.signif") +
scale_fill_manual(values=c("#56B4E9", "#E69F00"))+ theme(legend.position="top",)

shan_class

ggsave("2023MAY21_infancy_pregnancy_shan_abx_class_Figure_3b.tiff", units="in",
width=7.5, height=5, dpi=300)

#2023APR28_Rich_shan_BP_class_All3mos_MR


#stacked bar plot

sumabundance<-aggregate(boxplot~type+class, boxplot2,median)

stackplot<-ggplot(data = sumabundance, aes(x = class, y=boxplot)) +
geom_bar(stat="identity", aes(fill=type))+theme_classic() +
scale_fill_brewer(palette="Paired")+ labs(title = "", y = "Shannon Index", x = "")+theme
(axis.text = element_text(size=14), axis.title=element_text(size=14))

stackplot

#ggexport(stackplot, filename = "/Users/madeleinerussell/Desktop/Comstock
Lab/AbxResistance/ABXR_Ranalysis/ShannonDiv_ALL.pdf")
```

```
#2023APR28_stackplot_shan_class_All3mos_MR

#ggsave("2023NOV30_stackplot_shan_class_All3mos_MR_MR.tiff", units="in", width=7.5, height=5, dpi=300)

#2023APR28_Rich_shan_BP_class_All3mos_MR



#INVERSE SIMPSON INDEX

#Inverse Simpson diversity index = Relative abundance

indices$SimpsonMGERA<-diversity(abundance2[,1:27], index="invsimpson")

indices$SimpsonaminoRA<-diversity(abundance2[,28:48], index="invsimpson")

indices$SimpsonMDRRA<-diversity(abundance2[,49:69], index="invsimpson")

indices$SimpsonbetaRA<-diversity(abundance2[,70:85], index="invsimpson")

indices$SimpsontetraRA<-diversity(abundance2[,86:98], index="invsimpson")

indices$SimpsonMLSBRA<-diversity(abundance2[,99:116], index="invsimpson")

indices$SimpsonsulfoRA<-diversity(abundance2[,117:121], index="invsimpson")

indices$SimpsonvancoRA<-diversity(abundance2[,122:129], index="invsimpson")

indices$SimpsonfluoroRA<-diversity(abundance2[,130:133], index="invsimpson")

indices$SimpsonotherRA<-diversity(abundance2[,134:143], index="invsimpson")

indices$SimpsonARGRA<-diversity(abundance2[,28:143], index="invsimpson")



mean(indices$SimpsonARGRA)

#5.657836

sd(indices$SimpsonARGRA)

#3.500716
```

```
class<-indices$class

SimpsonvancoRA<-indices$SimpsonvancoRA

tapply(SimpsonvancoRA, class, summary)


simp_Inf <- subset(indices, (class == "Infancy")) # subset

simp_Preg <- subset(indices, (class == "Pregnancy")) # subset


median(simp_Inf$SimpsonvancoRA)

#5.929954 aRG 9.2212 amino 2.395676 beta 1.855746 tetra 7.074994 vanco

sd(simp_Inf$SimpsonvancoRA)

#3.624068 arg 7.098675 amino 2.726942 beta 1.143665 tetra 2.111149 vanco


mean(simp_Preg$SimpsonvancoRA)

#5.929954 ARG 4.633676 amino 1.387228 beta 2.124203 tetra 3.978111 vanco

sd(simp_Preg$SimpsonvancoRA)

#3.159845 arg 2.332543 amino 1.38403 beta 0.7068627 tetra 2.210344 vanco



#inv simp

wilcox.test(SimpsonMGERA~class, data=indices)

#p-value =  5.381e-12

wilcox.test(SimpsonARGRA~class, data=indices)

#p-value = 0.01057


indices$SimpsonvancoRA
```

```
#invsimp

e<-ggboxplot(data=indices, x="class", y="SimpsonMGERA", color="black", fill="class",palette="d3", ylab="Inverse Simpson Diversity Index", xlab="", outlier.colour = "black", outlier.shape = 1, main="Inverse Simpson MGE")+ theme_classic() + guides(fill=FALSE) + coord_cartesian(ylim = c(0, 8.0))+ stat_compare_means(label.x=1.3, label.y=7.5)

e

f<-ggboxplot(data=indices, x="class", y="SimpsonARGRA", color="black", fill="class",palette="d3", ylab="Inverse Simpson Diversity Index", xlab="", outlier.colour = "black", outlier.shape = 1, main="Inverse Simpson ARG")+ theme_classic() + guides(fill=FALSE) + coord_cartesian(ylim = c(0, 21.0))+ stat_compare_means(label.x=1.3, label.y=20.5)

f


#combine all 6

#

figure <- ggarrange(b, d, f, a, c, e, labels = c("A", "B","C","D"," E", "F", ncol = 2, nrow = 3))

figure



#dev.off()

#ggsave("2023MAY21_InvSimp_MGE_ARG_All_MR.tiff", units="in", width=9, height=5, dpi=300)

#2023APR28_Rich_shan_BP_class_All3mos_MR


#ggexport(figure, filename = "InvSimp_MGE_ARG_All.pdf")
```

```
boxplot_simp<-
c(indices$SimpsonMGERA,indices$SimpsonaminoRA,indices$SimpsonMDRRA,
indices$SimpsonbetaRA, indices$SimpsontetraRA,  indices$SimpsonMLSBRA,
indices$SimpsonsulfoRA, indices$SimpsonvancoRA, indices$SimpsonfluoroRA)

class<-rep(indices$class,9)

type<-
rep(c("MGE","aminoglycoside","MDR","betalactamase","tetracycline","MLSB","sulfonamide","
vancomycin","fluoroquinolone"),each=311)

length(boxplot)


boxplot4<-data.frame(boxplot_simp,type,class)


inv_class<-ggplot(boxplot2, aes(x = type, y = boxplot, fill=class)) +ylim(1,25)+
geom_boxplot(width=0.4,position=position_dodge(0.4)) + theme_minimal() +
theme(axis.text.x = element_text(size =10, angle = 45)) + labs(title = "", y = "Inverse Simpson
Diversity Index", x = "") + stat_compare_means(aes(group = class),label="p.signif") +
scale_fill_manual(values=c("#56B4E9", "#E69F00"))+ theme(legend.position="top",)

inv_class


ggsave("2023MAY21_infancy_pregnancy_invsimp_abx_class_Figure_3c.tiff", units="in",
width=7.5, height=5, dpi=300)


figure <- ggarrange(rich_class, shan_class, inv_class, labels = c("A", "B","C"))

figure

grid.arrange(rich_class, shan_class, inv_class, nrow = 3)


# library(cowplot)

# #moms higher amino, tetra, vanco

# #infants higher in beta, MGE
```

```
#
# av<-plot_grid(rich_class, shan_class, inv_class, labels = c('A', 'B', 'C'), label_size = 12)
# av
# par(mfrow=c(3,1))
#
# rich_class
# shan_class
# inv_class


#aug 24 2025


# Richness MGE
a <- ggboxplot(
  data = indices, x = "class", y = "RichMGE",
  color = "black", fill = "class", palette = "d3",
  ylab = "Richness", xlab = "",
  outlier.colour = "black", outlier.shape = 1,
  main = "Richness MGE"
) +
  theme_classic() +
  guides(fill = FALSE) +
  coord_cartesian(ylim = c(0, 35)) +
```

```
  stat_compare_means(label.x = 1.5, label.y = 34, size = 4, fontface = "bold") +  # center +
high
  theme(
    axis.text.x   = element_text(size = 14),
    axis.text.y   = element_text(size = 14),
    axis.title.y  = element_text(size = 16),
    plot.title    = element_text(size = 18, face = "bold")
  )


# Richness ARG
b <- ggboxplot(
  data = indices, x = "class", y = "RichARG",
  color = "black", fill = "class", palette = "d3",
  ylab = "Richness", xlab = "",
  outlier.colour = "black", outlier.shape = 1,
  main = "Richness ARG"
) +
  theme_classic() +
  guides(fill = FALSE) +
  coord_cartesian(ylim = c(0, 125)) +
  stat_compare_means(label.x = 1.5, label.y = 120, size = 4, fontface = "bold") +  # center +
high
  theme(
    axis.text.x   = element_text(size = 14),
    axis.text.y   = element_text(size = 14),
    axis.title.y  = element_text(size = 16),
```

```
    plot.title    = element_text(size = 18, face = "bold")
  )


d <- ggboxplot(
  data = indices, x = "class", y = "ShannonARGRA",
  color = "black", fill = "class", palette = "d3",
  ylab = "Shannon", xlab = "",
  outlier.colour = "black", outlier.shape = 1,
  main = "Shannon ARG"
) +
  theme_classic() +
  guides(fill = FALSE) +
  coord_cartesian(ylim = c(0, 5.0)) +
  stat_compare_means(label.x = 1.5, label.y = 4.9, size = 4, fontface = "bold") +  # centered +
high + bold
  theme(
    axis.text.x   = element_text(size = 14),
    axis.text.y   = element_text(size = 14),
    axis.title.y  = element_text(size = 16),
    plot.title    = element_text(size = 18, face = "bold")
  )
d

c <- ggboxplot(
  data = indices, x = "class", y = "ShannonMGERA",
```

```
  color = "black", fill = "class", palette = "d3",
  ylab = "Shannon", xlab = "",
  outlier.colour = "black", outlier.shape = 1,
  main = "Shannon MGE"
) +
  theme_classic() +
  guides(fill = FALSE) +
  coord_cartesian(ylim = c(0, 4.0)) +
  stat_compare_means(label.x = 1.5, label.y = 3.9, size = 4, fontface = "bold") +  # centered +
high
  theme(
    axis.text.x   = element_text(size = 14),
    axis.text.y   = element_text(size = 14),
    axis.title.y  = element_text(size = 16),
    plot.title    = element_text(size = 18, face = "bold")
  )
c


e <- ggboxplot(
  data = indices, x = "class", y = "SimpsonMGERA",
  color = "black", fill = "class", palette = "d3",
  ylab = "Inverse Simpson", xlab = "",
  outlier.colour = "black", outlier.shape = 1,
  main = "Inverse Simpson MGE"
) +
  theme_classic() +
```

```
  guides(fill = FALSE) +
  coord_cartesian(ylim = c(0, 8.0)) +
  stat_compare_means(label.x = 1.5, label.y = 7.8, size = 4, fontface = "bold") +  # centered +
high
  theme(
    axis.text.x   = element_text(size = 14),
    axis.text.y   = element_text(size = 14),
    axis.title.y  = element_text(size = 16),
    plot.title    = element_text(size = 18, face = "bold")
  )
e

f <- ggboxplot(
  data = indices, x = "class", y = "SimpsonARGRA",
  color = "black", fill = "class", palette = "d3",
  ylab = "Inverse Simpson", xlab = "",
  outlier.colour = "black", outlier.shape = 1,
  main = "Inverse Simpson ARG"
) +
  theme_classic() +
  guides(fill = FALSE) +
  coord_cartesian(ylim = c(0, 21.0)) +
  stat_compare_means(label.x = 1.5, label.y = 20, size = 4, fontface = "bold") +  # centered +
high
  theme(
    axis.text.x   = element_text(size = 14),
    axis.text.y   = element_text(size = 14),
```

```
    axis.title.y  = element_text(size = 16),
    plot.title    = element_text(size = 18, face = "bold")
  )
f

combined <- ggarrange(
  b, a,    # Row 1
  d, c,    # Row 2
  f, e,    # Row 3
  ncol = 2, nrow = 3,
  labels = c("A", "B", "C", "D", "E", "F"),
  font.label = list(size = 12, face = "bold"),
  align = "hv"
)

combined

# Save as high-res TIFF optimized for journal

ggsave(
  filename = "diversity_panels_final.tiff",
  plot = combined,
  device = "tiff",
  width = 6.5, height = 9, units = "in",   # max publication size
  dpi = 600,
  compression = "lzw"
```

```
)

#test 2
# Custom label function for p-values
pval_fmt <- function(p) {
  ifelse(p < 0.001, "p < 0.001", sprintf("p = %.3f", p))
}

# Richness MGE
a <- ggboxplot(
  data = indices, x = "class", y = "RichMGE",
  color = "black", fill = "class", palette = "d3",
  ylab = "Richness", xlab = "",
  outlier.colour = "black", outlier.shape = 1,
  main = "Richness MGE"
) +
  theme_classic() +
  guides(fill = FALSE) +
  coord_cartesian(ylim = c(0, 35)) +
  stat_compare_means(
    label.x = 1.5, label.y = 34, size = 4, fontface = "bold",
    label = pval_fmt
  ) +
  theme(
    axis.text.x   = element_text(size = 14),
    axis.text.y   = element_text(size = 14),
```

```
    axis.title.y  = element_text(size = 16),
    plot.title    = element_text(size = 18, face = "bold")
  )


# Richness ARG
b <- ggboxplot(
  data = indices, x = "class", y = "RichARG",
  color = "black", fill = "class", palette = "d3",
  ylab = "Richness", xlab = "",
  outlier.colour = "black", outlier.shape = 1,
  main = "Richness ARG"
) +
  theme_classic() +
  guides(fill = FALSE) +
  coord_cartesian(ylim = c(0, 125)) +
  stat_compare_means(
    label.x = 1.5, label.y = 120, size = 4, fontface = "bold",
    label = pval_fmt
  ) +
  theme(
    axis.text.x   = element_text(size = 14),
    axis.text.y   = element_text(size = 14),
    axis.title.y  = element_text(size = 16),
    plot.title    = element_text(size = 18, face = "bold")
  )
```

```
# Shannon ARG
d <- ggboxplot(
  data = indices, x = "class", y = "ShannonARGRA",
  color = "black", fill = "class", palette = "d3",
  ylab = "Shannon", xlab = "",
  outlier.colour = "black", outlier.shape = 1,
  main = "Shannon ARG"
) +
  theme_classic() +
  guides(fill = FALSE) +
  coord_cartesian(ylim = c(0, 5.0)) +
  stat_compare_means(
    label.x = 1.5, label.y = 4.9, size = 4, fontface = "bold",
    label = pval_fmt
  ) +
  theme(
    axis.text.x   = element_text(size = 14),
    axis.text.y   = element_text(size = 14),
    axis.title.y  = element_text(size = 16),
    plot.title    = element_text(size = 18, face = "bold")
  )


# Shannon MGE
```

```
c <- ggboxplot(
  data = indices, x = "class", y = "ShannonMGERA",
  color = "black", fill = "class", palette = "d3",
  ylab = "Shannon", xlab = "",
  outlier.colour = "black", outlier.shape = 1,
  main = "Shannon MGE"
) +
  theme_classic() +
  guides(fill = FALSE) +
  coord_cartesian(ylim = c(0, 4.0)) +
  stat_compare_means(
    label.x = 1.5, label.y = 3.9, size = 4, fontface = "bold",
    label = pval_fmt
  ) +
  theme(
    axis.text.x   = element_text(size = 14),
    axis.text.y   = element_text(size = 14),
    axis.title.y  = element_text(size = 16),
    plot.title    = element_text(size = 18, face = "bold")
  )


# Inverse Simpson MGE
e <- ggboxplot(
  data = indices, x = "class", y = "SimpsonMGERA",
  color = "black", fill = "class", palette = "d3",
```

```
  ylab = "Inverse Simpson", xlab = "",
  outlier.colour = "black", outlier.shape = 1,
  main = "Inverse Simpson MGE"
) +
  theme_classic() +
  guides(fill = FALSE) +
  coord_cartesian(ylim = c(0, 8.0)) +
  stat_compare_means(
    label.x = 1.5, label.y = 7.8, size = 4, fontface = "bold",
    label = pval_fmt
  ) +
  theme(
    axis.text.x   = element_text(size = 14),
    axis.text.y   = element_text(size = 14),
    axis.title.y  = element_text(size = 16),
    plot.title    = element_text(size = 18, face = "bold")
  )


# Inverse Simpson ARG
f <- ggboxplot(
  data = indices, x = "class", y = "SimpsonARGRA",
  color = "black", fill = "class", palette = "d3",
  ylab = "Inverse Simpson", xlab = "",
  outlier.colour = "black", outlier.shape = 1,
  main = "Inverse Simpson ARG"
```

```
) +
  theme_classic() +
  guides(fill = FALSE) +
  coord_cartesian(ylim = c(0, 21.0)) +
  stat_compare_means(
    label.x = 1.5, label.y = 20, size = 4, fontface = "bold",
    label = pval_fmt
  ) +
  theme(
    axis.text.x   = element_text(size = 14),
    axis.text.y   = element_text(size = 14),
    axis.title.y  = element_text(size = 16),
    plot.title    = element_text(size = 18, face = "bold")
  )


# Combine plots
combined <- ggarrange(
  b, a,
  d, c,
  f, e,
  ncol = 2, nrow = 3,
  labels = c("A", "B", "C", "D", "E", "F"),
  font.label = list(size = 12, face = "bold"),
  align = "hv"
)
```

```
combined

# Save for publication
ggsave(
  filename = "diversity_panels_final.tiff",
  plot = combined,
  device = "tiff",
  width = 6.5, height = 9, units = "in",
  dpi = 600,
  compression = "lzw"
)


# ---- Custom P-value Formatter ----
pval_fmt <- function(p) {
  ifelse(p < 0.001, "p < 0.001", sprintf("p = %.3f", p))
}

# ---- Richness MGE ----
a <- ggboxplot(
  data = indices, x = "class", y = "RichMGE",
  color = "black", fill = "class", palette = "d3",
  ylab = "Richness", xlab = "",
  outlier.colour = "black", outlier.shape = 1,
  main = "Richness MGE"
```

```
) +
  theme_classic() +
  guides(fill = FALSE) +
  coord_cartesian(ylim = c(0, 35)) +
  stat_compare_means(
    aes(label = after_stat(pval_fmt(p))),
    label.x = 1.5, label.y = 34, size = 5, fontface = "bold"
  ) +
  theme(
    axis.text.x   = element_text(size = 14),
    axis.text.y   = element_text(size = 14),
    axis.title.y  = element_text(size = 16),
    plot.title    = element_text(size = 16, face = "bold")
  )


# ---- Richness ARG ----
b <- ggboxplot(
  data = indices, x = "class", y = "RichARG",
  color = "black", fill = "class", palette = "d3",
  ylab = "Richness", xlab = "",
  outlier.colour = "black", outlier.shape = 1,
  main = "Richness ARG"
) +
  theme_classic() +
  guides(fill = FALSE) +
```

```
  coord_cartesian(ylim = c(0, 125)) +
  stat_compare_means(
    aes(label = after_stat(pval_fmt(p))),
    label.x = 1.5, label.y = 120, size = 5, fontface = "bold"
  ) +
  theme(
    axis.text.x   = element_text(size = 14),
    axis.text.y   = element_text(size = 14),
    axis.title.y  = element_text(size = 16),
    plot.title    = element_text(size = 16, face = "bold")
  )


# ---- Shannon ARG ----
d <- ggboxplot(
  data = indices, x = "class", y = "ShannonARGRA",
  color = "black", fill = "class", palette = "d3",
  ylab = "Shannon", xlab = "",
  outlier.colour = "black", outlier.shape = 1,
  main = "Shannon ARG"
) +
  theme_classic() +
  guides(fill = FALSE) +
  coord_cartesian(ylim = c(0, 5.0)) +
  stat_compare_means(
    aes(label = after_stat(pval_fmt(p))),
```

```r
    label.x = 1.5, label.y = 4.9, size = 5, fontface = "bold"
  ) +
  theme(
    axis.text.x   = element_text(size = 14),
    axis.text.y   = element_text(size = 14),
    axis.title.y  = element_text(size = 16),
    plot.title    = element_text(size = 16, face = "bold")
  )


# ---- Shannon MGE ----
c <- ggboxplot(
  data = indices, x = "class", y = "ShannonMGERA",
  color = "black", fill = "class", palette = "d3",
  ylab = "Shannon", xlab = "",
  outlier.colour = "black", outlier.shape = 1,
  main = "Shannon MGE"
) +
  theme_classic() +
  guides(fill = FALSE) +
  coord_cartesian(ylim = c(0, 4.0)) +
  stat_compare_means(
    aes(label = after_stat(pval_fmt(p))),
    label.x = 1.5, label.y = 3.9, size = 5, fontface = "bold"
  ) +
  theme(
```

```
    axis.text.x   = element_text(size = 14),
    axis.text.y   = element_text(size = 14),
    axis.title.y  = element_text(size = 16),
    plot.title    = element_text(size = 16, face = "bold")
  )


# ---- Inverse Simpson MGE ----
e <- ggboxplot(
  data = indices, x = "class", y = "SimpsonMGERA",
  color = "black", fill = "class", palette = "d3",
  ylab = "Inverse Simpson", xlab = "",
  outlier.colour = "black", outlier.shape = 1,
  main = "Inverse Simpson MGE"
) +
  theme_classic() +
  guides(fill = FALSE) +
  coord_cartesian(ylim = c(0, 8.0)) +
  stat_compare_means(
    aes(label = after_stat(pval_fmt(p))),
    label.x = 1.5, label.y = 7.8, size = 5, fontface = "bold"
  ) +
  theme(
    axis.text.x   = element_text(size = 14),
    axis.text.y   = element_text(size = 14),
    axis.title.y  = element_text(size = 16),
```

```
    plot.title    = element_text(size = 16, face = "bold")
  )


# ---- Inverse Simpson ARG ----
f <- ggboxplot(
  data = indices, x = "class", y = "SimpsonARGRA",
  color = "black", fill = "class", palette = "d3",
  ylab = "Inverse Simpson", xlab = "",
  outlier.colour = "black", outlier.shape = 1,
  main = "Inverse Simpson ARG"
) +
  theme_classic() +
  guides(fill = FALSE) +
  coord_cartesian(ylim = c(0, 21.0)) +
  stat_compare_means(
    aes(label = after_stat(pval_fmt(p))),
    label.x = 1.5, label.y = 20, size = 5, fontface = "bold"
  ) +
  theme(
    axis.text.x   = element_text(size = 14),
    axis.text.y   = element_text(size = 14),
    axis.title.y  = element_text(size = 16),
    plot.title    = element_text(size = 16, face = "bold")
  )
```

```
# ---- Combine all panels ----
combined <- ggarrange(
  b, a,    # Row 1
  d, c,    # Row 2
  f, e,    # Row 3
  ncol = 2, nrow = 3,
  labels = c("A", "B", "C", "D", "E", "F"),
  font.label = list(size = 12, face = "bold"),
  align = "hv"
)

combined

# ---- Save high-res ----
ggsave(
  filename = "diversity_panels_final.tiff",
  plot = combined,
  device = "tiff",
  width = 6.5, height = 9, units = "in",
  dpi = 600,
  compression = "lzw"
)
```

```
library(ggplot2)

library(ggpubr)

library(gridExtra)


# ----- three diversity plots (already adjusted) -----

# Remove x text and ticks from ALL panels

base_theme <- theme_classic() +

 theme(

  axis.text.x = element_blank(),

  axis.ticks.x = element_blank(),

  axis.title.x = element_blank()

 )


rich_class <- ggplot(boxplot2, aes(x = type, y = boxplot_rich, fill = class)) +

 geom_boxplot(width = 0.4, position = position_dodge(0.4)) +

 ylim(0, 30) +

 base_theme +

 theme(axis.text.y = element_text(size = 12),

     axis.title.y = element_text(size = 13, face = "bold"),

     legend.position = "none") +

 labs(y = "Richness") +

 stat_compare_means(aes(group = class), label = "p.signif",

           size = 4, fontface = "bold") +

 scale_fill_manual(values = c("#56B4E9", "#E69F00"))
```

```
shan_class <- ggplot(boxplot3, aes(x = type, y = boxplot_shan, fill = class)) +
 geom_boxplot(width = 0.4, position = position_dodge(0.4)) +
 ylim(0, 4) +
 base_theme +
 theme(axis.text.y = element_text(size = 12),
      axis.title.y = element_text(size = 13, face = "bold"),
      legend.position = "none") +
 labs(y = "Shannon") +
 stat_compare_means(aes(group = class), label = "p.signif",
                size = 4, fontface = "bold") +
 scale_fill_manual(values = c("#56B4E9", "#E69F00"))

inv_class <- ggplot(boxplot4, aes(x = type, y = boxplot_simp, fill = class)) +
 geom_boxplot(width = 0.4, position = position_dodge(0.4)) +
 ylim(0, 25) +
 base_theme +
 theme(axis.text.y = element_text(size = 12),
      axis.title.y = element_text(size = 13, face = "bold"),
      legend.position = "top",
      legend.title = element_blank(),
      legend.text = element_text(size = 12)) +
 labs(y = "Inverse Simpson") +
 stat_compare_means(aes(group = class), label = "p.signif",
                size = 4, fontface = "bold") +
 scale_fill_manual(values = c("#56B4E9", "#E69F00"))
```

```
# ----- Combine the three main plots -----

combined_div <- ggarrange(

  rich_class, shan_class, inv_class,

  ncol = 1, nrow = 3,

  labels = c("A", "B", "C"),

  font.label = list(size = 12, face = "bold"),

  align = "v",

  common.legend = TRUE, legend = "top",

  heights = c(1, 1, 1)

)


# ----- Create a dummy plot just for x labels -----

drug_classes <- c("aminoglycoside","betalactamase","fluoroquinolone", "MDR", "MGE",
"MLSB", "sulfonamide",

                  "tetracycline","vancomycin")


xlab_plot <- ggplot(data.frame(type = factor(drug_classes, levels = drug_classes), y = 0),

                    aes(x = type, y = y)) +

  geom_blank() +

  theme_void() +

  theme(

    axis.text.x = element_text(size = 11, angle = 45, hjust = 0.5, vjust = 0.7),

    axis.ticks.x = element_blank()

  ) +

  labs(x = "ARG Class")
```

```
# ----- Stack main figure + shared x axis -----
final_plot <- grid.arrange(combined_div, xlab_plot, heights = c(9, 1))

# ----- Save -----
ggsave(
  filename = "2025AUG28_class_fig2_test1.tiff",
  plot = final_plot,
  device = "tiff",
  width = 6.5, height = 9, units = "in",
  dpi = 600,
  compression = "lzw",
  bg = "white"
)

# ----- Shared base theme -----
base_theme <- theme_classic() +
  theme(
    axis.text.x = element_blank(),
    axis.ticks.x = element_blank(),
    axis.title.x = element_blank()
  )

# ----- Richness -----
rich_class2 <- ggplot(boxplot2_mod, aes(x = type, y = value, fill = mode)) +
  geom_boxplot(width = 0.4, position = position_dodge(0.4)) +
```

```
  ylim(0, 30) +
  base_theme +
  theme(axis.text.y = element_text(size = 12),
        axis.title.y = element_text(size = 13, face = "bold"),
        legend.position = "none") +
  labs(y = "Richness") +
  stat_compare_means(aes(group = mode), label = "p.signif",
                     size = 4, fontface = "bold") +
  scale_fill_manual(values = c("#56B4E9", "#E69F00"))

# ----- Shannon -----
shan_class2 <- ggplot(boxplot2_shan_mod, aes(x = type, y = value, fill = mode)) +
  geom_boxplot(width = 0.4, position = position_dodge(0.4)) +
  ylim(0, 4) +
  base_theme +
  theme(axis.text.y = element_text(size = 12),
        axis.title.y = element_text(size = 13, face = "bold"),
        legend.position = "none") +
  labs(y = "Shannon") +
  stat_compare_means(aes(group = mode), label = "p.signif",
                     size = 4, fontface = "bold") +
  scale_fill_manual(values = c("#56B4E9", "#E69F00"))

# ----- Inverse Simpson -----
inv_class2 <- ggplot(boxplot2_simp_mod, aes(x = type, y = value, fill = mode)) +
  geom_boxplot(width = 0.4, position = position_dodge(0.4)) +
```

```
  ylim(0, 12) +
  base_theme +
  theme(axis.text.y = element_text(size = 12),
        axis.title.y = element_text(size = 13, face = "bold"),
        legend.position = "top",
        legend.title = element_blank(),
        legend.text = element_text(size = 12)) +
  labs(y = "Inverse Simpson") +
  stat_compare_means(aes(group = mode), label = "p.signif",
                     size = 4, fontface = "bold") +
  scale_fill_manual(values = c("#56B4E9", "#E69F00"))

# ----- Combine vertically with shared legend -----
combined_div2 <- ggarrange(
  rich_class2, shan_class2, inv_class2,
  ncol = 1, nrow = 3,
  labels = c("A", "B", "C"),
  font.label = list(size = 12, face = "bold"),
  align = "v",
  common.legend = TRUE, legend = "top",
  heights = c(1, 1, 1)
)

# ----- Dummy x-axis for ARG classes -----
drug_classes <- c("aminoglycoside","betalactamase","fluoroquinolone", "MDR", "MGE",
"MLSB", "sulfonamide",
```

```
        "tetracycline","vancomycin")

xlab_plot2 <- ggplot(data.frame(type = factor(drug_classes, levels = drug_classes), y = 0),
         aes(x = type, y = y)) +
  geom_blank() +
  theme_void() +
  theme(
    axis.text.x = element_text(size = 12, angle = 45, hjust = 0.5, vjust = 0.7),
    axis.ticks.x = element_blank()
  ) +
  labs(x = "ARG Class")

# ----- Stack plots + shared x labels -----
final_plot2 <- grid.arrange(combined_div2, xlab_plot2, heights = c(9, 1))

# ----- Save publication-ready TIFF -----
ggsave(
  filename = "diversity_indices_mode_shared_xaxis_labels.tiff",
  plot = final_plot2,
  device = "tiff",
  width = 6.5, height = 9, units = "in",
  dpi = 600,
  compression = "lzw",
  bg = "white"
)
```

```
#stopped here
#####Beta Diversity####

presence2<- presence3mos[,4:146]
abundance2<-abundance3mos[,4:146]
abun<-abundance2[28:143]
head(abun)

dev.off()
#par(mfrow = c(2,2),mar=c(4,4.3,1,1))
# line = -2
# cex = 2
# adj  = 0.025
#bray curtis
Sor.bray.pcoa<-function(abun,Dim=2,Color=1,binary,pch=16,Title="Bray-Curtis MGE"){
 Data.df<-vegdist(abun,method="bray", binary)
 Data.df.PCoA<-cmdscale(Data.df, k = Dim, eig = FALSE)
 Data.df.PCoA.eig<-cmdscale(Data.df, k = Dim, eig = TRUE)
 eig.Data.df.PCoA<-Data.df.PCoA.eig$eig
 eig.Data.df.PCoA.sum<-sum(eig.Data.df.PCoA)
 a<-(eig.Data.df.PCoA/eig.Data.df.PCoA.sum)*100
 xlab<-paste("PC1","(",round(a[1],1),"%",")",sep="")
 ylab<-paste("PC2","(",round(a[2],1),"%",")",sep="")
 if(binary==TRUE){
  main<-"Sorensen PCoA"
```

```
  }else(main<-"Bray-Curtis PCoA")
  plot(Data.df.PCoA, col=Color,
     main=Title,xlab=xlab,ylab=ylab,pch=c(pch))
  return(Data.df.PCoA)
}

#for MGE, 1:27
#for ARG, 28:143
#set binary = false for bray, binary = true for Sorensen
abun<-abundance2[1:27]
b<-as.factor(abundance$class)
abundance$class

Color.Class<-ifelse(grepl("Infancy", abundance$class),"#56B4E9", "#E69F00")

df.spe.bray.Sor<-Sor.bray.pcoa(abun, Dim = 2, Color = Color.Class, binary = FALSE)
#PC1 (22.9%)
#plot(df.spe.bray.Sor,cex.axis=1.5,cex.lab=1.5,cex.main=2,col=1,
#   pch=21,cex=2,xlim=c(-.45,.65),ylim=c(-.6,.45),xlab="PC1 (22.9%)",ylab="PC2
(16%)",bg=Color.Class)
ordiellipse(df.spe.bray.Sor,abundance$class,col=c("#56B4E9", "#E69F00"),lwd=2,bg=2)
#legend(.25,-.33,c("Normal","Overweight","Obese"),
#      pch=21,col=1,pt.bg=c("#000000","#E79F00","#0072B2"))
title(outer=outer,adj=adj,main="A",cex.main=cex,col="black",font=2,line=line)
text(x = 0.40, y = 0.43, labels = "p = 0.01", xpd = NA,cex=2)
# text(x = -0.43, y = .43, labels = "A", xpd = NA,cex=2.5)
```

```
# ordiellipse(df.spe.bray.Sor,groups=b,col=c(1,2),lwd=1)

# text(x = -0.43, y = .43, labels = "A", xpd = NA,cex=2.5)

#legend(-0.4,0.35,c("Infancy","Pregnancy"), pch=21,col=1,pt.bg=c(1,2))

#text(df.spe.bray.Sor,labels=b,col=as.numeric(b))

#2023APR28_bray_MGE_class_All3mos_MR

#png("2023OCT08_bray_MGE_class_All3mos_MR", res=300, height=7, width=7.5, units="in")



#permanova

PERMANOVA<-function(abun,Group,binary,iters=9999){

  Data.Dist<-vegdist(abun,method="bray", binary=binary)

  adonis2(Data.Dist~Group,permutations=iters)

}

PERMDISP<-function(abun,Group,binary,iters=9999){

  Data.Dist<-vegdist(abun,method="bray", binary=binary)

  Data.betadisper<-betadisper(Data.Dist, group=Group)

  permutest(Data.betadisper, group=Group, permutations=iters)

}



#ab<-PERMANOVA(abundance2[,c(1:27)],b,FALSE,9999)

#p-value= 1e-04 ***

#PERMDISP(abundance2[,c(1:27)],b,FALSE,9999)

#1e-04 ***
```

```
#Sorensen--Change title if need
abun<-presence2[1:27]
Sor.bray.pcoa<-function(abun,Dim=2,Color=1,binary,pch=16,Title="Sorensen MGE"){
 Data.df<-vegdist(abun,method="bray", binary)
 Data.df.PCoA<-cmdscale(Data.df, k = Dim, eig = FALSE)
 Data.df.PCoA.eig<-cmdscale(Data.df, k = Dim, eig = TRUE)
 eig.Data.df.PCoA<-Data.df.PCoA.eig$eig
 eig.Data.df.PCoA.sum<-sum(eig.Data.df.PCoA)
 a<-(eig.Data.df.PCoA/eig.Data.df.PCoA.sum)*100
 xlab<-paste("PC1","(",round(a[1],1),"%",")",sep="")
 ylab<-paste("PC2","(",round(a[2],1),"%",")",sep="")
 if(binary==TRUE){
  main<-"Sorensen PCoA"
 }else(main<-"Bray-Curtis PCoA")
 plot(Data.df.PCoA, col=Color,
    main=Title,xlab=xlab,ylab=ylab,pch=c(pch))
 return(Data.df.PCoA)
}
#sor
b<-as.factor(abundance$class)
df.spe.bray.Sor<-Sor.bray.pcoa(abun, Dim = 2, Color = Color.Class, binary = TRUE)
ordiellipse(df.spe.bray.Sor,abundance$class,col=c("#56B4E9", "#E69F00"),lwd=2,bg=2)
#ordiellipse(df.spe.bray.Sor,groups=b,col=c(1,2),lwd=1)
title(outer=outer,adj=adj,main="B",cex.main=cex,col="black",font=2,line=line)
text(x = 0.3, y = 0.5, labels = "p < 0.001", xpd = NA,cex=2)
```

```
#text(x = -0.38, y = .50, labels = "B", xpd = NA,cex=2.5)

#legend(-0.3,0.4,c("Infancy","Pregnancy"), pch=21,col=1,pt.bg=c(1,2))

#text(df.spe.bray.Sor,labels=b,col=as.numeric(b))

#2023APR28_sor_MGE_class_All3mos_MR

#



#ARG

#bray curtis

Sor.bray.pcoa<-function(abun,Dim=2,Color=1,binary,pch=16,Title="Bray-Curtis ARG"){

 Data.df<-vegdist(abun,method="bray", binary)

 Data.df.PCoA<-cmdscale(Data.df, k = Dim, eig = FALSE)

 Data.df.PCoA.eig<-cmdscale(Data.df, k = Dim, eig = TRUE)

 eig.Data.df.PCoA<-Data.df.PCoA.eig$eig

 eig.Data.df.PCoA.sum<-sum(eig.Data.df.PCoA)

 a<-(eig.Data.df.PCoA/eig.Data.df.PCoA.sum)*100

 xlab<-paste("PC1","(",round(a[1],1),"%",")",sep="")

 ylab<-paste("PC2","(",round(a[2],1),"%",")",sep="")

 if(binary==TRUE){

  main<-"Sorensen PCoA"

 }else(main<-"Bray-Curtis PCoA")

 plot(Data.df.PCoA, col=Color,

     main=Title,xlab=xlab,ylab=ylab,pch=c(pch))

 return(Data.df.PCoA)

}
```

```
#for MGE, 1:27

#for ARG, 28:143

#set binary = false for bray, binary = true for Sorensen

abun<-abundance2[28:143]

b<-as.factor(abundance$class)

df.spe.bray.Sor<-Sor.bray.pcoa(abun, Dim = 2, Color = Color.Class, binary = FALSE)

ordiellipse(df.spe.bray.Sor,abundance$class,col=c("#56B4E9", "#E69F00"),lwd=2,bg=2)

#ordiellipse(df.spe.bray.Sor,groups=b,col=c(1,2),lwd=1)

title(outer=outer,adj=adj,main="C",cex.main=cex,col="black",font=2,line=line)

text(x = 0.3, y = 0.45, labels = "p = 0.01", xpd = NA,cex=2)

#text(x = -0.55, y = .43, labels = "C", xpd = NA,cex=2.5)

#legend(-0.4,0.35,c("Infancy","Pregnancy"), pch=21,col=1,pt.bg=c(1,2))

#text(df.spe.bray.Sor,labels=b,col=as.numeric(b))

#2023APR28_bray_MGE_class_All3mos_MR

#png("2023OCT08_bray_MGE_class_All3mos_MR", res=300, height=7, width=7.5,
units="in")



#permanova

PERMANOVA<-function(abun,Group,binary,iters=9999){

  Data.Dist<-vegdist(abun,method="bray", binary=binary)

  adonis2(Data.Dist~Group,permutations=iters)

}

PERMDISP<-function(abun,Group,binary,iters=9999){

  Data.Dist<-vegdist(abun,method="bray", binary=binary)

  Data.betadisper<-betadisper(Data.Dist, group=Group)
```

```
  permutest(Data.betadisper, group=Group, permutations=iters)
}



#ac<-PERMANOVA(abundance2[,c(28:143)],b,FALSE,9999)
#p-value= 1e-04 ***
#PERMDISP(abundance2[,c(28:143)],b,FALSE,9999)
#1e-04 ***
head(abun)
#Sorensen--Change title if need
abun<-presence2[28:143]
Sor.bray.pcoa<-function(abun,Dim=2,Color=1,binary,pch=16,Title="Sorensen ARG"){
  Data.df<-vegdist(abun,method="bray", binary)
  Data.df.PCoA<-cmdscale(Data.df, k = Dim, eig = FALSE)
  Data.df.PCoA.eig<-cmdscale(Data.df, k = Dim, eig = TRUE)
  eig.Data.df.PCoA<-Data.df.PCoA.eig$eig
  eig.Data.df.PCoA.sum<-sum(eig.Data.df.PCoA)
  a<-(eig.Data.df.PCoA/eig.Data.df.PCoA.sum)*100
  xlab<-paste("PC1","(",round(a[1],1),"%",")",sep="")
  ylab<-paste("PC2","(",round(a[2],1),"%",")",sep="")
  if(binary==TRUE){
    main<-"Sorensen PCoA"
  }else(main<-"Bray-Curtis PCoA")
  plot(Data.df.PCoA, col=Color,
       main=Title,xlab=xlab,ylab=ylab,pch=c(pch))
  return(Data.df.PCoA)
```

```
}
#sor
b<-as.factor(abundance$class)
df.spe.bray.Sor<-Sor.bray.pcoa(abun, Dim = 2, Color = Color.Class, binary = TRUE)
ordiellipse(df.spe.bray.Sor,abundance$class,col=c("#56B4E9", "#E69F00"),lwd=2,bg=2)
title(outer=outer,adj=adj,main="D",cex.main=cex,col="black",font=2,line=line)
text(x = 0.3, y = 0.28, labels = "p = 0.0014", xpd = NA,cex=2)
#dev.off()
#blue = infants, orange = pregnant women
#legend(-0.3,-0.25,c("Infancy","Pregnancy"), pch=21,col=1,pt.bg=c(1,2))
#text(df.spe.bray.Sor,labels=b,col=as.numeric(b))
#2023APR28_sor_MGE_class_All3mos_MR
# text(x = -0.43, y = .43, labels = "A", xpd = NA,cex=2.5)
# text(x = -0.43, y = .43, labels = "B", xpd = NA,cex=2.5)
# text(x = -0.43, y = .43, labels = "C", xpd = NA,cex=2.5)
png("2023DEC06_bray_sor_arg_mge_class_All3mos_MR.png", res=300, height=7, width=9, units="in")

plot.new()

#dev.off()
#dev.cur()

#permanova
PERMANOVA<-function(abun,Group,binary,iters=9999){
  Data.Dist<-vegdist(abun,method="bray", binary=binary)
```

```
  adonis2(Data.Dist~Group,permutations=iters)
}
PERMDISP<-function(abun,Group,binary,iters=9999){
  Data.Dist<-vegdist(abun,method="bray", binary=binary)
  Data.betadisper<-betadisper(Data.Dist, group=Group)
  permutest(Data.betadisper, group=Group, permutations=iters)
}

PERMANOVA(presence2[,c(28:143)],b,TRUE,9999)
#p-value= 1e-04 ***
PERMDISP(presence2[,c(28:143)],b,TRUE,9999)
#1e-04 ***



#aug 25 2025


# Open a TIFF device
# Open a TIFF device at 6.5 x 9 in, 600 dpi
tiff("PCoA_Figure.test.tiff", width = 6.5, height = 9, units = "in", res = 600)


# Arrange 2x2 panels with tighter margins
par(mfrow = c(2,2),
    mar = c(4,4,2,1),   # smaller top margin inside each panel
    oma = c(1,1,1,1),   # reduced outer top margin
    cex.lab=1.2,
    cex.axis=1.2,
```

```
   cex.main=1.6)


#### A: Bray–Curtis MGE ####

df.spe.bray.MGE <- Sor.bray.pcoa(abundance2[1:27], Dim=2, Color="black", binary=FALSE, pch=19, Title="Bray-Curtis MGE")

points(df.spe.bray.MGE, pch=19, col=Color.Class, cex=0.7)   # no outline

ordiellipse(df.spe.bray.MGE, abundance$class, col=c("#56B4E9","#E69F00"), lwd=2)

mtext("A", side=3, line=-1, adj=0.05, outer=TRUE, font=2, cex=1.4)

usr <- par("usr")

text(x=usr[1] + 0.05*(usr[2]-usr[1]),

    y=usr[4] - 0.08*(usr[4]-usr[3]),

    labels="p = 0.01", cex=1.2, font=2, adj=0)


#### B: Sorensen MGE ####

df.spe.sor.MGE <- Sor.bray.pcoa(presence2[1:27], Dim=2, Color="black", binary=TRUE, pch=19, Title="Sorensen MGE")

points(df.spe.sor.MGE, pch=19, col=Color.Class, cex=0.7)   # no outline

ordiellipse(df.spe.sor.MGE, abundance$class, col=c("#56B4E9","#E69F00"), lwd=2)

mtext("B", side=3, line=-1, adj=0.55, outer=TRUE, font=2, cex=1.4)

usr <- par("usr")

text(x=usr[1] + 0.05*(usr[2]-usr[1]),

    y=usr[4] - 0.08*(usr[4]-usr[3]),

    labels="p < 0.001", cex=1.2, font=2, adj=0)


#### C: Bray–Curtis ARG ####

df.spe.bray.ARG <- Sor.bray.pcoa(abundance2[28:143], Dim=2, Color="black", binary=FALSE, pch=19, Title="Bray-Curtis ARG")
```

```
points(df.spe.bray.ARG, pch=19, col=Color.Class, cex=0.7)   # no outline

ordiellipse(df.spe.bray.ARG, abundance$class, col=c("#56B4E9","#E69F00"), lwd=2)

mtext("C", side=3, line=-27, adj=0.05, outer=TRUE, font=2, cex=1.4)

usr <- par("usr")

text(x=usr[1] + 0.05*(usr[2]-usr[1]),

   y=usr[4] - 0.08*(usr[4]-usr[3]),

   labels="p = 0.01", cex=1.2, font=2, adj=0)


#### D: Sorensen ARG ####

df.spe.sor.ARG <- Sor.bray.pcoa(presence2[28:143], Dim=2, Color="black", binary=TRUE,
pch=19, Title="Sorensen ARG")

points(df.spe.sor.ARG, pch=19, col=Color.Class, cex=0.7)   # no outline

ordiellipse(df.spe.sor.ARG, abundance$class, col=c("#56B4E9","#E69F00"), lwd=2)

mtext("D", side=3, line=-27, adj=0.55, outer=TRUE, font=2, cex=1.4)

usr <- par("usr")

text(x=usr[1] + 0.05*(usr[2]-usr[1]),

   y=usr[4] - 0.08*(usr[4]-usr[3]),

   labels="p = 0.001", cex=1.2, font=2, adj=0)


# Close TIFF

dev.off()


#######Matched Pair Analysis ######


###Found an easier way to do this -- see above
#2023APR18_AMR_3mos_matchedmoms_bbys.xlsx

# abun3matchedbabies
```

```
# abundance_pair<-abundance3mos[which(abundance3mos$pair>0),] #To select pairs

# abundance_pair<-abundance_pair[-grep("121",abundance_pair$pair),]

# abundance_pair$pair<-as.numeric(abundance_pair$pair)

# is.numeric(abundance_pair$pair)

# df2<-
abundance_pair[duplicated(abundance_pair$pair)|duplicated(abundance_pair$pair,
fromLast=TRUE),]

# is.numeric(df2$pair)

# head(df2$pair)

# str(df2)

#

#

# presence_pair<-presence3mos[which(presence3mos$pair>0),] #To select pairs

# presence_pair<-presence_pair[-grep("121",presence_pair$pair),]

# presence_pair$pair<-as.numeric(presence_pair$pair)

# is.numeric(presence_pair$pair)

# df3<-presence_pair[duplicated(presence_pair$pair)|duplicated(presence_pair$pair,
fromLast=TRUE),]

# is.numeric(df3$pair)

# head(df3$pair)

# str(df3)




#2023APR18_AMR_3mos_matchedmoms_bbys.xlsx

abundance3mos.matched<-
read.xlsx("2023APR18_AMR_3mos_matchedmoms_bbys.xlsx")
```

```
presence3mos.matched<-read.xlsx("2023APR18_pres_3mos_matchedmoms_bbys.xlsx")


abundance3mos.matched$class2<-as.factor(ifelse(abundance3mos.matched$class==
'Mother','Pregnancy','Infancy'))

abundance3mos.matched$class2


presence<-presence3mos.matched

abundance<-abundance3mos.matched

#make it so just abundance data, not IDs etc

abundance2<- abundance[,4:146]

head(abundance2)


#set NAs to 0

abundance2[is.na(abundance2)]<- 0


abundance$SumMGE<-rowSums(abundance2[,1:27])

abundance$Sumamino<-rowSums(abundance2[,28:48])

abundance$SumMDR<-rowSums(abundance2[,49:69])

abundance$Sumbeta<-rowSums(abundance2[,70:85])

abundance$Sumtetra<-rowSums(abundance2[,86:98])

abundance$SumMLSB<-rowSums(abundance2[,99:116])

abundance$Sumsulfo<-rowSums(abundance2[,117:121])

abundance$Sumvanco<-rowSums(abundance2[,122:129])

abundance$Sumfluoro<-rowSums(abundance2[,130:133])

abundance$Sumother<-rowSums(abundance2[,134:143])

abundance$SumARG<-rowSums(abundance2[,28:143])
```

```
#reassign variables
presence2<- presence[,4:146]
presence2

abundance2<- abundance[,4:146]
abundance2
abundance2[is.na(abundance2)]<- 0

abundance$class<-abundance$class2
indices <- abundance[,c("class","pair")]
head(indices)

##alpha diversity analysis
indices$RichMGE<-rowSums(presence2[,1:27])
summary(indices$RichMGE)
indices$Richamino <- rowSums(presence2[,28:48])
summary(indices$Richamino)
indices$RichMDR<-rowSums(presence2[,49:69])
summary(indices$RichMDR)
indices$Richbeta<-rowSums(presence2[,70:85])
summary(indices$Richbeta)
indices$Richtetra<-rowSums(presence2[,86:98])
summary(indices$Richtetra)
indices$RichMLSB<-rowSums(presence2[,99:116])
```

```
summary(indices$RichMLSB)

indices$Richsulfo<-rowSums(presence2[,117:121])

summary(indices$Richsulfo)

indices$Richvanco<-rowSums(presence2[,122:129])

summary(indices$Richvanco)

indices$Richfluoro<-rowSums(presence2[,130:133])

summary(indices$Richfluoro)

indices$Richother<-rowSums(presence2[,134:143])

summary(indices$Richother)

indices$RichARG<-rowSums(presence2[,28:143])


dev.off()


table(indices$RichMGE)


#stacked box plot

boxplot<-c(indices$RichMGE,indices$Richamino,indices$RichMDR, indices$Richbeta, indices$Richtetra,  indices$RichMLSB, indices$Richsulfo, indices$Richvanco, indices$Richfluoro)

class<-rep(indices$class,9)

type<-rep(c("MGE","aminoglycoside","MDR","betalactamase","tetracycline","MLSB","sulfonamide","vancomycin","fluoroquinolone"),each=66)

length(boxplot)



boxplot2<-data.frame(boxplot,type,class)
```

ab<-ggplot(boxplot2, aes(x = type, y = boxplot, fill=class)) +ylim(0,30)+ geom_boxplot(width=0.4,position=position_dodge(0.4)) + theme_minimal() + theme(axis.text.x = element_text(size= 10, angle = 45)) + labs(title = "", y = "Richness", x = "") + stat_compare_means(aes(group = class),label="p.signif") + scale_fill_manual(values=c("#56B4E9", "#E69F00"))+ theme(legend.position="top",)

ab

ggsave("2023DEC04_RichnessClass_matched.tiff", units="in", width=7.5, height=5, dpi=300)

#2023APR28_RichnessClass_MatchedPairs_3mos_abxclass_MR

wilcox.test(RichARG~class, data=indices)

#p-value = 0.0008812

wilcox.test(RichMGE~class, data=indices)

# p-value = 0.1207

#Richness

b<-ggboxplot(data=indices, x="class", y="RichARG", color="black", fill="class",palette="d3", ylab="Richness", xlab="", outlier.colour = "black", outlier.shape = 1, main="Rich ARG")+ theme_classic() + guides(fill=FALSE) + coord_cartesian(ylim = c(0, 100))+ stat_compare_means(label.x=1.3, label.y=95)

b

a<-ggboxplot(data=indices, x="class", y="RichMGE", color="black", fill="class",palette="d3", ylab="Richness", xlab="", outlier.colour = "black", outlier.shape = 1, main="Rich MGE")+ theme_classic() + guides(fill=FALSE) + coord_cartesian(ylim = c(0, 40))+ stat_compare_means(label.x=1.7, label.y=35)

a

```
#combine these

figure <- ggarrange(a, b,labels = c("A", "B", ncol = 2, nrow = 1))

figure

#ggexport(figure, filename = "RichMGE_ARG_matchedpairs.tiff")



#SHANNON INDEX



#shannon diversity index as default = Relative abundance

indices$ShannonMGERA<-diversity(abundance2[,1:27])

indices$ShannonaminoRA <- diversity(abundance2[,28:48])

indices$ShannonMDRRA<-diversity(abundance2[,49:69])

indices$ShannonbetaRA<-diversity(abundance2[,70:85])

indices$ShannontetraRA<-diversity(abundance2[,86:98])

indices$ShannonMLSBRA<-diversity(abundance2[,99:116])

indices$ShannonsulfoRA<-diversity(abundance2[,117:121])

indices$ShannonvancoRA<-diversity(abundance2[,122:129])

indices$ShannonfluoroRA<-diversity(abundance2[,130:133])

indices$ShannonotherRA<-diversity(abundance2[,134:143])

indices$ShannonARGRA<-diversity(abundance2[,28:143])


##shan tests

wilcox.test(ShannonMGERA~class, data=indices)

#p-value = 0.0001238

wilcox.test(ShannonARGRA~class, data=indices)
```

```
#p-value = 0.004114

#shannon

d<-ggboxplot(data=indices, x="class", y="ShannonARGRA", color="black", fill="class",palette="d3", ylab="Shannon Diversity Index", xlab="", outlier.colour = "black", outlier.shape = 1, main="Shannon ARG")+ theme_classic() + guides(fill=FALSE) + coord_cartesian(ylim = c(0, 4.0))+ stat_compare_means(label.x=1.3, label.y=3.8)

d

c<-ggboxplot(data=indices, x="class", y="ShannonMGERA", color="black", fill="class",palette="d3", ylab="Shannon Diversity Index", xlab="", outlier.colour = "black", outlier.shape = 1, main="Shannon MGE")+ theme_classic() + guides(fill=FALSE) + coord_cartesian(ylim = c(0, 4.0))+ stat_compare_means(label.x=1.3, label.y=3.8)

c

#combine all 4

#combine all 6

#

figure <- ggarrange(b, d, f, a, c, e, labels = c("A", "B","C","D"," E", "F", ncol = 2, nrow = 3))

figure

#ggexport(figure, filename = "ShannonMGE_ARG_matchedpairs.pdf")

##2023APR28_Rich_shan_Class_MatchedPairs_3mos_MR

#stacked box plot

boxplot<-c(indices$ShannonMGERA,indices$ShannonaminoRA,indices$ShannonMDRRA, indices$ShannonbetaRA, indices$ShannontetraRA,  indices$ShannonMLSBRA, indices$ShannonsulfoRA, indices$ShannonvancoRA, indices$ShannonfluoroRA)
```

```
class<-rep(indices$class,9)

type<-
rep(c("MGE","aminoglycoside","MDR","betalactamase","tetracycline","MLSB","sulfonamide","
vancomycin","fluoroquinolone"),each=66)

length(boxplot)


boxplot2<-data.frame(boxplot,type,class)


ac<-ggplot(boxplot2, aes(x = type, y = boxplot, fill=class)) +ylim(0,4)+
geom_boxplot(width=0.4,position=position_dodge(0.4)) + theme_minimal() +
theme(axis.text.x = element_text(size = 10, angle = 45)) + labs(title = "", y = "Shannon
Diversity Index", x = "") + stat_compare_means(aes(group = class),label="p.signif") +
scale_fill_manual(values=c("#56B4E9", "#E69F00"))+ theme(legend.position="top",)

ac

ggsave("2023DEC04_Shannon_matched.tiff", units="in", width=7.5, height=5, dpi=300)

#2023APR28__shan_Class_MatchedPairs_ABXclass_3mos_MR



#stacked bar plot

sumabundance<-aggregate(boxplot~type+class, boxplot2,median)

stackplot<-ggplot(data = sumabundance, aes(x = class, y=boxplot)) +
geom_bar(stat="identity", aes(fill=type))+theme_classic() +
scale_fill_brewer(palette="Paired")+ labs(title = "", y = "Shannon Index", x = "")+theme
(axis.text = element_text(size=14), axis.title=element_text(size=14))

stackplot

#2023APR28__Stackplot_shan_Class_MatchedPairs_ABXclass_3mos_MR

#ggexport(stackplot, filename = "/Users/madeleinerussell/Desktop/Comstock
Lab/AbxResistance/ABXR_Ranalysis/ShannonDiv_ALL.pdf")
```

```
#INVERSE SIMPSON INDEX
#Inverse Simpson diversity index = Relative abundance
indices$SimpsonMGERA<-diversity(abundance2[,1:27], index="invsimpson")
indices$SimpsonaminoRA<-diversity(abundance2[,28:48], index="invsimpson")
indices$SimpsonMDRRA<-diversity(abundance2[,49:69], index="invsimpson")
indices$SimpsonbetaRA<-diversity(abundance2[,70:85], index="invsimpson")
indices$SimpsontetraRA<-diversity(abundance2[,86:98], index="invsimpson")
indices$SimpsonMLSBRA<-diversity(abundance2[,99:116], index="invsimpson")
indices$SimpsonsulfoRA<-diversity(abundance2[,117:121], index="invsimpson")
indices$SimpsonvancoRA<-diversity(abundance2[,122:129], index="invsimpson")
indices$SimpsonfluoroRA<-diversity(abundance2[,130:133], index="invsimpson")
indices$SimpsonotherRA<-diversity(abundance2[,134:143], index="invsimpson")
indices$SimpsonARGRA<-diversity(abundance2[,28:143], index="invsimpson")

#inv simp
wilcox.test(SimpsonMGERA~class, data=indices)
#p-value = 8.707e-05
wilcox.test(SimpsonARGRA~class, data=indices)
#p-value = 0.005081
#amino, beta, MGE, Tetra, vanco

class<-indices$class
SimpsonvancoRA<-indices$SimpsonvancoRA
tapply(SimpsonvancoRA, class, summary)
```

```
boxplot<-c(indices$SimpsonMGERA,indices$SimpsonaminoRA,indices$SimpsonMDRRA,
indices$SimpsonbetaRA, indices$SimpsontetraRA,  indices$SimpsonMLSBRA,
indices$SimpsonsulfoRA, indices$SimpsonvancoRA, indices$SimpsonfluoroRA)

class<-rep(indices$class,9)

type<-
rep(c("MGE","aminoglycoside","MDR","betalactamase","tetracycline","MLSB","sulfonamide","
vancomycin","fluoroquinolone"),each=66)

length(boxplot)


boxplot2<-data.frame(boxplot,type,class)


ab<-ggplot(boxplot2, aes(x = type, y = boxplot, fill=class)) +ylim(0,5)+
geom_boxplot(width=0.4,position=position_dodge(0.4)) + theme_minimal() +
theme(axis.text.x = element_text(size=10, angle = 45)) + labs(title = "", y = "Inverse Simpson
Diversity Index", x = "") + stat_compare_means(aes(group = class),label="p.signif") +
scale_fill_manual(values=c("#56B4E9", "#E69F00"))+ theme(legend.position="top",)

ab


#invsimp

e<-ggboxplot(data=indices, x="class", y="SimpsonMGERA", color="black",
fill="class",palette="d3", ylab="Inverse Simpson Diversity Index", xlab="", outlier.colour =
"black", outlier.shape = 1, main="Inverse Simpson MGE")+ theme_classic() +
guides(fill=FALSE) + coord_cartesian(ylim = c(0, 8.0))+ stat_compare_means(label.x=1.3,
label.y=7.5)

e

f<-ggboxplot(data=indices, x="class", y="SimpsonARGRA", color="black",
fill="class",palette="d3", ylab="Inverse Simpson Diversity Index", xlab="", outlier.colour =
"black", outlier.shape = 1, main="Inverse Simpson ARG")+ theme_classic() +
guides(fill=FALSE) + coord_cartesian(ylim = c(0, 21.0))+ stat_compare_means(label.x=1.3,
label.y=20.5)

f
```

```
#combine all 4

figure <- ggarrange(e, f, labels = c("E", "F", ncol = 2, nrow = 3))

figure

#2023APR28_invsimp_Class_MatchedPairs_3mos_MR

#ggexport(figure, filename = "INVsimpMGE_ARG_matchedpairs.pdf")


#combine all 6

figure <- ggarrange(b, d, f, a, c, e, labels = c("A", "B","C","D"," E", "F", ncol = 2, nrow = 3))

figure


ggsave("2023DEC_InvSimp_MGE_ARG_matched_MR.tiff", units="in", width=9, height=5,
dpi=300)


#####Beta Diversity####




presence2<- presence[,4:146]

presence2


abundance2<- abundance[,4:146]

abun<-abundance2[28:143]

head(abun)


# dev.off()

par(mfrow = c(2,2),mar=c(4,4.3,1,1)) #use this if you want to make a 4 panel figure
```

```
line = -2

cex = 2

adj  = 0.025


#for sorenson/bray curtis, if binary = true (sorenson), if binary = false (bray-curtis)

Sor.bray.pcoa<-function(abun,Dim=2,Color=1,binary,pch=16,Title="Bray-Curtis MGE"){

 Data.df<-vegdist(abun,method="bray", binary)

 Data.df.PCoA<-cmdscale(Data.df, k = Dim, eig = FALSE)

 Data.df.PCoA.eig<-cmdscale(Data.df, k = Dim, eig = TRUE)

 eig.Data.df.PCoA<-Data.df.PCoA.eig$eig

 eig.Data.df.PCoA.sum<-sum(eig.Data.df.PCoA)

 a<-(eig.Data.df.PCoA/eig.Data.df.PCoA.sum)*100

 xlab<-paste("PC1","(",round(a[1],1),"%",")",sep="")

 ylab<-paste("PC2","(",round(a[2],1),"%",")",sep="")

 if(binary==TRUE){

  main<-"Sorensen PCoA"

 }else(main<-"Bray-Curtis PCoA")

 plot(Data.df.PCoA, col=Color,

    main=Title,xlab=xlab,ylab=ylab,pch=c(pch))

 return(Data.df.PCoA)

}

Color.Class<-ifelse(grepl("Infancy", abundance$class),"#56B4E9", "#E69F00")


#bray curtis for MGE

abun<-abundance2[1:27] #select MGE

b<-as.factor(abundance$class) #for this make sure its infancy/pregnancy
```

```
df.spe.bray.Sor<-Sor.bray.pcoa(abun, Dim = 2, Color = Color.Class, binary = FALSE)
ordiellipse(df.spe.bray.Sor,abundance$class,col=c("#56B4E9", "#E69F00"),lwd=2,bg=2)
#legend(.25,-.33,c("Normal","Overweight","Obese"),
#	pch=21,col=1,pt.bg=c("#000000","#E79F00","#0072B2"))
title(outer=outer,adj=adj,main="A",cex.main=cex,col="black",font=2,line=line)
text(x = 0.35, y = 0.4, labels = "p < 0.001", xpd = NA,cex=2)
# legend(-0.3,0.3,c("Infants","Women"), pch=21,col=1,pt.bg=c(1,2))
# text(df.spe.bray.Sor,labels=b,col=as.numeric(b))


#permanova
PERMANOVA<-function(abun,Group,binary,iters=9999){
 Data.Dist<-vegdist(abun,method="bray", binary=binary)
 adonis2(Data.Dist~Group,permutations=iters)
}
PERMDISP<-function(abun,Group,binary,iters=9999){
 Data.Dist<-vegdist(abun,method="bray", binary=binary)
 Data.betadisper<-betadisper(Data.Dist, group=Group)
 permutest(Data.betadisper, group=Group, permutations=iters)
}



PERMANOVA(abundance2[,c(1:27)],b,FALSE,9999) #bray curtis, MGE
#p-value= 1e-04 ***
PERMDISP(abundance2[,c(1:27)],b,FALSE,9999) #bray curtis, MGE
#1e-04 **
```

```
#Sorensen, MGE

abun<-presence2[1:27]

Sor.bray.pcoa<-function(abun,Dim=2,Color=1,binary,pch=16,Title="Sorensen MGE"){
 Data.df<-vegdist(abun,method="bray", binary)
 Data.df.PCoA<-cmdscale(Data.df, k = Dim, eig = FALSE)
 Data.df.PCoA.eig<-cmdscale(Data.df, k = Dim, eig = TRUE)
 eig.Data.df.PCoA<-Data.df.PCoA.eig$eig
 eig.Data.df.PCoA.sum<-sum(eig.Data.df.PCoA)
 a<-(eig.Data.df.PCoA/eig.Data.df.PCoA.sum)*100
 xlab<-paste("PC1","(",round(a[1],1),"%",")",sep="")
 ylab<-paste("PC2","(",round(a[2],1),"%",")",sep="")
 if(binary==TRUE){
  main<-"Sorensen PCoA"
 }else(main<-"Bray-Curtis PCoA")
 plot(Data.df.PCoA, col=Color,
    main=Title,xlab=xlab,ylab=ylab,pch=c(pch))
 return(Data.df.PCoA)
}

b<-as.factor(abundance$class)
df.spe.bray.Sor<-Sor.bray.pcoa(abun, Dim = 2, Color = Color.Class, binary = TRUE)
#ordiellipse(df.spe.bray.Sor,groups=b,col=c(1,2),lwd=1) # another way to do ordiollipse
ordiellipse(df.spe.bray.Sor,abundance$class,col=c("#56B4E9", "#E69F00"),lwd=2,bg=2)
#text(x = -0.58, y = .35, labels = "B", xpd = NA,cex=2.5)
title(outer=outer,adj=adj,main="B",cex.main=cex,col="black",font=2,line=line)
```

```
text(x = 0.18, y = 0.35, labels = "p < 0.001", xpd = NA,cex=2)

#legend(-0.3,0.1,c("Infants","Women"), pch=21,col=1,pt.bg=c(1,2))

#text(df.spe.bray.Sor,labels=b,col=as.numeric(b))


#permanova

PERMANOVA<-function(abun,Group,binary,iters=9999){

  Data.Dist<-vegdist(abun,method="bray", binary=binary)

  adonis2(Data.Dist~Group,permutations=iters)

}

PERMDISP<-function(abun,Group,binary,iters=9999){

  Data.Dist<-vegdist(abun,method="bray", binary=binary)

  Data.betadisper<-betadisper(Data.Dist, group=Group)

  permutest(Data.betadisper, group=Group, permutations=iters)

}


#MGE sorenson

PERMANOVA(presence2[,c(1:27)],b,TRUE,9999)

#1e-04 ***

PERMDISP(presence2[,c(1:27)],b,TRUE,9999)

#0.7968


###ARG-- need to select correct columns

presence2<- presence[,4:146]

presence2


abundance2<- abundance[,4:146]
```

```
abun<-abundance2[28:143]

head(abun)


#bray curtis

Sor.bray.pcoa<-function(abun,Dim=2,Color=1,binary,pch=16,Title="Bray-Curtis ARG"){

 Data.df<-vegdist(abun,method="bray", binary)

 Data.df.PCoA<-cmdscale(Data.df, k = Dim, eig = FALSE)

 Data.df.PCoA.eig<-cmdscale(Data.df, k = Dim, eig = TRUE)

 eig.Data.df.PCoA<-Data.df.PCoA.eig$eig

 eig.Data.df.PCoA.sum<-sum(eig.Data.df.PCoA)

 a<-(eig.Data.df.PCoA/eig.Data.df.PCoA.sum)*100

 xlab<-paste("PC1","(",round(a[1],1),"%",")",sep="")

 ylab<-paste("PC2","(",round(a[2],1),"%",")",sep="")

 if(binary==TRUE){

  main<-"Sorensen PCoA"

 }else(main<-"Bray-Curtis PCoA")

 plot(Data.df.PCoA, col=Color,

     main=Title,xlab=xlab,ylab=ylab,pch=c(pch))

 return(Data.df.PCoA)

}


abun<-abundance2[28:143] #select ARG data

b<-as.factor(abundance$class)

abundance$class

df.spe.bray.Sor<-Sor.bray.pcoa(abun, Dim = 2, Color = Color.Class, binary = FALSE)

#ordiellipse(df.spe.bray.Sor,groups=b,col=c(1,2),lwd=1)
```

```
ordiellipse(df.spe.bray.Sor,abundance$class,col=c("#56B4E9", "#E69F00"),lwd=2,bg=2)
title(outer=outer,adj=adj,main="C",cex.main=cex,col="black",font=2,line=line)
text(x = 0.5, y = 0.33, labels = "p < 0.001", xpd = NA,cex=2)

#text(x = -0.325, y = 0.32, labels = "C", xpd = NA,cex=2.5)
#legend(-0.3,-0.3,c("Infants","Women"), pch=21,col=1,pt.bg=c(1,2))
#text(df.spe.bray.Sor,labels=b,col=as.numeric(b))

#permanova
PERMANOVA<-function(abun,Group,binary,iters=9999){
  Data.Dist<-vegdist(abun,method="bray", binary=binary)
  adonis2(Data.Dist~Group,permutations=iters)
}
PERMDISP<-function(abun,Group,binary,iters=9999){
  Data.Dist<-vegdist(abun,method="bray", binary=binary)
  Data.betadisper<-betadisper(Data.Dist, group=Group)
  permutest(Data.betadisper, group=Group, permutations=iters)
}

#bray curtis, ARG
PERMANOVA(abundance2[,c(28:143)],b,FALSE,9999)
#p-value= 1e-04 ***
PERMDISP(abundance2[,c(28:143)],b,FALSE,9999)
#1e-04 ***

#Sorensen, ARG
```

```
abun<-presence2[28:143]

Sor.bray.pcoa<-function(abun,Dim=2,Color=1,binary,pch=16,Title="Sorensen ARG"){

  Data.df<-vegdist(abun,method="bray", binary)

  Data.df.PCoA<-cmdscale(Data.df, k = Dim, eig = FALSE)

  Data.df.PCoA.eig<-cmdscale(Data.df, k = Dim, eig = TRUE)

  eig.Data.df.PCoA<-Data.df.PCoA.eig$eig

  eig.Data.df.PCoA.sum<-sum(eig.Data.df.PCoA)

  a<-(eig.Data.df.PCoA/eig.Data.df.PCoA.sum)*100

  xlab<-paste("PC1","(",round(a[1],1),"%",")",sep="")

  ylab<-paste("PC2","(",round(a[2],1),"%",")",sep="")

  if(binary==TRUE){

    main<-"Sorensen PCoA"

  }else(main<-"Bray-Curtis PCoA")

  plot(Data.df.PCoA, col=Color,

       main=Title,xlab=xlab,ylab=ylab,pch=c(pch))

  return(Data.df.PCoA)

}

#sor

b<-as.factor(abundance$class)

df.spe.bray.Sor<-Sor.bray.pcoa(abun, Dim = 2, Color = Color.Class, binary = TRUE)

#ordiellipse(df.spe.bray.Sor,groups=b,col=c(1,2),lwd=1)

ordiellipse(df.spe.bray.Sor,abundance$class,col=c("#56B4E9", "#E69F00"),lwd=2,bg=2)

title(outer=outer,adj=adj,main="D",cex.main=cex,col="black",font=2,line=line)

text(x = 0.25, y = 0.2, labels = "p < 0.001", xpd = NA,cex=2)


#text(x = -0.35, y = .2, labels = "D", xpd = NA,cex=2.5)
```

```
#legend(-0.3,0.1,c("Infants","Women"), pch=21,col=1,pt.bg=c(1,2))

#text(df.spe.bray.Sor,labels=b,col=as.numeric(b))


#permanova

PERMANOVA<-function(abun,Group,binary,iters=9999){

  Data.Dist<-vegdist(abun,method="bray", binary=binary)

  adonis2(Data.Dist~Group,permutations=iters)

}

PERMDISP<-function(abun,Group,binary,iters=9999){

  Data.Dist<-vegdist(abun,method="bray", binary=binary)

  Data.betadisper<-betadisper(Data.Dist, group=Group)

  permutest(Data.betadisper, group=Group, permutations=iters)

}


#PERMANOVA(presence2[,c(28:143)],b,TRUE,9999)

#0.0014***

#PERMDISP(presence2[,c(28:143)],b,TRUE,9999)

#1e-04***


#png("2023DEC04_bray_sor_arg_mge_class_matched_MR.tiff", res=300, height=7,
width=9, units="in")

# how to save it


#now, go back and do it with making 4 graphs on one plot


plot.new()
```

```
#####Density graph using Beta diversity####


#need to do it with only matched data
abundance3mos.matched<-
read.xlsx("2023APR18_AMR_3mos_matchedmoms_bbys.xlsx")
presence3mos.matched<-read.xlsx("2023APR18_pres_3mos_matchedmoms_bbys.xlsx")
abundance<-abundance3mos.matched
presence<-presence3mos.matched
presence2<-presence[,4:146]

if (!require("BiocManager", quietly = TRUE))
 install.packages("BiocManager")

#BiocManager::install("phyloseq")
library("phyloseq")
lmp = function(y, x, n.perms = 9999) {
 test = numeric(n.perms)
 test[1] = abs(summary(lm(y ~ x))$coefficients[2, 3])
 for (ii in 2:n.perms) {
  test[ii] = abs(summary(lm(sample(y) ~ x))$coefficients[2, 3])
 }
 p.value = sum(test >= test[1])/n.perms
 return(p.value)
}
```

```
# abundance_pair<-abundance3mos[which(abundance3mos$pair>0),] #To select pairs

# abundance_pair<-abundance_pair[-grep("121",abundance_pair$pair),]

# abundance_pair$pair<-as.numeric(abundance_pair$pair)

# is.numeric(abundance_pair$pair)

# df2<-
abundance_pair[duplicated(abundance_pair$pair)|duplicated(abundance_pair$pair,
fromLast=TRUE),]

# is.numeric(df2$pair)

# head(df2$pair)

# str(df2)

df2<-abundance


#MGE= 1:27, ARG = 28:143

abundance2<-abundance[,4:146]

amrdata_RA<-abundance2[28:143]

amrdata_RA[is.na(amrdata_RA)]<- 0


dissimilarity<-vegdist(amrdata_RA, upper=TRUE, diag=TRUE, method="bray")
#diagonal=dissimilarities within pairs

view(dissimilarity)

distance<-as.vector(dissimilarity)

str(distance)


#Beta diversity matrix by family status

##look for other sources off of NCBI/SRA/github

#the sample size is what makes up the matrix size

x = matrix(NA, 66, 66)
```

```
Fvec <- sample_data(df2)$pair

Fvec

for (n in 1:(ncol(x) - 1)) {

 for (m in (n + 1):ncol(x)) {

  f <- eval(Fvec[n] == Fvec[m])

  x[n, m] = f

 }

}

x <- t(x)

family_vec <- x[lower.tri(x, diag = F)]

#bouellian=T/F



#Beta diversity matrix by type of sample

x = matrix(NA, 66, 66)

Yvec <- sample_data(df2)$class

for (n in 1:(ncol(x) - 1)) {

 for (m in (n + 1):ncol(x)) {

  f <- eval(Yvec[n] == Yvec[m])

  x[n, m] = f

 }

}

x <- t(x)

class_vec <- x[lower.tri(x, diag = F)]


#Creating dataframes
```

```
zz <- data.frame(distance, family_vec,class_vec)

zz$comb <- paste(zz$family_vec, zz$class_vec, sep = "-")


# Remove the FALSE-TRUE, or different family same type


#ARG

#family

#ARG Bray= 0.8829883

#type and family

#AMR Bray: 0.01870187

#type

#AMR Bray: 0.00010001


#MGE

#family

#MGE Bray= 0.5392539

#type and family

#MGE Bray:  0.3051305

#MGE Bray: 0.00010001


#family

#Comparing Different mother-infants pairs between different family and same family

#Indicates that infants feces are more similar to mothers feces than to unrelated mother feces.

#COMPARING TRUE-FALSE TO FALSE FALSE (same family, diff type to diff family diff type )

zz_temp1 <- zz[grep("FALSE-TRUE", zz$comb, invert = TRUE), ] # Remove the FALSE-TRUE, or different family same type
```

```
lmp(zz_temp1$distance, zz_temp1$comb)

#ARG Bray= 0.8829883

#MGE Bray= 0.5392539

boxplot(zz_temp1$distance ~ zz_temp1$comb, main="Bray dissimilarity index of ARG patterns")


library(ggpubr)

zz_temp2<-zz_temp1


zz_temp2$combinations<-as.factor(ifelse(zz_temp2$comb== 'FALSE-FALSE', 'Different Family - Different Type',

                                ifelse(zz_temp2$comb== 'TRUE-FALSE', 'Same Family - Different Type', 'other')))



# zz_temp2$comb<-levels(zz_temp2$comb)[levels(zz_temp2$comb)=="FALSE-FALSE"] <- "Different Family - Different Type"

# zz_temp2$comb<-levels(zz_temp2$comb)[levels(zz_temp2$comb)=="TRUE-FALSE"] <- "Same Family - Different Type"




brayARG_box<-ggboxplot(zz_temp2, "combinations", "distance",

            color = "black", fill = "gray",

            title = "Between-Sample Bray Dissimilarity ARG - Family",

            xlab = "Comparisons",

            ylab = "Between-Sample Bray Dissimilarity")
```

```
brayARG_box2<-brayARG_box + annotate("text", x = 1.5, y = 1,  label = "p = 0.883")

brayARG_box2



#type and family

#indicates that infants are more similar to each other than to their own mothers

#COMPARING FALSE-TRUE TO TRUE-FALSE (diff family, same type to same family diff type )

zz_temp3 <- zz[grep("FALSE-FALSE", zz$comb, invert = TRUE), ]

lmp(zz_temp3$distance, zz_temp3$comb)

#AMR Bray: 0.01870187

#MGE Bray:  0.3051305

boxplot(zz_temp3$distance ~ zz_temp3$comb, main="Bray dissimilarity index of ARG patterns")

zz_temp3$combinations<-as.factor(ifelse(zz_temp3$comb== 'FALSE-TRUE', 'Different Family - Same Type',

                                ifelse(zz_temp3$comb== 'TRUE-FALSE', 'Same Family - Different Type', 'other')))



brayARG_box3<-ggboxplot(zz_temp3, "combinations", "distance",

                  color = "black", fill = "gray",

                  title = "Between-Sample Bray Dissimilarity ARG - Type & Family",

                  xlab = "Comparisons",

                  ylab = "Between-Sample Bray Dissimilarity")

brayARG_box4<-brayARG_box3 + annotate("text", x = 1.5, y = 1,  label = "p = 0.0187")

brayARG_box4
```

```
#type

#indicates mothers and infants are significantly different from each other

zz_temp4 <- zz[grep("TRUE-FALSE", zz$comb, invert = TRUE), ]

lmp(zz_temp4$distance, zz_temp4$comb)

#AMR Bray: 0.00010001

#MGE Bray: 0.00010001


boxplot(zz_temp4$distance ~ zz_temp4$comb, main="Bray dissimilarity index of ARG patterns")

zz_temp4$combinations<-as.factor(ifelse(zz_temp4$comb== 'FALSE-TRUE', 'Different Family - Same Type',

                              ifelse(zz_temp4$comb== 'FALSE-FALSE', 'Different Family - Different Type', 'other')))



brayARG_box5<-ggboxplot(zz_temp4, "combinations", "distance",

            color = "black", fill = "gray",

            title = "Between-Sample Bray Dissimilarity ARG - Type",

            xlab = "Comparisons",

            ylab = "Between-Sample Bray Dissimilarity")

brayARG_box6<-brayARG_box5 + annotate("text", x = 1.5, y = 1,  label = "p = 0.0001")

brayARG_box6


#boxplot(zz_temp$distance ~ zz_temp$comb, main="Horn- Morisita dissimilarity index of AMG patterns")

#To get boxplot

#dev.off()
```

#Density plot using RA and Bray Curtis Dissimilarity

#2023APR28_ARG_dissim_plot_bray_MR

#smoothed density estimate (geom density)

BrayRA_ARG<- ggplot(zz, aes(x = distance, fill = factor(comb))) + geom_density(alpha = 0.5) + theme_minimal()+

theme(legend.justification = c(0.2,4), legend.position = c(0.05, 1), legend.text = element_text(size = rel(0.6))) +

scale_fill_manual(values = c("brown1", "dodgerblue ", "gold", "green"), labels = c("Different family, mother and infant","Different family, same type", "Same family, mother and infant"), "Comparisons") +

xlab("Between-sample Bray-Curtis dissimilarity ") + theme(axis.title.x = element_text(size=7)) + ggtitle("ARG") +

ylim(0, 5)

BrayRA_ARG2<-BrayRA_ARG + theme(legend.position="top")

BrayRA_ARG2

figure <- ggarrange(BrayRA_ARG2, brayARG_box2, brayARG_box6, brayARG_box4, labels = c("A", "B", "C", "D", ncol = 2, nrow = 2), common.legend = TRUE, legend="top")

figure

#PRESENCE/ABSENCE

presence_pair<-presence3mos[which(presence3mos$pair>0),]

presence_pair<-presence_pair[-grep("121",presence_pair$pair),]

presence_pair$pair<-as.numeric(presence_pair$pair)

```
# df3<-presence_pair[duplicated(presence_pair$pair)|duplicated(presence_pair$pair, fromLast=TRUE),]

# is.numeric(df3$pair)

# head(df3$pair)

# str(df3)




df3<-presence

df3$pair.y

#MGE: [,4:30], ARG:[,31:146]

amrdata<- presence2[,28:143]

# comparing RA mother and their own children

dissimilarity<-vegdist(amrdata, upper=TRUE, diag=TRUE, method="bray")
#diagonal=dissimilarities within pairs

distance<-as.vector(dissimilarity)

str(distance)


#Matrix for pair

x = matrix(NA, 66, 66)

Fvec <- sample_data(df3)$pair.y

for (n in 1:(ncol(x) - 1)) {

 for (m in (n + 1):ncol(x)) {

  f <- eval(Fvec[n] == Fvec[m])

  x[n, m] = f

 }

}
```

```
x <- t(x)
family_vec <- x[lower.tri(x, diag = F)]

#Matrix for class
x = matrix(NA, 66, 66)
Yvec <- sample_data(df3)$class.y
for (n in 1:(ncol(x) - 1)) {
  for (m in (n + 1):ncol(x)) {
    f <- eval(Yvec[n] == Yvec[m])
    x[n, m] = f
  }
}
x <- t(x)
class_vec <- x[lower.tri(x, diag = F)]

#Creating dataframes
zz <- data.frame(distance, family_vec,class_vec)
zz$comb <- paste(zz$family_vec, zz$class_vec, sep = "-")

#family
#ARG Sor: 0.08790879
#MGE Sor: 0.2254225

#type and family
#ARG Sor:0.00020002
#MGE Sor: 0.8391839
```

```
#type

#ARG Sor:0.00010001

#MGE Sor: 0.00020002



# Remove the FALSE-TRUE, or different family same type

zz_temp5 <- zz[grep("FALSE-TRUE", zz$comb, invert = TRUE), ]

lmp(zz_temp5$distance, zz_temp5$comb)

#ARG Sor: 0.08790879

#MGE Sor: 0.2254225

zz_temp5$combinations<-as.factor(ifelse(zz_temp5$comb== 'FALSE-FALSE', 'Different Family - Different Type',

                              ifelse(zz_temp5$comb== 'TRUE-FALSE', 'Same Family - Different Type', 'other')))



boxplot(zz_temp5$distance ~ zz_temp5$comb, main="Sorensen dissimilarity index of ARG patterns")

sorARG_box<-ggboxplot(zz_temp5, "combinations", "distance",

           color = "black", fill = "gray",

           title = "Between-Sample Sorensen Dissimilarity ARG - Family",

           xlab = "Comparisons",

           ylab = "Between-Sample Sorensen Dissim.")

sorARG_box2<-sorARG_box + annotate("text", x = 1.5, y = 1,  label = "p = 0.088")

sorARG_box2
```

```
#type and family

zz_temp6 <- zz[grep("FALSE-FALSE", zz$comb, invert = TRUE), ]

lmp(zz_temp6$distance, zz_temp6$comb)

zz_temp6$combinations<-as.factor(ifelse(zz_temp6$comb== 'FALSE-TRUE', 'Different Family - Same Type',
                    ifelse(zz_temp6$comb== 'TRUE-FALSE', 'Same Family - Different Type', 'other')))


#ARG Sor:0.00020002

#MGE Sor: 0.8391839


sorARG_box3<-ggboxplot(zz_temp6, "combinations", "distance",
            color = "black", fill = "gray",
            title = "Between-Sample Sorensen Dissimilarity ARG - Type & Family",
            xlab = "Comparisons",
            ylab = "Between-Sample Sorensen Dissim.")

sorARG_box4<-sorARG_box3 + annotate("text", x = 1.5, y = 1,  label = "p = 0.0002")

sorARG_box4



#type

zz_temp7 <- zz[grep("TRUE-FALSE", zz$comb, invert = TRUE), ]

lmp(zz_temp7$distance, zz_temp7$comb)

#ARG Sor:0.00010001

#MGE Sor: 0.00020002
```

```
boxplot(zz_temp7$distance ~ zz_temp7$comb, main="Sorensen dissimilarity index of AMG patterns")

zz_temp7$combinations<-as.factor(ifelse(zz_temp7$comb== 'FALSE-TRUE', 'Different Family - Same Type',

                    ifelse(zz_temp7$comb== 'FALSE-FALSE', 'Different Family - Different Type', 'other')))


sorARG_box5<-ggboxplot(zz_temp7, "combinations", "distance",

            color = "black", fill = "gray",

            title = "Between-Sample Sorensen Dissimilarity ARG - Type",

            xlab = "Comparisons",

            ylab = "Between-Sample Sorensen Dissim.")

sorARG_box6<-sorARG_box5 + annotate("text", x = 1.5, y = 1,  label = "p = 0.0001")

sorARG_box6



SorRA_ARG<- ggplot(zz, aes(x = distance, fill = factor(comb))) + geom_density(alpha = 0.5) +  theme_minimal()+

  theme(legend.justification = c(0.05, 1), legend.position = c(0.05, 1), legend.text = element_text(size = rel(0.6))) +

  scale_fill_manual(values = c("brown1", "dodgerblue ", "gold", "green"), labels = c("Different family, mother and infant","Different family, same type", "Same family, mother and infant"), "Comparisons") +

  xlab("Between-sample Sorensen dissimilarity ") + theme(axis.title.x = element_text(size=7)) + ggtitle("ARG") +

  ylim(0, 5)
```

```
SorRA_ARG

figure2 <- ggarrange(SorRA_ARG, sorARG_box2, sorARG_box6, sorARG_box4, labels = c("A", "B", "C", "D", ncol = 2, nrow = 2), common.legend = TRUE, legend="top")

figure2

#MGE

#MGE= 1:27, ARG = 28:143

abundance2<-abundance[,4:146]

amrdata_RA<-abundance2[1:27]

amrdata_RA[is.na(amrdata_RA)]<- 0

dissimilarity<-vegdist(amrdata_RA, upper=TRUE, diag=TRUE, method="bray") #diagonal=dissimilarities within pairs

view(dissimilarity)

distance<-as.vector(dissimilarity)

str(distance)

#Beta diversity matrix by family status

##look for other sources off of NCBI/SRA/github

#the sample size is what makes up the matrix size

x = matrix(NA, 66, 66)

Fvec <- sample_data(df2)$pair

Fvec

for (n in 1:(ncol(x) - 1)) {

  for (m in (n + 1):ncol(x)) {
```

```
    f <- eval(Fvec[n] == Fvec[m])
    x[n, m] = f
  }
}
x <- t(x)
family_vec <- x[lower.tri(x, diag = F)]
#bouellian=T/F


#Beta diversity matrix by type of sample
x = matrix(NA, 66, 66)
Yvec <- sample_data(df2)$class
for (n in 1:(ncol(x) - 1)) {
  for (m in (n + 1):ncol(x)) {
    f <- eval(Yvec[n] == Yvec[m])
    x[n, m] = f
  }
}
x <- t(x)
class_vec <- x[lower.tri(x, diag = F)]

#Creating dataframes
zz <- data.frame(distance, family_vec,class_vec)
zz$comb <- paste(zz$family_vec, zz$class_vec, sep = "-")

# Remove the FALSE-TRUE, or different family same type
```

#ARG

#family

#ARG Bray= 0.8829883

#type and family

#AMR Bray: 0.01870187

#type

#AMR Bray: 0.00010001

#MGE

#family

#MGE Bray= 0.5392539

#type and family

#MGE Bray: 0.3051305

#MGE Bray: 0.00010001

#family

#Comparing Different mother-infants pairs between different family and same family

#Indicates that infants feces are more similar to mothers feces than to unrelated mother feces.

#COMPARING TRUE-FALSE TO FALSE FALSE (same family, diff type to diff family diff type)

zz_temp8 <- zz[grep("FALSE-TRUE", zz$comb, invert = TRUE),] # Remove the FALSE-TRUE, or different family same type

lmp(zz_temp8$distance, zz_temp8$comb)

#ARG Bray= 0.8829883

#MGE Bray= 0.5392539

```
boxplot(zz_temp8$distance ~ zz_temp8$comb, main="Bray dissimilarity index of MGE patterns")

zz_temp8$combinations<-as.factor(ifelse(zz_temp8$comb== 'FALSE-FALSE', 'Different Family - Different Type',
                    ifelse(zz_temp8$comb== 'TRUE-FALSE', 'Same Family - Different Type', 'other')))


library(ggpubr)

brayMGE_box<-ggboxplot(zz_temp8, "combinations", "distance",
          color = "black", fill = "gray",
          title = "Between-Sample Bray Dissimilarity MGE - Family",
          xlab = "Comparisons",
          ylab = "Between-Sample Bray Dissimilarity")

brayMGE_box2<-brayMGE_box + annotate("text", x = 1.5, y = 1,  label = "p = 0.539")

brayMGE_box2



#type and family

#indicates that infants are more similar to each other than to their own mothers

#COMPARING FALSE-TRUE TO TRUE-FALSE (diff family, same type to same family diff type )

zz_temp9 <- zz[grep("FALSE-FALSE", zz$comb, invert = TRUE), ]

lmp(zz_temp9$distance, zz_temp9$comb)


zz_temp9$combinations<-as.factor(ifelse(zz_temp9$comb== 'FALSE-TRUE', 'Different Family - Same Type',
                    ifelse(zz_temp9$comb== 'TRUE-FALSE', 'Same Family - Different Type', 'other')))
```

```
#AMR Bray: 0.01870187

#MGE Bray:  0.3051305

boxplot(zz_temp9$distance ~ zz_temp9$comb, main="Bray dissimilarity index of MGE patterns")

brayMGE_box3<-ggboxplot(zz_temp9, "combinations", "distance",
          color = "black", fill = "gray",
          title = "Between-Sample Bray Dissimilarity MGE - Type & Family",
          xlab = "Comparisons",
          ylab = "Between-Sample Bray Dissimilarity")

brayMGE_box4<-brayMGE_box3 + annotate("text", x = 1.5, y = 1,  label = "p = 0.305")

brayMGE_box4


#type

#indicates mothers and infants are significantly different from each other

zz_temp10 <- zz[grep("TRUE-FALSE", zz$comb, invert = TRUE), ]

lmp(zz_temp10$distance, zz_temp10$comb)

zz_temp10$combinations<-as.factor(ifelse(zz_temp10$comb== 'FALSE-TRUE', 'Different Family - Same Type',
                  ifelse(zz_temp10$comb== 'FALSE-FALSE', 'Different Family - Different Type', 'other')))

#AMR Bray: 0.00010001

#MGE Bray: 0.00010001

boxplot(zz_temp10$distance ~ zz_temp10$combinations, main="Bray dissimilarity index of MGE patterns")

brayMGE_box5<-ggboxplot(zz_temp10, "combinations", "distance",
          color = "black", fill = "gray",
```

```
                title = "Between-Sample Bray Dissimilarity MGE - Type",

                xlab = "Comparisons",

                ylab = "Between-Sample Bray Dissimilarity")

brayMGE_box6<-brayMGE_box5 + annotate("text", x = 1.5, y = 1,  label = "p = 0.0001")

brayMGE_box6


#boxplot(zz_temp$distance ~ zz_temp$comb, main="Horn- Morisita dissimilarity index of AMG patterns")

#To get boxplot

#dev.off()

#Density plot using RA and Bray Curtis Dissimilarity



#2023APR28_ARG_dissim_plot_bray_MR


#smoothed density estimate (geom density )

BrayRA_MGE<- ggplot(zz, aes(x = distance, fill = factor(comb))) + geom_density(alpha = 0.5) +  theme_minimal()+

  theme(legend.justification = c(0.2,4), legend.position = c(0.05, 1), legend.text = element_text(size = rel(0.6))) +

  scale_fill_manual(values = c("brown1", "dodgerblue ", "gold", "green"), labels = c("Different family, mother and infant", "Different family, same type", "Same family, mother and infant"), "Comparisons") +

  xlab("Between-sample Bray-Curtis dissimilarity ") + theme(axis.title.x = element_text(size=7)) + ggtitle("MGE") +

  ylim(0, 5)

BrayRA_MGE2<-BrayRA_MGE + theme(legend.position="top")

BrayRA_MGE2
```

```
figure3 <- ggarrange(BrayRA_MGE2, brayMGE_box2, brayMGE_box6, brayMGE_box4, labels
= c("A", "B", "C", "D", ncol = 2, nrow = 2), common.legend = TRUE, legend="top")

figure3


#PRESENCE/ABSENCE


# presence_pair<-presence3mos[which(presence3mos$pair>0),]

# presence_pair<-presence_pair[-grep("121",presence_pair$pair),]

# presence_pair$pair<-as.numeric(presence_pair$pair)

# df3<-presence_pair[duplicated(presence_pair$pair)|duplicated(presence_pair$pair,
fromLast=TRUE),]

# is.numeric(df3$pair)

# head(df3$pair)

# str(df3)


#pres--MGE


df3<-presence

df3$pair.y

#MGE: [,4:30], ARG:[,31:146]

amrdata<- presence2[,1:27]

# comparing RA mother and their own children

dissimilarity<-vegdist(amrdata, upper=TRUE, diag=TRUE, method="bray")
#diagonal=dissimilarities within pairs

distance<-as.vector(dissimilarity)

str(distance)
```

```
#Matrix for pair
x = matrix(NA, 66, 66)
Fvec <- sample_data(df3)$pair.y
for (n in 1:(ncol(x) - 1)) {
 for (m in (n + 1):ncol(x)) {
  f <- eval(Fvec[n] == Fvec[m])
  x[n, m] = f
 }
}
x <- t(x)
family_vec <- x[lower.tri(x, diag = F)]

#Matrix for class
x = matrix(NA, 66, 66)
Yvec <- sample_data(df3)$class.y
for (n in 1:(ncol(x) - 1)) {
 for (m in (n + 1):ncol(x)) {
  f <- eval(Yvec[n] == Yvec[m])
  x[n, m] = f
 }
}
x <- t(x)
class_vec <- x[lower.tri(x, diag = F)]

#Creating dataframes
zz <- data.frame(distance, family_vec,class_vec)
```

```
zz$comb <- paste(zz$family_vec, zz$class_vec, sep = "-")

#family
#ARG Sor: 0.08790879
#MGE Sor: 0.2254225

#type and family
#ARG Sor:0.00020002
#MGE Sor: 0.8391839

#type
#ARG Sor:0.00010001
#MGE Sor: 0.00020002



# Remove the FALSE-TRUE, or different family same type
zz_temp11 <- zz[grep("FALSE-TRUE", zz$comb, invert = TRUE), ]
lmp(zz_temp11$distance, zz_temp11$comb)
#ARG Sor: 0.08790879
#MGE Sor: 0.2254225
zz_temp11$combinations<-as.factor(ifelse(zz_temp11$comb== 'FALSE-FALSE', 'Different Family - Different Type',
                          ifelse(zz_temp11$comb== 'TRUE-FALSE', 'Same Family - Different Type', 'other')))
```

```
boxplot(zz_temp11$distance ~ zz_temp11$comb, main="Sorensen dissimilarity index of
MGE patterns")

sorMGE_box<-ggboxplot(zz_temp11, "combinations", "distance",

                color = "black", fill = "gray",

                title = "Between-Sample Sorensen Dissimilarity MGE - Family",

                xlab = "Comparisons",

                ylab = "Between-Sample Sorensen Dissim.")

sorMGE_box2<-sorMGE_box + annotate("text", x = 1.5, y = 1,  label = "p = 0.225")

sorMGE_box2



#type and family

zz_temp12 <- zz[grep("FALSE-FALSE", zz$comb, invert = TRUE), ]

lmp(zz_temp12$distance, zz_temp12$comb)

zz_temp12$combinations<-as.factor(ifelse(zz_temp12$comb== 'FALSE-TRUE', 'Different
Family - Same Type',

                            ifelse(zz_temp12$comb== 'TRUE-FALSE', 'Same Family - Different Type',
'other')))

#AMR Bray: 0.00010001

#ARG Sor:0.00020002

#MGE Sor: 0.8391839


sorMGE_box3<-ggboxplot(zz_temp12, "combinations", "distance",

                color = "black", fill = "gray",

                title = "Between-Sample Sorensen Dissimilarity MGE - Type & Family",

                xlab = "Comparisons",

                ylab = "Between-Sample Sorensen Dissim.")
```

```
sorMGE_box4<-sorMGE_box3 + annotate("text", x = 1.5, y = 1,  label = "p = 0.839")

sorMGE_box4



#type

zz_temp13 <- zz[grep("TRUE-FALSE", zz$comb, invert = TRUE), ]

lmp(zz_temp13$distance, zz_temp13$comb)


zz_temp13$combinations<-as.factor(ifelse(zz_temp13$comb== 'FALSE-TRUE', 'Different Family - Same Type',

                             ifelse(zz_temp13$comb== 'FALSE-FALSE', 'Different Family - Different Type', 'other')))

#ARG Sor:0.00010001

#MGE Sor: 0.00020002


boxplot(zz_temp13$distance ~ zz_temp13$comb, main="Sorensen dissimilarity index of AMG patterns")


sorMGE_box5<-ggboxplot(zz_temp13, "combinations", "distance",

              color = "black", fill = "gray",

              title = "Between-Sample Sorensen Dissimilarity MGE - Type",

              xlab = "Comparisons",

              ylab = "Between-Sample Sorensen Dissim.")

sorMGE_box6<-sorMGE_box5 + annotate("text", x = 1.5, y = 1,  label = "p = 0.0002")

sorMGE_box6
```

```
SorRA_MGE<- ggplot(zz, aes(x = distance, fill = factor(comb))) + geom_density(alpha = 0.5)
+  theme_minimal()+

  theme(legend.justification = c(0.05, 1), legend.position = c(0.05, 1), legend.text =
element_text(size = rel(0.6))) +

  scale_fill_manual(values = c("brown1", "dodgerblue ", "gold", "green"), labels = c("Different
family, mother and infant","Different family, same type", "Same family, mother and infant"),
"Comparisons") +

  xlab("Between-sample Sorensen dissimilarity ") + theme(axis.title.x =
element_text(size=7)) + ggtitle("MGE") +

  ylim(0, 5)

SorRA_MGE


figure4 <- ggarrange(SorRA_MGE, sorMGE_box2, sorMGE_box6, sorMGE_box4, labels =
c("A", "B", "C", "D", ncol = 2, nrow = 2), common.legend = TRUE, legend="top")

figure4


figure

figure2

figure3

figure4



#complete plot

amrdata_RAARG<- df2[,31:146] #[,31:146] for ARG   [,4:30] for MGE

amrdata_RAARG [is.na(amrdata_RAARG)]<- 0
```

```
dissimilarityRAARG<-vegdist(amrdata_RAARG, upper=TRUE, diag=TRUE, method="bray")
#diagonal=dissimilarities within pairs

distanceRAARG<-as.vector(dissimilarityRAARG)

str(distanceRAARG)

zzRAARG <- data.frame(distanceRAARG, family_vec,class_vec)

zzRAARG$comb <- paste(zz$family_vec, zz$class_vec, sep = "-")


amrdata_RAMGE<- df2[,4:30] #[,31:146] for ARG   [,4:30] for MGE

amrdata_RAMGE [is.na(amrdata_RAMGE)]<- 0

dissimilarityRAMGE<-vegdist(amrdata_RAMGE, upper=TRUE, diag=TRUE, method="bray")
#diagonal=dissimilarities within pairs

distanceRAMGE<-as.vector(dissimilarityRAMGE)

zzRAMGE <- data.frame(distanceRAMGE, family_vec,class_vec)

zzRAMGE$comb <- paste(zz$family_vec, zz$class_vec, sep = "-")


amrdata_BIARG<- df3[,31:146] #[,31:146] for ARG   [,4:30] for MGE

dissimilarityBIARG<-vegdist(amrdata_BIARG, upper=TRUE, diag=TRUE, method="horn")
#diagonal=dissimilarities within pairs

distanceBIARG<-as.vector(dissimilarityBIARG)

str(distanceBIARG)

zzBIARG <- data.frame(distanceBIARG, family_vec,class_vec)

zzBIARG$comb <- paste(zz$family_vec, zz$class_vec, sep = "-")


amrdataBIMGE<- df3[,4:30]

dissimilarityBIMGE<-vegdist(amrdataBIMGE, upper=TRUE, diag=TRUE, method="horn")
#diagonal=dissimilarities within pairs

distanceBIMGE<-as.vector(dissimilarityBIMGE)
```

```
zzBIMGE <- data.frame(distanceBIMGE, family_vec,class_vec)

zzBIMGE$comb <- paste(zz$family_vec, zz$class_vec, sep = "-")



#Complete plot


#ARG

#family

#ARG Bray= 0.8829883

#type and family

#AMR Bray: 0.01870187

#type

#AMR Bray: 0.00010001


#MGE

#family

#MGE Bray= 0.5392539

#type and family

#MGE Bray:  0.3051305

#MGE Bray: 0.00010001


##Sor

#family

#ARG Sor: 0.08790879

#MGE Sor: 0.2254225
```

#type and family

#ARG Sor:0.00020002

#MGE Sor: 0.8391839

#type

#ARG Sor:0.00010001

#MGE Sor: 0.00020002

HornRA_ARG

HornRA_ARG<- ggplot(zzRAARG, aes(x = distanceRAARG, fill = factor(comb))) + geom_density(alpha = 0.5) + theme_minimal()+

  theme(legend.justification = c(0.05, 1), legend.position = c(0.05, 1), legend.text = element_text(size = rel(0.6))) +

  scale_fill_manual(values = c("brown1", "dodgerblue ", "gold"), labels = c("Different family, mother and infant",

                                            "Different family, same type", "Same family, mother and infant"), "Comparisons") +

  annotate("text", x = 0.5, y = 3, label = "type, p=0.0001   \ntype and family, p=0.0187 \nfamily, p=0.8830   ", cex=3) +

  xlab("Between-sample Bray-Curtis dissimilarity ") + theme(axis.title.x = element_text(size=7)) + ggtitle("ARG") +

  ylim(0, 5)

HornRA_ARG

HornRA_MGE<- ggplot(zzRAMGE, aes(x = distanceRAMGE, fill = factor(comb))) + geom_density(alpha = 0.5) + theme_minimal() +

  theme(legend.justification = c(0.05, 1), legend.position = c(0.05, 1), legend.text = element_text(size = rel(0.8))) +

```
  scale_fill_manual(values = c("brown1", "dodgerblue ", "gold"), labels = c("Different family, mother and infant",
                                                "Different family, same type", "Same family, mother and infant"), "Comparisons") +
  annotate("text", x = 0.5, y = 5, label = "type, p=0.0001    \ntype and family, p=0.3051 \nfamily, p=0.5392                        ", cex=3) +
  xlab("Between-sample Bray-Curtis dissimilarity ") + theme(axis.title.x = element_text(size=7))+ ggtitle("MGE") +
  ylim(0, 8)
HornBI_ARG<- ggplot(zzBIARG, aes(x = distanceBIARG, fill = factor(comb))) + geom_density(alpha = 0.5) +  theme_minimal()+
  theme(legend.justification = c(0.05, 1), legend.position = c(0.05, 1), legend.text = element_text(size = rel(0.8))) +
  scale_fill_manual(values = c("brown1", "dodgerblue ", "gold"), labels = c("Different family, mother and infant",
                                                "Different family, same type", "Same family, mother and infant"), "Comparisons") +
  annotate("text", x = 0.7, y = 5, label = "type, p=0.0001    \ntype and family, p=0.0002 \nfamily, p=0.0879", cex=3) +
  xlab("Between-sample Sorensen dissimilarity ") + theme(axis.title.x = element_text(size=7)) + ggtitle("ARG") +
  ylim(0, 6)
HornBI_MGE<- ggplot(zzBIMGE, aes(x = distanceBIMGE, fill = factor(comb))) + geom_density(alpha = 0.5) +  theme_minimal() +
  theme(legend.justification = c(0.05, 1), legend.position = c(0.05, 1), legend.text = element_text(size = rel(0.8))) +
  scale_fill_manual(values = c("brown1", "dodgerblue ", "gold"), labels = c("Different family, mother and infant",
                                                "Different family, same type", "Same family, mother and infant"), "Comparisons") +
```

```
  annotate("text", x = 0.8, y = 4, label = "type, p=0.0002    \ntype and family, p=0.8391
\nfamily, p=0.2254                              ", cex=3) +

  xlab("Between-sample Sorensen dissimilarity") + theme(axis.title.x =
element_text(size=7)) + ggtitle("MGE") +

  ylim(0, 5)


library(ggplot2)

figure <- ggarrange(HornRA_ARG, HornRA_MGE,  HornBI_ARG, HornBI_MGE, labels = c("A",
"B", "C", "D", ncol = 2, nrow = 2), common.legend = TRUE, legend="bottom")

figure

#ggexport(figure, filename = "/Users/andreasosamoreno/Desktop/LixinData/Mother-
Child/DensityPlots_WomenandChild.pdf")


##### Pie Chart####


#did this already -- instead pull these files from above

# abundance_pair<-abundance3mos[which(abundance3mos$pair>0),]

# abundance_pair<-abundance_pair[-grep("121",abundance_pair$pair),]

# abundance_pair$pair<-as.numeric(abundance_pair$pair)

# abundance_pairs<-
abundance_pair[duplicated(abundance_pair$pair)|duplicated(abundance_pair$pair,
fromLast=TRUE),]

# is.numeric(abundance_pairs$pair)

# head(abundance_pairs$pair)

# str(abundance_pairs)
```

```
#write.csv(abundance_pairs, "/Users/madeleinerussell/Desktop/Comstock Lab/AbxResistance/ABXR_Ranalysis/AMR_Data/2022DEC05_abundance3mos_plusmoms_pairs_MR.csv", row.names = FALSE)

#only those who we have matching data
# presence_pair<-presence3mos[which(presence3mos$pair>0),]
# presence_pair<-presence_pair[-grep("121",presence_pair$pair),]
# presence_pair$pair<-as.numeric(presence_pair$pair)
# df3<-presence_pair[duplicated(presence_pair$pair)|duplicated(presence_pair$pair, fromLast=TRUE),]
# is.numeric(df3$pair)
# head(df3$pair)
# str(df3)

abundance3mos.matched<-read.xlsx("2023APR18_AMR_3mos_matchedmoms_bbys.xlsx")
presence3mos.matched<-read.xlsx("2023APR18_pres_3mos_matchedmoms_bbys.xlsx")

presence<-presence3mos.matched
abundance<-abundance3mos.matched

df3<-presence
#getting max and min shared genes
All<-df3[,c(4:146)]
sharedgenes<-colSums(All)
a<-data.frame(sharedgenes)
df2 <- tibble::rownames_to_column(a, "Genename")
```

```
summary(df2)

write.xlsx(df2, "/Users/madeleinerussell/Desktop/Comstock Lab/AbxResistance/ABXR_Ranalysis/AMR_Data/APR28_Data/2023APR28_matchedpairs_sharedgenes_MR.xlsx", quote=FALSE, rowNames=FALSE)


#select for ARG or MGE

#149 is the complete pair list

ARG<-df3[,c(149,31:146)]

MGE<-df3[,c(149,4:30)]


#selecting all genes

All<-df3[,c(149,4:146)]

#creating function that basically groups by the pair, and if it is present in at least

#either mom and baby, add them (ie give it a 2 or a 1 or a 0)

Allpair_sums<-All %>%

  group_by(pair.y) %>%

  filter(n() > 1) %>%

  dplyr::summarise(across(1:143, (sum)))

Pieindex<- Allpair_sums[,c("pair.y")]


Allpair_sums_remove_ID<- data.frame(row.names = Allpair_sums[,1])

Allpair_sums_remove_ID<-Allpair_sums[,c(2:144)]

rownames(Allpair_sums_remove_ID)<-Allpair_sums$pair.y


Shared_fun <- function(m){

  m2 <- apply(m,  2,  function(x) {ifelse(x > 1, 1, 0)})

  colnames(m2) <- colnames(m)
```

```
  rownames(m2) <- rownames(m)
  return(m2)
}
Allshared<-Shared_fun(Allpair_sums_remove_ID)
#MGEshared$allMGEshared<-rowSums(MGEshared)
Occurs_fun <- function(m){
  m2 <- apply(m,  2,  function(x) {ifelse(x > 0, 1, 0)})
  colnames(m2) <- colnames(m)
  rownames(m2) <- rownames(m)
  return(m2)
}
ALLoccurs<-Occurs_fun(Allpair_sums_remove_ID)


Allchild<-df3[,c(2, 149, 4:146)]
Allchild2<-Allchild[grep("3mos", Allchild$Timepoint), ]
#MGEChild_remove_ID<-MGE.child2[,c(3:29)]
#rownames(MGE.child2)<-MGE.child2$pair
Allchild2<-Allchild2[order(Allchild2$pair.y),]
All.child.occurs<-Occurs_fun(Allchild2[,-c(1:2)])
rownames(All.child.occurs)<-Allchild2$pair


#find shared MGE
Pieindex$AllShared<-rowSums(Allshared)
Pieindex$AllOccurs<-rowSums(ALLoccurs)
```

```
Pieindex$All.child.occurs<-rowSums(All.child.occurs)


#second index because easier to visualize

Pie2<- Allpair_sums[,c("pair.y")]

rownames(Pie2)<-Pie2$pair.y


#summing ARG + MGE

percentshared<-(Pieindex$AllShared/Pieindex$All.child.occurs)

percentnotshared<-((Pieindex$All.child.occurs-
Pieindex$AllShared)/Pieindex$All.child.occurs)


#creating two data frames so can combine them

test<- Allpair_sums[,c("pair.y")]

test$type<-"common"

test$number<-(Pieindex$AllShared)

test$origin<-"maternal"

test$percentshared<-(Pieindex$AllShared/Pieindex$All.child.occurs)


#creating other data frame

test2<- Allpair_sums[,c("pair.y")]

test2$type<-"child"

test2$number<-(Pieindex$All.child.occurs)

test2$origin<-"unknown"

test2$percentnotshared<-((Pieindex$All.child.occurs-
Pieindex$AllShared)/Pieindex$All.child.occurs)
```

```
#combine them!
library(dplyr)
test3<-bind_rows(test2, setNames(test, names(test2))) %>%
  arrange(pair.y)


#make it into a csv, read in weird so removed a column
write.csv (test3, "2023AP28_SharedGenesMotherinfants.3mos.csv")
multiplepie<-read.csv("2023AP28_SharedGenesMotherinfants.3mos.csv")
#put rownames as a column, so remove that
multiplepie<-multiplepie[,-c(1)]


#lets see if it works to make a pie chart with shared genes
colnames(multiplepie)[5]  <- "percentage"    # change column name for x column


df <- multiplepie #remake so it won't mess it up


df$percentage<-signif(df$percentage, 2) #limiting sig figs
df$percentage


df <- df %>%
  mutate(labels = scales::percent(percentage))


df$labels


#for labels in the plots
#this is andreas code-- I modified to show labels
```

```
# ggplot(df, aes(x = "", y = percentage, fill = factor(origin))) +
#   geom_bar(stat = "identity", width = 0.01) +
#   coord_polar(theta = "y") +
#   facet_wrap(~pair.y)+ labs(x = NULL, y = NULL, fill = NULL)+
#   theme_classic()+ theme(axis.line = element_blank(), axis.text =
element_blank(),axis.ticks = element_blank())+theme(strip.background =
element_blank(),strip.text.x = element_blank())+
#   scale_fill_manual(values=c("dodgerblue3 ", "grey50"))


ggplot(df, aes(x = "", y = percentage, fill = factor(origin))) +
  geom_col(color = "black") +
  geom_label(aes(label = labels), size = 3, color = "black",
             position = position_stack(vjust = 0.5),
             show.legend = FALSE) +
  facet_wrap(~pair.y)+ labs(x = NULL, y = NULL, fill = NULL) +
  guides(fill = guide_legend(title = "Shared")) +
  coord_polar(theta = "y") +
  theme_void()
df$MOD<-gsub("69|82|84|106|119|121|132|138|147", "Cesarean", df$pair.y)
df$MOD
df$MOD<-as.factor(ifelse(df$MOD== 'Cesarean','Cesarean','Vaginal'))
df$MOD


###mode of delivery
bcdata2$MOD<-as.factor(ifelse(bcdata2$MD_FINAL_ROUTE== '1','Vaginal',
                        ifelse(bcdata2$MD_FINAL_ROUTE== '2', 'Vaginal',
                           ifelse(bcdata2$MD_FINAL_ROUTE== '3', 'Vaginal',
```

```
                                    ifelse(bcdata2$MD_FINAL_ROUTE== '4', 'Cesarean', 'other')))))
table(bcdata$pair, bcdata2$MOD)

names(bcdata2)[names(bcdata2) == 'pair.y'] <- 'pair'
bcdata2$pair
sharedpie<-multiplepie[grep("common", multiplepie$type), ]

summary(sharedpie$percentage)
names(sharedpie)[names(sharedpie) == 'pair.y'] <- 'pair'
sharedpie$pair<-as.factor(as.character(sharedpie$pair))

sharedgenes2<-inner_join(sharedpie, bcdata2, by='pair')
sharedgenes2

table(sharedgenes2$pair, sharedgenes2$MOD)

pies<-ggplot(df, aes(x = "", y = percentage, fill = factor(origin))) +
  geom_col(color = "black") +
  geom_label(aes(label = labels), size = 3, color = "white",
          position = position_stack(vjust = 0.5),
          show.legend = FALSE) +
  facet_wrap(~pair.y)+ labs(x = NULL, y = NULL, fill = NULL) +
  theme(axis.line = element_blank(), axis.text = element_blank(),axis.ticks =
element_blank())+theme(strip.background = element_blank(),strip.text.x =
element_blank()) +
  guides(fill = guide_legend(title = "Shared")) +
  coord_polar(theta = "y")
```

```
pies

type %in% c("b", "c")


df$labels

df$percentage

df$origin

pies2<-ggplot(df, aes(x = "", y = percentage, fill = factor(origin))) +
  geom_col(color = "black") +
  geom_label(aes(label = labels), size = 4, color = "black",
             position = position_stack(vjust = 0.5),
             show.legend = FALSE) +
  geom_rect(data = subset(df, pair.y %in% c("69","82","84","106","119","121",
"132","138","147") & MOD %in% c("Cesarean")),
            fill = NA, color = "red", xmin = -Inf, xmax = Inf,
            ymin = -Inf,ymax = Inf, linewidth = 1.5, ) +
  facet_wrap(~pair.y)+ labs(x = NULL, y = NULL, fill = NULL) +
  theme(axis.line = element_blank(), axis.text = element_blank(),axis.ticks =
element_blank())+theme(strip.background = element_blank(),strip.text.x =
element_blank()) +
  guides(fill = guide_legend(title = "Shared")) +
  coord_polar(theta = "y")

pies3<-pies2 + scale_fill_brewer(palette="Accent")

pies3


library(ggplot2)

library(ggh4x)

#install.packages('ggforce')
```

```
library(ggforce)


# mark which panels should be highlighted

library(ggplot2)

library(dplyr)


highlight_panels <- df %>%

  filter(pair.y %in% c("69","82","84","106","119","121","132","138","147"),

         MOD == "Cesarean")


pies3<- pies3 +

  # Add a "ghost pie" as an outline

  geom_col(

    data = highlight_panels,

    aes(x = "", y = percentage),    # same setup as real pie

    fill = NA, color = "red", linewidth = 1.5,

    inherit.aes = FALSE

  )

pies3




#take 2

library(dplyr)

library(ggplot2)


# Compute maternal % per pair
```

```
library(dplyr)
library(ggplot2)

df

# Compute maternal % per pair (grab percentage + labels)
maternal_labels <- df %>%
  filter(origin == "maternal") %>%
  distinct(pair.y, .keep_all = TRUE) %>%
  dplyr::select(pair.y, labels)



df <- df %>%
  group_by(pair.y) %>%
  mutate(ordering_val = percentage[origin == "maternal"]) %>%
  ungroup() %>%
  mutate(pair.y = reorder(pair.y, -ordering_val))

# Build custom facet labels: pair ID + % maternal
facet_labels <- maternal_labels %>%
  mutate(facet_lab = paste0(pair.y, " (", labels, ")")) %>%
  tibble::deframe()

# Use Arial everywhere
base_theme <- theme_minimal(base_family = "Helvetica") +
  theme(
```

```
    axis.line   = element_blank(),
    axis.text   = element_blank(),
    axis.ticks  = element_blank(),
    panel.grid  = element_blank(),
    panel.background = element_rect(fill = "transparent", colour = NA),
    plot.background  = element_rect(fill = "transparent", colour = NA),
    strip.background = element_blank(),
    strip.text.x     = element_text(size = 16, face = "bold"),
    legend.position  = "top",
    legend.title     = element_text(size = 18, face = "bold", family = "Helvetica"),
    legend.text      = element_text(size = 16, face = "bold", family = "Helvetica"),
    legend.background = element_rect(fill = "transparent", colour = NA),
    legend.key       = element_rect(fill = "transparent", colour = NA)
  )
# Plot pies
# pies2 <- ggplot(df, aes(x = "", y = percentage, fill = factor(origin))) +
#   geom_col(color = "black") +
#   facet_wrap(~pair.y, labeller = labeller(pair.y = facet_labels)) +
#   labs(x = NULL, y = NULL, fill = NULL) +
#   theme(
#     axis.line = element_blank(),
#     axis.text = element_blank(),
#     axis.ticks = element_blank(),
#     strip.background =  element_rect(fill = NA, colour = NA),
#     strip.text.x = element_text(size = 16),
#     legend.position = "top"
```

```
#   ) +
#   guides(fill = guide_legend(title = "Shared")) +
#   coord_polar(theta = "y") +
#   scale_fill_brewer(palette = "Accent")
# pies2

# Apply to pies
pies2 <- ggplot(df, aes(x = "", y = percentage, fill = factor(origin))) +
  geom_col(color = "black") +
  facet_wrap(~pair.y, labeller = labeller(pair.y = facet_labels)) +
  labs(x = NULL, y = NULL, fill = "Shared") +
  guides(fill = guide_legend(title.position = "top")) +
  coord_polar(theta = "y") +
  scale_fill_brewer(palette = "Accent") +
  base_theme

# Highlight Cesarean pies
highlight_panels <- df %>%
  filter(pair.y %in% c("69","82","84","106","119","121","132","138","147"),
         MOD == "Cesarean")

pies3 <- pies2 +
  geom_col(
    data = highlight_panels,
    aes(x = "", y = percentage),
    fill = NA, color = "red", linewidth = 1.5,
```

```
  inherit.aes = FALSE
 )

pies3



library(ggpubr)
library("ggsci")
shared_MOD_box<-ggboxplot(sharedgenes2, "MOD", "percentage",
                    add = "mean",
                    fill = "MOD",
                    palette = c("#3399FF","#E7B800"),
                    title = "Shared Genes",
                    xlab = "Mode of Delivery",
                    ylab = "Proportion of Shared Genes")
shared_MOD_box
shared_MOD_box2<-shared_MOD_box + annotate("text", x = 1.5, y = 1.0,  label = "p = 
0.975")
shared_MOD_box2

shared_MOD_box2 <- shared_MOD_box +
 annotate("text",
        x = 1.5,
        y = 1.0,
        label = "p = 0.975",
        fontface = "bold",   # makes text bold
```

```
        size = 6)           # adjust size if you want larger text
shared_MOD_box2


shared_MOD_box2 <- shared_MOD_box +
 annotate("text",
        x = 1.5,
        y = 1.0,
        label = "p = 0.975",
        fontface = "bold",
        size = 6) +          # larger p-value text
 theme(
  legend.position = "none",
  plot.title = element_text(size = 16, face = "bold"),
  axis.title.x = element_text(size = 14, face = "bold"),   # bigger x-axis label
  axis.title.y = element_text(size = 12, face = "bold"),   # bigger y-axis label
  axis.text.x  = element_text(size = 12),                # tick labels (x)
  axis.text.y  = element_text(size = 14)                 # tick labels (y)
 )
shared_MOD_box2


shapiro.test(sharedgenes2$percentage)
#p-value: p-value = 0.01836
a<-sharedgenes2$MOD
perc<-sharedgenes2$percentage


wilcox.test(sharedgenes2$percentage~sharedgenes2$MOD, data=sharedgenes2)
```

```
#p-value = 0.9746


figure <- ggarrange(pies3, shared_MOD_box2,labels = c("A", "B", ncol = 2, nrow = 1))
figure


pies4 <- pies3 +
  facet_wrap(~pair.y, labeller = labeller(pair.y = facet_labels), ncol = 6) +
  theme(
    plot.background = element_rect(fill = "white", color = NA),
    panel.background = element_rect(fill = "white", color = NA),
    panel.border= element_blank(),
    aspect.ratio = 1,
    panel.spacing = unit(0.2, "lines"),
    strip.text = element_text(size = 12)
  )


pies4


shared_MOD_box2 <- shared_MOD_box2 +
  theme(
    plot.background = element_rect(fill = "white", color = NA),
    panel.background = element_rect(fill = "white", color = NA),
    panel.border     = element_blank()
  )
```

```
figure <- ggarrange(
  pies4,
  shared_MOD_box2,
  labels = c("A", "B"),
  ncol = 2,
  nrow = 1,
  widths = c(2, 1)   # pies3 gets twice the space of boxplot
)



figure


# Save as TIFF (print ready)
ggsave("pies_figure_pub2.tiff", figure,
       width = 6.5, height = 9, units = "in",
       bg = "white",
       dpi = 600,
       compression = "lzw")



# Apply to your plots




###id genes that are shared between mom and baby by type #####
abundance3mos.matched<-
read.xlsx("2023APR18_AMR_3mos_matchedmoms_bbys.xlsx")
```

```
presence3mos.matched<-read.xlsx("2023APR18_pres_3mos_matchedmoms_bbys.xlsx")

write.csv(presence3mos.matched, "/Users/madeleinerussell/Desktop/Comstock Lab/AbxResistance/ABXR_Ranalysis/AMR_Data/2023APR20_presence3mos_matched_MR.csv", row.names = FALSE)

write.csv(abundance3mos.matched, "/Users/madeleinerussell/Desktop/Comstock Lab/AbxResistance/ABXR_Ranalysis/AMR_Data/2023APR20_abundance3mos_matched_MR.csv", row.names = FALSE)



#looking for genes most abundant in moms samples######

binary<-read.csv("2023APR20_presence3mos_matched_MR.csv", header=TRUE, row.names=1)

str(binary)


binary2<-binary[,3:145]

#str(binary3) #we have data from 99 women


#To have gene presence in at least 25% of samples the gene has to be present in at least 24 samples

library(tibble)


#this next little chunk was to get the names of the most abundant present number of genes

binary2<-binary[,3:145]

minimum<-colSums(binary2)

summary(minimum)

a<-data.frame(minimum)

df2 <- tibble::rownames_to_column(a, "Genename")
```

```
summary(df2)

df2 %>% group_by(Genename) %>% slice_max(n = 1, minimum)


#write.xlsx(df2, "/Users/madeleinerussell/Desktop/Comstock Lab/AbxResistance/ABXR_Ranalysis/AMR_Data/APR28_data/2023APR28_3mos_matched_geneslist_MR.xlsx", quote=FALSE, rowNames=FALSE)


#####Creating Pie Chart of Shared Genes######


library('sqldf')

# array_name = sqldf("select DISTINCT *colname1* as '*column_title*' from *table_name*")

# # factor1 <- sqldf("select distinct abx1type as 'abx_type' from metadata")

# factor1

#add in meta data

#metadata<-read.xlsx("2023APR17_AMR_3mosMetaData.xlsx")

metadata<-read.xlsx("2023APR17_AMR_3mosMetaData_only.xlsx")


matched.meta<-inner_join(abundance3mos.matched, metadata, by='ID')


# metadata<-meta[,-c(233)]

write.xlsx(metadata, "/Users/madeleinerussell/Desktop/Comstock Lab/AbxResistance/ABXR_Ranalysis/AMR_Data/2023APR24_AMR_3mos_MetaData_plusanti.xlsx", quote=FALSE, rowNames=FALSE)
```

```
abx<-levels(as.factor(as.character(metadata$abx1type)))

metadata$abx1type

levels(abx)


factor1 <- sqldf("select distinct abx1type as 'abx_type' from metadata")

factor1


# antifungal

# cephalosporin

# macrolide

# nitrofuran

# nitromidazol

# penicillin

# other




#first abx

metadata$first_antityp<-
gsub(".*metro.*|.*Metro.*|.*Keflex.*|.*keflex.*|.*flagyl.*|.*Flagyl.*|.*tinidazole.*",
"nitromidazol",

                        gsub(".*nitrofur.*|.*Nitrofur.*|.*Macrobid.*", "nitrofuran",

                            gsub(".*augmentin.*|.*Augmentin.*|.*amoxicillin.*|.*Amp.*|.*Amoxicillin.*",
"penicillin",

                                gsub(".*Bactrim.*", "sulfamethoxazole",
```

```
gsub(".*Azithromycin.*|.*azithromycin.*|.*clindamycin.*|.*Clindamycin.*|.*Z pack.*|.*Zithromax.*", "macrolide",
                                  gsub(".*doxycycline.*", "tetracycline",
                                     gsub(".*Ceftriaxone.*|.*cefixime.*|.*Cephalexin.*|.*cephalexin.*", "cephalosporin",
                                        gsub(".*Diflucan.*|.*Terazol.*", "anti-fungal",
                                           gsub(".*Bacterial Vaginosis.*|.*not documented.*", "NA", metadata$abx1type)))))))))

df1 = metadata

factor2 <- sqldf("select distinct first_antityp as 'abx_type' from df1")

factor2


levels(metadata$first_antityp)

levels(as.factor(as.character(metadata$first_antityp)))


#second abx

metadata$second_antityp<-
gsub(".*metro.*|.*Metro.*|.*Keflex.*|.*keflex.*|.*flagyl.*|.*Flagyl.*|.*tinidazole.*", "nitromidazol",
                    gsub(".*nitrofur.*|.*Nitrofur.*|.*Macrobid.*", "nitrofuran",
                       gsub(".*augmentin.*|.*Augmentin.*|.*amoxicillin.*|.*Amp.*|.*Amoxicillin.*", "penicillin",
                          gsub(".*Bactrim.*", "sulfamethoxazole",

gsub(".*Azithromycin.*|.*azithromycin.*|.*clindamycin.*|.*Clindamycin.*|.*Z pack.*|.*Zithromax.*", "macrolide",
                                  gsub(".*doxycycline.*", "tetracycline",
```

```
                                   gsub(".*Ceftriaxone.*|.*cefixime.*|.*Cephalexin.*|.*cephalexin.*",
"cephalosporin",
                                     gsub(".*Diflucan.*|.*Terazol.*", "anti-fungal",
                                       gsub(".*acetasol.*", "other",
                                         gsub(".*Bacterial Vaginosis.*|.*not documented.*", "NA",
metadata$abxtype2))))))))))
metadata$second_antityp
metadata$third_antityp


df1 = metadata
factor2 <- sqldf("select distinct second_antityp as 'abx_type' from df1")
factor2
levels(as.factor(as.character(metadata$second_antityp)))


#third abx
metadata$third_antityp<-
gsub(".*metro.*|.*Metro.*|.*Keflex.*|.*keflex.*|.*flagyl.*|.*Flagyl.*|.*tinidazole.*",
"nitromidazol",
            gsub(".*nitrofur.*|.*Nitrofur.*|.*Macrobid.*", "nitrofuran",
              gsub(".*augmentin.*|.*Augmentin.*|.*amoxicillin.*|.*Amp.*|.*Amoxicillin.*",
"penicillin",
                gsub(".*Bactrim.*", "sulfamethoxazole",

gsub(".*Azithromycin.*|.*azithromycin.*|.*clindamycin.*|.*Clindamycin.*|.*Z
pack.*|.*Zithromax.*", "macrolide",
                    gsub(".*doxycycline.*", "tetracycline",

gsub(".*Ceftriaxone.*|.*cefixime.*|.*Cephalexin.*|.*cephalexin.*|.*ancef.*", "cephalosporin",
```

```
                                             gsub(".*Diflucan.*|.*Terazol.*", "anti-fungal",
                                                gsub(".*acetasol.*", "other",
                                                   gsub(".*Bacterial Vaginosis.*|.*not documented.*", "NA",
metadata$abxtype3))))))))))



df1 = metadata

factor2 <- sqldf("select distinct third_antityp as 'abx_type' from df1")

factor2

levels(as.factor(as.character(metadata$third_antityp)))


#fourth abx

metadata$fourth_antityp<-
gsub(".*metro.*|.*Metro.*|.*Keflex.*|.*keflex.*|.*flagyl.*|.*Flagyl.*|.*tinidazole.*",
"nitromidazol",

                    gsub(".*nitrofur.*|.*Nitrofur.*|.*Macrobid.*", "nitrofuran",

                       gsub(".*augmentin.*|.*Augmentin.*|.*amoxicillin.*|.*Amp.*|.*Amoxicillin.*",
"penicillin",

                          gsub(".*Bactrim.*", "sulfamethoxazole",

                             gsub(".*Azithromycin.*|.*azithromycin.*|.*clindamycin.*|.*mycin.*|.*Z
pack.*|.*Zithromax.*", "macrolide",

                                gsub(".*doxycycline.*", "tetracycline",


gsub(".*Ceftriaxone.*|.*cefixime.*|.*Cephalexin.*|.*cephalexin.*|.*ancef.*", "cephalosporin",

                                             gsub(".*Diflucan.*|.*Terazol.*", "anti-fungal",

                                                gsub(".*acetasol.*", "other",

                                                   gsub(".*Bacterial Vaginosis.*|.*not documented.*", "NA",
metadata$abxtype4))))))))))
```

```
df1 = metadata

factor2 <- sqldf("select distinct fourth_antityp as 'abx_type' from df1")

factor2

levels(as.factor(as.character(metadata$fourth_antityp)))


#fifth abx

metadata$fifth_antityp<-
gsub(".*metro.*|.*Metro.*|.*Keflex.*|.*keflex.*|.*flagyl.*|.*Flagyl.*|.*tinidazole.*",
"nitromidazol",

                    gsub(".*nitrofur.*|.*Nitrofur.*|.*Macrobid.*", "nitrofuran",

                       gsub(".*augmentin.*|.*Augmentin.*|.*amoxicillin.*|.*Amp.*|.*Amoxicillin.*",
"penicillin",

                          gsub(".*Bactrim.*", "sulfamethoxazole",

                             gsub(".*Azithromycin.*|.*azithromycin.*|.*clindamycin.*|.*mycin.*|.*Z
pack.*|.*Zithromax.*", "macrolide",

                                gsub(".*doxycycline.*", "tetracycline",


gsub(".*Ceftriaxone.*|.*cefixime.*|.*Cephalexin.*|.*cephalexin.*|.*ancef.*", "cephalosporin",

                                      gsub(".*Diflucan.*|.*Terazol.*", "anti-fungal",

                                         gsub(".*acetasol.*", "other",

                                            gsub(".*Bacterial Vaginosis.*|.*not documented.*", "NA",
metadata$abxtype5))))))))))



df1 = metadata

factor2 <- sqldf("select distinct fifth_antityp as 'abx_type' from df1")
```

```
factor2

levels(as.factor(as.character(metadata$fifth_antityp)))


#sixth abx

metadata$sixth_antityp<-
gsub(".*metro.*|.*Metro.*|.*Keflex.*|.*keflex.*|.*flagyl.*|.*Flagyl.*|.*tinidazole.*",
"nitromidazol",

                      gsub(".*nitrofur.*|.*Nitrofur.*|.*Macrobid.*", "nitrofuran",

                          gsub(".*augmentin.*|.*Augmentin.*|.*amoxicillin.*|.*Amp.*|.*Amoxicillin.*",
"penicillin",

                              gsub(".*Bactrim.*", "sulfamethoxazole",

                                  gsub(".*Azithromycin.*|.*azithromycin.*|.*clindamycin.*|.*mycin.*|.*Z
pack.*|.*Zithromax.*", "macrolide",

                                      gsub(".*doxycycline.*", "tetracycline",


gsub(".*Ceftriaxone.*|.*cefixime.*|.*Cephalexin.*|.*cephalexin.*|.*ancef.*", "cephalosporin",

                                              gsub(".*Diflucan.*|.*Terazol.*", "anti-fungal",

                                                  gsub(".*acetasol.*", "other",

                                                      gsub(".*Bacterial Vaginosis.*|.*not documented.*", "NA",
metadata$abxtype6))))))))))



df1 = metadata

factor2 <- sqldf("select distinct sixth_antityp as 'abx_type' from df1")

factor2

levels(as.factor(as.character(metadata$sixth_antityp)))
```

```
###making pie chart
ID <- seq.int(nrow(metadata))

first_antityp<-metadata$first_antityp
second_antityp<- metadata$second_antityp
third_antityp<- metadata$third_antityp
fourth_antityp<- metadata$fourth_antityp
fifth_antityp<- metadata$fifth_antityp

data_antfreq<-data.frame(ID, first_antityp, second_antityp, third_antityp, fourth_antityp,
fifth_antityp)
data_antfreq<- melt(data_antfreq, id=c('ID'))

#Classification of medication exposure : medication ever used during pregnancy

dev.off()
#Pie chart with distribution of medications during pregnancy
a<-table(data_antfreq$value)
abx<-levels(as.factor(as.character(data_antfreq$value)))
piepercent <- round(100*a/sum(a),1)
piepercent<-paste(piepercent,"%",sep="")
pie(a,labels=piepercent, main="Antimicrobial prescription distribution among pregnant
women", col=rainbow(length(a)))
legend(-1.6,- 0.1, legend=abx, cex = 0.5, fill = rainbow(length(a)))

table(data_antfreq$value)
#####pie chart all 267 infants####
```

```
metadata.all<-read.xlsx("2023FEB03_AMR_MetaData.xlsx")

metadata<-metadata.all


#first abx

metadata$first_antityp<-
gsub(".*metro.*|.*Metro.*|.*Keflex.*|.*keflex.*|.*flagyl.*|.*Flagyl.*|.*tinidazole.*",
"nitromidazol",

                    gsub(".*nitrofur.*|.*Nitrofur.*|.*Macrobid.*", "nitrofuran",

                       gsub(".*augmentin.*|.*Augmentin.*|.*amoxicillin.*|.*Amp.*|.*Amoxicillin.*",
"penicillin",

                          gsub(".*Bactrim.*", "sulfamethoxazole",


gsub(".*Azithromycin.*|.*azithromycin.*|.*clindamycin.*|.*Clindamycin.*|.*Z
pack.*|.*Zithromax.*", "macrolide",

                                gsub(".*doxycycline.*", "tetracycline",

                                   gsub(".*Ceftriaxone.*|.*cefixime.*|.*Cephalexin.*|.*cephalexin.*",
"cephalosporin",

                                      gsub(".*Diflucan.*|.*Terazol.*", "anti-fungal",

                                         gsub(".*Bacterial Vaginosis.*|.*not documented.*", "NA",
metadata$abx1type)))))))))

df1 = metadata

factor2 <- sqldf("select distinct first_antityp as 'abx_type' from df1")

factor2


levels(metadata$first_antityp)

levels(as.factor(as.character(metadata$first_antityp)))
```

```
#second abx

metadata$second_antityp<-
gsub(".*metro.*|.*Metro.*|.*Keflex.*|.*keflex.*|.*flagyl.*|.*Flagyl.*|.*tinidazole.*",
"nitromidazol",

            gsub(".*nitrofur.*|.*Nitrofur.*|.*Macrobid.*", "nitrofuran",

              gsub(".*augmentin.*|.*Augmentin.*|.*amoxicillin.*|.*Amp.*|.*Amoxicillin.*",
"penicillin",

                gsub(".*Bactrim.*", "sulfamethoxazole",


gsub(".*Azithromycin.*|.*azithromycin.*|.*clindamycin.*|.*Clindamycin.*|.*Z
pack.*|.*Zithromax.*", "macrolide",

                    gsub(".*doxycycline.*", "tetracycline",

                      gsub(".*Ceftriaxone.*|.*cefixime.*|.*Cephalexin.*|.*cephalexin.*",
"cephalosporin",

                        gsub(".*Diflucan.*|.*Terazol.*", "anti-fungal",

                          gsub(".*acetasol.*", "other",

                            gsub(".*Bacterial Vaginosis.*|.*not documented.*", "NA",
metadata$abxtype2))))))))))



df1 = metadata

factor2 <- sqldf("select distinct second_antityp as 'abx_type' from df1")

factor2


#third abx

metadata$third_antityp<-
gsub(".*metro.*|.*Metro.*|.*Keflex.*|.*keflex.*|.*flagyl.*|.*Flagyl.*|.*tinidazole.*",
"nitromidazol",

            gsub(".*nitrofur.*|.*Nitrofur.*|.*Macrobid.*", "nitrofuran",
```

```
                    gsub(".*augmentin.*|.*Augmentin.*|.*amoxicillin.*|.*Amp.*|.*Amoxicillin.*", 
"penicillin",
                       gsub(".*Bactrim.*", "sulfamethoxazole",
                                                  
gsub(".*Azithromycin.*|.*azithromycin.*|.*clindamycin.*|.*Clindamycin.*|.*Z 
pack.*|.*Zithromax.*", "macrolide",
                             gsub(".*doxycycline.*", "tetracycline",
                                                        
gsub(".*Ceftriaxone.*|.*cefixime.*|.*Cephalexin.*|.*cephalexin.*|.*ancef.*", "cephalosporin",
                                   gsub(".*Diflucan.*|.*Terazol.*", "anti-fungal",
                                      gsub(".*acetasol.*", "other",
                                         gsub(".*Bacterial Vaginosis.*|.*not documented.*", "NA", 
metadata$abxtype3))))))))))



df1 = metadata

factor2 <- sqldf("select distinct third_antityp as 'abx_type' from df1")

factor2


#fourth abx

metadata$fourth_antityp<-
gsub(".*metro.*|.*Metro.*|.*Keflex.*|.*keflex.*|.*flagyl.*|.*Flagyl.*|.*tinidazole.*", 
"nitromidazol",
                  gsub(".*nitrofur.*|.*Nitrofur.*|.*Macrobid.*", "nitrofuran",
                     gsub(".*augmentin.*|.*Augmentin.*|.*amoxicillin.*|.*Amp.*|.*Amoxicillin.*", 
"penicillin",
                        gsub(".*Bactrim.*", "sulfamethoxazole",
                           gsub(".*Azithromycin.*|.*azithromycin.*|.*clindamycin.*|.*mycin.*|.*Z 
pack.*|.*Zithromax.*", "macrolide",
```

```
gsub(".*doxycycline.*", "tetracycline",

gsub(".*Ceftriaxone.*|.*cefixime.*|.*Cephalexin.*|.*cephalexin.*|.*ancef.*", "cephalosporin",
gsub(".*Diflucan.*|.*Terazol.*", "anti-fungal",
gsub(".*acetasol.*", "other",
gsub(".*Bacterial Vaginosis.*|.*not documented.*", "NA",
metadata$abxtype4))))))))))

df1 = metadata
factor2 <- sqldf("select distinct fourth_antityp as 'abx_type' from df1")
factor2

#fifth abx
metadata$fifth_antityp<-
gsub(".*metro.*|.*Metro.*|.*Keflex.*|.*keflex.*|.*flagyl.*|.*Flagyl.*|.*tinidazole.*",
"nitromidazol",
gsub(".*nitrofur.*|.*Nitrofur.*|.*Macrobid.*", "nitrofuran",
gsub(".*augmentin.*|.*Augmentin.*|.*amoxicillin.*|.*Amp.*|.*Amoxicillin.*",
"penicillin",
gsub(".*Bactrim.*", "sulfamethoxazole",
gsub(".*Azithromycin.*|.*azithromycin.*|.*clindamycin.*|.*mycin.*|.*Z
pack.*|.*Zithromax.*", "macrolide",
gsub(".*doxycycline.*", "tetracycline",

gsub(".*Ceftriaxone.*|.*cefixime.*|.*Cephalexin.*|.*cephalexin.*|.*ancef.*", "cephalosporin",
gsub(".*Diflucan.*|.*Terazol.*", "anti-fungal",
gsub(".*acetasol.*", "other",
```

```
                                   gsub(".*Bacterial Vaginosis.*|.*not documented.*", "NA",
metadata$abxtype5))))))))))



df1 = metadata

factor2 <- sqldf("select distinct fifth_antityp as 'abx_type' from df1")

factor2


#sixth abx

metadata$sixth_antityp<-
gsub(".*metro.*|.*Metro.*|.*Keflex.*|.*keflex.*|.*flagyl.*|.*Flagyl.*|.*tinidazole.*",
"nitromidazol",

              gsub(".*nitrofur.*|.*Nitrofur.*|.*Macrobid.*", "nitrofuran",

                gsub(".*augmentin.*|.*Augmentin.*|.*amoxicillin.*|.*Amp.*|.*Amoxicillin.*",
"penicillin",

                  gsub(".*Bactrim.*", "sulfamethoxazole",

                    gsub(".*Azithromycin.*|.*azithromycin.*|.*clindamycin.*|.*mycin.*|.*Z
pack.*|.*Zithromax.*", "macrolide",

                      gsub(".*doxycycline.*", "tetracycline",


gsub(".*Ceftriaxone.*|.*cefixime.*|.*Cephalexin.*|.*cephalexin.*|.*ancef.*", "cephalosporin",

                             gsub(".*Diflucan.*|.*Terazol.*", "anti-fungal",

                               gsub(".*acetasol.*", "other",

                                 gsub(".*Bacterial Vaginosis.*|.*not documented.*", "NA",
metadata$abxtype6))))))))))



df1 = metadata
```

```
factor2 <- sqldf("select distinct sixth_antityp as 'abx_type' from df1")

factor2


#write.xlsx(metadata, "/Users/madeleinerussell/Desktop/Comstock Lab/AbxResistance/ABXR_Ranalysis/AMR_Data/2023FEB28_3mosmetadata_267_infants_MR.xlsx", quote=FALSE, rowNames=FALSE)



###making pie chart

ID <- seq.int(nrow(metadata))

first_antityp<-metadata$first_antityp

second_antityp<- metadata$second_antityp

third_antityp<- metadata$third_antityp

fourth_antityp<- metadata$fourth_antityp

fifth_antityp<- metadata$fifth_antityp


data_antfreq<-data.frame(ID, first_antityp, second_antityp, third_antityp, fourth_antityp, fifth_antityp)

data_antfreq<- melt(data_antfreq, id=c('ID'))


# table(metadata$first_antityp)

# table(metadata$second_antityp)

# table(metadata$third_antityp)

# table(metadata$fourth_antityp)

# table(metadata$fifth_antityp)

#

# as.list(metadata$first_antityp)
```

```
# na.omit(data_antfreq)
# data_antfreq %>% replace_with_na(replace = list(x = "NA"))

# install.packages("naniar")
# library(naniar)


data_antfreq2<-data_antfreq[-grep("NA", data_antfreq$value), ]
data_antfreq2<-data_antfreq2[order(data_antfreq2$value),]
table(data_antfreq2$value)

library(dplyr)
library(ggplot2)
library(ggrepel)
library(forcats)
library(scales)
library(viridis)
library(dplyr)
library(forcats)
library(hrbrthemes)


#Classification of medication exposure : medication ever used during pregnancy
#Pie chart with distribution of medications during pregnancy
a<-table(data_antfreq2$value)
```

```
b<-as.data.frame(a)

abx<-levels(as.factor(as.character(data_antfreq2$value)))


piepercent <- round(100*a/sum(a),1)

piepercent<-paste(piepercent,"%",sep="")

pie(a,labels=piepercent, main="Antimicrobial prescription distribution among pregnant women", col=rainbow(length(a)))

legend(-1.6,- 0.1, legend=abx, cex = 0.5, fill = rainbow(length(a)))


# install.packages("viridis")

# install.packages("hrbrthemes")

library(viridis)

library(dplyr)

library(forcats)

library(hrbrthemes)


df3 <- b  %>%

  group_by(`Var1`) %>% # Variable to be transformed

  count() %>%

  ungroup() %>%

  mutate(perc = b$Freq / sum(b$Freq)) %>%

  arrange(perc) %>%

  mutate(labels = scales::percent(perc))


#inside the pie

ggplot(df3, aes(x = "", y = perc, fill = Var1)) +
```

```
 geom_col() +
 geom_text(aes(x=1.6, label = labels),
         position = position_stack(vjust = 0.5)) +
 coord_polar(theta = "y") +
 scale_fill_viridis_d() +
 theme_void()


########Going away from matched pairs, back to regular programming######
######Descriptive based on Andrea's code#####

abundance3mos<-read.xlsx("2023APR18_abundance3mos_plusmoms_MR.xlsx")
presence3mos<-read.xlsx("2023APR18_presence3mos_plusmoms_MR.xlsx")
presence<-presence3mos
abundance<-abundance3mos
abundance3mos_mom<-abundance3mos[which(abundance3mos$class=="Mother"),]
abundance3mos_infant<-abundance3mos[which(abundance3mos$class=="Infant"),]

#Number of genes expressed overall
presence2<-abundance3mos[,c(4:146)]
abundance_mom<-presence2[which(abundance3mos$class=="Mother"),]
abundance_infant<-presence2[which(abundance3mos$class=="Infant"),]

overallsum<- colSums(presence2[,28:143])
#which(overallsum==0)
```

```
momsum<- colSums(abundance_mom[,28:143])
which(overallsum==0)

childsum<- colSums(presence_infant[,28:143])
which(childsum==0)

overallsum<- colSums(presence2[,28:143])
which(overallsum==0)
max(overallsum)
summary(overallsum)
summary(momsum)
summary(childsum)



###OVERALL
#Number of ARGs per individual
overallsum2<- rowSums(presence2[,28:143])
summary(overallsum2)

#Number of MGEs per individual
overallsum2<- rowSums(presence2[,1:27])
which(overallsum==0)
summary(overallsum2)
```

```
###FOR MOTHERS

#Number of ARGs per individual


momsum<- rowSums(presence2[,28:143])

summary(momsum)


#Number of MGEs per individual

momsum<- rowSums(presence2[,1:27])

which(overallsum==0)

summary(momsum)



###FOR INFANTS


#Number of ARGs per individual

childsum<- rowSums(presence_infant[,28:143])

summary(childsum)


#Number of MGEs per individual sum<-

childsum<- rowSums(presence_infant[,1:27])

summary(childsum)


#n=237 infants, n=99 moms


#####Correlation plots#####
```

```
library(reshape2)

library("corrplot")



#2023APR18_abundance3mos_plusmoms_MR.xlsx

abundance3mos<-read.xlsx("2023APR18_abundance3mos_plusmoms_MR.xlsx")

presence3mos<-read.xlsx("2023APR18_presence3mos_plusmoms_MR.xlsx")

write.csv(presence3mos, "/Users/madeleinerussell/Desktop/Comstock Lab/AbxResistance/ABXR_Ranalysis/AMR_Data/2023APR20_presence3mos_plusmoms_MR.csv", row.names = FALSE)

write.csv(abundance3mos, "/Users/madeleinerussell/Desktop/Comstock Lab/AbxResistance/ABXR_Ranalysis/AMR_Data/2023APR20_abundance3mos_plusmoms_MR.csv", row.names = FALSE)



#co-occurance analysis in women's samples

binary<-read.csv("2023APR20_presence3mos_plusmoms_MR.csv", header=TRUE, row.names=1)

str(binary)


binary2<-binary[,3:145]

binary3<-binary2[which(binary$class=="Mother"),]

str(binary3) #we have data from 99 women


#To have gene presence in at least 25% of samples the gene has to be present in at least 24 samples

library(tibble)
```

```
#this next little chunk was to get the names of the most abundant present number of genes

binary2<-binary[,3:145]

minimum<-colSums(binary2)

summary(minimum)

a<-data.frame(minimum)

df2 <- tibble::rownames_to_column(a, "Genename")

summary(df2)

df2 %>% group_by(Genename) %>% slice_max(n = 1, minimum)


#write.xlsx(df2, "/Users/madeleinerussell/Desktop/Comstock Lab/AbxResistance/ABXR_Ranalysis/AMR_Data/2022APR29_data/2023APR29_3mosCorr_allinfants_geneslist_MR.xlsx", quote=FALSE, rowNames=FALSE)


#repeat but just for moms

binary2<-binary[,3:145]

binary3<-binary2[which(binary$class=="Mother"),]

minimum<-colSums(binary3)

summary(minimum)

a<-data.frame(minimum)

df4 <- tibble::rownames_to_column(a, "Genename")

summary(df4)

df4 %>% group_by(Genename) %>% slice_max(n = 1, minimum)


#write.xlsx(df4, "/Users/madeleinerussell/Desktop/Comstock Lab/AbxResistance/ABXR_Ranalysis/AMR_Data/2022APR29_data/2023APR29_3mosCorr_moms_geneslist_MR.xlsx", quote=FALSE, rowNames=FALSE)
```

```
#to get just infants

binary4<-binary2[which(binary$class=="Infant"),]

minimum<-colSums(binary4)

summary(minimum)

a<-data.frame(minimum)

df5 <- tibble::rownames_to_column(a, "Genename")

summary(df5)

df5 %>% group_by(Genename) %>% slice_max(n = 1, minimum)

# write.xlsx(df5, "/Users/madeleinerussell/Desktop/Comstock Lab/AbxResistance/ABXR_Ranalysis/AMR_Data/2022APR29_data/2023APR29_3mosCorr_infants_geneslist_MR.xlsx", quote=FALSE, rowNames=FALSE)

# #to get gene names/functions, use gene names list mentioned below


#continue w corr table


minimum<-colSums(binary3)

str(minimum)


summary(minimum)


vars20<-minimum[which(minimum>=49)]

str(vars20)

a<-data.frame(vars20)

df2 <- tibble::rownames_to_column(a, "Genename")


summary(df2)
```

```
#To select those genes present in 50% of the samples

resistome<-read.csv("2023APR20_presence3mos_plusmoms_MR.csv", header=TRUE, row.names=1)

resistome [is.na(resistome)]<- 0

str(resistome)

log_resistome<-log(resistome[3:145]+0.00000000000001)



resistome_mom<-log_resistome[which(resistome$class=="Mother"),]

str(resistome_mom)


df1<-resistome_mom

#basically, making a list of only genes identified in over 50% of mom samples

list.df2 <- dput(as.character(df2$Genename))

#list of genes names

df3<-df1 %>% select(one_of(list.df2)) #only selecting genes from df1 that are in top 20 abundance

resistome_mom<-df3

#resistome data


#correlation matrix between OTU vs GENE

correlation<- cor(resistome_mom, method="spearman")

correlation[lower.tri(correlation)]<- NA


#Selecting correlation values more than 0.9
```

```
correlation2<- melt(correlation)

str(correlation2)



dataframe<-data.frame(correlation2)

busqueda<-dataframe[which(dataframe$value>0.8),]

str(busqueda)


#Real name of genes

realname<-read.csv("gene_realname.csv")

realname$gene<-paste("X", realname$Assay, sep="")

correlationGephi<-merge(realname, busqueda, by.x="gene", by.y="Var1")

str(correlationGephi)


correlationGephi2<-merge(correlationGephi, realname, by.x="Var2", by.y="gene")

str(correlationGephi2)

myvars<-c("Name.x",
"Name.y","value","ANDREA.CLASSIFICATION.x","ANDREA.CLASSIFICATION.y")


CorrelationTable<-correlationGephi2[myvars]

CorrelationTable<-CorrelationTable[which(CorrelationTable$value<1),]

str(CorrelationTable)


#renaming variables to match for gephi--all of this is based off of Andrea's file that she
made for gephi
```

```
CorrelationTable$class<-
paste(CorrelationTable$ANDREA.CLASSIFICATION.x,CorrelationTable$ANDREA.CLASSIFI
CATION.y,sep="-")

CorrelationTable2<-CorrelationTable

names(CorrelationTable2)[names(CorrelationTable2) == "value"] <- "Weight" #rename
column

names(CorrelationTable2)[names(CorrelationTable2) == "Name.x"] <- "Source" #rename
column

names(CorrelationTable2)[names(CorrelationTable2) == "Name.y"] <- "Target" #rename
column


#make the rownames the ID column

CorrelationTable2 <- tibble::rownames_to_column(CorrelationTable2, "ID")


#add columns that are missing

CorrelationTable3 <- CorrelationTable2 %>%

 add_column(Label = NA,

        Interval = NA,

        Type = "Directed")

#reorder columns

col_order <- c("Source", "Target", "Type",

         "ID", "Label", "Interval", "Weight", "ANDREA.CLASSIFICATION.x",
"ANDREA.CLASSIFICATION.y", "class")

CorrelationTable4 <- CorrelationTable3[, col_order]


write.xlsx(CorrelationTable4, "/Users/madeleinerussell/Desktop/Comstock
Lab/AbxResistance/ABXR_Ranalysis/AMR_Data/2023APR28_3mosCorr_moms_MR.xlsx",
quote=FALSE, rowNames=FALSE)
```

```
mom.nodes.table<-CorrelationTable4$Source

write.xlsx(mom.nodes.table, "/Users/madeleinerussell/Desktop/Comstock Lab/AbxResistance/ABXR_Ranalysis/AMR_Data/2023APR28_moms_nodes_MR.xlsx", quote=FALSE, rowNames=FALSE)



####Co-ocurrence analysis in infant samples###

#####To select those genes present in 50% of the samples


binary2<-binary[,3:145]

binary3<-binary2[which(binary$class=="Infant"),]

str(binary3) #we have data from 237 infants


#To have gene presence in at least 50% of samples the gene has to be present in at least 118 infant samples


minimum<-colSums (binary3)

str(minimum)

vars20<-minimum[which(minimum>=118)]

str(vars20)

a<-data.frame(vars20)

df2 <- tibble::rownames_to_column(a, "Genename")


#To select those genes present in 25% of the samples
```

```
resistome<-read.csv("2023APR20_abundance3mos_plusmoms_MR.csv", header=TRUE,
row.names=1)

resistome [is.na(resistome)]<- 0

str(resistome)

log_resistome<-log(resistome[3:145]+0.00000000000001)


resistome_child<-log_resistome[which(resistome$class=="Infant"),]

str(resistome_child)


df1<-resistome_child

list.df2 <- dput(as.character(df2$Genename))

df3<-df1 %>% select(one_of(list.df2))

resistome_child<-df3


resistome_child #resistome data


#correlation matrix between OTU vs GENE

correlation<- cor(resistome_child, method="spearman")

correlation[lower.tri(correlation)]<- NA


#Selecting correlation values more than 0.8


correlation2<- melt(correlation)

str(correlation2)


dataframe<-data.frame(correlation2)
```

```
busqueda<-dataframe[which(dataframe$value>0.8),]

str(busqueda)


#Real name of genes

realname<-read.csv("gene_realname.csv")

realname$gene<-paste("X", realname$Assay, sep="")

correlationGephi<-merge(realname, busqueda, by.x="gene", by.y="Var1")

str(correlationGephi)


correlationGephi2<-merge(correlationGephi, realname, by.x="Var2", by.y="gene")

str(correlationGephi2)

myvars<-c("Name.x",
"Name.y","value","ANDREA.CLASSIFICATION.x","ANDREA.CLASSIFICATION.y")


CorrelationTable<-correlationGephi2[myvars]

CorrelationTable<-CorrelationTable[which(CorrelationTable$value<1),]

str(CorrelationTable)


#write.csv(CorrelationTable, "3mos_CorrelationTable_infant_RA.csv")


#combining two columns (x & y) to get classification

CorrelationTable$class<-
paste(CorrelationTable$ANDREA.CLASSIFICATION.x,CorrelationTable$ANDREA.CLASSIFI
CATION.y,sep="-")


#renaming variables to match for gephi--all of this is based off of Andrea's file

CorrelationTable2<-CorrelationTable
```

```
names(CorrelationTable2)[names(CorrelationTable2) == "value"] <- "Weight"

names(CorrelationTable2)[names(CorrelationTable2) == "Name.x"] <- "Source"

names(CorrelationTable2)[names(CorrelationTable2) == "Name.y"] <- "Target"


#make the rownames the ID column

CorrelationTable2 <- tibble::rownames_to_column(CorrelationTable2, "ID")


CorrelationTable3 <- CorrelationTable2 %>%

  add_column(Label = NA,

          Interval = NA,

          Type = "Directed")

#reorder columns

col_order <- c("Source", "Target", "Type",

           "ID", "Label", "Interval", "Weight", "ANDREA.CLASSIFICATION.x",
"ANDREA.CLASSIFICATION.y", "class")

CorrelationTable4 <- CorrelationTable3[, col_order]


write.xlsx(CorrelationTable4, "/Users/madeleinerussell/Desktop/Comstock
Lab/AbxResistance/ABXR_Ranalysis/AMR_Data/2023APR28_3mosCorr_infants_only_MR.xl
sx", quote=FALSE, rowNames=FALSE)



infant.nodes.table<-as.data.frame(CorrelationTable4$Source)

names(infant.nodes.table)[names(infant.nodes.table) == "CorrelationTable4$Source"] <-
"ID"

infant.nodes.table$Label<-infant.nodes.table$ID
```

```
write.xlsx(infant.nodes.table, "/Users/madeleinerussell/Desktop/Comstock Lab/AbxResistance/ABXR_Ranalysis/AMR_Data/2023APR28_infant_nodes_table_MR.xlsx", quote=FALSE, rowNames=FALSE)



######present in all samples


binary2<-binary[,3:145]

binary3<-binary2 #no class assigned

str(binary3) #we have data from 334 infants + moms


#To have gene presence in at least 50% of samples the gene has to be present in at least 167.5 samples


minimum<-colSums (binary3)

str(minimum)

vars20<-minimum[which(minimum>=168)]

str(vars20)


#To select those genes present in 25% of the samples

resistome<-read.csv("2023APR20_presence3mos_plusmoms_MR.csv", header=TRUE, row.names=1)

resistome [is.na(resistome)]<- 0

str(resistome)

log_resistome<-log(resistome[3:145]+0.00000000000001)


str(resistome)
```

```
#correlation matrix between OTU vs GENE

correlation<- cor(log_resistome, method="spearman")

correlation[lower.tri(correlation)]<- NA


#Selecting correlation values more than 0.9


correlation2<- melt(correlation)

str(correlation2)


dataframe<-data.frame(correlation2)

busqueda<-dataframe[which(dataframe$value>0.6),]

str(busqueda)


#Real name of genes

realname<-read.csv("gene_realname.csv")

realname$gene<-paste("X", realname$Assay, sep="")

correlationGephi<-merge(realname, busqueda, by.x="gene", by.y="Var1")

str(correlationGephi)


correlationGephi2<-merge(correlationGephi, realname, by.x="Var2", by.y="gene")

str(correlationGephi2)

myvars<-c("Name.x",
"Name.y","value","ANDREA.CLASSIFICATION.x","ANDREA.CLASSIFICATION.y")


CorrelationTable<-correlationGephi2[myvars]
```

```
CorrelationTable<-CorrelationTable[which(CorrelationTable$value<1),]

str(CorrelationTable)


write.csv(CorrelationTable, "3mos_CorrelationTable_all_RA.csv")


#combining two columns (x & y) to get classification

CorrelationTable$class<-
paste(CorrelationTable$ANDREA.CLASSIFICATION.x,CorrelationTable$ANDREA.CLASSIFI
CATION.y,sep="-")


#renaming variables to match for gephi--all of this is based off of Andrea's file

CorrelationTable2<-CorrelationTable

names(CorrelationTable2)[names(CorrelationTable2) == "value"] <- "Weight"

names(CorrelationTable2)[names(CorrelationTable2) == "Name.x"] <- "Source"

names(CorrelationTable2)[names(CorrelationTable2) == "Name.y"] <- "Target"


#make the rownames the ID column

CorrelationTable2 <- tibble::rownames_to_column(CorrelationTable2, "ID")


CorrelationTable3 <- CorrelationTable2 %>%

  add_column(Label = NA,

         Interval = NA,

         Type = "Directed")

#reorder columns

col_order <- c("Source", "Target", "Type",

          "ID", "Label", "Interval", "Weight", "ANDREA.CLASSIFICATION.x",
"ANDREA.CLASSIFICATION.y", "class")
```

```
CorrelationTable4 <- CorrelationTable3[, col_order]

write.xlsx(CorrelationTable4, "/Users/madeleinerussell/Desktop/Comstock Lab/AbxResistance/ABXR_Ranalysis/AMR_Data/2023APR28_3mosCorr_infants_and_moms_MR.xlsx", quote=FALSE, rowNames=FALSE)

all.nodes.table<-as.data.frame(CorrelationTable4$Source)
names(all.nodes.table)[names(all.nodes.table) == "CorrelationTable4$Source"] <- "ID"
all.nodes.table$Label<-all.nodes.table$ID

write.xlsx(all.nodes.table, "/Users/madeleinerussell/Desktop/Comstock Lab/AbxResistance/ABXR_Ranalysis/AMR_Data/2023APR28_all_nodes_table_MR.xlsx", quote=FALSE, rowNames=FALSE)

#####MetaData Analysis####
#install.packages("gplots")
library("gplots")
library("ggpubr")
library(RColorBrewer)
library(ggplot2)
#install.packages("viridis")
library(viridis)
library(dplyr)
library(forcats)
library(hrbrthemes)
```

```
metadata.267infants<-read.xlsx("2023FEB28_3mosmetadata_267_infants_MR.xlsx")


table(metadata.267infants$location)


#####Infections yes or no####

names(metadata.267infants)[names(metadata.267infants) ==
'number_infections_treated'] <- 'any_infections'

table(metadata.267infants$any_infections)

table1<-as.table(table(metadata.267infants$location,
metadata.267infants$any_infections))

table1

prop.table(table1,1)


#kind of a pretty plot

dt <- as.table(as.matrix(table1))


balloonplot(t(dt), main ="Infections", xlab ="", ylab="",

        label = FALSE, show.margins = FALSE)




chisq.test(table1)

#p-value = 5.584e-16


FUN = function(i,j){

  chisq.test(matrix(c(table1[i,1], table1[i,2],
```

```
          table1[j,1], table1[j,2]),
         nrow=2,
         byrow=TRUE))$ p.value
}

pairwise.table(FUN,
       rownames(table1),
       p.adjust.method="none")

table(metadata.267infants$location)

metadata.267infants %>%
  filter(!is.na(metadata.267infants$any_infections)) %>%
  ggplot(metadata.267infants, mapping= aes(x= location, fill = any_infections, na.rm =
TRUE))+
  geom_bar(position='stack', na.rm = TRUE) +
  scale_fill_viridis(discrete = T) +
  ggtitle("Any Infections") +
  theme_ipsum() +
  xlab("")

metadata.267infants %>%
  filter(!is.na(metadata.267infants$any_infections)) %>%
  ggplot(metadata.267infants, mapping= aes(x= location, fill = any_infections, na.rm =
TRUE))+
  geom_bar(position='dodge') +
  scale_fill_viridis(discrete = T) +
```

```
  ggtitle("Any Infections") +
  theme_ipsum() +
  xlab("")

metadata.267infants %>%
  filter(!is.na(metadata.267infants$any_infections)) %>%
  ggplot(metadata.267infants, mapping= aes(x= location, fill = any_infections, na.rm =
TRUE)) +
  geom_bar(position='fill') +
  scale_fill_viridis(discrete = T) +
  ggtitle("Any Infections") +
  theme_ipsum() +
  xlab("")

#####Number_of_infections####
metadata.267infants$total_number_inf_recorded<-
gsub('44595','2',metadata$total_number_inf_recorded)

table2 =
as.table(table(metadata.267infants$location,metadata.267infants$total_number_inf_reco
rded))
table2
```

```
prop.table(table2,1)

#(using a 2 would have given you proportion by column)


dt <- as.table(as.matrix(table2))

balloonplot(t(dt), main ="Infections", xlab ="", ylab="",

        label = FALSE, show.margins = FALSE)

chisq.test(table2)


FUN = function(i,j){

  chisq.test(matrix(c(table2[i,1], table2[i,2],

                table2[j,1], table2[j,2]),

               nrow=2,

               byrow=TRUE))$ p.value

}


pairwise.table(FUN,

          rownames(table2),

          p.adjust.method="none")


metadata.267infants$total_number_inf_recorded<-
as.character(metadata.267infants$total_number_inf_recorded)



metadata.267infants %>%

  filter(!is.na(metadata.267infants$total_number_inf_recorded)) %>%

  ggplot(metadata.267infants, mapping= aes(x= location, fill = total_number_inf_recorded,
na.rm = TRUE)) +
```

```
  geom_bar(position='fill', na.rm = TRUE) +

  scale_fill_viridis(discrete = T) +

  ggtitle("Any Infections") +

  theme_ipsum() +

  xlab("")



#####Location*Women treated with ABX#####

#####Has patient taken antibiotics from 30 days before

#####pregnancy up to but excluding the time of delivery?

table4 = as.table(table(metadata.267infants$location,
metadata.267infants$` abx_w/in_30days` ))

table4

prop.table(table4,1)

#(using a 2 would have given you proportion by column)


dt <- as.table(as.matrix(table4))

balloonplot(t(dt), main ="Infections", xlab ="", ylab="",

        label = FALSE, show.margins = FALSE)

chisq.test(table4)


FUN = function(i,j){

  chisq.test(matrix(c(table4[i,1], table4[i,2],

                table4[j,1], table4[j,2]),

               nrow=2,

               byrow=TRUE))$ p.value
```

```
}

pairwise.table(FUN,
        rownames(table4),
        p.adjust.method="none")

metadata.267infants %>%
  filter(!is.na(metadata.267infants$`abx_w/in_30days`)) %>%
  ggplot(metadata.267infants, mapping= aes(x= location, fill = `abx_w/in_30days`))+
  geom_bar(position='stack') +
  scale_fill_viridis(discrete = T) +
  ggtitle("Antibiotics during Pregnancy") +
  theme_ipsum() +
  xlab("")

metadata.267infants %>%
  filter(!is.na(metadata.267infants$`abx_w/in_30days`)) %>%
  ggplot(MetaData, mapping= aes(x= location, fill = `abx_w/in_30days`))+
  geom_bar(position='fill') +
  scale_fill_viridis(discrete = T) +
  ggtitle("Antibiotics during Pregnancy") +
  theme_ipsum() +
  xlab("")


#####Total Inf Treated####
```

```
metadata.267infants<-meta

metadata.267infants$total_inf_treated<-gsub('4495|99|-
8','0',metadata.267infants$total_inf_treated)

metadata.267infants$total_inf_treated<-gsub('2 -
antifungal','2',metadata.267infants$total_inf_treated)



table5 = as.table(table(metadata.267infants$location,
metadata.267infants$total_inf_treated))

table5

prop.table(table5,1)


dt <- as.table(as.matrix(table5))

balloonplot(t(dt), main ="Infections Treated", xlab ="", ylab="",

      label = FALSE, show.margins = FALSE)

chisq.test(table5)


FUN = function(i,j){

 chisq.test(matrix(c(table5[i,1], table5[i,2],

           table5[j,1], table5[j,2]),

          nrow=2,

          byrow=TRUE))$ p.value

}


pairwise.table(FUN,

       rownames(table5),

       p.adjust.method="none")
```

```
metadata.267infants %>%
  filter(!is.na(metadata.267infants$total_inf_treated)) %>%
  ggplot(metadata.267infants, mapping= aes(x= total_inf_treated, fill = total_inf_treated))+
  geom_bar(position='stack') +
  scale_fill_viridis(discrete = T) +
  ggtitle("Total Infections Treated") +
  theme_ipsum() +
  xlab("")

metadata.267infants %>%
  filter(!is.na(metadata.267infants$total_inf_treated)) %>%
  ggplot(metadata.267infants, mapping= aes(x= Timepoint.x, fill = total_inf_treated))+
  geom_bar(position='fill') +
  scale_fill_viridis(discrete = T) +
  ggtitle("Total Infections Treated") +
  theme_ipsum() +
  xlab("")

#####Total Inf NOT Treated####
metadata.267infants$total_inf_not_treated<-gsub('99|-8','0',metadata.267infants$total_inf_not_treated)

table3 = as.table(table(metadata.267infants$location,metadata.267infants$total_inf_not_treated))
table3
prop.table(table3,1)
```

```
#(using a 2 would have given you proportion by column)


dt <- as.table(as.matrix(table3))

balloonplot(t(dt), main ="Infections", xlab ="", ylab="",

        label = FALSE, show.margins = FALSE)

chisq.test(table3)


FUN = function(i,j){

  chisq.test(matrix(c(table3[i,1], table3[i,2],

                 table3[j,1], table3[j,2]),

               nrow=2,

               byrow=TRUE))$ p.value

}


pairwise.table(FUN,

          rownames(table3),

          p.adjust.method="none")


##same analysis but remove unknowns

remove.unknowns<-metadata.267infants[-grep("Unknown",
metadata.267infants$Group_B_pos),]

remove.unknowns


table3 = as.table(table(remove.unknowns$location,remove.unknowns$Group_B_pos))

table3

prop.table(table3,1)
```

```
dt <- as.table(as.matrix(table3))

balloonplot(t(dt), main ="Infections", xlab ="", ylab="",

        label = FALSE, show.margins = FALSE)

chisq.test(table3)


FUN = function(i,j){

  chisq.test(matrix(c(table3[i,1], table3[i,2],

              table3[j,1], table3[j,2]),

             nrow=2,

             byrow=TRUE))$ p.value

}


pairwise.table(FUN,

          rownames(table3),

          p.adjust.method="none")




#####Group B Strep Positive####

table3 =
as.table(table(metadata.267infants$location,metadata.267infants$Group_B_pos))

table3

prop.table(table3,1)

#(using a 2 would have given you proportion by column)
```

```
dt <- as.table(as.matrix(table3))

balloonplot(t(dt), main ="Infections", xlab ="", ylab="",

        label = FALSE, show.margins = FALSE)

chisq.test(table3)


FUN = function(i,j){

  chisq.test(matrix(c(table3[i,1], table3[i,2],

                 table3[j,1], table3[j,2]),

                nrow=2,

                byrow=TRUE))$ p.value

}


pairwise.table(FUN,

          rownames(table3),

          p.adjust.method="none")


##same analysis but remove unknowns

remove.unknowns<-metadata.267infants[-grep("Unknown",
metadata.267infants$Group_B_pos),]

remove.unknowns


table3 = as.table(table(remove.unknowns$location,remove.unknowns$Group_B_pos))

table3

prop.table(table3,1)
```

```
dt <- as.table(as.matrix(table3))

balloonplot(t(dt), main ="Infections", xlab ="", ylab="",

        label = FALSE, show.margins = FALSE)

chisq.test(table3)


FUN = function(i,j){

  chisq.test(matrix(c(table3[i,1], table3[i,2],

              table3[j,1], table3[j,2]),

            nrow=2,

            byrow=TRUE))$ p.value

}


pairwise.table(FUN,

         rownames(table3),

         p.adjust.method="none")


ggplot(data=subset(remove.unknowns, !is.na(Group_B_pos)), aes(x= location, fill = Group_B_pos))+

  geom_bar(position='fill') +

  scale_fill_viridis(discrete = T) +

  ggtitle("") +

  theme_ipsum() +

  xlab("")


#####women with group B strep given ABX#####

remove.strepneg<-remove.unknowns[-grep("No",remove.unknowns$Group_B_pos),]
```

```
remove.strepneg


table3 = as.table(table(remove.strepneg$location,remove.strepneg$abx_group_b))
table3
prop.table(table3,1)

dt <- as.table(as.matrix(table3))
balloonplot(t(dt), main ="Infections", xlab ="", ylab="",
      label = FALSE, show.margins = FALSE)
chisq.test(table3)

FUN = function(i,j){
 chisq.test(matrix(c(table3[i,1], table3[i,2],
           table3[j,1], table3[j,2]),
          nrow=2,
          byrow=TRUE))$ p.value
}

pairwise.table(FUN,
       rownames(table3),
       p.adjust.method="none")



table9 = as.table(table(metadata.267infants$location, metadata.267infants$first_antityp))
```

```
table9
prop.table(table9,1)


dt <- as.table(as.matrix(table9))
balloonplot(t(dt), main ="Infections", xlab ="", ylab="",
        label = FALSE, show.margins = FALSE)
chisq.test(table9)


FUN = function(i,j){
  chisq.test(matrix(c(table9[i,1], table9[i,2],
                      table9[j,1], table9[j,2]),
                    nrow=2,
                    byrow=TRUE))$ p.value
}

pairwise.table(FUN,
           rownames(table9),
           p.adjust.method="none")
abx<-remove.abx1none$ABX_Type1
local<-remove.abx1none$Setting


tbl2 <- table(local,abx)
tbl2
```

```
df <- as.data.frame(tbl2)

head(df)

df


#percent going to each

ggplot(remove.abx1none, aes(x= Setting, fill = ABX_Type1))+
  geom_bar(position='fill') +
  scale_fill_viridis(discrete = T) +
  ggtitle("") +
  theme_ipsum() +
  xlab("")

ggplot(remove.abx1none, aes(x= Setting, fill = ABX_Type1))+
  geom_bar(position='stack') +
  scale_fill_viridis(discrete = T) +
  ggtitle("") +
  theme_ipsum() +
  xlab("")

ggplot(remove.abx1none, aes(x= Setting, fill = ABX_Type1))+
  geom_bar(position='dodge') +
  scale_fill_viridis(discrete = T) +
  ggtitle("") +
  theme_ipsum() +
  xlab("")
```

```
########

########

#######adding in 3 mos survery, etc. #######

mos3survey<-read.xlsx("2022Feb08data4Madi_set1_3mos_survey.xlsx")

mos3survey

mos3survey$ID<-gsub('P8',"", mos3survey$ID)

mos3survey$ID


birthcert<-read.xlsx("2022JAN26_data4Madi_set2_bc_info.xlsx")

birthcert

birthcert$ID<-gsub('P8',"",birthcert$ID)

birthcert$ID


metadata.267infants$ID<-gsub('P8',"",metadata.267infants$ID)

metadata.267infants$ID


temp<-merge(metadata.267infants, birthcert,by="ID")

temp


temp2<-merge(temp, mos3survey,by="ID")

temp2


metadata.203infants<-temp2

AllData<-metadata.203infants
```

```
#######analyzing with

#make age a factor

a<-as.factor(AllData$MOM_AGE)

a

#make it a numeric factor

mom_age<-as.numeric(as.character(a))

mom_age

is.factor(mom_age)

age<-as.vector(mom_age)


location<-as.factor(AllData$location)

is.factor(location)

setting<-as.vector(location)


table(AllData$location, mom_age)


group_by(AllData, location) %>%

 summarise(

  count = n(),

  mean = mean(mom_age, na.rm = TRUE),

  sd = sd(mom_age, na.rm = TRUE)

 )


#boxplot

ggboxplot(AllData, x = "location", y = "mom_age",
```

```
        color = "location",
        palette = c("#00AFBB", "#E7B800", "#FC4E07", "#00FF00", "#663300", "#DB3FBC"),
        add = "mean_sd")

summary(mom_age)

table11= as.table(table(AllData$location, AllData$MOM_AGE))
table11
prop.table(table11,1)

dt <- as.table(as.matrix(table11))
balloonplot(t(dt), main ="Age", xlab ="", ylab="",
         label = FALSE, show.margins = FALSE)
chisq.test(table11)
#p-value = 0.02455

FUN = function(i,j){
  chisq.test(matrix(c(table11[i,1], table11[i,2],
                    table11[j,1], table11[j,2]),
                  nrow=2,
                  byrow=TRUE))$ p.value
}

pairwise.table(FUN,
            rownames(table11),
            p.adjust.method="none")
```

```
AllData$location

bcdata

# AllData<-read.xlsx("2021MAR15_ABX_Complete_MetaData_MR.xlsx")
# AllData$BMI
# a<-as.factor(AllData$BMI)
# a
# #make it a numeric factor
# BMI<-as.numeric(as.character(a))
# BMI


bcdata$BRIDGEMOMRACE
AllData<-bcdata
#1=white
#2=black
#5=chinese
#8=Korean
#10=Asian
#15=Other Race
#22=Black Multiple Race
#24=Asian/Pacific Islander
#99=unknown
AllData$Race<-as.factor(ifelse(AllData$BRIDGEMOMRACE== '1', 'white',
                        ifelse(AllData$BRIDGEMOMRACE== '2', 'black',
```

```
                    ifelse(AllData$BRIDGEMOMRACE== '24', 'asian',
                        ifelse(AllData$BRIDGEMOMRACE== '10', 'asian',
                            ifelse(AllData$BRIDGEMOMRACE== '22', 'black',
                                ifelse(AllData$BRIDGEMOMRACE== '8', 'asian', 'other')))))))




AllData$Race2<-as.factor(ifelse(AllData$Race== 'white', 'white', 'non-white'))


table(AllData$Race)


AllData_omit<-AllData

AllData_omit<-AllData_omit %>% drop_na(Race)



table20 = as.table(table(AllData$location,AllData$Race))

table20

prop.table(table20,1)


dt <- as.table(as.matrix(table20))

balloonplot(t(dt), main ="race", xlab ="", ylab="",

      label = FALSE, show.margins = FALSE)

chisq.test(table20)

#p-value < 2.2e-16


FUN = function(i,j){
```

```
  chisq.test(matrix(c(table20[i,1], table20[i,2],
                table20[j,1], table20[j,2]),
               nrow=4,
               byrow=TRUE))$ p.value
}

pairwise.table(FUN,
          rownames(table20),
          p.adjust.method="none")

ggplot(data=subset(AllData, !is.na(Race)), aes(x= location, fill = Race, rm.NA = TRUE))+
  geom_bar(position='fill') +
  scale_fill_viridis(discrete = T) +
  ggtitle("") +
  theme_ipsum() +
  xlab("")

#just 2 categories
AllData$Race2<-as.factor(ifelse(AllData$Race== 'white', 'white', 'non-white'))
table(AllData$Race2)

#overall statistics
tableR = table(AllData$Race)
tableR
prop.table(tableR)
```

```
###all races all locations

table20 = as.table(table(AllData$location,AllData$Race))

table20

prop.table(table20,1)

#(using a 2 would have given you proportion by column)


dt <- as.table(as.matrix(table20))

balloonplot(t(dt), main ="race", xlab ="", ylab="",

        label = FALSE, show.margins = FALSE)

chisq.test(table20)


#just w and nw

table21 = as.table(table(AllData$location,AllData$Race2))

table21

prop.table(table21,1)


dt <- as.table(as.matrix(table21))

balloonplot(t(dt), main ="Infections", xlab ="", ylab="",

        label = FALSE, show.margins = FALSE)

chisq.test(table21)


FUN = function(i,j){

  chisq.test(matrix(c(table21[i,1], table21[i,2],

                      table21[j,1], table21[j,2]),

                    nrow=2,
```

```
            byrow=TRUE))$ p.value
}

pairwise.table(FUN,
        rownames(table21),
        p.adjust.method="none")

ggplot(data=subset(AllData, !is.na(Race2)), aes(x= location, fill = Race2, rm.NA = TRUE))+
  geom_bar(position='fill') +
  scale_fill_viridis(discrete = T) +
  ggtitle("") +
  theme_ipsum() +
  xlab("")

######wks gestation######
AllData$ESTWKSGEST.x
#make age a factor
a<-as.factor(AllData$ESTWKSGEST.x)
a
#make it a numeric factor
weeks_gest<-as.numeric(as.character(a))
weeks_gest
is.factor(weeks_gest)
is.character(weeks_gest)

location<-as.factor(AllData$location)
```

```
is.factor(location)




group_by(AllData, location) %>%
  summarise(
    count = n(),
    mean = mean(weeks_gest, na.rm = TRUE),
    sd = sd(weeks_gest, na.rm = TRUE)
  )

#boxplot
ggboxplot(AllData, x = "location", y = "weeks_gest",
          color = "location",
          palette = c("#00AFBB", "#E7B800", "#FC4E07", "#00FF00","#663300", "#DB3FBC"))

summary(weeks_gest)

tapply(weeks_gest, location, summary)

###baby weight
AllData$baby1weight

a<-as.factor(AllData$baby1weight)
a
#make it a numeric factor
```

```
baby_wt<-as.numeric(as.character(a))

baby_wt


location<-as.factor(AllData$location)

is.factor(location)


group_by(AllData, location) %>%

  summarise(

    count = n(),

    mean = mean(baby_wt, na.rm = TRUE),

    sd = sd(baby_wt, na.rm = TRUE)

  )




summary(baby_wt)


ggstripchart(AllData, x = "Setting", y = "baby_wt",

          color = "Setting",

          palette = c("#00AFBB", "#E7B800", "#FC4E07", "#00FF00"),

          add = "mean_sd")


tapply(baby_wt, location, summary)




###mode of delivery

AllData$MOD<-as.factor(ifelse(AllData$MD_FINAL_ROUTE== '1','vaginal',
```

```
            ifelse(AllData$MD_FINAL_ROUTE== '2', 'vaginal',
               ifelse(AllData$MD_FINAL_ROUTE== '3', 'vaginal',
                  ifelse(AllData$MD_FINAL_ROUTE== '4', 'cesarean', 'other')))))
AllData$MOD

##########BMI######

height<-AllData$Height
height
weight<-AllData$PRE_PREG_WT
weight

AllData$PRE_PREG_WT<-gsub('999',"",AllData$PRE_PREG_WT)

a<-as.factor(AllData$Height)
a
b<-as.factor(AllData$PRE_PREG_WT)
b
#make it a numeric factor
height<-as.numeric(as.character(a))
height<-height/100
height

weight<-as.numeric(as.character(b))
weight<-weight/2.205
```

```
BMI<-weight/(height*height)

AllData$BMI<-BMI

a<-as.factor(AllData$BMI)
BMI<-as.numeric(as.character(a))

#BMI
table21 = as.table(table(AllData$location,AllData$BMI))
table21
prop.table(table21,1)

dt <- as.table(as.matrix(table21))
balloonplot(t(dt), main ="Infections", xlab ="", ylab="",
        label = FALSE, show.margins = FALSE)
chisq.test(table21)



FUN = function(i,j){
  chisq.test(matrix(c(table21[i,1], table21[i,2],
                      table21[j,1], table21[j,2]),
                    nrow=2,
                    byrow=TRUE))$ p.value
}

pairwise.table(FUN,
```

```
            rownames(table21),
            p.adjust.method="none")



AllData_omit<-AllData
AllData_omit<-AllData_omit %>% drop_na(BMI)
a<-as.factor(AllData_omit$BMI)
BMI<-as.numeric(as.character(a))



AllData$BMI
group_by(AllData, location) %>%
  summarise(
    count = n(),
    mean = mean(BMI, na.rm = TRUE),
    sd = sd(BMI, na.rm = TRUE)
  )

ggboxplot(AllData, x = "location", y = "BMI",
          color = "location",
          palette = c("#00AFBB", "#E7B800", "#FC4E07", "#00FF00","#663300", "#DB3FBC"))
ggstripchart(AllData, x = "location", y = "BMI",
             color = "location",
             palette = c("#00AFBB", "#E7B800", "#FC4E07", "#00FF00","#663300", "#DB3FBC"),
             add = "mean_sd")
```

```
summary(BMI)

tapply(weeks_gest, location, summary)




####All data-- antibiotic

# na.omit(data_antfreq)
# data_antfreq %>% replace_with_na(replace = list(x = "NA"))

# install.packages("naniar")
# library(naniar)

library(tidyr)
# DF %>% drop_na(y)

table(meta$number_infections_treated)
AllData<-meta
AllData_omit<-AllData
AllData_omit<-AllData_omit %>% drop_na(first_antityp)
table(AllData_omit$first_antityp)

table2 = as.table(table(AllData_omit$location,AllData_omit$first_antityp))
table2
prop.table(table2,1)
```

```
dt <- as.table(as.matrix(table2))
balloonplot(t(dt), main ="First Abx", xlab ="", ylab="",
        label = FALSE, show.margins = FALSE)
chisq.test(table2)
#0.01497
FUN = function(i,j){
  chisq.test(matrix(c(table1[i,1], table1[i,2],
                 table1[j,1], table1[j,2]),
                nrow=2,
                byrow=TRUE))$ p.value
}

pairwise.table(FUN,
           rownames(table2),
           p.adjust.method="none")

##messing with graphs
table(temp3$Race2)

ggplot(AllData_omit, aes(x = first_antityp, fill=first_antityp))+
  geom_bar() +
  coord_flip()+
  scale_fill_viridis(discrete = T) +
  ggtitle("") +
  theme_ipsum() +
```

```
  xlab("First Abx Type")


re_size <- function(x) {
  factor(x, levels = names(sort(table(x), decreasing = TRUE)))
}


ggplot(AllData_omit, aes(x = reorder_size(first_antityp), fill=first_antityp)) +
  geom_bar() +
  coord_flip()+
  scale_fill_viridis(discrete = T) +
  ggtitle("") +
  theme_ipsum() +
  xlab("First Abx Type")



ggplot(AllData_omit, aes(x = reorder_size(first_antityp))) +
  geom_bar(aes(y = (..count..)/sum(..count..))) +
  xlab("First Abx Type") +
  scale_y_continuous(labels = scales::percent, name = "Proportion") +
  theme(axis.text.x = element_text(angle = 45, hjust = 1))


#by hospital
ggplot(AllData_omit, aes(x = reorder_size(first_antityp), fill=first_antityp)) +
  geom_bar() +
  coord_flip() +
  scale_fill_viridis(discrete = T) +
```

```
  ggtitle("") +
  facet_wrap(~location) +
  theme_ipsum() +
  xlab("First Abx Type")

ggplot(AllData_omit, aes(x= location, fill = first_antityp))+
  geom_bar(position='fill') +
  scale_fill_viridis(discrete = T) +
  ggtitle("First abx Type") +
  theme_ipsum() +
  xlab("")

#to look at all abx used
ID <- seq.int(nrow(AllData))
first_antityp<-AllData$first_antityp
second_antityp<- AllData$second_antityp
third_antityp<- AllData$third_antityp
fourth_antityp<- AllData$fourth_antityp
fifth_antityp<- AllData$fifth_antityp

data_antfreq<-data.frame(ID, first_antityp, second_antityp, third_antityp, fourth_antityp, fifth_antityp)
data_antfreq<- melt(data_antfreq, id=c('ID'))

data_antfreq_omit<-data_antfreq
data_antfreq_omit<-data_antfreq %>% drop_na(value)
```

```
ggplot(data_antfreq_omit, aes(x = reorder_size(value), fill=value)) +
 geom_bar() +
 coord_flip()+
 scale_fill_viridis(discrete = T) +
 ggtitle("") +
 theme_ipsum() +
 xlab("All Abx Type")

ggplot(AllData_omit, aes(x = reorder_size(first_antityp))) +
 geom_bar(aes(y = (..count..)/sum(..count..))) +
 xlab("All Abx Type") +
 scale_y_continuous(labels = scales::percent, name = "Proportion") +
 theme(axis.text.x = element_text(angle = 45, hjust = 1))




#######
########
######## n = 60
##looking at how specific factors of mom influence baby

#2023APR18_abundance3mos_plusmoms_MR.xlsx
abundance3mos<-read.xlsx("2023APR18_abundance3mos_plusmoms_MR.xlsx")
presence3mos<-read.xlsx("2023APR18_presence3mos_plusmoms_MR.xlsx")
abundance<-abundance3mos
```

```
presence<-presence3mos



######I used to make it this way-- but I made them above lol so don't need to do this
# abundance.metadata<-read.xlsx("2023FEB01_AMR_3mosMetaData.xlsx")
# abundance.metadata$MARCHID<-gsub('P8',"",abundance.metadata$MARCHID)
# names(AllData)[names(AllData) == 'ID'] <- 'MARCHID'
#
# temp3<-merge(abundance.metadata, AllData,by="MARCHID")
# temp4<-left_join(abundance.metadata, presence, by="ID")
# temp5<-merge(temp4, AllData,by="MARCHID")
#
# presence.60<-temp5[,248:535]

bcdata<-read.xlsx("2023APR21_AMR_3mosBCData_plusabun.xlsx")
table(bcdata$INF_GB_STREP)
meta<-read.xlsx("2023APR17_AMR_3mosMetaData.xlsx")
table(meta$total_inf_treated)



#2023APR18_abundance3mos_plusmoms_MR.xlsx
abundance3mos<-read.xlsx("2023APR18_abundance3mos_plusmoms_MR.xlsx")
presence3mos<-read.xlsx("2023APR18_presence3mos_plusmoms_MR.xlsx")

#make matching presence
df6<-bcdata[,c(1,150:172)]
```

```
pres.bcdata<-inner_join(presence3mos, df6, by='ID')

abun.bcdata<-inner_join(abundance3mos, df6, by='ID')


mean(bcdata$GRAMS, na.rm=TRUE)

sd(bcdata$GRAMS, na.rm=TRUE)


mean(bcdata$ESTWKSGEST, na.rm=TRUE)

sd(bcdata$ESTWKSGEST, na.rm=TRUE)



sd(bcdata$MOM_AGE, na.rm=TRUE)


abundance<-abun.bcdata

presence<-pres.bcdata


presence2<- presence[,4:146]

presence2


abundance2<- abundance[,4:146]






#did this in case you skip the medium stuff above and need to make variable names

bcdata$Race<-as.factor(ifelse(bcdata$BRIDGEMOMRACE== '1', 'white',

                    ifelse(bcdata$BRIDGEMOMRACE== '2', 'black',
```

```
                    ifelse(bcdata$BRIDGEMOMRACE== '24', 'asian',
                      ifelse(bcdata$BRIDGEMOMRACE== '10', 'asian',
                        ifelse(bcdata$BRIDGEMOMRACE== '22', 'black',
                          ifelse(bcdata$BRIDGEMOMRACE== '8', 'asian', 'other')))))))

bcdata$Race
bcdata$Race2<-as.factor(ifelse(bcdata$Race== 'white', 'white', 'non-white'))
bcdata$SEX

bcdata$Race2

#make indices with columns you want to test
indices <- bcdata[,c("ID", "class","Race2")]
head(indices)

#can only do this part if using the full meta data:
#indices$firstanti<-temp3$first_antityp

#Sum of all antibiotic resistance genes found per class
#attaches the ID to the actual data set
#here she is attaching each indices to the row sums of presence/absence
#in doing so she is creating a relative abundance
indices$RichMGE<-rowSums(presence2[,1:27])
summary(indices$RichMGE)
```

```
indices$Richamino <- rowSums(presence2[,28:48])

summary(indices$Richamino)

indices$RichMDR<-rowSums(presence2[,49:69])

summary(indices$RichMDR)

indices$Richbeta<-rowSums(presence2[,70:85])

summary(indices$Richbeta)

indices$Richtetra<-rowSums(presence2[,86:98])

summary(indices$Richtetra)

indices$RichMLSB<-rowSums(presence2[,99:116])

summary(indices$RichMLSB)

indices$Richsulfo<-rowSums(presence2[,117:121])

summary(indices$Richsulfo)

indices$Richvanco<-rowSums(presence2[,122:129])

summary(indices$Richvanco)

indices$Richfluoro<-rowSums(presence2[,130:133])

summary(indices$Richfluoro)

indices$Richother<-rowSums(presence2[,134:143])

summary(indices$Richother)

indices$RichARG<-rowSums(presence2[,28:143])


#stacked box plot


table(bcdata$Race2)


head(bcdata$Race2)
```

```
boxplot<-c(indices$RichMGE,indices$Richamino,indices$RichMDR, indices$Richbeta, indices$Richtetra,  indices$RichMLSB, indices$Richsulfo, indices$Richvanco, indices$Richfluoro)

race<-rep(indices$Race2,9)

type<-rep(c("MGE","aminoglycoside","MDR","betalactamase","tetracycline","MLSB","sulfonamide","vancomycin","fluoroquinolone"),each=205)

length(boxplot)


boxplot2<-data.frame(boxplot,type,race)




ab<-ggplot(boxplot2, aes(x = type, y = boxplot, fill=race)) +ylim(0,30)+ geom_boxplot(width=0.4,position=position_dodge(0.4)) + theme_minimal() + theme(axis.text.x = element_text(angle = 90)) + labs(title = "", y = "Richness", x = "") + stat_compare_means(aes(group = race),label="p.signif") + scale_fill_manual(values=c("#E69F00", "#00AFBB", "#E7B800", "#FC4E07", "#00FF00","#00FF06"))+ theme(legend.position="top",)

ab

#ggsave("RichnessRace60All.tiff", units="in", width=7.5, height=5, dpi=300)

#


table(indices$RichARG)

length(indices$RichARG)


wilcox.test(RichARG~Race2, data=indices)

#p-value = 0.001038

wilcox.test(RichMGE~Race2, data=indices)
```

```
#p-value = 0.0002685

#Richness



indices$Race2

indices_omit<-indices %>% drop_na(Race2)

indices<-indices_omit




df3<-abundance %>%

  filter(ID %in% indices_omit$ID)

abundance<-df3


df4<-presence %>%

  filter(ID %in% indices$ID)

abundance<-df3

presence<-df4


b<-ggboxplot(data=indices_omit, x="Race2", y="RichARG", color="black",
fill="Race2",palette=c("#800080", "#A9A9A9"), ylab="Richness", xlab="", outlier.colour =
"black", outlier.shape = 1, main="Rich ARG")+ theme_classic() + guides(fill=FALSE) +
coord_cartesian(ylim = c(0, 100))+ stat_compare_means(label.x=1.3, label.y=95)

b


a<-ggboxplot(data=indices_omit, x="Race2", y="RichMGE", color="black",
fill="Race2",palette=c("#800080", "#A9A9A9"), ylab="Richness", xlab="", outlier.colour =
```

```
"black", outlier.shape = 1, main="Rich MGE")+ theme_classic() + guides(fill=FALSE) +
coord_cartesian(ylim = c(0, 40))+ stat_compare_means(label.x=1.7, label.y=35)

a


#combine these

figure <- ggarrange(a, b,labels = c("A", "B", ncol = 2, nrow = 1))

figure

#ggexport(figure, filename = "RichMGE_ARG_All.pdf")


presence2<- presence[,4:146]

presence2


abundance2<- abundance[,4:146]






#SHANNON INDEX


#shannon diversity index as default = Relative abundance

indices$ShannonMGERA<-diversity(abundance2[,1:27])

indices$ShannonaminoRA <- diversity(abundance2[,28:48])

indices$ShannonMDRRA<-diversity(abundance2[,49:69])

indices$ShannonbetaRA<-diversity(abundance2[,70:85])

indices$ShannontetraRA<-diversity(abundance2[,86:98])
```

```
indices$ShannonMLSBRA<-diversity(abundance2[,99:116])

indices$ShannonsulfoRA<-diversity(abundance2[,117:121])

indices$ShannonvancoRA<-diversity(abundance2[,122:129])

indices$ShannonfluoroRA<-diversity(abundance2[,130:133])

indices$ShannonotherRA<-diversity(abundance2[,134:143])

indices$ShannonARGRA<-diversity(abundance2[,28:143])


##shan tests

wilcox.test(ShannonMGERA~Race2, data=indices)

#p-value =  0.04706

wilcox.test(ShannonARGRA~Race2, data=indices)

#p-value:  0.5504

#beta, fluro, MDR, MGE, tetra



#shannon

d<-ggboxplot(data=indices, x="Race2", y="ShannonARGRA", color="black",
fill="Race2",palette=c("#800080", "#A9A9A9"), ylab="Shannon Diversity Index", xlab="",
outlier.colour = "black", outlier.shape = 1, main="Shannon ARG")+ theme_classic() +
guides(fill=FALSE) + coord_cartesian(ylim = c(0, 4.0))+ stat_compare_means(label.x=1.3,
label.y=3.8)

d

c<-ggboxplot(data=indices, x="Race2", y="ShannonMGERA", color="black",
fill="Race2",palette=c("#800080", "#A9A9A9"), ylab="Shannon Diversity Index", xlab="",
outlier.colour = "black", outlier.shape = 1, main="Shannon MGE")+ theme_classic() +
guides(fill=FALSE) + coord_cartesian(ylim = c(0, 4.0))+ stat_compare_means(label.x=1.3,
label.y=3.8)

c
```

```
#combine all 4

#figure <- ggarrange(a, b, c, d, labels = c("A", "B", "C","D", ncol = 2, nrow = 2))

#figure

#ggexport(figure, filename = "ShannonMGE_ARG_All.tiff")

#2023APR24_race_shan_rich_135


#stacked box plot

boxplot<-c(indices$ShannonMGERA,indices$ShannonaminoRA,indices$ShannonMDRRA,
indices$ShannonbetaRA, indices$ShannontetraRA,  indices$ShannonMLSBRA,
indices$ShannonsulfoRA, indices$ShannonvancoRA, indices$ShannonfluoroRA)

race<-rep(indices$Race2,9)

type<-
rep(c("MGE","aminoglycoside","MDR","betalactamase","tetracycline","MLSB","sulfonamide","
vancomycin","fluoroquinolone"),each=204)

length(boxplot)




boxplot2<-data.frame(boxplot,type,race)




bc<-ggplot(boxplot2, aes(x = type, y = boxplot, fill=race)) +ylim(0,5)+
geom_boxplot(width=0.4,position=position_dodge(0.4)) + theme_minimal() +
theme(axis.text.x = element_text(angle = 90)) + labs(title = "", y = "Shannon Diversity Index ",
x = "") + stat_compare_means(aes(group = race),label="p.signif") +
scale_fill_manual(values=c("#800080", "#A9A9A9"))+ theme(legend.position="top",)

bc
```

```
#ggsave("2023DEC04_race_shan_abun_abxclass_204.tiff", units="in", width=7.5, height=5, dpi=300)

#2023APR24_race_shan_abun_abxclass_135



#stacked bar plot

sumabundance<-aggregate(boxplot~type+race, boxplot2,median)

stackplot<-ggplot(data = sumabundance, aes(x = race, y=boxplot)) +
geom_bar(stat="identity", aes(fill=type))+theme_classic() +
scale_fill_brewer(palette="Paired")+ labs(title = "", y = "Shannon Index", x = "")+theme
(axis.text = element_text(size=14), axis.title=element_text(size=14))

stackplot

#ggexport(stackplot, filename = "/Users/madeleinerussell/Desktop/Comstock
Lab/AbxResistance/ABXR_Ranalysis/ShannonDiv_ALL.pdf")

#2023APR24_race_shan_abun_stackplot_135



#INVERSE SIMPSON INDEX

#Inverse Simpson diversity index = Relative abundance

indices$SimpsonMGERA<-diversity(abundance2[,1:27], index="invsimpson")

indices$SimpsonaminoRA<-diversity(abundance2[,28:48], index="invsimpson")

indices$SimpsonMDRRA<-diversity(abundance2[,49:69], index="invsimpson")

indices$SimpsonbetaRA<-diversity(abundance2[,70:85], index="invsimpson")

indices$SimpsontetraRA<-diversity(abundance2[,86:98], index="invsimpson")

indices$SimpsonMLSBRA<-diversity(abundance2[,99:116], index="invsimpson")

indices$SimpsonsulfoRA<-diversity(abundance2[,117:121], index="invsimpson")

indices$SimpsonvancoRA<-diversity(abundance2[,122:129], index="invsimpson")
```

```
indices$SimpsonfluoroRA<-diversity(abundance2[,130:133], index="invsimpson")

indices$SimpsonotherRA<-diversity(abundance2[,134:143], index="invsimpson")

indices$SimpsonARGRA<-diversity(abundance2[,28:143], index="invsimpson")


wilcox.test(SimpsonARGRA~Race2, data=indices)

#p-value =  0.307

wilcox.test(SimpsonMGERA~Race2, data=indices)

#p-value: 0.06063

#fluro, MGE



#shannon

e<-ggboxplot(data=indices, x="Race2", y="SimpsonMGERA", color="black",
fill="Race2",palette=c("#800080", "#A9A9A9"), ylab="Inverse Simpson Diversity Index",
xlab="", outlier.colour = "black", outlier.shape = 1, main="Inverse Simpson MGE")+
theme_classic() + guides(fill=FALSE) + coord_cartesian(ylim = c(0, 8.0))+
stat_compare_means(label.x=1.3, label.y=7.5)

e

f<-ggboxplot(data=indices, x="Race2", y="SimpsonARGRA", color="black",
fill="Race2",palette=c("#800080", "#A9A9A9"), ylab="Inverse Simpson Diversity Index",
xlab="", outlier.colour = "black", outlier.shape = 1, main="Inverse Simpson ARG")+
theme_classic() + guides(fill=FALSE) + coord_cartesian(ylim = c(0, 21.0))+
stat_compare_means(label.x=1.3, label.y=20.5)

f


#combine all 4

# figure <- ggarrange(a, b, c, d, e, f,  labels = c("A", "B", "C","D","E", "F", ncol = 2, nrow = 2))

# figure
```

```
figure <- ggarrange(b, d, f, a, c, e, labels = c("A", "B","C","D"," E", "F", ncol = 2, nrow = 3))

figure

# ggexport(figure, filename = "ShannonMGE_ARG_All.tiff")

ggsave("2023DEC05_InvSimp_MGE_ARG_non_white_MR.tiff", units="in", width=10, 
height=5, dpi=300)



#2023APR24_race_shan_rich_135

# figure <- ggarrange(e, f, labels = c("E", "F", ncol = 2, nrow = 3))

# figure

#ggexport(figure, filename = "SimpsonMGE_ARG_All.pdf")




boxplot<-c(indices$SimpsonMGERA,indices$SimpsonaminoRA,indices$SimpsonMDRRA, 
indices$SimpsonbetaRA, indices$SimpsontetraRA,  indices$SimpsonMLSBRA, 
indices$SimpsonsulfoRA, indices$SimpsonvancoRA, indices$SimpsonfluoroRA)

Race<-rep(indices$Race2,9)

type<-
rep(c("MGE","aminoglycoside","MDR","betalactamase","tetracycline","MLSB","sulfonamide","
vancomycin","fluoroquinolone"),each=204)

length(boxplot)


boxplot2<-data.frame(boxplot,type,Race)


ab<-ggplot(boxplot2, aes(x = type, y = boxplot, fill=Race)) +ylim(0,3)+
geom_boxplot(width=0.4,position=position_dodge(0.4)) + theme_minimal() +
```

```
theme(axis.text.x = element_text(angle = 90)) + labs(title = "", y = "Inverse Inverse Simpson Diversity Index", x = "") + stat_compare_means(aes(group = Race),label="p.signif") + scale_fill_manual(values=c("#800080", "#A9A9A9"))+ theme(legend.position="top",)

ab


#####Beta Diversity####

dev.off()

par(mfrow = c(2,2),mar=c(4,4.3,1,1))

line = -2

cex = 2

adj  = 0.025

Sor.bray.pcoa<-function(abun,Dim=2,Color=1,binary,pch=16,Title="Bray-Curtis MGE"){

 Data.df<-vegdist(abun,method="bray", binary)

 Data.df.PCoA<-cmdscale(Data.df, k = Dim, eig = FALSE)

 Data.df.PCoA.eig<-cmdscale(Data.df, k = Dim, eig = TRUE)

 eig.Data.df.PCoA<-Data.df.PCoA.eig$eig

 eig.Data.df.PCoA.sum<-sum(eig.Data.df.PCoA)

 a<-(eig.Data.df.PCoA/eig.Data.df.PCoA.sum)*100

 xlab<-paste("PC1","(",round(a[1],1),"%",")",sep="")

 ylab<-paste("PC2","(",round(a[2],1),"%",")",sep="")

 if(binary==TRUE){

  main<-"Sorensen PCoA"

 }else(main<-"Bray-Curtis PCoA")

 plot(Data.df.PCoA, col=Color,

    main=Title,xlab=xlab,ylab=ylab,pch=c(pch))

 return(Data.df.PCoA)

}
```

```
abun<-abundance2[1:27]

indices$Race2

Color.Class<-ifelse(grepl("non-white", indices$Race2),"#800080", "#A9A9A9")

b<-as.factor(indices$Race2)

df.spe.bray.Sor<-Sor.bray.pcoa(abun, Dim = 2, Color = Color.Class, binary = FALSE)

#ordiellipse(df.spe.bray.Sor,groups=b,col=c(1,2),lwd=1)


ordiellipse(df.spe.bray.Sor,indices$Race2,col=c("#800080", "#A9A9A9"),lwd=2,bg=2)


title(outer=outer,adj=adj,main="A",cex.main=cex,col="black",font=2,line=line)

text(x = 0.55, y = 0.43, labels = "p = 0.01", xpd = NA,cex=2)

#ordiellipse(df.spe.bray.Sor,groups=b,col=c(1,2),lwd=1)

# text(x = -0.32, y = .42, labels = "A", xpd = NA,cex=2.5)


#legend(-0.3,0.3,c("Infants","Women"), pch=21,col=1,pt.bg=c(1,2))

#text(df.spe.bray.Sor,labels=b,col=as.numeric(b))


#permanova


PERMDISP<-function(abun,Group,binary,iters=9999){

  Data.Dist<-vegdist(abun,method="bray", binary=binary)

  Data.betadisper<-betadisper(Data.Dist, group=Group)

  permutest(Data.betadisper, group=Group, permutations=iters)

}
```

```
#PERMANOVA(abundance2[,c(1:27)],b,FALSE,9999)

#

# PERMDISP(abundance2[,c(1:27)],b,FALSE,9999)




#Sorensen

abun<-presence2[1:27]

Sor.bray.pcoa<-function(abun,Dim=2,Color=1,binary,pch=16,Title="Sorensen MGE"){

  Data.df<-vegdist(abun,method="bray", binary)

  Data.df.PCoA<-cmdscale(Data.df, k = Dim, eig = FALSE)

  Data.df.PCoA.eig<-cmdscale(Data.df, k = Dim, eig = TRUE)

  eig.Data.df.PCoA<-Data.df.PCoA.eig$eig

  eig.Data.df.PCoA.sum<-sum(eig.Data.df.PCoA)

  a<-(eig.Data.df.PCoA/eig.Data.df.PCoA.sum)*100

  xlab<-paste("PC1","(",round(a[1],1),"%",")",sep="")

  ylab<-paste("PC2","(",round(a[2],1),"%",")",sep="")

  if(binary==TRUE){

    main<-"Sorensen PCoA"

  }else(main<-"Bray-Curtis PCoA")

  plot(Data.df.PCoA, col=Color,

       main=Title,xlab=xlab,ylab=ylab,pch=c(pch))

  return(Data.df.PCoA)

}

#sor
```

```
b<-as.factor(indices$Race2)

df.spe.bray.Sor<-Sor.bray.pcoa(abun, Dim = 2, Color = Color.Class, binary = TRUE)

#ordiellipse(df.spe.bray.Sor,groups=b,col=c(1,2),lwd=1)

ordiellipse(df.spe.bray.Sor,indices$Race2,col=c("#800080", "#A9A9A9"),lwd=2,bg=2)

#ordiellipse(df.spe.bray.Sor,groups=b,col=c(1,2),lwd=1)

title(outer=outer,adj=adj,main="B",cex.main=cex,col="black",font=2,line=line)

text(x = 0.38, y = 0.41, labels = "p < 0.001", xpd = NA,cex=2)

#text(x = -0.38, y = .40, labels = "B", xpd = NA,cex=2.5)



#legend(-0.3,0.1,c("Infants","Women"), pch=21,col=1,pt.bg=c(1,2))

#text(df.spe.bray.Sor,labels=b,col=as.numeric(b))


#permanova

PERMANOVA<-function(abun,Group,binary,iters=9999){

  Data.Dist<-vegdist(abun,method="bray", binary=binary)

  adonis2(Data.Dist~Group,permutations=iters)

}

PERMDISP<-function(abun,Group,binary,iters=9999){

  Data.Dist<-vegdist(abun,method="bray", binary=binary)

  Data.betadisper<-betadisper(Data.Dist, group=Group)

  permutest(Data.betadisper, group=Group, permutations=iters)

}


#PERMANOVA(presence2[,c(1:27)],b,TRUE,9999)

#
```

```
# PERMDISP(presence2[,c(1:27)],b,TRUE,9999)


#ARG
abun<-abundance2[28:143]

#par(mfrow = c(2,2),mar=c(4,4.3,1,1))
Sor.bray.pcoa<-function(abun,Dim=2,Color=1,binary,pch=16,Title="Bray-Curtis ARG"){
 Data.df<-vegdist(abun,method="bray", binary)
 Data.df.PCoA<-cmdscale(Data.df, k = Dim, eig = FALSE)
 Data.df.PCoA.eig<-cmdscale(Data.df, k = Dim, eig = TRUE)
 eig.Data.df.PCoA<-Data.df.PCoA.eig$eig
 eig.Data.df.PCoA.sum<-sum(eig.Data.df.PCoA)
 a<-(eig.Data.df.PCoA/eig.Data.df.PCoA.sum)*100
 xlab<-paste("PC1","(",round(a[1],1),"%",")",sep="")
 ylab<-paste("PC2","(",round(a[2],1),"%",")",sep="")
 if(binary==TRUE){
  main<-"Sorensen PCoA"
 }else(main<-"Bray-Curtis PCoA")
 plot(Data.df.PCoA, col=Color,
    main=Title,xlab=xlab,ylab=ylab,pch=c(pch))
 return(Data.df.PCoA)
}

#bray curtis
b<-as.factor(indices$Race2)
```

```
df.spe.bray.Sor<-Sor.bray.pcoa(abun, Dim = 2, Color = Color.Class, binary = FALSE)

#ordiellipse(df.spe.bray.Sor,groups=b,col=c(1,2),lwd=1)

ordiellipse(df.spe.bray.Sor,indices$Race2,col=c("#800080", "#A9A9A9"),lwd=2,bg=2)

title(outer=outer,adj=adj,main="C",cex.main=cex,col="black",font=2,line=line)

text(x = 0.4, y = 0.4, labels = "p = 0.01", xpd = NA,cex=2)

#ordiellipse(df.spe.bray.Sor,groups=b,col=c(1,2),lwd=1)

#text(x = -0.46, y = .39, labels = "C", xpd = NA,cex=2.5)



#legend(0.35,0.5,c("non-white","white"), pch=21,col=1,pt.bg=c(1,2))

#text(df.spe.bray.Sor,labels=b,col=as.numeric(b))


#permanova

# PERMANOVA<-function(abun,Group,binary,iters=9999){

#   Data.Dist<-vegdist(abun,method="bray", binary=binary)

#   adonis2(Data.Dist~Group,permutations=iters)

# }

# PERMDISP<-function(abun,Group,binary,iters=9999){

#   Data.Dist<-vegdist(abun,method="bray", binary=binary)

#   Data.betadisper<-betadisper(Data.Dist, group=Group)

#   permutest(Data.betadisper, group=Group, permutations=iters)

# }

#

# #2023APR24_race_bray_MGE_135

# PERMANOVA(abundance2[,c(28:143)],b,FALSE,9999)

#
```

```
# PERMDISP(abundance2[,c(28:143)],b,FALSE,9999)



#Sorensen

abun<-presence2[28:143]

Sor.bray.pcoa<-function(abun,Dim=2,Color=1,binary,pch=16,Title="Sorensen ARG"){
 Data.df<-vegdist(abun,method="bray", binary)
 Data.df.PCoA<-cmdscale(Data.df, k = Dim, eig = FALSE)
 Data.df.PCoA.eig<-cmdscale(Data.df, k = Dim, eig = TRUE)
 eig.Data.df.PCoA<-Data.df.PCoA.eig$eig
 eig.Data.df.PCoA.sum<-sum(eig.Data.df.PCoA)
 a<-(eig.Data.df.PCoA/eig.Data.df.PCoA.sum)*100
 xlab<-paste("PC1","(",round(a[1],1),"%",")",sep="")
 ylab<-paste("PC2","(",round(a[2],1),"%",")",sep="")
 if(binary==TRUE){
  main<-"Sorensen PCoA"
 }else(main<-"Bray-Curtis PCoA")
 plot(Data.df.PCoA, col=Color,
    main=Title,xlab=xlab,ylab=ylab,pch=c(pch))
 return(Data.df.PCoA)
}
#sor
b<-as.factor(indices$Race2)
df.spe.bray.Sor<-Sor.bray.pcoa(abun, Dim = 2, Color = Color.Class, binary = TRUE)
#ordiellipse(df.spe.bray.Sor,groups=b,col=c(1,2),lwd=1)
#legend(-0.2,0.3,c("non-white","white"), pch=21,col=1,pt.bg=c(1,2))
```

```
#text(df.spe.bray.Sor,labels=b,col=as.numeric(b))

ordiellipse(df.spe.bray.Sor,indices$Race2,col=c("#800080", "#A9A9A9"),lwd=2,bg=2)

title(outer=outer,adj=adj,main="D",cex.main=cex,col="black",font=2,line=line)

text(x = 0.3, y = 0.43, labels = "p < 0.001", xpd = NA,cex=2)

#ordiellipse(df.spe.bray.Sor,groups=b,col=c(1,2),lwd=1)

#text(x = -0.38, y = .44, labels = "D", xpd = NA,cex=2.5)


png("2023DEC04_bray_sor_arg_mge_class_race_MR.tiff", res=300, height=7, width=9,
units="in")


table(indices$Race2)

#permanova

PERMANOVA<-function(abun,Group,binary,iters=9999){

 Data.Dist<-vegdist(abun,method="bray", binary=binary)

 adonis2(Data.Dist~Group,permutations=iters)

}

PERMDISP<-function(abun,Group,binary,iters=9999){

 Data.Dist<-vegdist(abun,method="bray", binary=binary)

 Data.betadisper<-betadisper(Data.Dist, group=Group)

 permutest(Data.betadisper, group=Group, permutations=iters)

}


# PERMANOVA(presence2[,c(28:143)],b,TRUE,9999)

# #p-value= 8e-04 ***

# PERMDISP(presence2[,c(28:143)],b,TRUE,9999)

# #0.0211 ***
```

```
##########mode of delivery################
# 1 = vaginal
# 2= vaginal/forceps
# 3 = vaginal/vacuum
# 4 = cesarean


####doing alpha/beta diveristy by different variables
pres.bcdata
presence<-pres.bcdata
presence2<- presence[,4:146]
presence2

#make sure its the right columns!!!!!
abundance<-bcdata
abundance2<- abundance[,4:146]
head(abundance2)
abundance2[is.na(abundance2)]<- 0
presence2[is.na(presence2)]<- 0

#make indices with columns you want to test
bcdata$BMIcategory
bcdata
bcdata$BMIcategory
indices <- bcdata[,c("ID", "class","pair","Race", "Race2","ESTWKSGEST", "PLURALITY","SEX")]
```

```
#indices <- bcdata[,c("ID", "class","pair","BMIcategory" )]

head(indices)

head(indices)



#Richness

indices$RichMGE<-rowSums(presence2[,1:27])

summary(indices$RichMGE)

indices$Richamino <- rowSums(presence2[,28:48])

summary(indices$Richamino)

indices$RichMDR<-rowSums(presence2[,49:69])

summary(indices$RichMDR)

indices$Richbeta<-rowSums(presence2[,70:85])

summary(indices$Richbeta)

indices$Richtetra<-rowSums(presence2[,86:98])

summary(indices$Richtetra)

indices$RichMLSB<-rowSums(presence2[,99:116])

summary(indices$RichMLSB)

indices$Richsulfo<-rowSums(presence2[,117:121])

summary(indices$Richsulfo)

indices$Richvanco<-rowSums(presence2[,122:129])

summary(indices$Richvanco)

indices$Richfluoro<-rowSums(presence2[,130:133])

summary(indices$Richfluoro)

indices$Richother<-rowSums(presence2[,134:143])

summary(indices$Richother)
```

```
indices$RichARG<-rowSums(presence2[,28:143])


#SHANNON INDEX

#shannon diversity index as default = Relative abundance
indices$ShannonMGERA<-diversity(abundance2[,1:27])
indices$ShannonaminoRA <- diversity(abundance2[,28:48])
indices$ShannonMDRRA<-diversity(abundance2[,49:69])
indices$ShannonbetaRA<-diversity(abundance2[,70:85])
indices$ShannontetraRA<-diversity(abundance2[,86:98])
indices$ShannonMLSBRA<-diversity(abundance2[,99:116])
indices$ShannonsulfoRA<-diversity(abundance2[,117:121])
indices$ShannonvancoRA<-diversity(abundance2[,122:129])
indices$ShannonfluoroRA<-diversity(abundance2[,130:133])
indices$ShannonotherRA<-diversity(abundance2[,134:143])
indices$ShannonARGRA<-diversity(abundance2[,28:143])


#INVERSE SIMPSON INDEX
#Inverse Inverse Simpson Diversity Index = Relative abundance
indices$SimpsonMGERA<-diversity(abundance2[,1:27], index="invsimpson")
indices$SimpsonaminoRA<-diversity(abundance2[,28:48], index="invsimpson")
indices$SimpsonMDRRA<-diversity(abundance2[,49:69], index="invsimpson")
indices$SimpsonbetaRA<-diversity(abundance2[,70:85], index="invsimpson")
indices$SimpsontetraRA<-diversity(abundance2[,86:98], index="invsimpson")
```

```
indices$SimpsonMLSBRA<-diversity(abundance2[,99:116], index="invsimpson")

indices$SimpsonsulfoRA<-diversity(abundance2[,117:121], index="invsimpson")

indices$SimpsonvancoRA<-diversity(abundance2[,122:129], index="invsimpson")

indices$SimpsonfluoroRA<-diversity(abundance2[,130:133], index="invsimpson")

indices$SimpsonotherRA<-diversity(abundance2[,134:143], index="invsimpson")

indices$SimpsonARGRA<-diversity(abundance2[,28:143], index="invsimpson")




indices$MOD<-bcdata$MD_FINAL_ROUTE

indices$MOD

indices$mode<-as.factor(ifelse(indices$MOD== '1','vaginal',

                   ifelse(indices$MOD== '2', 'vaginal',

                       ifelse(indices$MOD== '3', 'vaginal',

                           ifelse(indices$MOD== '4', 'cesarean', 'other')))))



indices$mode

#stacked box plot


dev.off()   # closes the current graphics device

graphics.off() # closes all open graphics devices (safe reset)

boxplot_mod<-c(indices$RichMGE,indices$Richamino,indices$RichMDR,
indices$Richbeta, indices$Richtetra,  indices$RichMLSB, indices$Richsulfo,
indices$Richvanco, indices$Richfluoro)

MOD<-rep(indices$mode,9)
```

```
type<-
rep(c("MGE","aminoglycoside","MDR","betalactamase","tetracycline","MLSB","sulfonamide","
vancomycin","fluoroquinolone"),each=205)

length(boxplot)


boxplot2_mod<-data.frame(boxplot_mod,type,MOD)


rich_class2<-ggplot(boxplot2_mod, aes(x = type, y = boxplot_mod, fill=MOD)) +ylim(0,30)+
geom_boxplot(width=0.4,position=position_dodge(0.4)) + theme_minimal() +
theme(axis.text.x = element_text(size=10, angle = 45)) + labs(title = "", y = "Richness", x = "")
+ stat_compare_means(aes(group = MOD),label="p.signif") +
scale_fill_manual(values=c("#CDB79E","#FF6EB4"))+ theme(legend.position="top",)

rich_class2

#ggexport(ab, filename = "RichMGE_ARG_mode60.tiff")

#ggsave("2023DEC05_rich_MOD_S7a3.tiff", units="in", width=7.5, height=5, dpi=300)


levels(indices$mode)


a<-indices$mode


sumabundance<-aggregate(boxplot~type+MOD, boxplot2,median)

stackplot<-ggplot(data = sumabundance, aes(x = MOD, y=boxplot)) +
geom_bar(stat="identity", aes(fill=type))+theme_classic() +
scale_fill_brewer(palette="Paired")+ labs(title = "", y = "Rich Index", x = "")+theme (axis.text =
element_text(size=14), axis.title=element_text(size=14))

stackplot
```

```
wilcox.test(RichARG~a, data=indices)

#p-value = 0.4976

wilcox.test(RichMGE~a, data=indices)

# p-value =0.6959

#amino


#Richness

b<-ggboxplot(data=indices, x="mode", y="RichARG", color="black", fill="mode",palette=c("#CDB79E","#FF6EB4"), ylab="Richness", xlab="", outlier.colour = "black", outlier.shape = 1, main="Rich ARG")+ theme_classic() + guides(fill=FALSE) + coord_cartesian(ylim = c(0, 100))+ stat_compare_means(label.x=1.3, label.y=95)

b


a<-ggboxplot(data=indices, x="mode", y="RichMGE", color="black", fill="mode",palette=c("#CDB79E","#FF6EB4"), ylab="Richness", xlab="", outlier.colour = "black", outlier.shape = 1, main="Rich MGE")+ theme_classic() + guides(fill=FALSE) + coord_cartesian(ylim = c(0, 40))+ stat_compare_means(label.x=1.7, label.y=35)

a


#combine these

figure <- ggarrange(a, b,labels = c("A", "B", ncol = 2, nrow = 1))

figure

#ggexport(figure, filename = "RichMGE_ARG_mode60.tiff")




##shan tests

method<-indices$mode
```

```
wilcox.test(ShannonMGERA~method, data=indices)

#p-value = 0.524

wilcox.test(ShannonARGRA~method, data=indices)

#p-value = 0.167

#beta, tetra



#shannon

d<-ggboxplot(data=indices, x="mode", y="ShannonARGRA", color="black",
fill="mode",palette=c("#CDB79E","#FF6EB4"), ylab="Shannon Diversity Index", xlab="",
outlier.colour = "black", outlier.shape = 1, main="Shannon ARG")+ theme_classic() +
guides(fill=FALSE) + coord_cartesian(ylim = c(0, 4.0))+ stat_compare_means(label.x=1.3,
label.y=3.8)

d

c<-ggboxplot(data=indices, x="mode", y="ShannonMGERA", color="black",
fill="mode",palette=c("#CDB79E","#FF6EB4"), ylab="Shannon Diversity Index", xlab="",
outlier.colour = "black", outlier.shape = 1, main="Shannon MGE")+ theme_classic() +
guides(fill=FALSE) + coord_cartesian(ylim = c(0, 4.0))+ stat_compare_means(label.x=1.3,
label.y=3.8)

c


#combine all 4

figure <- ggarrange(a, b, c, d, labels = c("A", "B", "C","D", ncol = 2, nrow = 2))

figure

#ggexport(figure, filename = "ShannonMGE_ARG_modeofdeliv60.tiff")



#stacked box plot
```

```
boxplot_shan_mod<-
c(indices$ShannonMGERA,indices$ShannonaminoRA,indices$ShannonMDRRA,
indices$ShannonbetaRA, indices$ShannontetraRA,  indices$ShannonMLSBRA,
indices$ShannonsulfoRA, indices$ShannonvancoRA, indices$ShannonfluoroRA)

method<-rep(indices$mode,9)

type<-
rep(c("MGE","aminoglycoside","MDR","betalactamase","tetracycline","MLSB","sulfonamide","
vancomycin","fluoroquinolone"),each=205)

length(boxplot)


boxplot2_shan_mod<-data.frame(boxplot_shan_mod,type,method)


shan_class2<-ggplot(boxplot2_shan_mod, aes(x = type, y = boxplot_shan_mod, fill=MOD))
+ylim(0,4)+ geom_boxplot(width=0.4,position=position_dodge(0.4)) + theme_minimal() +
theme(axis.text.x = element_text(size=10, angle = 45)) + labs(title = "", y = "Shannon
Diversity Index ", x = "") + stat_compare_means(aes(group = method),label="p.signif") +
scale_fill_manual(values=c("#CDB79E","#FF6EB4"))+ theme(legend.position="top",)

shan_class2

#none were significant

#ggsave("ShanAbudancesMOD_60.tiff", units="in", width=7.5, height=5, dpi=300)

ggsave("2023DEC05_shan_mod_S7b3.tiff", units="in", width=7.5, height=5, dpi=300)


#stacked bar plot

sumabundance<-aggregate(boxplot~type+method, boxplot2,median)

stackplot<-ggplot(data = sumabundance, aes(x = method, y=boxplot)) +
geom_bar(stat="identity", aes(fill=type))+theme_classic() +
scale_fill_brewer(palette="Paired")+ labs(title = "", y = "Shannon Index", x = "")+theme
(axis.text = element_text(size=14), axis.title=element_text(size=14))
```

```
stackplot


#inv simp

method<-indices$mode

wilcox.test(SimpsonMGERA~method, data=indices)

#p-value = 0.6216

wilcox.test(SimpsonARGRA~method, data=indices)

# p-value = 0.0436**

#beta, tetra


#inv simp-- boxplots

e<-ggboxplot(data=indices, x="mode", y="SimpsonMGERA", color="black",
fill="mode",palette=c("#CDB79E","#FF6EB4"), ylab="Inverse Simpson Diversity Index",
xlab="", outlier.colour = "black", outlier.shape = 1, main="Inverse Simpson MGE")+
theme_classic() + guides(fill=FALSE) + coord_cartesian(ylim = c(0, 8.0))+
stat_compare_means(label.x=1.3, label.y=7.5)

e

f<-ggboxplot(data=indices, x="mode", y="SimpsonARGRA", color="black",
fill="mode",palette=c("#CDB79E","#FF6EB4"), ylab="Inverse Simpson Diversity Index",
xlab="", outlier.colour = "black", outlier.shape = 1, main="Inverse Simpson ARG")+
theme_classic() + guides(fill=FALSE) + coord_cartesian(ylim = c(0, 21.0))+
stat_compare_means(label.x=1.3, label.y=20.5)

f


boxplot_simp_mod<-
c(indices$SimpsonMGERA,indices$SimpsonaminoRA,indices$SimpsonMDRRA,
indices$SimpsonbetaRA, indices$SimpsontetraRA,  indices$SimpsonMLSBRA,
indices$SimpsonsulfoRA, indices$SimpsonvancoRA, indices$SimpsonfluoroRA)

mode<-rep(indices$mode,9)
```

```
type<-
rep(c("MGE","aminoglycoside","MDR","betalactamase","tetracycline","MLSB","sulfonamide","
vancomycin","fluoroquinolone"),each=205)
length(boxplot)

boxplot2_simp_mod<-data.frame(boxplot_simp_mod,type,mode)

inv_class2<-ggplot(boxplot2_simp_mod, aes(x = type, y = boxplot_simp_mod, fill=mode)) +
  ylim(0,12)+ geom_boxplot(width=0.4,position=position_dodge(0.4)) +
  theme_minimal() + theme(axis.text.x = element_text(size=10, angle = 45)) +
  labs(title = "", y = "Inverse Simpson Diversity Index", x = "") +
  stat_compare_means(aes(group = mode),label="p.signif") +
  scale_fill_manual(values=c("#CDB79E","#FF6EB4"))+ theme(legend.position="top",)
inv_class2
ggsave("2023DEC05_simp_mod_S7c3.tiff", units="in", width=7.5, height=5, dpi=300)

figure <- ggarrange(rich_class2, shan_class2, inv_class2, labels = c("A", "B","C"))
figure
grid.arrange(rich_class2, shan_class2, inv_class2, nrow = 3)
#combine all 4
figure <- ggarrange(e, f, labels = c("E", "F", ncol = 2, nrow = 3))
figure
ggexport(figure, filename = "ShannonMGE_ARG_All.pdf")

table(indices$pair, indices$mode)

table
```

```
### Richness
boxplot_mod <- c(indices$RichMGE, indices$Richamino, indices$RichMDR,
            indices$Richbeta, indices$Richtetra, indices$RichMLSB,
            indices$Richsulfo, indices$Richvanco, indices$Richfluoro)

MOD <- rep(indices$mode, 9)

type <- rep(c("MGE","aminoglycoside","MDR","betalactamase",
          "tetracycline","MLSB","sulfonamide",
          "vancomycin","fluoroquinolone"), each = 205)

boxplot2_mod <- data.frame(value = boxplot_mod, type = type, mode = MOD)

rich_class2 <- ggplot(boxplot2_mod, aes(x = type, y = value, fill = mode)) +
  geom_boxplot(width = 0.4, position = position_dodge(0.4)) +
  ylim(0, 30) +
  theme_minimal() +
  theme(
    axis.text.x = element_text(size = 10, angle = 45, hjust = 1),
    axis.title = element_text(size = 14),
    legend.position = "top"
  ) +
  labs(y = "Richness", x = "") +
  stat_compare_means(aes(group = mode), label = "p.signif") +
```

```
  scale_fill_manual(values = c("#CDB79E", "#FF6EB4"))



### Shannon

boxplot_shan_mod <- c(indices$ShannonMGERA, indices$ShannonaminoRA,
indices$ShannonMDRRA,
                 indices$ShannonbetaRA, indices$ShannontetraRA, indices$ShannonMLSBRA,
                 indices$ShannonsulfoRA, indices$ShannonvancoRA,
indices$ShannonfluoroRA)


method <- rep(indices$mode, 9)


type <- rep(c("MGE","aminoglycoside","MDR","betalactamase",
          "tetracycline","MLSB","sulfonamide",
          "vancomycin","fluoroquinolone"), each = 205)


boxplot2_shan_mod <- data.frame(value = boxplot_shan_mod, type = type, mode =
method)


shan_class2 <- ggplot(boxplot2_shan_mod, aes(x = type, y = value, fill = mode)) +
  geom_boxplot(width = 0.4, position = position_dodge(0.4)) +
  ylim(0, 4) +
  theme_minimal() +
  theme(
    axis.text.x = element_text(size = 10, angle = 45, hjust = 1),
    axis.title = element_text(size = 14),
    legend.position = "top"
```

```
 ) +
 labs(y = "Shannon Diversity Index", x = "") +
 stat_compare_means(aes(group = mode), label = "p.signif") +
 scale_fill_manual(values = c("#CDB79E", "#FF6EB4"))



### Inverse Simpson
boxplot_simp_mod <- c(indices$SimpsonMGERA, indices$SimpsonaminoRA, indices$SimpsonMDRRA,
                 indices$SimpsonbetaRA, indices$SimpsontetraRA, indices$SimpsonMLSBRA,
                 indices$SimpsonsulfoRA, indices$SimpsonvancoRA, indices$SimpsonfluoroRA)

mode <- rep(indices$mode, 9)

type <- rep(c("MGE","aminoglycoside","MDR","betalactamase",
          "tetracycline","MLSB","sulfonamide",
          "vancomycin","fluoroquinolone"), each = 205)

boxplot2_simp_mod <- data.frame(value = boxplot_simp_mod, type = type, mode = mode)

inv_class2 <- ggplot(boxplot2_simp_mod, aes(x = type, y = value, fill = mode)) +
 geom_boxplot(width = 0.4, position = position_dodge(0.4)) +
 ylim(0, 12) +
 theme_minimal() +
 theme(
  axis.text.x = element_text(size = 10, angle = 45, hjust = 1),
```

```
    axis.title = element_text(size = 14),
    legend.position = "top"
  ) +
  labs(y = "Inverse Simpson Diversity Index", x = "") +
  stat_compare_means(aes(group = mode), label = "p.signif") +
  scale_fill_manual(values = c("#CDB79E", "#FF6EB4"))
inv_class2

###
# ----- Shared base theme -----
base_theme <- theme_classic() +
  theme(
    axis.text.x = element_blank(),
    axis.ticks.x = element_blank(),
    axis.title.x = element_blank()
  )

# ----- Richness -----
rich_class2 <- ggplot(boxplot2_mod, aes(x = type, y = value, fill = mode)) +
  geom_boxplot(width = 0.4, position = position_dodge(0.4)) +
  ylim(0, 30) +
  base_theme +
  theme(axis.text.y = element_text(size = 12),
        axis.title.y = element_text(size = 13, face = "bold"),
        legend.position = "none") +
  labs(y = "Richness") +
```

```
  stat_compare_means(aes(group = mode), label = "p.signif",
                     size = 4, fontface = "bold") +
  scale_fill_manual(values = c("#CDB79E", "#FF6EB4"))

# ----- Shannon -----
shan_class2 <- ggplot(boxplot2_shan_mod, aes(x = type, y = value, fill = mode)) +
  geom_boxplot(width = 0.4, position = position_dodge(0.4)) +
  ylim(0, 4) +
  base_theme +
  theme(axis.text.y = element_text(size = 12),
        axis.title.y = element_text(size = 13, face = "bold"),
        legend.position = "none") +
  labs(y = "Shannon") +
  stat_compare_means(aes(group = mode), label = "p.signif",
                     size = 4, fontface = "bold") +
  scale_fill_manual(values = c("#CDB79E", "#FF6EB4"))

# ----- Inverse Simpson -----
inv_class2 <- ggplot(boxplot2_simp_mod, aes(x = type, y = value, fill = mode)) +
  geom_boxplot(width = 0.4, position = position_dodge(0.4)) +
  ylim(0, 12) +
  base_theme +
  theme(
    axis.text.y = element_text(size = 12),
    axis.title.y = element_text(size = 13, face = "bold"),
    legend.position = "top",
```

```
  legend.title = element_blank(),
  legend.text = element_text(size = 14),     # increase text size
  legend.key.size = unit(1.2, "cm"),        # increase box size
  legend.spacing.x = unit(0.6, "cm")        # spacing between items
 ) +
 labs(y = "Inverse Simpson") +
 stat_compare_means(aes(group = mode), label = "p.signif",
                size = 4, fontface = "bold") +
 scale_fill_manual(values = c("#CDB79E", "#FF6EB4"))

# ----- Combine vertically with shared legend -----
combined_div2 <- ggarrange(
 rich_class2, shan_class2, inv_class2,
 ncol = 1, nrow = 3,
 labels = c("A", "B", "C"),
 font.label = list(size = 12, face = "bold"),
 align = "v",
 common.legend = TRUE, legend = "top",
 heights = c(1, 1, 1)
)
combined_div2


# ----- Dummy x-axis for ARG classes -----
drug_classes <- c("aminoglycoside","betalactamase","fluoroquinolone", "MDR", "MGE",
"MLSB", "sulfonamide",
```

```
            "tetracycline","vancomycin")



xlab_plot2 <- ggplot(data.frame(type = factor(drug_classes, levels = drug_classes), y = 0),
             aes(x = type, y = y)) +
  geom_blank() +
  theme_void() +
  theme(
    axis.text.x = element_text(size = 11, angle = 45, hjust = 0.5, vjust = 0.7),
    axis.ticks.x = element_blank()
  ) +
  labs(x = "ARG Class")

# ----- Stack plots + shared x labels -----
final_plot2 <- grid.arrange(combined_div2, xlab_plot2, heights = c(9, 1))
final_plot2
# ----- Save publication-ready TIFF -----
ggsave(
  filename = "diversity_indices_mode_shared_xaxis_labels.tiff",
  plot = final_plot2,
  device = "tiff",
  width = 6.5, height = 9, units = "in",
  dpi = 600,
  compression = "lzw",
  bg = "white"
)
```

```
#####Beta Diversity

#ARG
abun<-abundance2[28:143]

Sor.bray.pcoa<-function(abun,Dim=2,Color=1,binary,pch=16,Title="Bray-Curtis ARG"){
 Data.df<-vegdist(abun,method="bray", binary)
 Data.df.PCoA<-cmdscale(Data.df, k = Dim, eig = FALSE)
 Data.df.PCoA.eig<-cmdscale(Data.df, k = Dim, eig = TRUE)
 eig.Data.df.PCoA<-Data.df.PCoA.eig$eig
 eig.Data.df.PCoA.sum<-sum(eig.Data.df.PCoA)
 a<-(eig.Data.df.PCoA/eig.Data.df.PCoA.sum)*100
 xlab<-paste("PC1","(",round(a[1],1),"%",")",sep="")
 ylab<-paste("PC2","(",round(a[2],1),"%",")",sep="")
 if(binary==TRUE){
  main<-"Sorensen PCoA"
 }else(main<-"Bray-Curtis PCoA")
 plot(Data.df.PCoA, col=Color,
    main=Title,xlab=xlab,ylab=ylab,pch=c(pch))
 return(Data.df.PCoA)
}
```

```
#bray curtis

dev.off()

par(mfrow = c(2,2),mar=c(4,4.3,1,1))

b<-as.factor(indices$mode)

df.spe.bray.Sor<-Sor.bray.pcoa(abun, Dim = 2, Color = b, binary = FALSE)

ordiellipse(df.spe.bray.Sor,groups=b,col=c(1,2),lwd=1)

#legend(0.3,0.3,c("cesarean","vaginal"), pch=21,col=1,pt.bg=c(1,2))

#black= C, red = vaginal

#text(df.spe.bray.Sor,labels=b,col=as.numeric(b))

#Bray_modeofdelivery_60


#permanova

PERMANOVA<-function(abun,Group,binary,iters=9999){

  Data.Dist<-vegdist(abun,method="bray", binary=binary)

  adonis2(Data.Dist~Group,permutations=iters)

}

PERMDISP<-function(abun,Group,binary,iters=9999){

  Data.Dist<-vegdist(abun,method="bray", binary=binary)

  Data.betadisper<-betadisper(Data.Dist, group=Group)

  permutest(Data.betadisper, group=Group, permutations=iters)

}


PERMANOVA(abundance2[,c(28:143)],b,FALSE,9999)

#p-value=  1e-04 ***

PERMDISP(abundance2[,c(28:143)],b,FALSE,9999)

# 0.7252
```

```
table(indices$mode)


#Sorensen
abun<-presence2[28:143]
Sor.bray.pcoa<-function(abun,Dim=2,Color=1,binary,pch=16,Title="Sorensen ARG"){
 Data.df<-vegdist(abun,method="bray", binary)
 Data.df.PCoA<-cmdscale(Data.df, k = Dim, eig = FALSE)
 Data.df.PCoA.eig<-cmdscale(Data.df, k = Dim, eig = TRUE)
 eig.Data.df.PCoA<-Data.df.PCoA.eig$eig
 eig.Data.df.PCoA.sum<-sum(eig.Data.df.PCoA)
 a<-(eig.Data.df.PCoA/eig.Data.df.PCoA.sum)*100
 xlab<-paste("PC1","(",round(a[1],1),"%",")",sep="")
 ylab<-paste("PC2","(",round(a[2],1),"%",")",sep="")
 if(binary==TRUE){
  main<-"Sorensen PCoA"
 }else(main<-"Bray-Curtis PCoA")
 plot(Data.df.PCoA, col=Color,
    main=Title,xlab=xlab,ylab=ylab,pch=c(pch))
 return(Data.df.PCoA)
}
#sor
b<-as.factor(indices$mode)
df.spe.bray.Sor<-Sor.bray.pcoa(abun, Dim = 2, Color = b, binary = TRUE)
ordiellipse(df.spe.bray.Sor,groups=b,col=c(1,2),lwd=1)
legend(-0.2,-0.4,c("cesarean","vaginal"), pch=21,col=1,pt.bg=c(1,2))
#text(df.spe.bray.Sor,labels=b,col=as.numeric(b))
```

```
#permanova
PERMANOVA<-function(abun,Group,binary,iters=9999){
  Data.Dist<-vegdist(abun,method="bray", binary=binary)
  adonis2(Data.Dist~Group,permutations=iters)
}
PERMDISP<-function(abun,Group,binary,iters=9999){
  Data.Dist<-vegdist(abun,method="bray", binary=binary)
  Data.betadisper<-betadisper(Data.Dist, group=Group)
  permutest(Data.betadisper, group=Group, permutations=iters)
}

PERMANOVA(presence2[,c(28:143)],b,TRUE,9999)
#p-value= 0.0048 **
PERMDISP(presence2[,c(28:143)],b,TRUE,9999)
#0.9034

#ARG--MGE
abun<-abundance2[1:27]

Sor.bray.pcoa<-function(abun,Dim=2,Color=1,binary,pch=16,Title="Bray-Curtis MGE"){
  Data.df<-vegdist(abun,method="bray", binary)
  Data.df.PCoA<-cmdscale(Data.df, k = Dim, eig = FALSE)
  Data.df.PCoA.eig<-cmdscale(Data.df, k = Dim, eig = TRUE)
  eig.Data.df.PCoA<-Data.df.PCoA.eig$eig
  eig.Data.df.PCoA.sum<-sum(eig.Data.df.PCoA)
```

```
  a<-(eig.Data.df.PCoA/eig.Data.df.PCoA.sum)*100
  xlab<-paste("PC1","(",round(a[1],1),"%",")",sep="")
  ylab<-paste("PC2","(",round(a[2],1),"%",")",sep="")
  if(binary==TRUE){
    main<-"Sorensen PCoA"
  }else(main<-"Bray-Curtis PCoA")
  plot(Data.df.PCoA, col=Color,
       main=Title,xlab=xlab,ylab=ylab,pch=c(pch))
  return(Data.df.PCoA)
}

#bray curtis
#dev.off()
#par(mfrow = c(2,2),mar=c(4,4.3,1,1))
b<-as.factor(indices$mode)
df.spe.bray.Sor<-Sor.bray.pcoa(abun, Dim = 2, Color = b, binary = FALSE)
ordiellipse(df.spe.bray.Sor,groups=b,col=c(1,2),lwd=1)
#legend(0.3,0.3,c("cesarean","vaginal"), pch=21,col=1,pt.bg=c(1,2))
#black= C, red = vaginal
#text(df.spe.bray.Sor,labels=b,col=as.numeric(b))
#Bray_modeofdelivery_60

#permanova
PERMANOVA<-function(abun,Group,binary,iters=9999){
  Data.Dist<-vegdist(abun,method="bray", binary=binary)
  adonis2(Data.Dist~Group,permutations=iters)
```

```
}
PERMDISP<-function(abun,Group,binary,iters=9999){
  Data.Dist<-vegdist(abun,method="bray", binary=binary)
  Data.betadisper<-betadisper(Data.Dist, group=Group)
  permutest(Data.betadisper, group=Group, permutations=iters)
}

PERMANOVA(abundance2[,c(1:27)],b,FALSE,9999)
#p-value=   0.0034 **
PERMDISP(abundance2[,c(1:27)],b,FALSE,9999)
# 0.3354
table(indices$mode)

#Sorensen
abun<-presence2[1:27]
Sor.bray.pcoa<-function(abun,Dim=2,Color=1,binary,pch=16,Title="Sorensen MGE"){
  Data.df<-vegdist(abun,method="bray", binary)
  Data.df.PCoA<-cmdscale(Data.df, k = Dim, eig = FALSE)
  Data.df.PCoA.eig<-cmdscale(Data.df, k = Dim, eig = TRUE)
  eig.Data.df.PCoA<-Data.df.PCoA.eig$eig
  eig.Data.df.PCoA.sum<-sum(eig.Data.df.PCoA)
  a<-(eig.Data.df.PCoA/eig.Data.df.PCoA.sum)*100
  xlab<-paste("PC1","(",round(a[1],1),"%",")",sep="")
  ylab<-paste("PC2","(",round(a[2],1),"%",")",sep="")
  if(binary==TRUE){
    main<-"Sorensen PCoA"
```

```
  }else(main<-"Bray-Curtis PCoA")
  plot(Data.df.PCoA, col=Color,
       main=Title,xlab=xlab,ylab=ylab,pch=c(pch))
  return(Data.df.PCoA)
}
#sor
b<-as.factor(indices$mode)
df.spe.bray.Sor<-Sor.bray.pcoa(abun, Dim = 2, Color = b, binary = TRUE)
ordiellipse(df.spe.bray.Sor,groups=b,col=c(1,2),lwd=1)
#legend(-0.2,-0.4,c("cesarean","vaginal"), pch=21,col=1,pt.bg=c(1,2))
#text(df.spe.bray.Sor,labels=b,col=as.numeric(b))

#permanova
PERMANOVA<-function(abun,Group,binary,iters=9999){
  Data.Dist<-vegdist(abun,method="bray", binary=binary)
  adonis2(Data.Dist~Group,permutations=iters)
}
PERMDISP<-function(abun,Group,binary,iters=9999){
  Data.Dist<-vegdist(abun,method="bray", binary=binary)
  Data.betadisper<-betadisper(Data.Dist, group=Group)
  permutest(Data.betadisper, group=Group, permutations=iters)
}

PERMANOVA(presence2[,c(1:27)],b,TRUE,9999)
#p-value= 0.6869
PERMDISP(presence2[,c(1:27)],b,TRUE,9999)
```

```
#0.6641


#####Sex of infant######

####1 = male

####2 = female

indices$SEX<-as.factor(ifelse(indices$SEX== '1','male',

                    ifelse(indices$SEX== '2', 'female','unknown')))

indices$SEX



#stacked box plot

boxplot<-c(indices$RichMGE,indices$Richamino,indices$RichMDR, indices$Richbeta,
indices$Richtetra,  indices$RichMLSB, indices$Richsulfo, indices$Richvanco,
indices$Richfluoro)

SEX<-rep(indices$SEX,9)

type<-
rep(c("MGE","aminoglycoside","MDR","betalactamase","tetracycline","MLSB","sulfonamide","
vancomycin","fluoroquinolone"),each=205)

length(boxplot)


boxplot2<-data.frame(boxplot,type,SEX)


ab<-ggplot(boxplot2, aes(x = type, y = boxplot, fill=SEX)) +ylim(0,30)+
geom_boxplot(width=0.4,position=position_dodge(0.4)) + theme_minimal() +
theme(axis.text.x = element_text(angle = 90)) + labs(title = "", y = "Richness", x = "") +
stat_compare_means(aes(group = SEX),label="p.signif") +
scale_fill_manual(values=c("#E69F00", "#00AFBB", "#E7B800", "#FC4E07",
"#00FF00","#00FF06"))+ theme(legend.position="top",)

ab
```

```
table(indices$SEX)

SEX<-indices$SEX

wilcox.test(RichARG~SEX, data=indices)
#p-value = 0.8348
wilcox.test(RichMGE~SEX, data=indices)
# p-value = 0.8836

#Richness
b<-ggboxplot(data=indices, x="SEX", y="RichARG", color="black", fill="SEX",palette="d3",
ylab="Richness", xlab="", outlier.colour = "black", outlier.shape = 1, main="Rich ARG")+
theme_classic() + guides(fill=FALSE) + coord_cartesian(ylim = c(0, 100))+
stat_compare_means(label.x=1.3, label.y=95)
b

a<-ggboxplot(data=indices, x="SEX", y="RichMGE", color="black", fill="SEX",palette="d3",
ylab="Richness", xlab="", outlier.colour = "black", outlier.shape = 1, main="Rich MGE")+
theme_classic() + guides(fill=FALSE) + coord_cartesian(ylim = c(0, 40))+
stat_compare_means(label.x=1.7, label.y=35)
a

#combine these
figure <- ggarrange(a, b,labels = c("A", "B", ncol = 2, nrow = 1))
figure
#ggexport(figure, filename = "RichMGE_ARG_sex60.tiff")
```

```
##shan tests

SEX<-indices$SEX

wilcox.test(ShannonMGERA~SEX, data=indices)

#p-value = 0.5568

wilcox.test(ShannonARGRA~SEX, data=indices)

#p-value = 0.2919

table(indices$PLURALITY)



#shannon

d<-ggboxplot(data=indices, x="SEX", y="ShannonARGRA", color="black",
fill="SEX",palette="d3", ylab="Shannon Diversity Index", xlab="", outlier.colour = "black",
outlier.shape = 1, main="Shannon ARG")+ theme_classic() + guides(fill=FALSE) +
coord_cartesian(ylim = c(0, 4.0))+ stat_compare_means(label.x=1.3, label.y=3.8)

d

c<-ggboxplot(data=indices, x="SEX", y="ShannonMGERA", color="black",
fill="SEX",palette="d3", ylab="Shannon Diversity Index", xlab="", outlier.colour = "black",
outlier.shape = 1, main="Shannon MGE")+ theme_classic() + guides(fill=FALSE) +
coord_cartesian(ylim = c(0, 4.0))+ stat_compare_means(label.x=1.3, label.y=3.8)

c


#combine all 4

figure <- ggarrange(a, b, c, d, labels = c("A", "B", "C","D", ncol = 2, nrow = 2))

figure

#ggexport(figure, filename = "ShannonMGE_ARG_sex60.tiff")



#stacked box plot
```

```
boxplot<-c(indices$ShannonMGERA,indices$ShannonaminoRA,indices$ShannonMDRRA,
indices$ShannonbetaRA, indices$ShannontetraRA,  indices$ShannonMLSBRA,
indices$ShannonsulfoRA, indices$ShannonvancoRA, indices$ShannonfluoroRA)

SEX<-rep(indices$SEX,9)

type<-
rep(c("MGE","aminoglycoside","MDR","betalactamase","tetracycline","MLSB","sulfonamide","
vancomycin","fluoroquinolone"),each=205)

length(boxplot)


boxplot2<-data.frame(boxplot,type,SEX)


ab<-ggplot(boxplot2, aes(x = type, y = boxplot, fill=SEX)) +ylim(0,4)+
geom_boxplot(width=0.4,position=position_dodge(0.4)) + theme_minimal() +
theme(axis.text.x = element_text(angle = 90)) + labs(title = "", y = "Shannon Diversity Index ",
x = "") + stat_compare_means(aes(group = SEX),label="p.signif") +
scale_fill_manual(values=c("#E69F00", "#56B4E9", "#E69F00"))+
theme(legend.position="top",)

ab

#ggsave("ShanAbudancesMOD_60.tiff", units="in", width=7.5, height=5, dpi=300)


#stacked bar plot

sumabundance<-aggregate(boxplot~type+SEX, boxplot2,median)

stackplot<-ggplot(data = sumabundance, aes(x = SEX, y=boxplot)) +
geom_bar(stat="identity", aes(fill=type))+theme_classic() +
scale_fill_brewer(palette="Paired")+ labs(title = "", y = "Shannon Index", x = "")+theme
(axis.text = element_text(size=14), axis.title=element_text(size=14))

stackplot


#inv simp

SEX<-indices$SEX
```

```
wilcox.test(SimpsonMGERA~SEX, data=indices)

#p-value = 0.4631

wilcox.test(SimpsonARGRA~SEX, data=indices)

# p-value = 0.06955


#inv simp-- boxplots

e<-ggboxplot(data=indices, x="SEX", y="SimpsonMGERA", color="black", fill="SEX",palette="d3", ylab="Inverse Simpson Diversity Index", xlab="", outlier.colour = "black", outlier.shape = 1, main="Inverse Simpson MGE")+ theme_classic() + guides(fill=FALSE) + coord_cartesian(ylim = c(0, 8.0))+ stat_compare_means(label.x=1.3, label.y=7.5)

e

f<-ggboxplot(data=indices, x="SEX", y="SimpsonARGRA", color="black", fill="SEX",palette="d3", ylab="Inverse Simpson Diversity Index", xlab="", outlier.colour = "black", outlier.shape = 1, main="Inverse Simpson ARG")+ theme_classic() + guides(fill=FALSE) + coord_cartesian(ylim = c(0, 21.0))+ stat_compare_means(label.x=1.3, label.y=20.5)

f


#combine all 4

figure <- ggarrange(e, f, labels = c("E", "F", ncol = 2, nrow = 3))

figure

ggexport(figure, filename = "SimpsonMGE_ARG_Sex.pdf")


summary(indices$ESTWKSGEST)

#####Beta Diversity####


#ARG= 28:143, MGE = 1:27
```

```
abun<-abundance2[28:143]

Sor.bray.pcoa<-function(abun,Dim=2,Color=1,binary,pch=16,Title="Bray-Curtis ARG"){
 Data.df<-vegdist(abun,method="bray", binary)
 Data.df.PCoA<-cmdscale(Data.df, k = Dim, eig = FALSE)
 Data.df.PCoA.eig<-cmdscale(Data.df, k = Dim, eig = TRUE)
 eig.Data.df.PCoA<-Data.df.PCoA.eig$eig
 eig.Data.df.PCoA.sum<-sum(eig.Data.df.PCoA)
 a<-(eig.Data.df.PCoA/eig.Data.df.PCoA.sum)*100
 xlab<-paste("PC1","(",round(a[1],1),"%",")",sep="")
 ylab<-paste("PC2","(",round(a[2],1),"%",")",sep="")
 if(binary==TRUE){
  main<-"Sorensen PCoA"
 }else(main<-"Bray-Curtis PCoA")
 plot(Data.df.PCoA, col=Color,
    main=Title,xlab=xlab,ylab=ylab,pch=c(pch))
 return(Data.df.PCoA)
}

#bray curtis
dev.off()
par(mfrow = c(2,2),mar=c(4,4.3,1,1))
b<-as.factor(indices$SEX)
df.spe.bray.Sor<-Sor.bray.pcoa(abun, Dim = 2, Color = b, binary = FALSE)
ordiellipse(df.spe.bray.Sor,groups=b,col=c(1,2),lwd=1)
legend(0.5,0.2,c("female","male"), pch=21,col=1,pt.bg=c(1,2))
```

```
#text(df.spe.bray.Sor,labels=b,col=as.numeric(b))
#Bray_sex_60
#black=female, red = male


#permanova
PERMANOVA<-function(abun,Group,binary,iters=9999){
  Data.Dist<-vegdist(abun,method="bray", binary=binary)
  adonis2(Data.Dist~Group,permutations=iters)
}
PERMDISP<-function(abun,Group,binary,iters=9999){
  Data.Dist<-vegdist(abun,method="bray", binary=binary)
  Data.betadisper<-betadisper(Data.Dist, group=Group)
  permutest(Data.betadisper, group=Group, permutations=iters)
}


PERMANOVA(abundance2[,c(28:143)],b,FALSE,9999)
#p-value=  0.0065 **
PERMDISP(abundance2[,c(28:143)],b,FALSE,9999)
# 0.9


#Sorensen
abun<-presence2[28:143]
Sor.bray.pcoa<-function(abun,Dim=2,Color=1,binary,pch=16,Title="Sorensen ARG"){
  Data.df<-vegdist(abun,method="bray", binary)
  Data.df.PCoA<-cmdscale(Data.df, k = Dim, eig = FALSE)
  Data.df.PCoA.eig<-cmdscale(Data.df, k = Dim, eig = TRUE)
```

```
  eig.Data.df.PCoA<-Data.df.PCoA.eig$eig
  eig.Data.df.PCoA.sum<-sum(eig.Data.df.PCoA)
  a<-(eig.Data.df.PCoA/eig.Data.df.PCoA.sum)*100
  xlab<-paste("PC1","(",round(a[1],1),"%",")",sep="")
  ylab<-paste("PC2","(",round(a[2],1),"%",")",sep="")
  if(binary==TRUE){
    main<-"Sorensen PCoA"
  }else(main<-"Bray-Curtis PCoA")
  plot(Data.df.PCoA, col=Color,
       main=Title,xlab=xlab,ylab=ylab,pch=c(pch))
  return(Data.df.PCoA)
}
#sor
b<-as.factor(indices$SEX)
df.spe.bray.Sor<-Sor.bray.pcoa(abun, Dim = 2, Color = b, binary = TRUE)
ordiellipse(df.spe.bray.Sor,groups=b,col=c(1,2),lwd=1)
#legend(-0.2,0.3,c("female","male"), pch=21,col=1,pt.bg=c(1,2))
#text(df.spe.bray.Sor,labels=b,col=as.numeric(b))

#permanova
PERMANOVA<-function(abun,Group,binary,iters=9999){
  Data.Dist<-vegdist(abun,method="bray", binary=binary)
  adonis2(Data.Dist~Group,permutations=iters)
}
PERMDISP<-function(abun,Group,binary,iters=9999){
  Data.Dist<-vegdist(abun,method="bray", binary=binary)
```

```
  Data.betadisper<-betadisper(Data.Dist, group=Group)
  permutest(Data.betadisper, group=Group, permutations=iters)
}


PERMANOVA(presence2[,c(28:143)],b,TRUE,9999)
#p-value=0.0048 **
PERMDISP(presence2[,c(28:143)],b,TRUE,9999)
# 0.9034

#MGE
#ARG= 28:143, MGE = 1:27
abun<-abundance2[1:27]

Sor.bray.pcoa<-function(abun,Dim=2,Color=1,binary,pch=16,Title="Bray-Curtis MGE"){
  Data.df<-vegdist(abun,method="bray", binary)
  Data.df.PCoA<-cmdscale(Data.df, k = Dim, eig = FALSE)
  Data.df.PCoA.eig<-cmdscale(Data.df, k = Dim, eig = TRUE)
  eig.Data.df.PCoA<-Data.df.PCoA.eig$eig
  eig.Data.df.PCoA.sum<-sum(eig.Data.df.PCoA)
  a<-(eig.Data.df.PCoA/eig.Data.df.PCoA.sum)*100
  xlab<-paste("PC1","(",round(a[1],1),"%",")",sep="")
  ylab<-paste("PC2","(",round(a[2],1),"%",")",sep="")
  if(binary==TRUE){
    main<-"Sorensen PCoA"
  }else(main<-"Bray-Curtis PCoA")
```

```
  plot(Data.df.PCoA, col=Color,
      main=Title,xlab=xlab,ylab=ylab,pch=c(pch))
  return(Data.df.PCoA)
}

#bray curtis
#dev.off()
#par(mfrow = c(2,2),mar=c(4,4.3,1,1))
b<-as.factor(indices$SEX)
df.spe.bray.Sor<-Sor.bray.pcoa(abun, Dim = 2, Color = b, binary = FALSE)
ordiellipse(df.spe.bray.Sor,groups=b,col=c(1,2),lwd=1)
#legend(0.5,0.2,c("female","male"), pch=21,col=1,pt.bg=c(1,2))
#text(df.spe.bray.Sor,labels=b,col=as.numeric(b))
#Bray_sex_60
#black=female, red = male

#permanova
PERMANOVA<-function(abun,Group,binary,iters=9999){
  Data.Dist<-vegdist(abun,method="bray", binary=binary)
  adonis2(Data.Dist~Group,permutations=iters)
}
PERMDISP<-function(abun,Group,binary,iters=9999){
  Data.Dist<-vegdist(abun,method="bray", binary=binary)
  Data.betadisper<-betadisper(Data.Dist, group=Group)
  permutest(Data.betadisper, group=Group, permutations=iters)
}
```

```
PERMANOVA(abundance2[,c(1:27)],b,FALSE,9999)
#p-value=  0.2415
PERMDISP(abundance2[,c(1:27)],b,FALSE,9999)
# 0.1002 .

#Sorensen
abun<-presence2[1:27]
Sor.bray.pcoa<-function(abun,Dim=2,Color=1,binary,pch=16,Title="Sorensen MGE"){
 Data.df<-vegdist(abun,method="bray", binary)
 Data.df.PCoA<-cmdscale(Data.df, k = Dim, eig = FALSE)
 Data.df.PCoA.eig<-cmdscale(Data.df, k = Dim, eig = TRUE)
 eig.Data.df.PCoA<-Data.df.PCoA.eig$eig
 eig.Data.df.PCoA.sum<-sum(eig.Data.df.PCoA)
 a<-(eig.Data.df.PCoA/eig.Data.df.PCoA.sum)*100
 xlab<-paste("PC1","(",round(a[1],1),"%",")",sep="")
 ylab<-paste("PC2","(",round(a[2],1),"%",")",sep="")
 if(binary==TRUE){
  main<-"Sorensen PCoA"
 }else(main<-"Bray-Curtis PCoA")
 plot(Data.df.PCoA, col=Color,
    main=Title,xlab=xlab,ylab=ylab,pch=c(pch))
 return(Data.df.PCoA)
}
#sor
b<-as.factor(indices$SEX)
```

```
df.spe.bray.Sor<-Sor.bray.pcoa(abun, Dim = 2, Color = b, binary = TRUE)
ordiellipse(df.spe.bray.Sor,groups=b,col=c(1,2),lwd=1)
#legend(-0.2,0.3,c("female","male"), pch=21,col=1,pt.bg=c(1,2))
#text(df.spe.bray.Sor,labels=b,col=as.numeric(b))

#permanova
PERMANOVA<-function(abun,Group,binary,iters=9999){
  Data.Dist<-vegdist(abun,method="bray", binary=binary)
  adonis2(Data.Dist~Group,permutations=iters)
}
PERMDISP<-function(abun,Group,binary,iters=9999){
  Data.Dist<-vegdist(abun,method="bray", binary=binary)
  Data.betadisper<-betadisper(Data.Dist, group=Group)
  permutest(Data.betadisper, group=Group, permutations=iters)
}


PERMANOVA(presence2[,c(1:27)],b,TRUE,9999)
#p-value=0.9187
PERMDISP(presence2[,c(1:27)],b,TRUE,9999)
#  0.5563

####BMI######

bcdata$MOM_HEIGHT_IN
ft<-bcdata$MOM_HEIGHT_FT
```

```
ft

ft_to_in<-ft*12

ft_to_in

inches<-bcdata$MOM_HEIGHT_IN

height_in_inches<-ft_to_in + inches

height_in_inches

bcdata$height<-height_in_inches

bcdata$height


height<-bcdata$height

bcdata$PRE_PREG_WT

weight<-bcdata$PRE_PREG_WT


a<-as.factor(bcdata$height)

a


bcdata$PRE_PREG_WT<-gsub('999',"",bcdata$PRE_PREG_WT)

b<-as.factor(bcdata$PRE_PREG_WT)

b

#make it a numeric factor

height<-as.numeric(as.character(a))

height<-height*0.0254

height #height in meters


weight<-as.numeric(as.character(b))

weight<-weight/2.205 #weight in kg
```

```
BMI<-weight/(height*height)

bcdata$BMI<-BMI

bcdata$weight<-weight

bcdata$height_in_meters<-height


bcdata$BMI

table(bcdata$Race2)


#creating BMI categories

bcdata$BMIcategory<-as.factor(ifelse(bcdata$BMI <= 18.5, 'normal',

                          ifelse((bcdata$BMI >= 25) & (bcdata$BMI <= 29.9), 'overweight',

                             ifelse(bcdata$BMI >= 30, 'obese', 'normal'))))




mean(bcdata$BMI, na.rm = TRUE)

sd(bcdata$BMI, na.rm = TRUE)


indices_omit<-indices %>% drop_na(BMIcategory)

indices$BMI<-bcdata$BMIcategory

mean(BMIcategory)


BMI<-indices$BMIcategory
```

```
indices_omit<-indices %>% drop_na(BMIcategory)

indices<-indices_omit



df3<-abundance %>%

  filter(ID %in% indices_omit$ID)

abundance<-df3


df4<-presence %>%

  filter(ID %in% indices$ID)

indices$BMI<-indices$BMIcategory


abundance<-df3

presence<-df4


presence2<- presence[,4:146]

presence2

abundance2<- abundance[,4:146]


#stacked box plot

indices$BMI2<-factor(indices$BMI,levels(indices$BMI)[c(1,3,2)])


boxplot<-c(indices$RichMGE,indices$Richamino,indices$RichMDR, indices$Richbeta,
indices$Richtetra,  indices$RichMLSB, indices$Richsulfo, indices$Richvanco,
indices$Richfluoro)

BMI<-rep(indices$BMI2,9)
```

```
type<-
rep(c("MGE","aminoglycoside","MDR","betalactamase","tetracycline","MLSB","sulfonamide","
vancomycin","fluoroquinolone"),each=200)

length(boxplot)


boxplot2<-data.frame(boxplot,type,BMI)


ab<-ggplot(boxplot2, aes(x = type, y = boxplot, fill=BMI)) +ylim(0,30)+
geom_boxplot(width=0.4,position=position_dodge(0.4)) + theme_minimal() +
theme(axis.text.x = element_text(size=10, angle = 45)) + labs(title = "", y = "Richness", x = "")
+ stat_compare_means(aes(group = BMI),label="p.signif") +
scale_fill_manual(values=c("#00BFFF", "#FC4E07", "#00FF00"))+
theme(legend.position="top",)

ab

ggsave("2023DEC05_rich_BMI_S82.tiff", units="in", width=7.5, height=5, dpi=300)


#MDR, amino

BMI<-indices$BMI

dev.off()

indices$Richamino

boxplot(indices$Richamino~BMI,main="richness BMI",ylab="Richness")



kruskal.test(RichARG~BMI)

#0.05191

kruskal.test(RichMGE~BMI, data=indices_omit)

# p-value = 0.2064

kruskal.test(indices$Richamino~BMI)
```

```
#0.01271

kruskal.test(indices$RichMDR~BMI)

#0.03974

#load library

library(FSA)

#install.packages("FSA")

dunnTest(Richamino ~ BMI,
      data=indices,
      method="bonferroni")



#Richness

b<-ggboxplot(data=indices_omit, x="BMI", y="RichARG", color="black", fill="BMI",palette="d3", ylab="Richness", xlab="", outlier.colour = "black", outlier.shape = 1, main="Rich ARG")+ theme_classic() + guides(fill=FALSE) + coord_cartesian(ylim = c(0, 100))+ stat_compare_means(label.x=1.3, label.y=95)

b


a<-ggboxplot(data=indices_omit, x="BMI", y="RichMGE", color="black", fill="BMI",palette="d3", ylab="Richness", xlab="", outlier.colour = "black", outlier.shape = 1, main="Rich MGE")+ theme_classic() + guides(fill=FALSE) + coord_cartesian(ylim = c(0, 40))+ stat_compare_means(label.x=1.7, label.y=35)

a


#combine these

figure <- ggarrange(a, b,labels = c("A", "B", ncol = 2, nrow = 1))

figure

#ggexport(figure, filename = "RichMGE_ARG_sex60.tiff")
```

```
##shan tests

BMI<-indices_omit$BMI

ShannonMGERA<-indices_omit$ShannonMGERA

kruskal.test(ShannonMGERA~BMI, data=indices_omit)

#p-value = 0.929

kruskal.test(ShannonARGRA~BMI, data=indices_omit)

#p-value = 0.3967


aggregate(ShannonMGERA,by=list(BMI),FUN=summary)



#shannon

d<-ggboxplot(data=indices_omit, x="BMI", y="ShannonARGRA", color="black",
fill="BMI",palette="d3", ylab="Shannon Diversity Index", xlab="", outlier.colour = "black",
outlier.shape = 1, main="Shannon ARG")+ theme_classic() + guides(fill=FALSE) +
coord_cartesian(ylim = c(0, 4.0))+ stat_compare_means(label.x=1.3, label.y=3.8)

d

c<-ggboxplot(data=indices_omit, x="BMI", y="ShannonMGERA", color="black",
fill="BMI",palette="d3", ylab="Shannon Diversity Index", xlab="", outlier.colour = "black",
outlier.shape = 1, main="Shannon MGE")+ theme_classic() + guides(fill=FALSE) +
coord_cartesian(ylim = c(0, 4.0))+ stat_compare_means(label.x=1.3, label.y=3.8)

c


#combine all 4

figure <- ggarrange(a, b, c, d, labels = c("A", "B", "C","D", ncol = 2, nrow = 2))

figure
```

```
#ggexport(figure, filename = "ShannonMGE_ARG_BMI60.tiff")



#stacked box plot

boxplot<-c(indices$ShannonMGERA,indices$ShannonaminoRA,indices$ShannonMDRRA, indices$ShannonbetaRA, indices$ShannontetraRA,  indices$ShannonMLSBRA, indices$ShannonsulfoRA, indices$ShannonvancoRA, indices$ShannonfluoroRA)

BMI<-rep(indices$BMI,9)

type<-
rep(c("MGE","aminoglycoside","MDR","betalactamase","tetracycline","MLSB","sulfonamide","vancomycin","fluoroquinolone"),each=200)

length(boxplot)


boxplot2<-data.frame(boxplot,type,BMI)


ab<-ggplot(boxplot2, aes(x = type, y = boxplot, fill=BMI)) +ylim(0,4)+
geom_boxplot(width=0.4,position=position_dodge(0.4)) + theme_minimal() +
theme(axis.text.x = element_text(angle = 90)) + labs(title = "", y = "Shannon Diversity Index ",
x = "") + stat_compare_means(aes(group = BMI),label="p.signif") +
scale_fill_manual(values=c("#E69F00", "#56B4E9", "#FC4E07","#00FF00"))+
theme(legend.position="top",)

ab

ggsave("ShanAbudancesBMI_60.tiff", units="in", width=7.5, height=5, dpi=300)

#MGE significant


#stacked bar plot

sumabundance<-aggregate(boxplot~type+BMI, boxplot2,median)

stackplot<-ggplot(data = sumabundance, aes(x = BMI, y=boxplot)) +
geom_bar(stat="identity", aes(fill=type))+theme_classic() +
```

```
scale_fill_brewer(palette="Paired")+ labs(title = "", y = "Shannon Index", x = "")+theme
(axis.text = element_text(size=14), axis.title=element_text(size=14))

stackplot


#inv simp

BMI<-indices$BMI

kruskal.test(SimpsonMGERA~BMI, data=indices)

#p-value =0.7826

kruskal.test(SimpsonARGRA~BMI, data=indices)

# p-value = 0.173


#inv simp-- boxplots

e<-ggboxplot(data=indices, x="BMI", y="SimpsonMGERA", color="black",
fill="BMI",palette="d3", ylab="Inverse Simpson Diversity Index", xlab="", outlier.colour =
"black", outlier.shape = 1, main="Inverse Simpson MGE")+ theme_classic() +
guides(fill=FALSE) + coord_cartesian(ylim = c(0, 8.0))+ stat_compare_means(label.x=1.3,
label.y=7.5)

e

f<-ggboxplot(data=indices, x="BMI", y="SimpsonARGRA", color="black",
fill="BMI",palette="d3", ylab="Inverse Simpson Diversity Index", xlab="", outlier.colour =
"black", outlier.shape = 1, main="Inverse Simpson ARG")+ theme_classic() +
guides(fill=FALSE) + coord_cartesian(ylim = c(0, 21.0))+ stat_compare_means(label.x=1.3,
label.y=20.5)

f


#combine all 4


figure <- ggarrange(c, d, e, f, labels = c("A", "B", "C","D", ncol = 2, nrow = 2))

figure
```

```
###beta diversity
#ARG
# abundance2$BMI<-indices$BMI
# abundance2$BMI
# abundance3<-abundance2 %>% drop_na(BMI)
# abundance2<-abundance3[,-c(144)]

abun<-abundance2[1:27]

Sor.bray.pcoa<-function(abun,Dim=2,Color=1,binary,pch=16,Title="Bray-Curtis MGE"){
 Data.df<-vegdist(abun,method="bray", binary)
 Data.df.PCoA<-cmdscale(Data.df, k = Dim, eig = FALSE)
 Data.df.PCoA.eig<-cmdscale(Data.df, k = Dim, eig = TRUE)
 eig.Data.df.PCoA<-Data.df.PCoA.eig$eig
 eig.Data.df.PCoA.sum<-sum(eig.Data.df.PCoA)
 a<-(eig.Data.df.PCoA/eig.Data.df.PCoA.sum)*100
 xlab<-paste("PC1","(",round(a[1],1),"%",")",sep="")
 ylab<-paste("PC2","(",round(a[2],1),"%",")",sep="")
 if(binary==TRUE){
  main<-"Sorensen PCoA"
 }else(main<-"Bray-Curtis PCoA")
 plot(Data.df.PCoA, col=Color,
    main=Title,xlab=xlab,ylab=ylab,pch=c(pch))
 return(Data.df.PCoA)
}
```

```
#bray curtis
b<-as.factor(indices$BMI)
df.spe.bray.Sor<-Sor.bray.pcoa(abun, Dim = 2, Color = b, binary = FALSE)
ordiellipse(df.spe.bray.Sor,groups=b,col=c(1,2),lwd=1)
legend(0.45,0.2,c("normal","obese", "overweight"), pch=21,col=1,pt.bg=c(1,2,3))
#text(df.spe.bray.Sor,labels=b,col=as.numeric(b))
#Bray_sex_60

#permanova
PERMANOVA<-function(abun,Group,binary,iters=9999){
 Data.Dist<-vegdist(abun,method="bray", binary=binary)
 adonis2(Data.Dist~Group,permutations=iters)
}
PERMDISP<-function(abun,Group,binary,iters=9999){
 Data.Dist<-vegdist(abun,method="bray", binary=binary)
 Data.betadisper<-betadisper(Data.Dist, group=Group)
 permutest(Data.betadisper, group=Group, permutations=iters)
}

PERMANOVA(abundance2[,c(1:27)],b,FALSE,9999)
#p-value=0.2557
PERMDISP(abundance2[,c(1:27)],b,FALSE,9999)
#0.1534

#Sorensen
```

```
# presence2$BMI<-indices_omit$BMI
# presence2$BMI
# presence3<-presence2 %>% drop_na(BMI)
presence2<-presence[,4:146]

abun<-presence2[1:27]
Sor.bray.pcoa<-function(abun,Dim=2,Color=1,binary,pch=16,Title="Sorensen MGE"){
 Data.df<-vegdist(abun,method="bray", binary)
 Data.df.PCoA<-cmdscale(Data.df, k = Dim, eig = FALSE)
 Data.df.PCoA.eig<-cmdscale(Data.df, k = Dim, eig = TRUE)
 eig.Data.df.PCoA<-Data.df.PCoA.eig$eig
 eig.Data.df.PCoA.sum<-sum(eig.Data.df.PCoA)
 a<-(eig.Data.df.PCoA/eig.Data.df.PCoA.sum)*100
 xlab<-paste("PC1","(",round(a[1],1),"%",")",sep="")
 ylab<-paste("PC2","(",round(a[2],1),"%",")",sep="")
 if(binary==TRUE){
  main<-"Sorensen PCoA"
 }else(main<-"Bray-Curtis PCoA")
 plot(Data.df.PCoA, col=Color,
    main=Title,xlab=xlab,ylab=ylab,pch=c(pch))
 return(Data.df.PCoA)
}
#sor
b<-as.factor(indices$BMI)
df.spe.bray.Sor<-Sor.bray.pcoa(abun, Dim = 2, Color = b, binary = TRUE)
ordiellipse(df.spe.bray.Sor,groups=b,col=c(1,2),lwd=1)
```

```
legend(-0.2, 0.4,c("normal","obese", "overweight"), pch=21,col=1,pt.bg=c(1,2,3))
#text(df.spe.bray.Sor,labels=b,col=as.numeric(b))
#normal = black
#obese = red
#overweight = green

#permanova
PERMANOVA<-function(abun,Group,binary,iters=9999){
  Data.Dist<-vegdist(abun,method="bray", binary=binary)
  adonis2(Data.Dist~Group,permutations=iters)
}
PERMDISP<-function(abun,Group,binary,iters=9999){
  Data.Dist<-vegdist(abun,method="bray", binary=binary)
  Data.betadisper<-betadisper(Data.Dist, group=Group)
  permutest(Data.betadisper, group=Group, permutations=iters)
}

PERMANOVA(presence2[1:27],b,TRUE,9999)
#p-value=0.5953
PERMDISP(presence2[1:27],b,TRUE,9999)
#0.097 .


#ARG
abun<-abundance2[28:143]
```

```
Sor.bray.pcoa<-function(abun,Dim=2,Color=1,binary,pch=16,Title="Bray-Curtis ARG"){
 Data.df<-vegdist(abun,method="bray", binary)
 Data.df.PCoA<-cmdscale(Data.df, k = Dim, eig = FALSE)
 Data.df.PCoA.eig<-cmdscale(Data.df, k = Dim, eig = TRUE)
 eig.Data.df.PCoA<-Data.df.PCoA.eig$eig
 eig.Data.df.PCoA.sum<-sum(eig.Data.df.PCoA)
 a<-(eig.Data.df.PCoA/eig.Data.df.PCoA.sum)*100
 xlab<-paste("PC1","(",round(a[1],1),"%",")",sep="")
 ylab<-paste("PC2","(",round(a[2],1),"%",")",sep="")
 if(binary==TRUE){
  main<-"Sorensen PCoA"
 }else(main<-"Bray-Curtis PCoA")
 plot(Data.df.PCoA, col=Color,
     main=Title,xlab=xlab,ylab=ylab,pch=c(pch))
 return(Data.df.PCoA)
}

#bray curtis
b<-as.factor(indices$BMI)
df.spe.bray.Sor<-Sor.bray.pcoa(abun, Dim = 2, Color = b, binary = FALSE)
ordiellipse(df.spe.bray.Sor,groups=b,col=c(1,2),lwd=1)
legend(-0.45,0.3,c("normal","obese", "overweight"), pch=21,col=1,pt.bg=c(1,2,3))
#text(df.spe.bray.Sor,labels=b,col=as.numeric(b))
#Bray_sex_60

#permanova
```

```
PERMANOVA<-function(abun,Group,binary,iters=9999){
 Data.Dist<-vegdist(abun,method="bray", binary=binary)
 adonis2(Data.Dist~Group,permutations=iters)
}
PERMDISP<-function(abun,Group,binary,iters=9999){
 Data.Dist<-vegdist(abun,method="bray", binary=binary)
 Data.betadisper<-betadisper(Data.Dist, group=Group)
 permutest(Data.betadisper, group=Group, permutations=iters)
}

PERMANOVA(abundance2[,c(28:143)],b,FALSE,9999)
#p-value=0.2792
PERMDISP(abundance2[,c(28:143)],b,FALSE,9999)
#0.7547

#Sorensen
abun<-presence2[28:143]
Sor.bray.pcoa<-function(abun,Dim=2,Color=1,binary,pch=16,Title="Sorensen MGE"){
 Data.df<-vegdist(abun,method="bray", binary)
 Data.df.PCoA<-cmdscale(Data.df, k = Dim, eig = FALSE)
 Data.df.PCoA.eig<-cmdscale(Data.df, k = Dim, eig = TRUE)
 eig.Data.df.PCoA<-Data.df.PCoA.eig$eig
 eig.Data.df.PCoA.sum<-sum(eig.Data.df.PCoA)
 a<-(eig.Data.df.PCoA/eig.Data.df.PCoA.sum)*100
 xlab<-paste("PC1","(",round(a[1],1),"%",")",sep="")
 ylab<-paste("PC2","(",round(a[2],1),"%",")",sep="")
```

```
  if(binary==TRUE){
    main<-"Sorensen PCoA"
  }else(main<-"Bray-Curtis PCoA")
  plot(Data.df.PCoA, col=Color,
       main=Title,xlab=xlab,ylab=ylab,pch=c(pch))
  return(Data.df.PCoA)
}
#sor
b<-as.factor(indices$BMI)
df.spe.bray.Sor<-Sor.bray.pcoa(abun, Dim = 2, Color = b, binary = TRUE)
ordiellipse(df.spe.bray.Sor,groups=b,col=c(1,2),lwd=1)
legend(-0.45,0.3,c("normal","obese", "overweight"), pch=21,col=1,pt.bg=c(1,2,3))
#text(df.spe.bray.Sor,labels=b,col=as.numeric(b))


#permanova
PERMANOVA<-function(abun,Group,binary,iters=9999){
  Data.Dist<-vegdist(abun,method="bray", binary=binary)
  adonis2(Data.Dist~Group,permutations=iters)
}
PERMDISP<-function(abun,Group,binary,iters=9999){
  Data.Dist<-vegdist(abun,method="bray", binary=binary)
  Data.betadisper<-betadisper(Data.Dist, group=Group)
  permutest(Data.betadisper, group=Group, permutations=iters)
}


PERMANOVA(presence2[28:143],b,TRUE,9999)
```

```
#p-value=0.1282

PERMDISP(presence2[28:143],b,TRUE,9999)

#0.2163


#####Smoking######

abundance$MOMSMOKE

#1 = yes

#2 = no

#9 = unknown

abundance$smoking<-as.factor(ifelse(abundance$MOMSMOKE== '1','smoking',

                        ifelse(abundance$MOMSMOKE== '2', 'non-smoking','non-smoking')))


indices$smoking<-abundance$smoking


###alpha diversity

#richness

table(indices$smoking)

boxplot<-c(indices$RichMGE,indices$Richamino,indices$RichMDR, indices$Richbeta,
indices$Richtetra,  indices$RichMLSB, indices$Richsulfo, indices$Richvanco,
indices$Richfluoro)

smoking<-rep(indices$smoking,9)

type<-
rep(c("MGE","aminoglycoside","MDR","betalactamase","tetracycline","MLSB","sulfonamide","
vancomycin","fluoroquinolone"),each=200)

length(boxplot)


boxplot2<-data.frame(boxplot,type,smoking)
```

```
ab<-ggplot(boxplot2, aes(x = type, y = boxplot, fill=smoking)) +ylim(0,30)+
geom_boxplot(width=0.4,position=position_dodge(0.4)) + theme_minimal() +
theme(axis.text.x = element_text(angle = 90)) + labs(title = "", y = "Richness", x = "") +
stat_compare_means(aes(group = smoking),label="p.signif") +
scale_fill_manual(values=c("#E69F00", "#00AFBB", "#E7B800", "#FC4E07",
"#00FF00","#00FF06"))+ theme(legend.position="top",)

ab

#ggsave("Richnesssmoking60All.tiff", units="in", width=7.5, height=5, dpi=300)

#significant in beta, flouro, MDR, MGE, MLSB, and tetry class

table(indices$smoking)


wilcox.test(RichARG~smoking, data=indices)

#p-value = 0.0007762

wilcox.test(RichMGE~smoking, data=indices)

#p-value = 8.883e-05


#Richness

b<-ggboxplot(data=indices, x="smoking", y="RichARG", color="black",
fill="smoking",palette="d3", ylab="Richness", xlab="", outlier.colour = "black", outlier.shape
= 1, main="Rich ARG")+ theme_classic() + guides(fill=FALSE) + coord_cartesian(ylim = c(0,
100))+ stat_compare_means(label.x=1.3, label.y=95)

b


a<-ggboxplot(data=indices, x="smoking", y="RichMGE", color="black",
fill="smoking",palette="d3", ylab="Richness", xlab="", outlier.colour = "black", outlier.shape
= 1, main="Rich MGE")+ theme_classic() + guides(fill=FALSE) + coord_cartesian(ylim = c(0,
40))+ stat_compare_means(label.x=1.7, label.y=35)

a
```

```
#combine these

figure <- ggarrange(a, b,labels = c("A", "B", ncol = 2, nrow = 1))

figure

#ggexport(figure, filename = "RichMGE_ARG_All.pdf")



##shan tests

smoking<-indices$smoking

ShannonMGERA<-indices$ShannonMGERA

wilcox.test(ShannonMGERA~smoking, data=indices)

#p-value = 0.01831

wilcox.test(ShannonARGRA~smoking, data=indices)

#p-value =0.3076


#shannon

d<-ggboxplot(data=indices, x="smoking", y="ShannonARGRA", color="black",
fill="smoking",palette="d3", ylab="Shannon Diversity Index", xlab="", outlier.colour =
"black", outlier.shape = 1, main="Shannon ARG")+ theme_classic() + guides(fill=FALSE) +
coord_cartesian(ylim = c(0, 4.0))+ stat_compare_means(label.x=1.3, label.y=3.8)

d

c<-ggboxplot(data=indices, x="smoking", y="ShannonMGERA", color="black",
fill="smoking",palette="d3", ylab="Shannon Diversity Index", xlab="", outlier.colour =
"black", outlier.shape = 1, main="Shannon MGE")+ theme_classic() + guides(fill=FALSE) +
coord_cartesian(ylim = c(0, 4.0))+ stat_compare_means(label.x=1.3, label.y=3.8)

c


#combine all 4
```

```
figure <- ggarrange(a, b, c, d, labels = c("A", "B", "C","D", ncol = 2, nrow = 2))

figure

ggexport(figure, filename = "ShannonMGE_ARG_smoking60.tiff")



#stacked box plot

boxplot<-c(indices$ShannonMGERA,indices$ShannonaminoRA,indices$ShannonMDRRA,
indices$ShannonbetaRA, indices$ShannontetraRA,  indices$ShannonMLSBRA,
indices$ShannonsulfoRA, indices$ShannonvancoRA, indices$ShannonfluoroRA)

smoking<-rep(indices$smoking,9)

type<-
rep(c("MGE","aminoglycoside","MDR","betalactamase","tetracycline","MLSB","sulfonamide","
vancomycin","fluoroquinolone"),each=200)

length(boxplot)


boxplot2<-data.frame(boxplot,type,smoking)


ab<-ggplot(boxplot2, aes(x = type, y = boxplot, fill=smoking)) +ylim(0,4)+
geom_boxplot(width=0.4,position=position_dodge(0.4)) + theme_minimal() +
theme(axis.text.x = element_text(angle = 90)) + labs(title = "", y = "Shannon Diversity Index ",
x = "") + stat_compare_means(aes(group = smoking),label="p.signif") +
scale_fill_manual(values=c("#E69F00", "#56B4E9", "#FC4E07","#00FF00"))+
theme(legend.position="top",)

ab

#fluro, MDR, MGE

#flouro, MDR sig

#ggsave("ShanAbudancessmoking_60.tiff", units="in", width=7.5, height=5, dpi=300)
```

```
#stacked bar plot

sumabundance<-aggregate(boxplot~type+smoking, boxplot2,median)

stackplot<-ggplot(data = sumabundance, aes(x = smoking, y=boxplot)) +
geom_bar(stat="identity", aes(fill=type))+theme_classic() +
scale_fill_brewer(palette="Paired")+ labs(title = "", y = "Shannon Index", x = "")+theme
(axis.text = element_text(size=14), axis.title=element_text(size=14))

stackplot


#inv simp

wilcox.test(SimpsonMGERA~smoking, data=indices)

#p-value = 0.01441

wilcox.test(SimpsonARGRA~smoking, data=indices)

# p-value =  0.927


#inv simp-- boxplots

e<-ggboxplot(data=indices, x="smoking", y="SimpsonMGERA", color="black",
fill="smoking",palette="d3", ylab="Inverse Simpson Diversity Index", xlab="", outlier.colour =
"black", outlier.shape = 1, main="Inverse Simpson MGE")+ theme_classic() +
guides(fill=FALSE) + coord_cartesian(ylim = c(0, 8.0))+ stat_compare_means(label.x=1.3,
label.y=7.5)

e

f<-ggboxplot(data=indices, x="smoking", y="SimpsonARGRA", color="black",
fill="smoking",palette="d3", ylab="Inverse Simpson Diversity Index", xlab="", outlier.colour =
"black", outlier.shape = 1, main="Inverse Simpson ARG")+ theme_classic() +
guides(fill=FALSE) + coord_cartesian(ylim = c(0, 21.0))+ stat_compare_means(label.x=1.3,
label.y=20.5)

f


#combine all 4
```

```
figure <- ggarrange(c, d, e, f, labels = c("A", "B", "C","D", ncol = 2, nrow = 2))
figure


###beta diversity
#ARG
abun<-abundance2[1:27]
abundance2$ABX<-indices$ABX
abundance2_omit<-abundance2 %>% drop_na(ABX)
indices_omit<-indices %>% drop_na(ABX)
presence2$ABX<-indices$ABX
presence2_omit<-presence2 %>% drop_na(ABX)


indices<-indices_omit
presence2<-presence2_omit
abundance2<-abundance2_omit


Sor.bray.pcoa<-function(abun,Dim=2,Color=1,binary,pch=16,Title="Bray-Curtis MGE"){
 Data.df<-vegdist(abun,method="bray", binary)
 Data.df.PCoA<-cmdscale(Data.df, k = Dim, eig = FALSE)
 Data.df.PCoA.eig<-cmdscale(Data.df, k = Dim, eig = TRUE)
 eig.Data.df.PCoA<-Data.df.PCoA.eig$eig
 eig.Data.df.PCoA.sum<-sum(eig.Data.df.PCoA)
 a<-(eig.Data.df.PCoA/eig.Data.df.PCoA.sum)*100
 xlab<-paste("PC1","(",round(a[1],1),"%",")",sep="")
 ylab<-paste("PC2","(",round(a[2],1),"%",")",sep="")
```

```
  if(binary==TRUE){
    main<-"Sorensen PCoA"
  }else(main<-"Bray-Curtis PCoA")
  plot(Data.df.PCoA, col=Color,
       main=Title,xlab=xlab,ylab=ylab,pch=c(pch))
  return(Data.df.PCoA)
}

#bray curtis
b<-as.factor(indices$smoking)
df.spe.bray.Sor<-Sor.bray.pcoa(abun, Dim = 2, Color = b, binary = FALSE)
ordiellipse(df.spe.bray.Sor,groups=b,col=c(1,2),lwd=1)
legend(0.30,0.2,c("non-smoking","smoking"), pch=21,col=1,pt.bg=c(1,2,3))
#text(df.spe.bray.Sor,labels=b,col=as.numeric(b))
#black= non-smoking, red = smoking

#permanova
PERMANOVA<-function(abun,Group,binary,iters=9999){
  Data.Dist<-vegdist(abun,method="bray", binary=binary)
  adonis2(Data.Dist~Group,permutations=iters)
}
PERMDISP<-function(abun,Group,binary,iters=9999){
  Data.Dist<-vegdist(abun,method="bray", binary=binary)
  Data.betadisper<-betadisper(Data.Dist, group=Group)
  permutest(Data.betadisper, group=Group, permutations=iters)
}
```

```
PERMANOVA(abundance2[,c(1:27)],b,FALSE,9999)
#p-value=0.0615 .
PERMDISP(abundance2[,c(1:27)],b,FALSE,9999)
#0.7147

#Sorensen
abun<-presence2[1:27]
Sor.bray.pcoa<-function(abun,Dim=2,Color=1,binary,pch=16,Title="Sorensen MGE"){
 Data.df<-vegdist(abun,method="bray", binary)
 Data.df.PCoA<-cmdscale(Data.df, k = Dim, eig = FALSE)
 Data.df.PCoA.eig<-cmdscale(Data.df, k = Dim, eig = TRUE)
 eig.Data.df.PCoA<-Data.df.PCoA.eig$eig
 eig.Data.df.PCoA.sum<-sum(eig.Data.df.PCoA)
 a<-(eig.Data.df.PCoA/eig.Data.df.PCoA.sum)*100
 xlab<-paste("PC1","(",round(a[1],1),"%",")",sep="")
 ylab<-paste("PC2","(",round(a[2],1),"%",")",sep="")
 if(binary==TRUE){
  main<-"Sorensen PCoA"
 }else(main<-"Bray-Curtis PCoA")
 plot(Data.df.PCoA, col=Color,
    main=Title,xlab=xlab,ylab=ylab,pch=c(pch))
 return(Data.df.PCoA)
}
#sor
b<-as.factor(indices$smoking)
```

```
df.spe.bray.Sor<-Sor.bray.pcoa(abun, Dim = 2, Color = b, binary = TRUE)
ordiellipse(df.spe.bray.Sor,groups=b,col=c(1,2),lwd=1)
legend(-0.25,-0.3,c("non-smoking","smoking"), pch=21,col=1,pt.bg=c(1,2))
#text(df.spe.bray.Sor,labels=b,col=as.numeric(b))

#permanova
PERMANOVA<-function(abun,Group,binary,iters=9999){
  Data.Dist<-vegdist(abun,method="bray", binary=binary)
  adonis2(Data.Dist~Group,permutations=iters)
}
PERMDISP<-function(abun,Group,binary,iters=9999){
  Data.Dist<-vegdist(abun,method="bray", binary=binary)
  Data.betadisper<-betadisper(Data.Dist, group=Group)
  permutest(Data.betadisper, group=Group, permutations=iters)
}

PERMANOVA(presence2[1:27],b,TRUE,9999)
#p-value=   4e-04 ***
PERMDISP(presence2[1:27],b,TRUE,9999)
#2e-04 ***

######Mom abx in pregnancy #######

meta<-read.xlsx("2023APR24_AMR_3mos_MetaData_plusanti.xlsx")
```

```
#make matching presence

df6<-meta[,c(1,148:241)]


pres.metadata<-inner_join(presence3mos, df6, by='ID')


####doing alpha/beta diveristy by different variables

presence<-pres.metadata

presence2<- presence[,4:146]

presence2


#make sure its the right columns!!!!!

abundance<-meta

abundance2<- abundance[,4:146]

head(abundance2)

abundance2[is.na(abundance2)]<- 0

presence2[is.na(presence2)]<- 0


abundance$first_antityp


######Contingency Tables######

# 11-MDR

# 81-MDR

# 1300-MDR

# 1513-Tetracycline

# 185-Tetracycline

# 191-Tetracycline
```

```
# 1511 - MLSB

# 206 - MGE



#trying to make this work

# create the wage_cat variable which takes two values

# such as Above if the wage is above median and Below if

# the wage is below median

meta<-metadata

meta$firstanti<-meta$first_antityp

temp3<-meta

meta$firstanti

#

names(temp3)[names(temp3) == '11'] <- 'eleven.g'

temp3$eleven.g

#tempT<-presence2

#names(tempT)[names(tempT) == '11'] <- 'eleven.gene'


#make it a numeric factor

eleven<-as.numeric(as.character(temp3))

temp3$eleven.gene<-as.numeric(temp3$eleven.g)

is.numeric(temp3$eleven.g)

temp3$eleven.g


summary(temp3$eleven.g)

#median: 1.586e-02
```

```
temp3$gene11<-as.factor(ifelse(temp3$eleven.g > 1.402e-02,"gene11","less gene11"))

temp3$gene11

levels(temp3$gene11)

temp3$first_antityp



temp3$first_antityp

temp3$nitro<-as.factor(ifelse(temp3$first_antityp =="nitrofuran","nitrofuran","no nitrofuran"))

temp3$nitro

table(temp3$gene11, temp3$nitro)



# Examine the Wage vs Job Class

# you could use also the command xtabs(~jobclass+wage_cat, data=Wage)

# con1<-table(temp3$,Wage$wage_cat)

# con1



#####using 3mos SIF in order to analyze the rest of the variables of interest (ie food patterns)

SIF<-read.xlsx("MARCH3mos_SIF_MR.xlsx")

names(SIF)[names(SIF) == 'Study.ID'] <- 'match'

SIF<-SIF[,-c(11:12)]
```

```
#joining them together

abundance3mos<-read.xlsx("2023APR18_abundance3mos_plusmoms_MR.xlsx")

presence3mos<-read.xlsx("2023APR18_presence3mos_plusmoms_MR.xlsx")

abun3mos<-abundance3mos[grep("3mos", abundance3mos$Timepoint), ]


bcdata$match


SIF$match

abun3mos$match


names(SIF)[names(SIF) == 'Study.ID'] <- 'match'

SIF<-SIF[,-c(11:12)]


SIFabun2<-inner_join(bcdata, SIF, by="match")

df1 <- SIFabun2 %>% distinct(match, .keep_all = TRUE)

df1


table(df1$`Has.baby.had.antibiotics.since.birth?`)

# table(df1$`Has.baby.had.antibiotics.since.birth?`)

table(df1$`During.the.past.week,.my.baby.ate:`)

table(df1$`During.the.past.week,.my.baby.ate:`)


df1$foodstatus<-gsub(".*100% breast.*", "100%",

                gsub(".*100% formula.*", "<20%",

                   gsub(".*20-50%.*", "50-80%",
```

```
                        gsub(".*50%.*", "50-80%",
                          gsub(".*20% breast milk.*", "<20%",
                            gsub(".*80% breast milk.*", "50-80%",
df1$`During.the.past.week,.my.baby.ate:`))))))


df1$foodstatus

table(df1$foodstatus)

#make presence

df6<-df1[,c(1,147:208)]

pres_sif<-inner_join(presence3mos, df6, by='ID')


df6<-df1[,c(1,147:208)]


abundance<-df1

tail(abundance)

presence<-pres_sif


#indices

indices <- abundance[,c("ID", "class","pair", "foodstatus")]

head(indices)

#drop NA

indices_omit<-indices %>% drop_na(foodstatus)

indices<-indices_omit

tail(indices)


df3<-abundance %>%
```

```
  filter(ID %in% indices$ID)
abundance<-df3

df4<-presence %>%
  filter(ID %in% indices$ID)
abundance<-df3
presence<-df4

abundance2<- abundance[,4:146]
head(abundance2)
tail(abundance2)



#list of genes names


#set NAs to 0
abundance2[is.na(abundance2)]<- 0

abundance$SumMGE<-rowSums(abundance2[,1:27])
abundance$Sumamino<-rowSums(abundance2[,28:48])
abundance$SumMDR<-rowSums(abundance2[,49:69])
abundance$Sumbeta<-rowSums(abundance2[,70:85])
abundance$Sumtetra<-rowSums(abundance2[,86:98])
abundance$SumMLSB<-rowSums(abundance2[,99:116])
abundance$Sumsulfo<-rowSums(abundance2[,117:121])
abundance$Sumvanco<-rowSums(abundance2[,122:129])
```

```
abundance$Sumfluoro<-rowSums(abundance2[,130:133])
abundance$Sumother<-rowSums(abundance2[,134:143])
abundance$SumARG<-rowSums(abundance2[,28:143])


#reassign variables
presence2<- presence[,4:146]
head(presence2)


##alpha diversity analysis
indices$RichMGE<-rowSums(presence2[,1:27])
summary(indices$RichMGE)
dunnTest(RichMGE ~ foodstatus,
     data=indices,
     method="bonferroni")
#
indices$Richamino <- rowSums(presence2[,28:48])
summary(indices$Richamino)
indices$RichMDR<-rowSums(presence2[,49:69])
summary(indices$RichMDR)
indices$Richbeta<-rowSums(presence2[,70:85])
summary(indices$Richbeta)
indices$Richtetra<-rowSums(presence2[,86:98])
summary(indices$Richtetra)
indices$RichMLSB<-rowSums(presence2[,99:116])
```

```
summary(indices$RichMLSB)

indices$Richsulfo<-rowSums(presence2[,117:121])

summary(indices$Richsulfo)

indices$Richvanco<-rowSums(presence2[,122:129])

summary(indices$Richvanco)

indices$Richfluoro<-rowSums(presence2[,130:133])

summary(indices$Richfluoro)

indices$Richother<-rowSums(presence2[,134:143])

summary(indices$Richother)

indices$RichARG<-rowSums(presence2[,28:143])


table(indices$RichMGE)




#n=195

#stacked box plot

indices$foodstatus

indices$foodstatus2<-as.factor(indices$foodstatus)


indices$foodstatus

indices$foodstatus2<-factor(indices$foodstatus2,levels(indices$foodstatus2)[c(1,3,2)])

indices$foodstatus2

indices$foodstatus<-indices$foodstatus2
```

```
boxplot_food_rich<-c(indices$RichMGE,indices$Richamino,indices$RichMDR,
indices$Richbeta, indices$Richtetra,  indices$RichMLSB, indices$Richsulfo,
indices$Richvanco, indices$Richfluoro)

foodstatus<-rep(indices$foodstatus,9)

type<-
rep(c("MGE","aminoglycoside","MDR","betalactamase","tetracycline","MLSB","sulfonamide","
vancomycin","fluoroquinolone"),each=195)

length(boxplot_food_rich)


boxplot2_food_rich<-data.frame(boxplot,type,foodstatus)


#dev.off()

rich_class3<-ggplot(boxplot2_food_rich, aes(x = type, y = boxplot_food_rich,
fill=foodstatus)) +ylim(0,30)+ geom_boxplot(width=0.4,position=position_dodge(0.4)) +
theme_minimal() + theme(axis.text.x = element_text(size=10,angle = 45)) + labs(title = "", y =
"Richness", x = "") + stat_compare_means(aes(group = foodstatus),label="p.signif") +
scale_fill_manual(values=c("#FFD700", "#0000FF", "#FFAEB9"))+
theme(legend.position="top",)

rich_class3

#ggsave("2023DEC05_Richness_foodstatus_SF12_MR.tiff", units="in", width=7.5, height=5,
dpi=300)

#2023APR28_RichnessClass_MatchedPairs_3mos_abxclass_MR


kruskal.test(Richtetra~foodstatus, data=indices)

#p-value = 9.047e-08

kruskal.test(RichMGE~foodstatus, data=indices)

# p-value = 7.003e-08

#beta, fluro, MDR, MGE, MLSB, tetra
```

```
#load library

library(FSA)

#install.packages("FSA")

dunnTest(Richamino ~ foodstatus,
        data=indices,
        method="bonferroni")



#Richness

b<-ggboxplot(data=indices, x="foodstatus", y="RichARG", color="black", fill="foodstatus",palette=c("#FFD700", "#0000FF", "#FFAEB9"), ylab="Richness", xlab="", outlier.colour = "black", outlier.shape = 1, main="Rich ARG")+ theme_classic() + guides(fill=FALSE) + coord_cartesian(ylim = c(0, 100))+ stat_compare_means(label.x=1.3, label.y=95)

b


a<-ggboxplot(data=indices, x="foodstatus", y="RichMGE", color="black", fill="foodstatus",palette=c("#FFD700", "#0000FF", "#FFAEB9"), ylab="Richness", xlab="", outlier.colour = "black", outlier.shape = 1, main="Rich MGE")+ theme_classic() + guides(fill=FALSE) + coord_cartesian(ylim = c(0, 40))+ stat_compare_means(label.x=1.7, label.y=35)

a


#combine these

figure <- ggarrange(a, b,labels = c("A", "B", ncol = 2, nrow = 1))

figure

#ggexport(figure, filename = "RichMGE_ARG_matchedpairs.tiff")
```

```
#SHANNON INDEX

#shannon diversity index as default = Relative abundance

indices$ShannonMGERA<-diversity(abundance2[,1:27])

indices$ShannonaminoRA <- diversity(abundance2[,28:48])

indices$ShannonMDRRA<-diversity(abundance2[,49:69])

indices$ShannonbetaRA<-diversity(abundance2[,70:85])

indices$ShannontetraRA<-diversity(abundance2[,86:98])

indices$ShannonMLSBRA<-diversity(abundance2[,99:116])

indices$ShannonsulfoRA<-diversity(abundance2[,117:121])

indices$ShannonvancoRA<-diversity(abundance2[,122:129])

indices$ShannonfluoroRA<-diversity(abundance2[,130:133])

indices$ShannonotherRA<-diversity(abundance2[,134:143])

indices$ShannonARGRA<-diversity(abundance2[,28:143])



levels(indices$foodstatus)

shan_food_20 <- subset(indices, (foodstatus == "<20%")) # subset

summary(shan_food_20$ShannontetraRA)

# Median Mean

# 0.83079 0.80696

shan_food_80 <- subset(indices, (foodstatus == "50-80%")) # subset

summary(shan_food_80$ShannontetraRA)

# > summary(shan_food_80$ShannontetraRA)
```

```
#  Median   Mean

# 0.47574 0.50918


head(shan_food_20)

shan_food_100 <- subset(indices, (foodstatus == "100%")) # subset

summary(shan_food_100$ShannontetraRA)

# > summary(shan_food_100$ShannontetraRA)

# Median    Mean

# 0.639373 0.663620

##shan tests

kruskal.test(ShannonMGERA~foodstatus, data=indices)

#p-value = 0.6626

kruskal.test(ShannonARGRA~foodstatus, data=indices)

#p-value =  0.3777

kruskal.test(ShannonaminoRA~foodstatus, data=indices)

#0.009386

kruskal.test(ShannontetraRA~foodstatus, data=indices)

#p-value = 0.001913

dunnTest(ShannontetraRA ~ foodstatus,

      data=indices,

      method="bonferroni")



#shannon

d<-ggboxplot(data=indices, x="foodstatus", y="ShannonARGRA", color="black", fill="foodstatus",palette=c("#FFD700", "#0000FF", "#FFAEB9"), ylab="Shannon Diversity Index", xlab="", outlier.colour = "black", outlier.shape = 1, main="Shannon ARG")+
```

```
theme_classic() + guides(fill=FALSE) + coord_cartesian(ylim = c(0, 4.0))+
stat_compare_means(label.x=1.3, label.y=3.8)

d

c<-ggboxplot(data=indices, x="foodstatus", y="ShannonMGERA", color="black",
fill="foodstatus",palette=c("#FFD700", "#0000FF", "#FFAEB9"), ylab="Shannon Diversity
Index", xlab="", outlier.colour = "black", outlier.shape = 1, main="Shannon MGE")+
theme_classic() + guides(fill=FALSE) + coord_cartesian(ylim = c(0, 4.0))+
stat_compare_means(label.x=1.3, label.y=3.8)

c


#combine all 4

figure <- ggarrange(a, b, c, d, labels = c("A", "B", "C","D", ncol = 2, nrow = 2))

figure

#ggexport(figure, filename = "ShannonMGE_ARG_matchedpairs.pdf")

##2023APR28_Rich_shan_Class_MatchedPairs_3mos_MR



#stacked box plot

boxplot_shan_food<-
c(indices$ShannonMGERA,indices$ShannonaminoRA,indices$ShannonMDRRA,
indices$ShannonbetaRA, indices$ShannontetraRA,  indices$ShannonMLSBRA,
indices$ShannonsulfoRA, indices$ShannonvancoRA, indices$ShannonfluoroRA)

foodstatus<-rep(indices$foodstatus,9)

type<-
rep(c("MGE","aminoglycoside","MDR","betalactamase","tetracycline","MLSB","sulfonamide","
vancomycin","fluoroquinolone"),each=195)

length(boxplot)


boxplot2_shan_food<-data.frame(boxplot_shan_food,type,foodstatus)
```

shan_class3<-ggplot(boxplot2_shan_food, aes(x = type, y = boxplot_shan_food, fill=foodstatus)) +ylim(0,4)+ geom_boxplot(width=0.4,position=position_dodge(0.4)) + theme_minimal() + theme(axis.text.x = element_text(size=10, angle = 45)) + labs(title = "", y = "Shannon Diversity Index ", x = "") + stat_compare_means(aes(group = foodstatus),label="p.signif") + scale_fill_manual(values=c("#FFD700", "#0000FF", "#FFAEB9"))+ theme(legend.position="top",)

shan_class3

#ggsave("2023DEC05_Shan_foodstatus_S12b.tiff", units="in", width=7.5, height=5, dpi=300)

#2023APR28__shan_Class_MatchedPairs_ABXclass_3mos_MR

#amino, fluro, tetra

#stacked bar plot

sumabundance<-aggregate(boxplot~type+foodstatus, boxplot2,median)

stackplot<-ggplot(data = sumabundance, aes(x = foodstatus, y=boxplot)) + geom_bar(stat="identity", aes(fill=type))+theme_classic() + scale_fill_brewer(palette="Paired")+ labs(title = "", y = "Shannon Index", x = "")+theme (axis.text = element_text(size=14), axis.title=element_text(size=14))

stackplot

#2023APR28__Stackplot_shan_Class_MatchedPairs_ABXclass_3mos_MR

#ggexport(stackplot, filename = "/Users/madeleinerussell/Desktop/Comstock Lab/AbxResistance/ABXR_Ranalysis/ShannonDiv_ALL.pdf")

#INVERSE SIMPSON INDEX

#Inverse Inverse Simpson Diversity Index = Relative abundance

indices$SimpsonMGERA<-diversity(abundance2[,1:27], index="invsimpson")

```
indices$SimpsonaminoRA<-diversity(abundance2[,28:48], index="invsimpson")



#p-value= 0.007428
dunnTest(SimpsonaminoRA ~ foodstatus,
         data=indices,
         method="bonferroni")
indices$SimpsonMDRRA<-diversity(abundance2[,49:69], index="invsimpson")
indices$SimpsonbetaRA<-diversity(abundance2[,70:85], index="invsimpson")
indices$SimpsontetraRA<-diversity(abundance2[,86:98], index="invsimpson")
#0.0008188
dunnTest(SimpsontetraRA ~ foodstatus,
         data=indices,
         method="bonferroni")
indices$SimpsonMLSBRA<-diversity(abundance2[,99:116], index="invsimpson")

indices$SimpsonsulfoRA<-diversity(abundance2[,117:121], index="invsimpson")
indices$SimpsonvancoRA<-diversity(abundance2[,122:129], index="invsimpson")
indices$SimpsonfluoroRA<-diversity(abundance2[,130:133], index="invsimpson")
indices$SimpsonotherRA<-diversity(abundance2[,134:143], index="invsimpson")
indices$SimpsonARGRA<-diversity(abundance2[,28:143], index="invsimpson")




#inv simp
kruskal.test(SimpsonMGERA~foodstatus, data=indices)
```

```
#p-value = 0.5555

kruskal.test(SimpsonARGRA~foodstatus, data=indices)

#p-value = 0.6629


#invsimp

f<-ggboxplot(data=indices, x="foodstatus", y="SimpsonARGRA", color="black", fill="foodstatus",palette=c("#FFD700", "#0000FF", "#FFAEB9"), ylab="Inverse Simpson Diversity Index", xlab="", outlier.colour = "black", outlier.shape = 1, main="Inverse Simpson ARG")+ theme_classic() + guides(fill=FALSE) + coord_cartesian(ylim = c(0, 25.0))+ stat_compare_means(label.x=1.3, label.y=22.5)

f

e<-ggboxplot(data=indices, x="foodstatus", y="SimpsonMGERA", color="black", fill="foodstatus",palette=c("#FFD700", "#0000FF", "#FFAEB9"), ylab="Inverse Simpson Diversity Index", xlab="", outlier.colour = "black", outlier.shape = 1, main="Inverse Simpson MGE")+ theme_classic() + guides(fill=FALSE) + coord_cartesian(ylim = c(0, 5.0))+ stat_compare_means(label.x=1.3, label.y=4.5)

e


#combine all 4

figure <- ggarrange(a, b, c, d, e, f, labels = c("A", "B", "C", "D", "E", "F", ncol = 2, nrow = 3))

figure

#2023APR28_invsimp_Class_MatchedPairs_3mos_MR

#ggexport(figure, filename = "INVsimpMGE_ARG_matchedpairs.pdf")


boxplot<-c(indices$SimpsonMGERA,indices$SimpsonaminoRA,indices$SimpsonMDRRA, indices$SimpsonbetaRA, indices$SimpsontetraRA,  indices$SimpsonMLSBRA, indices$SimpsonsulfoRA, indices$SimpsonvancoRA, indices$SimpsonfluoroRA)

foodstatus<-rep(indices$foodstatus,9)
```

```
type<-
rep(c("MGE","aminoglycoside","MDR","betalactamase","tetracycline","MLSB","sulfonamide","
vancomycin","fluoroquinolone"),each=195)

length(boxplot)


boxplot2<-data.frame(boxplot,type,foodstatus)


inv_class3<-ggplot(boxplot2, aes(x = type, y = boxplot, fill=foodstatus)) +ylim(0,25)+
geom_boxplot(width=0.4,position=position_dodge(0.4)) + theme_minimal() +
theme(axis.text.x = element_text(size=10, angle = 45)) + labs(title = "", y = "Inverse Simpson
Diversity Index", x = "") + stat_compare_means(aes(group = foodstatus),label="p.signif") +
scale_fill_manual(values=c("#FFD700", "#0000FF", "#FFAEB9"))+
theme(legend.position="top",)

inv_class3




library(ggplot2)

library(dplyr)

library(ggpubr)

library(dplyr)

library(ggplot2)


# --- Make sure df_simp is built as before ---

library(dplyr)

library(ggplot2)


# --- Build df_simp ---

df_simp <- data.frame(
```

```
  value = c(
    indices$SimpsonMGERA,
    indices$SimpsonaminoRA,
    indices$SimpsonMDRRA,
    indices$SimpsonbetaRA,
    indices$SimpsontetraRA,
    indices$SimpsonMLSBRA,
    indices$SimpsonsulfoRA,
    indices$SimpsonvancoRA,
    indices$SimpsonfluoroRA
  ),
  type = rep(c("MGE","aminoglycoside","MDR","betalactamase",
               "tetracycline","MLSB","sulfonamide","vancomycin","fluoroquinolone"),
             each = length(indices$foodstatus)),
  foodstatus = rep(indices$foodstatus, 9)
)

# --- Run KW per type manually ---
kw_tbl <- df_simp %>%
  group_by(type) %>%
  summarise(
    p = tryCatch(
      kruskal.test(value ~ foodstatus)$p.value,
      error = function(e) NA_real_
    ),
    .groups = "drop"
```

```
  ) %>%
  mutate(
    p_label = case_when(
      is.na(p) ~ "KW p = NA",
      p < 0.001 ~ "KW p < 0.001",
      TRUE ~ paste0("KW p = ", formatC(p, format = "f", digits = 3))
    )
  )

# --- Find label positions above boxes ---
y_top <- df_simp %>%
  group_by(type) %>%
  summarise(y = max(value, na.rm = TRUE), .groups = "drop")

kw_pos <- left_join(y_top, kw_tbl, by = "type") %>%
  mutate(y = y + 1)   # bump text above max

# --- Plot ---
inv_class3 <- ggplot(df_simp, aes(x = type, y = value, fill = foodstatus)) +
  geom_boxplot(width = 0.4, position = position_dodge(0.4)) +
  ylim(0,25) +
  theme_minimal() +
  theme(axis.text.x = element_text(size = 10, angle = 45, hjust = 1),
        legend.position = "top") +
  labs(y = "Inverse Simpson Diversity Index", x = "") +
  scale_fill_manual(values = c("#FFD700", "#0000FF", "#FFAEB9")) +
```

```
  geom_text(data = kw_pos,
            aes(x = type, y = y, label = p_label),
            inherit.aes = FALSE, size = 4.5, fontface = "bold")

inv_class3

#ggsave("2023DEC05_InvSimp_foodstatus_S12c2.tiff", units="in", width=7.5, height=5,
dpi=300)

#combine all 6
figure <- ggarrange(b, d, f, a, c, e, labels = c("A", "B","C","D"," E", "F", ncol = 2, nrow = 3))
figure

figure <- ggarrange(rich_class3, shan_class3, inv_class3, labels = c("A", "B","C"))
figure

#dev.off()
ggsave("2023OCT10_InvSimp_MGE_ARG_foodstatus_MR.tiff", units="in", width=7.5,
height=5, dpi=300)

##aug 25 2025
# --- Libraries ---
library(FSA)
library(rcompanion)
library(dplyr)
```

```
library(ggplot2)


library(FSA)

library(rcompanion)

library(dplyr)



# # --- Build dataframe ---

# boxplot_food_rich <- c(indices$RichMGE, indices$Richamino, indices$RichMDR,

#                   indices$Richbeta, indices$Richtetra, indices$RichMLSB,

#                   indices$Richsulfo, indices$Richvanco, indices$Richfluoro)

#

# foodstatus <- rep(indices$foodstatus, 9)

# type <- rep(c("MGE","aminoglycoside","MDR","betalactamase",

#            "tetracycline","MLSB","sulfonamide",

#            "vancomycin","fluoroquinolone"), each=length(indices$foodstatus))

#

# df <- data.frame(boxplot_food_rich, type, foodstatus)

#

# # --- Recode foodstatus to simpler labels for Dunn test ---

# food_map <- c("grp1", "grp2", "grp3")

# names(food_map) <- c("<20%", "50-80%", "100%")

#

# df <- df %>%

#   mutate(food_simple = food_map[foodstatus])

#
```

```
# # --- Function to get Dunn letters + KW p-values ---
# get_letters <- function(drug_class) {
#   sub_df <- df %>% filter(type == drug_class)
#
#   # Kruskal–Wallis
#   kw_res <- kruskal.test(boxplot_food_rich ~ food_simple, data=sub_df)
#
#   # Dunn test
#   dunn_out <- dunnTest(boxplot_food_rich ~ food_simple,
#                        data=sub_df, method="bonferroni")
#   dunn_res <- dunn_out$res
#
#   # Compact letter display
#   letters_df <- cldList(
#     P.adj ~ Comparison,
#     data = dunn_res,
#     threshold = 0.05
#   ) %>%
#     rename(food_simple = Group, Letters = Letter) %>%
#     mutate(foodstatus = names(food_map)[match(food_simple, food_map)],
#            type = drug_class,
#            KW_p = signif(kw_res$p.value, 3))
#
#   return(letters_df)
# }
#
```

```
# # --- Collect letters for all drug classes ---
# letters_all <- bind_rows(lapply(unique(df$type), get_letters))
#
# # --- Position letters above each box ---
# pos_df <- df %>%
#   group_by(foodstatus, type) %>%
#   summarise(y = max(boxplot_food_rich, na.rm=TRUE), .groups="drop") %>%
#   left_join(letters_all, by=c("foodstatus","type")) %>%
#   mutate(y = y + 2)
#
# # --- Create plot ---
# # Title with all KW p-values
# kw_summary <- letters_all %>%
#   group_by(type) %>%
#   summarise(KW_p = first(KW_p)) %>%
#   mutate(txt = paste0(type, " p=", KW_p)) %>%
#   pull(txt) %>%
#   paste(collapse=" | ")
#
# rich_class_all <- ggplot(df, aes(x = type, y = boxplot_food_rich, fill=foodstatus)) +
#   geom_boxplot(width=0.6, position=position_dodge(0.8)) +
#   theme_minimal() +
#   theme(axis.text.x = element_text(size=10, angle=45, hjust=1),
#       legend.position="top") +
#   labs(y = "Richness", x = "",
#      title = paste0("Kruskal–Wallis results: ", kw_summary)) +
```

```
#   scale_fill_manual(values=c("#FFD700", "#0000FF", "#FFAEB9")) +
#   geom_text(data = pos_df, aes(x = type, y = y, label = Letters, group=foodstatus),
#            inherit.aes = FALSE, size=4, fontface="bold",
#            position = position_dodge(0.8))
#
# print(rich_class_all)


library(ggplot2)
library(dplyr)
library(FSA)
library(rcompanion)

# --- Build dataframe ---
boxplot_food_rich <- c(indices$RichMGE, indices$Richamino, indices$RichMDR,
                   indices$Richbeta, indices$Richtetra, indices$RichMLSB,
                   indices$Richsulfo, indices$Richvanco, indices$Richfluoro)

foodstatus <- rep(indices$foodstatus, 9)
type <- rep(c("MGE","aminoglycoside","MDR","betalactamase",
          "tetracycline","MLSB","sulfonamide",
          "vancomycin","fluoroquinolone"), each=length(indices$foodstatus))

df <- data.frame(boxplot_food_rich, type, foodstatus)

# --- Recode foodstatus for Dunn test ---
```

```
# food_map <- c("grp1", "grp2", "grp3")
# names(food_map) <- c("<20%", "50-80%", "100%")
#
# df <- df %>%
#   mutate(food_simple = food_map[foodstatus])

# --- Recode foodstatus for Dunn test ---
food_map <- c("grp1", "grp2", "grp3")
names(food_map) <- c("<20%", "50-80%", "100%")

df <- df %>%
  mutate(
    food_simple = food_map[foodstatus],
    foodstatus = factor(foodstatus, levels = c("<20%", "50-80%", "100%")),
    food_simple = factor(food_simple, levels = c("grp1", "grp2", "grp3"))
  )

# # --- Function to get Dunn letters + KW p-value stars ---
# get_letters <- function(drug_class) {
#   sub_df <- df %>% filter(type == drug_class)
#
#   # Kruskal–Wallis
#   kw_res <- kruskal.test(boxplot_food_rich ~ food_simple, data=sub_df)
#   p <- kw_res$p.value
#   stars <- ifelse(p < 0.001, "***",
#                 ifelse(p < 0.01, "**",
```

```
#                    ifelse(p < 0.05, "*", "ns")))
#
#   # Dunn test
#   dunn_out <- dunnTest(boxplot_food_rich ~ food_simple,
#                  data=sub_df, method="bonferroni")
#   dunn_res <- dunn_out$res
#
#   # Compact letter display
#   letters_df <- cldList(
#    P.adj ~ Comparison,
#    data = dunn_res,
#    threshold = 0.05
#   ) %>%
#    rename(food_simple = Group, Letters = Letter) %>%
#    mutate(foodstatus = names(food_map)[match(food_simple, food_map)],
#         type = drug_class,
#         KW_p = p,
#         KW_stars = stars)
#
#   return(letters_df)
# }
#
# # --- Collect results ---
# letters_all <- bind_rows(lapply(unique(df$type), get_letters))
#
# # --- Position Dunn letters ---
```

```
# pos_df <- df %>%
#   group_by(foodstatus, type) %>%
#   summarise(y = max(boxplot_food_rich, na.rm=TRUE), .groups="drop") %>%
#   left_join(letters_all, by=c("foodstatus","type")) %>%
#   mutate(y = y + 2)
#
# # --- Position KW stars (per drug class, above all boxes) ---
# kw_pos <- df %>%
#   group_by(type) %>%
#   summarise(y = max(boxplot_food_rich, na.rm=TRUE) + 6, .groups="drop") %>%
#   left_join(
#     letters_all %>% dplyr::select(type, KW_stars) %>% distinct(),
#     by="type"
#   )
#
# # --- Global max y across the whole dataset ---
# y_max <- max(boxplot2_food_rich$boxplot_food_rich, na.rm = TRUE)
#
# # --- Adjust positions ---
# pos_df <- pos_df %>%
#   mutate(y = 0.90 * y_max)   # Dunn letters at 75% height, same for all classes
#
# kw_pos <- kw_pos %>%
#   mutate(y = y_max + 2)      # KW stars just above the tallest box
#
# # --- Only keep Dunn letters where KW is significant ---
```

```
# sig_classes <- kw_pos %>%
#   filter(KW_stars != "NS") %>%
#   pull(type)
#
# pos_df_sig <- pos_df %>%
#   filter(type %in% sig_classes)
#
# # --- Plot ---
# rich_class_all <- ggplot(df, aes(x = type, y = boxplot_food_rich, fill=foodstatus)) +
#   geom_boxplot(width=0.6, position=position_dodge(0.8)) +
#   theme_minimal() +
#   theme(axis.text.x = element_text(size=10, angle=45, hjust=1),
#         legend.position="top") +
#   labs(y = "Richness", x = "") +
#   scale_fill_manual(values=c("#FFD700", "#0000FF", "#FFAEB9")) +
#   # Dunn test letters
#   geom_text(data = pos_df_sig,
#             aes(x = type, y = y, label = Letters, group=foodstatus),
#             inherit.aes = FALSE, size = 4, fontface = "bold",
#             position = position_dodge(0.8)) +
#   # KW significance stars
#   geom_text(data = kw_pos,
#             aes(x = type, y = y, label = KW_stars),
#             inherit.aes = FALSE, size = 5, fontface = "bold")
#
# print(rich_class_all)
```

```
# --- Function to get Dunn letters + KW p-value stars ---
# --- Function to get Dunn letters + KW p-value stars ---
get_letters <- function(drug_class) {
 sub_df <- df %>% filter(type == drug_class)

 # Kruskal–Wallis
 kw_res <- kruskal.test(boxplot_food_rich ~ food_simple, data=sub_df)
 p <- kw_res$p.value
 stars <- ifelse(p < 0.001, "***",
         ifelse(p < 0.01, "**",
            ifelse(p < 0.05, "*", "NS")))

 if (stars == "NS") {
  # Return a record for KW only, no Dunn letters
  return(data.frame(
   food_simple = NA,
   Letters = NA,
   foodstatus = NA,
   type = drug_class,
   KW_p = p,
   KW_stars = stars,
   stringsAsFactors = FALSE
  ))
```

```
 }

 # Dunn test
 dunn_out <- dunnTest(boxplot_food_rich ~ food_simple,
                      data=sub_df, method="bonferroni")
 dunn_res <- dunn_out$res

 # Compact letter display
 letters_df <- cldList(
   P.adj ~ Comparison,
   data = dunn_res,
   threshold = 0.05
 ) %>%
   rename(food_simple = Group, Letters = Letter) %>%
   mutate(
     foodstatus = factor(names(food_map)[match(food_simple, food_map)],
                         levels = c("<20%", "50-80%", "100%")),
     type = drug_class,
     KW_p = p,
     KW_stars = stars
   )

 return(letters_df)
}
# --- Collect results (only significant Dunns kept) ---
letters_all <- bind_rows(lapply(unique(df$type), get_letters))
```

```
# --- Position Dunn letters ---
pos_df <- df %>%
  group_by(foodstatus, type) %>%
  summarise(y = max(boxplot_food_rich, na.rm=TRUE), .groups="drop") %>%
  left_join(letters_all, by=c("foodstatus","type")) %>%
  filter(!is.na(Letters)) %>%   # drop Dunns from NS classes
  mutate(y = 0.90 * max(df$boxplot_food_rich, na.rm=TRUE))

# --- KW stars ---
#--- Position KW stars (always keep NS + sig) ---
kw_pos <- df %>%
  group_by(type) %>%
  summarise(y = max(boxplot_food_rich, na.rm=TRUE) + 2, .groups="drop") %>%
  left_join(
    letters_all %>% dplyr::select(type, KW_stars) %>% distinct(),
    by="type"
  )

kw_pos <- kw_pos %>%
  mutate(y = y_max + 2)      # KW stars just above the tallest box



boxplot_food_rich
rich_class_all <- ggplot(df, aes(x = type, y = boxplot_food_rich, fill=foodstatus)) +
  geom_boxplot(width=0.6, position=position_dodge(0.8)) +
  theme_minimal() +
```

```
 theme(axis.text.x = element_text(size=10, angle=45, hjust=1),
    legend.position="top") +
 labs(y = "Richness", x = "") +
 scale_fill_manual(values=c("#FFD700", "#0000FF", "#FFAEB9")) +
 # Dunn test letters (only sig KW)
 geom_text(data = pos_df,
       aes(x = type, y = y, label = Letters, group=foodstatus),
       inherit.aes = FALSE, size = 4, fontface = "bold",
       position = position_dodge(0.8)) +
 # KW significance stars (always)
 geom_text(data = kw_pos,
       aes(x = type, y = y, label = KW_stars),
       inherit.aes = FALSE, size = 5, fontface = "bold")

print(rich_class_all)
#
# > dunnTest(Richbeta ~ foodstatus, data=indices, method="bonferroni")
##20-100 are difft
# Dunn (1964) Kruskal-Wallis multiple comparison
# p-values adjusted with the Bonferroni method.
# Comparison        Z     P.unadj       P.adj
# 1   <20% - 100%  4.849491 1.237785e-06 3.713355e-06
# 2 <20% - 50-80%  1.812127 6.996657e-02 2.098997e-01
# 3 100% - 50-80% -1.678782 9.319448e-02 2.795834e-01

# > dunnTest(Richfluoro ~ foodstatus, data=indices, method="bonferroni")
```

```
#20-100

# Dunn (1964) Kruskal-Wallis multiple comparison

# p-values adjusted with the Bonferroni method.

# Comparison         Z    P.unadj      P.adj

# 1   <20% - 100%  3.0441724 0.002333214 0.006999641


dunnTest(RichMGE ~ foodstatus, data=indices, method="bonferroni")

#20-100, 50-100

# > dunnTest(RichMDR ~ foodstatus, data=indices, method="bonferroni")

# Comparison         Z     P.unadj       P.adj

# 1   <20% - 100%  5.5883110 2.292887e-08 6.878661e-08

# 2 <20% - 50-80%  0.7587607 4.479957e-01 1.000000e+00

# 3 100% - 50-80% -3.2765016 1.051017e-03 3.153052e-03


# dunnTest(RichMLSB ~ foodstatus, data=indices, method="bonferroni")

#20-100

# Dunn (1964) Kruskal-Wallis multiple comparison

# p-values adjusted with the Bonferroni method.

# Comparison        Z     P.unadj       P.adj

# 1   <20% - 100%  5.599218 2.153203e-08 6.459608e-08

# 2 <20% - 50-80%  2.002176 4.526584e-02 1.357975e-01

# 3 100% - 50-80% -2.029273 4.243046e-02 1.272914e-01


#20-100

# > dunnTest(Richtetra ~ foodstatus, data=indices, method="bonferroni")

# Dunn (1964) Kruskal-Wallis multiple comparison
```

```
# p-values adjusted with the Bonferroni method.
# Comparison        Z     P.unadj       P.adj
# 1   <20% - 100%  5.371180 7.822309e-08 2.346693e-07
# 2 <20% - 50-80%  1.849862 6.433345e-02 1.930004e-01
# 3 100% - 50-80% -2.018065 4.358449e-02 1.307535e-01


library(ggplot2)
library(dplyr)
library(FSA)
library(rcompanion)

# ----------------
# SHANNON
# ----------------
df_shan <- data.frame(
  value = c(indices$ShannonMGERA, indices$ShannonaminoRA, indices$ShannonMDRRA,
        indices$ShannonbetaRA, indices$ShannontetraRA, indices$ShannonMLSBRA,
        indices$ShannonsulfoRA, indices$ShannonvancoRA, indices$ShannonfluoroRA),
  type = rep(c("MGE","aminoglycoside","MDR","betalactamase","tetracycline",
          "MLSB","sulfonamide","vancomycin","fluoroquinolone"),
         each = length(indices$foodstatus)),
  foodstatus = rep(indices$foodstatus, 9)
)
```

```
# Recode foodstatus

# food_map <- c("grp1","grp2","grp3")

# names(food_map) <- c("<20%", "50-80%", "100%")

# df_shan <- df_shan %>% mutate(food_simple = food_map[foodstatus])


# --- Recode foodstatus for Dunn test ---

food_map <- c("grp1", "grp2", "grp3")

names(food_map) <- c("<20%", "50-80%", "100%")


df_shan <- df_shan %>%

  mutate(

    food_simple = food_map[foodstatus],

    foodstatus = factor(foodstatus, levels = c("<20%", "50-80%", "100%")),

    food_simple = factor(food_simple, levels = c("grp1", "grp2", "grp3"))

  )


# --- Function (same as richness) ---

get_letters <- function(drug_class, df) {

  sub_df <- df %>% filter(type == drug_class)


  # Kruskal–Wallis

  kw_res <- kruskal.test(value ~ food_simple, data=sub_df)

  p <- kw_res$p.value

  stars <- ifelse(p < 0.001, "***",

                  ifelse(p < 0.01, "**",

                         ifelse(p < 0.05, "*", "NS")))
```

```
  if (stars == "NS") {
    return(data.frame(food_simple = NA, Letters = NA, foodstatus = NA,
                      type = drug_class, KW_p = p, KW_stars = stars,
                      stringsAsFactors = FALSE))
  }

  # Dunn
  dunn_out <- dunnTest(value ~ food_simple, data=sub_df, method="bonferroni")
  dunn_res <- dunn_out$res

  # CLD
  letters_df <- cldList(
    P.adj ~ Comparison,
    data = dunn_res,
    threshold = 0.05
  ) %>%
    rename(food_simple = Group, Letters = Letter) %>%
    mutate(
      foodstatus = factor(names(food_map)[match(food_simple, food_map)],
                          levels = c("<20%", "50-80%", "100%")),
      type = drug_class,
      KW_p = p,
      KW_stars = stars
    )
}
```

```
# Collect results
letters_all_shan <- bind_rows(lapply(unique(df_shan$type), get_letters, df=df_shan))

# Dunn positions
y_max_shan <- max(df_shan$value, na.rm=TRUE)
pos_df_shan <- df_shan %>%
 group_by(foodstatus, type) %>%
 summarise(y = max(value, na.rm=TRUE), .groups="drop") %>%
 left_join(letters_all_shan, by=c("foodstatus","type")) %>%
 filter(!is.na(Letters)) %>%
 mutate(y = 0.98 * y_max_shan)

# KW positions
kw_pos_shan <- df_shan %>%
 group_by(type) %>%
 summarise(y = max(value, na.rm=TRUE) + 2, .groups="drop") %>%
 left_join(letters_all_shan %>% dplyr::select(type, KW_stars) %>% distinct(),
      by="type") %>%
 mutate(y = y_max_shan + 0.5)

shan_plot <- ggplot(df_shan, aes(x=type, y=value, fill=foodstatus)) +
 geom_boxplot(width=0.6, position=position_dodge(0.8)) +
 theme_minimal() +
 theme(axis.text.x = element_text(size=10, angle=45, hjust=1),
    legend.position="top") +
```

```
 labs(y="Shannon", x="") +
 scale_fill_manual(values=c("#FFD700","#0000FF","#FFAEB9")) +
 geom_text(data=pos_df_shan,
       aes(x=type, y=y, label=Letters, group=foodstatus),
       inherit.aes=FALSE, size=4, fontface="bold",
       position=position_dodge(0.8)) +
 geom_text(data=kw_pos_shan,
       aes(x=type, y=y, label=KW_stars),
       inherit.aes=FALSE, size=5, fontface="bold")

print(shan_plot)

dunnTest(ShannontetraRA ~ foodstatus, data=indices, method="bonferroni")
#amino, fluoro, --20vs100, tetra - 20 vs. everybody



# ----------------
# INVERSE SIMPSON

indices$SimpsonMGERA
is.numeric(indices$SimpsonMGERA)
# ----------------
df_simp <- data.frame(
 value = c(indices$SimpsonMGERA, indices$SimpsonaminoRA, indices$SimpsonMDRRA,
       indices$SimpsonbetaRA, indices$SimpsontetraRA, indices$SimpsonMLSBRA,
```

```
      indices$SimpsonsulfoRA, indices$SimpsonvancoRA, indices$SimpsonfluoroRA),
 type = rep(c("MGE","aminoglycoside","MDR","betalactamase","tetracycline",
        "MLSB","sulfonamide","vancomycin","fluoroquinolone"),
       each = length(indices$foodstatus)),
 foodstatus = rep(indices$foodstatus, 9)
)

df_simp <- data.frame(
 value = c(
  unlist(indices$SimpsonMGERA),
  unlist(indices$SimpsonaminoRA),
  unlist(indices$SimpsonMDRRA),
  unlist(indices$SimpsonbetaRA),
  unlist(indices$SimpsontetraRA),
  unlist(indices$SimpsonMLSBRA),
  unlist(indices$SimpsonsulfoRA),
  unlist(indices$SimpsonvancoRA),
  unlist(indices$SimpsonfluoroRA)
 ),
 type = rep(c("MGE","aminoglycoside","MDR","betalactamase",
        "tetracycline","MLSB","sulfonamide","vancomycin","fluoroquinolone"),
       each = length(indices$foodstatus)),
 foodstatus = rep(indices$foodstatus, 9)
)

summary(indices$SimpsonaminoRA, foodstatus)
```

```
df_simp <- df_simp %>% mutate(food_simple = food_map[foodstatus])


df_simp <- df_simp %>%
  mutate(food_simple = food_map[foodstatus])


food_map <- c("grp1", "grp2", "grp3")
names(food_map) <- c("<20%", "50-80%", "100%")


df_simp <- df_simp %>%
  mutate(
    food_simple = food_map[foodstatus],
    foodstatus = factor(foodstatus, levels = c("<20%", "50-80%", "100%")),
    food_simple = factor(food_simple, levels = c("grp1", "grp2", "grp3"))
  )


#get letters function
get_letters <- function(drug_class, df) {
  sub_df <- df %>% filter(type == drug_class)


  # Kruskal–Wallis
  kw_res <- kruskal.test(value ~ food_simple, data=sub_df)
  p <- kw_res$p.value
  stars <- ifelse(p < 0.001, "***",
                  ifelse(p < 0.01, "**",
                         ifelse(p < 0.05, "*", "NS")))
```

```r
  if (stars == "NS") {
    return(data.frame(food_simple = NA, Letters = NA, foodstatus = NA,
                      type = drug_class, KW_p = p, KW_stars = stars,
                      stringsAsFactors = FALSE))
  }

  # Dunn
  dunn_out <- dunnTest(value ~ food_simple, data=sub_df, method="bonferroni")
  dunn_res <- dunn_out$res

  # CLD
  letters_df <- cldList(
    P.adj ~ Comparison,
    data = dunn_res,
    threshold = 0.05
  ) %>%
    rename(food_simple = Group, Letters = Letter) %>%
    mutate(
      foodstatus = factor(names(food_map)[match(food_simple, food_map)],
                          levels = c("<20%", "50-80%", "100%")),
      type = drug_class,
      KW_p = p,
      KW_stars = stars
    )
}
letters_all_simp <- bind_rows(lapply(unique(df_simp$type), get_letters, df=df_simp))
```

```
# Dunn positions
y_max_simp <- max(df_simp$value, na.rm=TRUE)
y_max_simp
# pos_df_simp <- df_simp %>%
#   group_by(foodstatus, type) %>%
#   summarise(y = max(value, na.rm=TRUE), .groups="drop") %>%
#   left_join(letters_all_simp, by=c("foodstatus","type")) %>%
#   filter(!is.na(Letters)) %>%
#   mutate(y = 0.98 * y_max_simp)
#
# # KW positions
# kw_pos_simp <- df_simp %>%
#   group_by(type) %>%
#   summarise(y = max(value, na.rm=TRUE) + 2, .groups="drop") %>%
#   left_join(letters_all_simp %>% dplyr::select(type, KW_stars) %>% distinct(),
#             by="type") %>%
#   mutate(y = y_max_simp + 0.5)



# --- Dunn test letters ---
pos_df_simp <- df_simp %>%
  group_by(foodstatus, type) %>%
  summarise(y = max(value, na.rm=TRUE), .groups="drop") %>%
  left_join(letters_all_simp %>% dplyr::select(type, foodstatus, Letters),
```

```
          by=c("foodstatus","type")) %>%
  filter(!is.na(Letters)) %>%
  mutate(y = 0.98 * y_max_simp)   # nudge slightly above each box

# --- KW results (per type only) ---
kw_pos_simp <- df_simp %>%
  group_by(type) %>%
  summarise(y = max(value, na.rm=TRUE), .groups="drop") %>%
  left_join(letters_all_simp %>% dplyr::select(type, KW_stars) %>% distinct(),
          by="type") %>%
  mutate(y = y_max_simp + 2)   # push above everything



simp_plot <- ggplot(df_simp, aes(x=type, y=value, fill=foodstatus)) +

  geom_boxplot(width=0.6, position=position_dodge(0.8)) +
  theme_minimal() +
  theme(axis.text.x = element_text(size=10, angle=45, hjust=1),
      legend.position="top") +
  labs(y="Inverse Simpson", x="") +
  scale_fill_manual(values=c("#FFD700","#0000FF","#FFAEB9")) +
  # Dunn letters
  geom_text(data=pos_df_simp,
          aes(x=type, y=y, label=Letters, group=foodstatus),
          inherit.aes=FALSE, size=4, fontface="bold",
          position=position_dodge(0.8)) +
```

```
  # KW stars
  geom_text(data=kw_pos_simp,
          aes(x=type, y=y, label=KW_stars),
          inherit.aes=FALSE, size=5, fontface="bold")
simp_plot

dunnTest(SimpsontetraRA ~ foodstatus, data=indices, method="bonferroni")
#amino - 20 vs. 100
#tetra - 20 vs. everybody

# simp_plot <- ggplot(df_simp, aes(x=type, y=value, fill=foodstatus)) +
#   geom_boxplot(width=0.6, position=position_dodge(0.8)) +
#   theme_minimal() +
#   theme(axis.text.x = element_text(size=10, angle=45, hjust=1),
#       legend.position="top") +
#   labs(y="Inverse Simpson", x="") +
#   scale_fill_manual(values=c("#FFD700","#0000FF","#FFAEB9")) +
#   geom_text(data=pos_df_simp,
#           aes(x=type, y=y, label=Letters, group=foodstatus),
#           inherit.aes=FALSE, size=4, fontface="bold",
#           position=position_dodge(0.8)) +
#   geom_text(data=kw_pos_simp,
#           aes(x=type, y=y, label=KW_stars),
#           inherit.aes=FALSE, size=5, fontface="bold")
#
# print(simp_plot)
```

```
# ----- Richness -----


# ----- Richness -----
rich_class_all2 <- rich_class_all +
 base_theme +
 labs(x = "", y = "Richness") +
 theme(
  axis.text.x = element_blank(),
  axis.title.x = element_blank(),
  axis.ticks.x = element_blank(),
  axis.text.y = element_text(size = 12),
  axis.title.y = element_text(size = 12, face = "bold")
 )
# ----- Shannon -----
shan_plot2 <- shan_plot +
 base_theme +
 labs(x = "", y = "Shannon") +
 theme(
  axis.text.x = element_blank(),
  axis.title.x = element_blank(),
  axis.ticks.x = element_blank(),
  axis.text.y = element_text(size = 12),
  axis.title.y = element_text(size = 12, face = "bold")
 )
```

```
# ----- Inverse Simpson (keep x-axis labels here) -----
# ----- Inverse Simpson (keep x-axis labels here) -----
simp_plot2 <- simp_plot +
  base_theme +
  labs(x = "", y = "Inverse Simpson") +
  theme(
    axis.text.x = element_blank(),
    axis.title.x = element_blank(),
    axis.ticks.x = element_blank(),
    axis.text.y = element_text(size = 12),
    axis.title.y = element_text(size = 12, face = "bold")
  )
simp_plot2
# ----- Combine vertically with shared legend -----
combined_div3 <- ggarrange(
  rich_class_all2, shan_plot2, simp_plot2,
  ncol = 1, nrow = 3,
  labels = c("C", "D", "E"),
  font.label = list(size = 12, face = "bold"),
  align = "v",
  common.legend = TRUE, legend = "none",
  heights = c(1, 1, 1)
)

print(combined_div3)
```

```
# ----- Dummy x-axis for ARG classes -----

drug_classes <- c("aminoglycoside","betalactamase","fluoroquinolone", "MDR", "MGE",
"MLSB", "sulfonamide",

          "tetracycline","vancomycin")


xlab_plot2 <- ggplot(data.frame(type = factor(drug_classes, levels = drug_classes), y = 0),

           aes(x = type, y = y)) +

 geom_blank() +

 labs(x = "ARG Class") +   # add label BEFORE theme

 theme_void() +

 theme(

  axis.text.x = element_text(size = 10, angle = 45, hjust = 0.5, vjust = 0.6),

  axis.ticks.x = element_blank()

 ) +

 labs(x = "ARG Class")



# ----- Stack plots + shared x labels -----

final_plot3 <- grid.arrange(combined_div3, xlab_plot2, heights = c(9, 1))


# # ----- Save publication-ready TIFF -----

# ggsave(

#   filename = "diversity_indices_food_shared_xaxis_labels.tiff",

#   plot = final_plot3,

#   device = "tiff",

#   width = 6.5, height = 9, units = "in",
```

```
#   dpi = 600,
#   compression = "lzw",
#   bg = "white"
# )

library(ggpubr)
library(ggplot2)

library(ggplot2)
library(dplyr)
library(ggpubr)
library(FSA)   # for dunnTest
library(rcompanion) # for cldList

# ---------------- Function ----------------
get_letters <- function(df, value_col, group_col = "foodstatus", label_y_shift = 2) {
  # explicit dplyr::recode
  mapping <- c("<20%" = "grp1", "50-80%" = "grp2", "100%" = "grp3")

  df <- df %>%
    mutate(group_simple = dplyr::recode(.data[[group_col]], !!!mapping))

  # Kruskal–Wallis
  kw_res <- kruskal.test(as.formula(paste(value_col, "~ group_simple")), data = df)
  p <- kw_res$p.value
  p_label <- paste0("KW p = ", signif(p, 3))
```

```
  # Dunn test
  dunn_out <- dunnTest(as.formula(paste(value_col, "~ group_simple")),
                  data = df, method = "bonferroni")
  dunn_res <- dunn_out$res

  # compact letter display
  letters_df <- cldList(
    P.adj ~ Comparison,
    data = dunn_res,
    threshold = 0.05
  ) %>%
    rename(group_simple = Group, Letters = Letter) %>%
    mutate(
      foodstatus = dplyr::recode(group_simple, "grp1" = "<20%", "grp2" = "50-80%", "grp3" =
"100%"),
      KW_p = p,
      KW_label = p_label,
      y = max(df[[value_col]], na.rm = TRUE) + label_y_shift
    )

  return(letters_df)
}

# ---------------- Data prep ----------------
# MGE
```

```
letters_mge <- get_letters(indices, "RichMGE", "foodstatus", label_y_shift = 3)

a <- ggplot(indices, aes(x = foodstatus, y = RichMGE, fill = foodstatus)) +
  geom_boxplot(color = "black", outlier.colour = "black", outlier.shape = 1) +
  scale_fill_manual(values = c("#FFD700", "#0000FF", "#FFAEB9")) +
  theme_classic(base_size = 12) +
  labs(y = "Richness", x = "Food status", title = "") +
  theme(legend.position = "") +
  # Dunn letters
  geom_text(data = letters_mge,
            aes(x = foodstatus, y = y, label = Letters),
            inherit.aes = FALSE, fontface = "bold", size = 5) +
  # KW p-value (top center)
  annotate("text", x = 2, y = max(indices$RichMGE, na.rm=TRUE) + 8,
           label = unique(letters_mge$KW_label),
           fontface = "bold", size = 5)

# ARG
letters_arg <- get_letters(indices, "RichARG", "foodstatus", label_y_shift = 3)

b <- ggplot(indices, aes(x = foodstatus, y = RichARG, fill = foodstatus)) +
  geom_boxplot(color = "black", outlier.colour = "black", outlier.shape = 1) +
  scale_fill_manual(values = c("#FFD700", "#0000FF", "#FFAEB9")) +
  theme_classic(base_size = 12) +
  labs(y = "Richness", x = "Food status", title = "") +
  theme(legend.position = "none") +
```

```
  # Dunn letters
  geom_text(data = letters_arg,
            aes(x = foodstatus, y = y, label = Letters),
            inherit.aes = FALSE, fontface = "bold", size = 5) +
  # KW p-value (top center)
  annotate("text", x = 2, y = max(indices$RichARG, na.rm=TRUE) + 8,
           label = unique(letters_arg$KW_label),
           fontface = "bold", size = 5)

# ---------------- Combine ----------------
figure <- ggarrange(b, a,
                    labels = c("A", "B"),
                    ncol = 2, nrow = 1,
                    common.legend = TRUE, legend = "top",
                    font.label = list(size = 14, face = "bold"))

print(figure)

dunnTest(RichMGE ~ foodstatus, data=indices, method="bonferroni")

library(ggpubr)

#if you want them with stars:

# Helper function to convert p-values to stars
p_to_stars <- function(p) {
```

```
  if (p <= 0.001) return("***")
  else if (p <= 0.01) return("**")
  else if (p <= 0.05) return("*")
  else return("ns")
}

# --- MGE ---
letters_mge <- get_letters(indices, "RichMGE", "foodstatus", label_y_shift = 3)
p_mge <- unique(letters_mge$KW_p)
p_mge_label <- p_to_stars(p_mge)

a <- ggplot(indices, aes(x = foodstatus, y = RichMGE, fill = foodstatus)) +
  geom_boxplot(color = "black", outlier.colour = "black", outlier.shape = 1) +
  scale_fill_manual(values = c("#FFD700", "#0000FF", "#FFAEB9")) +
  theme_classic(base_size = 12) +
  labs(y = "Richness", x = "", title = "") +
  theme(legend.position = "none") +
  # Dunn letters
  geom_text(data = letters_mge,
            aes(x = foodstatus, y = y, label = Letters),
            inherit.aes = FALSE, fontface = "bold", size = 5) +
  # KW significance stars
  annotate("text", x = 2, y = max(indices$RichMGE, na.rm=TRUE) + 8,
           label = p_mge_label, fontface = "bold", size = 6)

# --- ARG ---
```

```
letters_arg <- get_letters(indices, "RichARG", "foodstatus", label_y_shift = 3)
p_arg <- unique(letters_arg$KW_p)
p_arg_label <- p_to_stars(p_arg)

b <- ggplot(indices, aes(x = foodstatus, y = RichARG, fill = foodstatus)) +
  geom_boxplot(color = "black", outlier.colour = "black", outlier.shape = 1) +
  scale_fill_manual(values = c("#FFD700", "#0000FF", "#FFAEB9")) +
  theme_classic(base_size = 12) +
  labs(y = "Richness", x = "", title = "") +
  theme(legend.position = "none") +
  # Dunn letters
  geom_text(data = letters_arg,
            aes(x = foodstatus, y = y + 3, label = Letters),
            inherit.aes = FALSE, fontface = "bold", size = 5) +
  # KW significance stars
  annotate("text", x = 2, y = max(indices$RichARG, na.rm=TRUE) + 8,
           label = p_arg_label, fontface = "bold", size = 6)

# --- Combine panels ---
figure <- ggarrange(b, a,
                    labels = c("A", "B"),
                    ncol = 2, nrow = 1,
                    common.legend = TRUE, legend = "top",
                    font.label = list(size = 14, face = "bold"))

print(figure)
```

```
# --- First combine the two richness plots into one row ---
richness_panel <- ggarrange(
  b, a,
  labels = c("A", "B"),
  ncol = 2, nrow = 1,
  font.label = list(size = 12, face = "bold"),
  common.legend = TRUE, legend = "top"
)
richness_panel

richness_panel <- ggarrange(
  b, a,
  labels = c("A", "B"),
  ncol = 2, nrow = 1,
  font.label = list(size = 12, face = "bold"),
  common.legend = TRUE, legend = "top",
  label.x = 0.02,   # push labels a bit inside horizontally
  label.y = 0.98,   # push labels to the very top
  hjust = 0,        # left align
  vjust = 1         # top align
)

# --- Now combine with your 3-panel figure ---
final_pub_figure <- ggarrange(
```

```
  richness_panel,
  as_ggplot(final_plot3),   # wrap grid.arrange output
  ncol = 1, nrow = 2,
  heights = c(0.8, 2.2),
  labels = c("", ""),
  common.legend = TRUE, legend = "top"
)

final_pub_figure
# --- Save publication-ready figure ---
ggsave(
  filename = "final_5panel_publication2.tiff",
  plot = final_pub_figure,
  device = "tiff",
  width = 10, height = 12, units = "in",
  dpi = 600, compression = "lzw", bg = "white"
)

final_pub_figure
# --- Save publication-ready figure ---
ggsave(
  filename = "final_5panel_publication2.tiff",
  plot = final_pub_figure,
  device = "tiff",
  width = 6.5, height = 9, units = "in",
  dpi = 600,
```

```
  compression = "lzw",
  bg = "white"
)

# --- Combine with adjusted heights ---
final_pub_figure2 <- ggarrange(
  richness_panel,   # your 2-panel richness plots (A, B)
  as_ggplot(final_plot3),      # your 3-panel diversity plots (C–E)
  ncol = 1, nrow = 2,
  heights = c(0.3, 0.7),   # adjust ratio: top smaller, bottom larger
  labels = c("", ""),
  common.legend = TRUE, legend = "top"
)
final_pub_figure2

library(cowplot)

final_pub_figure3 <- plot_grid(
  richness_panel_tight,
  final_plot3_tight,
  ncol = 1,
  rel_heights = c(0.8, 2.2),  # adjust ratio
  align = "v"
)

final_pub_figure3
```

```r
library(gridExtra)
library(grid)

# Add tighter margins to each subplot before combining
richness_panel_tight <- richness_panel +
  theme(plot.margin = margin(0.5,0.5, 0.5, 0.5))   # top, right, bottom, left
richness_panel_tight

final_plot3_tight <- as_ggplot(final_plot3) +
  theme(plot.margin = margin(0.5,0.5, 0.5, 0.5))

# Combine with less vertical gap
final_pub_figure2 <- ggarrange(
  richness_panel_tight,
  final_plot3_tight,
  ncol = 1, nrow = 2,
  heights = c(0.3, 0.7),  # still bottom larger
  labels = c("", ""),
  common.legend = TRUE, legend = "top",
  align = "v"             # vertical alignment tight
)

final_pub_figure2
```

```
# --- Save publication-ready figure ---
ggsave(
  filename = "final_5panel_publication9.tiff",
  plot = final_pub_figure3,
  device = "tiff",
  width = 6.5, height = 9, units = "in",
  dpi = 600,
  compression = "lzw",
  bg = "white"
)


#####Beta Diversity####
table(indices$foodstatus)

presence2<- presence[,4:146]
presence2

abundance2<- abundance[,4:146]
abun<-abundance2[1:27]
head(abun)

#bray curtis
Sor.bray.pcoa<-function(abun,Dim=2,Color=1,binary,pch=16,Title="Bray-Curtis ARG"){
  Data.df<-vegdist(abun,method="bray", binary)
```

```
  Data.df.PCoA<-cmdscale(Data.df, k = Dim, eig = FALSE)
  Data.df.PCoA.eig<-cmdscale(Data.df, k = Dim, eig = TRUE)
  eig.Data.df.PCoA<-Data.df.PCoA.eig$eig
  eig.Data.df.PCoA.sum<-sum(eig.Data.df.PCoA)
  a<-(eig.Data.df.PCoA/eig.Data.df.PCoA.sum)*100
  xlab<-paste("PC1","(",round(a[1],1),"%",")",sep="")
  ylab<-paste("PC2","(",round(a[2],1),"%",")",sep="")
  if(binary==TRUE){
    main<-"Sorensen PCoA"
  }else(main<-"Bray-Curtis PCoA")
  plot(Data.df.PCoA, col=Color,
      main=Title,xlab=xlab,ylab=ylab,pch=c(pch))
  return(Data.df.PCoA)
}

abun<-abundance2[28:143]
b<-as.factor(abundance$foodstatus)
df.spe.bray.Sor<-Sor.bray.pcoa(abun, Dim = 2, Color = b, binary = FALSE)
ordiellipse(df.spe.bray.Sor,groups=b,col=c(1,3,2),lwd=1)
legend(-0.4,0.3,c(">20%","50-80%", "100%"), pch=21,col=1,pt.bg=c(1,3,2))
text(df.spe.bray.Sor,labels=b,col=as.numeric(b))

#permanova
PERMANOVA<-function(abun,Group,binary,iters=9999){
  Data.Dist<-vegdist(abun,method="bray", binary=binary)
  adonis2(Data.Dist~Group,permutations=iters)
```

```
}
PERMDISP<-function(abun,Group,binary,iters=9999){
 Data.Dist<-vegdist(abun,method="bray", binary=binary)
 Data.betadisper<-betadisper(Data.Dist, group=Group)
 permutest(Data.betadisper, group=Group, permutations=iters)
}


PERMANOVA(abundance2[,c(28:143)],b,FALSE,9999)
#p-value=  6e-04 ***
PERMDISP(abundance2[,c(28:143)],b,FALSE,9999)
#0.1532

#Sorensen
abun<-presence2[28:143]
Sor.bray.pcoa<-function(abun,Dim=2,Color=1,binary,pch=16,Title="Sorensen ARG"){
 Data.df<-vegdist(abun,method="bray", binary)
 Data.df.PCoA<-cmdscale(Data.df, k = Dim, eig = FALSE)
 Data.df.PCoA.eig<-cmdscale(Data.df, k = Dim, eig = TRUE)
 eig.Data.df.PCoA<-Data.df.PCoA.eig$eig
 eig.Data.df.PCoA.sum<-sum(eig.Data.df.PCoA)
 a<-(eig.Data.df.PCoA/eig.Data.df.PCoA.sum)*100
 xlab<-paste("PC1","(",round(a[1],1),"%",")",sep="")
 ylab<-paste("PC2","(",round(a[2],1),"%",")",sep="")
 if(binary==TRUE){
  main<-"Sorensen PCoA"
```

```
 }else(main<-"Bray-Curtis PCoA")
 plot(Data.df.PCoA, col=Color,
     main=Title,xlab=xlab,ylab=ylab,pch=c(pch))
 return(Data.df.PCoA)
}
#sor
b<-as.factor(abundance$foodstatus)
df.spe.bray.Sor<-Sor.bray.pcoa(abun, Dim = 2, Color = b, binary = TRUE)
ordiellipse(df.spe.bray.Sor,groups=b,col=c(1,3,2),lwd=1)
legend(-0.2,0.4,c(">20%","50-80%", "100%"), pch=21,col=1,pt.bg=c(1,3,2))
#text(df.spe.bray.Sor,labels=b,col=as.numeric(b))

#permanova
PERMANOVA<-function(abun,Group,binary,iters=9999){
 Data.Dist<-vegdist(abun,method="bray", binary=binary)
 adonis2(Data.Dist~Group,permutations=iters)
}
PERMDISP<-function(abun,Group,binary,iters=9999){
 Data.Dist<-vegdist(abun,method="bray", binary=binary)
 Data.betadisper<-betadisper(Data.Dist, group=Group)
 permutest(Data.betadisper, group=Group, permutations=iters)
}

PERMANOVA(presence2[,c(28:143)],b,TRUE,9999)
#1e-04 ***
PERMDISP(presence2[,c(28:143)],b,TRUE,9999)
```

```
#2e-04 ***



######GBS infections treated during pregnancy#######

table(abundance$INF_GB_STREP)

# 1; n = 24
#2; n = 103
#9 ; n = 3

indices$GBS<-gsub("1", "Yes",
          gsub("2", "No",
            gsub("9", 'NA', abundance$INF_GB_STREP)))

table(indices$GBS)

indices_omit<-indices[-grep("NA", indices$GBS), ]
indices<-indices_omit

df3<-abundance %>%
  filter(ID %in% indices$ID)
abundance<-df3

df4<-presence %>%
  filter(ID %in% indices$ID)
```

```
abundance<-df3

presence<-df4


#reassign variables

abundance2<- abundance[,4:146]

head(abundance2)


presence2<- presence[,4:146]

head(presence2)




#n=130

#stacked box plot

length(indices)


boxplot<-c(indices$RichMGE,indices$Richamino,indices$RichMDR, indices$Richbeta,
indices$Richtetra,  indices$RichMLSB, indices$Richsulfo, indices$Richvanco,
indices$Richfluoro)

GBS<-rep(indices$GBS,9)

type<-
rep(c("MGE","aminoglycoside","MDR","betalactamase","tetracycline","MLSB","sulfonamide","
vancomycin","fluoroquinolone"),each=192)

length(boxplot)


boxplot2<-data.frame(boxplot,type,GBS)
```

```
ab<-ggplot(boxplot2, aes(x = type, y = boxplot, fill=GBS)) +ylim(0,30)+
geom_boxplot(width=0.4,position=position_dodge(0.4)) + theme_minimal() +
theme(axis.text.x = element_text(size = 10, angle = 45)) + labs(title = "", y = "Richness", x = "")
+ stat_compare_means(aes(group = GBS),label="p.signif") +
scale_fill_manual(values=c("#EE0000", "#6C7B8B"))+ theme(legend.position="top",)

ab

#ggsave("RichnessClassAll.tiff", units="in", width=7.5, height=5, dpi=300)

#2023APR28_RichnessClass_MatchedPairs_3mos_abxclass_MR


wilcox.test(RichARG~GBS, data=indices)

#p-value = 0.3013

wilcox.test(RichMGE~GBS, data=indices)

# p-value = 0.5178


#Richness

b<-ggboxplot(data=indices, x="GBS", y="RichARG", color="black",
fill="GBS",palette=c("#EE0000", "#6C7B8B"), ylab="Richness", xlab="", outlier.colour =
"black", outlier.shape = 1, main="Rich ARG")+ theme_classic() + guides(fill=FALSE) +
coord_cartesian(ylim = c(0, 100))+ stat_compare_means(label.x=1.3, label.y=95)

b


a<-ggboxplot(data=indices, x="GBS", y="RichMGE", color="black",
fill="GBS",palette=c("#EE0000", "#6C7B8B"), ylab="Richness", xlab="", outlier.colour =
"black", outlier.shape = 1, main="Rich MGE")+ theme_classic() + guides(fill=FALSE) +
coord_cartesian(ylim = c(0, 40))+ stat_compare_means(label.x=1.7, label.y=35)

a


#combine these

figure <- ggarrange(a, b,labels = c("A", "B", ncol = 2, nrow = 1))
```

```
figure

#ggexport(figure, filename = "RichMGE_ARG_matchedpairs.tiff")


#Shannon

wilcox.test(ShannonMGERA~GBS, data=indices)

#p-value = 0.8841

wilcox.test(ShannonARGRA~GBS, data=indices)

#p-value = 0.8195

wilcox.test(ShannonaminoRA~GBS, data=indices)

#0.6485



#shannon

d<-ggboxplot(data=indices, x="GBS", y="ShannonARGRA", color="black",
fill="GBS",palette=c("#EE0000", "#6C7B8B"), ylab="Shannon Diversity Index", xlab="",
outlier.colour = "black", outlier.shape = 1, main="Shannon ARG")+ theme_classic() +
guides(fill=FALSE) + coord_cartesian(ylim = c(0, 4.0))+ stat_compare_means(label.x=1.3,
label.y=3.8)

d

c<-ggboxplot(data=indices, x="GBS", y="ShannonMGERA", color="black",
fill="GBS",palette=c("#EE0000", "#6C7B8B"), ylab="Shannon Diversity Index", xlab="",
outlier.colour = "black", outlier.shape = 1, main="Shannon MGE")+ theme_classic() +
guides(fill=FALSE) + coord_cartesian(ylim = c(0, 4.0))+ stat_compare_means(label.x=1.3,
label.y=3.8)

c


#combine all 4

figure <- ggarrange(a, b, c, d, labels = c("A", "B", "C","D", ncol = 2, nrow = 2))
```

```
figure

#ggexport(figure, filename = "ShannonMGE_ARG_matchedpairs.pdf")

##2023APR28_Rich_shan_Class_MatchedPairs_3mos_MR



#stacked box plot

boxplot<-c(indices$ShannonMGERA,indices$ShannonaminoRA,indices$ShannonMDRRA,
indices$ShannonbetaRA, indices$ShannontetraRA,  indices$ShannonMLSBRA,
indices$ShannonsulfoRA, indices$ShannonvancoRA, indices$ShannonfluoroRA)

GBS<-rep(indices$GBS,9)

type<-
rep(c("MGE","aminoglycoside","MDR","betalactamase","tetracycline","MLSB","sulfonamide","
vancomycin","fluoroquinolone"),each=192)

length(boxplot)


boxplot2<-data.frame(boxplot,type,GBS)


ab<-ggplot(boxplot2, aes(x = type, y = boxplot, fill=GBS)) +ylim(0,10)+
geom_boxplot(width=0.4,position=position_dodge(0.4)) + theme_minimal() +
theme(axis.text.x = element_text(size = 10, angle = 45)) + labs(title = "", y = "Shannon
Diversity Index ", x = "") + stat_compare_means(aes(group = GBS),label="p.signif") +
scale_fill_manual(values=c("#EE0000", "#6C7B8B"))+ theme(legend.position="top",)

ab

ggsave("SumShanAbudancesClass_matchedpairs.tiff", units="in", width=7.5, height=5,
dpi=300)

#2023APR28__shan_Class_MatchedPairs_ABXclass_3mos_MR
```

```
#stacked bar plot

sumabundance<-aggregate(boxplot~type+GBS, boxplot2,median)

stackplot<-ggplot(data = sumabundance, aes(x = GBS, y=boxplot)) +
geom_bar(stat="identity", aes(fill=type))+theme_classic() +
scale_fill_brewer(palette="Paired")+ labs(title = "", y = "Shannon Index", x = "")+theme
(axis.text = element_text(size=14), axis.title=element_text(size=14))

stackplot

#2023APR28__Stackplot_shan_Class_MatchedPairs_ABXclass_3mos_MR

#ggexport(stackplot, filename = "/Users/madeleinerussell/Desktop/Comstock
Lab/AbxResistance/ABXR_Ranalysis/ShannonDiv_ALL.pdf")

#invsimp

indices$SimpsonMGERA<-diversity(abundance2[,1:27], index="invsimpson")

indices$SimpsonaminoRA<-diversity(abundance2[,28:48], index="invsimpson")

#p-value= 0.007428

indices$SimpsonMDRRA<-diversity(abundance2[,49:69], index="invsimpson")

indices$SimpsonbetaRA<-diversity(abundance2[,70:85], index="invsimpson")

indices$SimpsontetraRA<-diversity(abundance2[,86:98], index="invsimpson")

#0.0008188

indices$SimpsonMLSBRA<-diversity(abundance2[,99:116], index="invsimpson")

indices$SimpsonsulfoRA<-diversity(abundance2[,117:121], index="invsimpson")

indices$SimpsonvancoRA<-diversity(abundance2[,122:129], index="invsimpson")

indices$SimpsonfluoroRA<-diversity(abundance2[,130:133], index="invsimpson")

indices$SimpsonotherRA<-diversity(abundance2[,134:143], index="invsimpson")
```

```
indices$SimpsonARGRA<-diversity(abundance2[,28:143], index="invsimpson")


wilcox.test(SimpsonMGERA~GBS, data=indices)

#p-value = 0.9949

wilcox.test(SimpsonARGRA~GBS, data=indices)

#p-value = 0.7974

wilcox.test(SimpsonfluoroRA~GBS, data=indices)

#0.005481

wilcox.test(SimpsonaminoRA~GBS, data=indices)

#0.4956




boxplot<-c(indices$SimpsonMGERA,indices$SimpsonaminoRA,indices$SimpsonMDRRA,
indices$SimpsonbetaRA, indices$SimpsontetraRA,  indices$SimpsonMLSBRA,
indices$SimpsonsulfoRA, indices$SimpsonvancoRA, indices$SimpsonfluoroRA)

GBS<-rep(indices$GBS,9)

type<-
rep(c("MGE","aminoglycoside","MDR","betalactamase","tetracycline","MLSB","sulfonamide","
vancomycin","fluoroquinolone"),each=191)

length(boxplot)


boxplot2<-data.frame(boxplot,type,GBS)


ab<-ggplot(boxplot2, aes(x = type, y = boxplot, fill=GBS)) +ylim(0,12)+
geom_boxplot(width=0.4,position=position_dodge(0.4)) + theme_minimal() +
theme(axis.text.x = element_text(size = 10, angle = 45)) + labs(title = "", y = "Inverse Simpson
```

```
Diversity Index", x = "") + stat_compare_means(aes(group = GBS),label="p.signif") + scale_fill_manual(values=c("#EE0000", "#6C7B8B"))+ theme(legend.position="top",)

ab


ggsave("2023DEC05_simp_GBS_SF9.tiff", units="in", width=7.5, height=5, dpi=300)


#beta diversity

abun<-abundance2[28:143]

head(abun)


#bray curtis

Sor.bray.pcoa<-function(abun,Dim=2,Color=1,binary,pch=16,Title="Bray-Curtis MGE"){

 Data.df<-vegdist(abun,method="bray", binary)

 Data.df.PCoA<-cmdscale(Data.df, k = Dim, eig = FALSE)

 Data.df.PCoA.eig<-cmdscale(Data.df, k = Dim, eig = TRUE)

 eig.Data.df.PCoA<-Data.df.PCoA.eig$eig

 eig.Data.df.PCoA.sum<-sum(eig.Data.df.PCoA)

 a<-(eig.Data.df.PCoA/eig.Data.df.PCoA.sum)*100

 xlab<-paste("PC1","(",round(a[1],1),"%",")",sep="")

 ylab<-paste("PC2","(",round(a[2],1),"%",")",sep="")

 if(binary==TRUE){

  main<-"Sorensen PCoA"

 }else(main<-"Bray-Curtis PCoA")

 plot(Data.df.PCoA, col=Color,

    main=Title,xlab=xlab,ylab=ylab,pch=c(pch))

 return(Data.df.PCoA)
```

```
}

#abun<-abundance2[28:143]
b<-as.factor(indices$GBS)
df.spe.bray.Sor<-Sor.bray.pcoa(abun, Dim = 2, Color = b, binary = FALSE)
ordiellipse(df.spe.bray.Sor,groups=b,col=c(1,2),lwd=1)
legend(-0.4,0.3,c("No","Yes"), pch=21,col=1,pt.bg=c(1,2))
text(df.spe.bray.Sor,labels=b,col=as.numeric(b))

#permanova
PERMANOVA<-function(abun,Group,binary,iters=9999){
 Data.Dist<-vegdist(abun,method="bray", binary=binary)
 adonis2(Data.Dist~Group,permutations=iters)
}
PERMDISP<-function(abun,Group,binary,iters=9999){
 Data.Dist<-vegdist(abun,method="bray", binary=binary)
 Data.betadisper<-betadisper(Data.Dist, group=Group)
 permutest(Data.betadisper, group=Group, permutations=iters)
}


PERMANOVA(abundance2[,c(28:143)],b,FALSE,9999)
#p-value=0.3711
PERMDISP(abundance2[,c(28:143)],b,FALSE,9999)
#0.3626
```

```
#Sorensen
abun<-presence2[28:143]
Sor.bray.pcoa<-function(abun,Dim=2,Color=1,binary,pch=16,Title="Sorensen ARG"){
 Data.df<-vegdist(abun,method="bray", binary)
 Data.df.PCoA<-cmdscale(Data.df, k = Dim, eig = FALSE)
 Data.df.PCoA.eig<-cmdscale(Data.df, k = Dim, eig = TRUE)
 eig.Data.df.PCoA<-Data.df.PCoA.eig$eig
 eig.Data.df.PCoA.sum<-sum(eig.Data.df.PCoA)
 a<-(eig.Data.df.PCoA/eig.Data.df.PCoA.sum)*100
 xlab<-paste("PC1","(",round(a[1],1),"%",")",sep="")
 ylab<-paste("PC2","(",round(a[2],1),"%",")",sep="")
 if(binary==TRUE){
  main<-"Sorensen PCoA"
 }else(main<-"Bray-Curtis PCoA")
 plot(Data.df.PCoA, col=Color,
    main=Title,xlab=xlab,ylab=ylab,pch=c(pch))
 return(Data.df.PCoA)
}
#sor
b<-as.factor(indices$GBS)
df.spe.bray.Sor<-Sor.bray.pcoa(abun, Dim = 2, Color = b, binary = TRUE)
ordiellipse(df.spe.bray.Sor,groups=b,col=c(1,2),lwd=1)
legend(-0.2,0.4,c("No","Yes"), pch=21,col=1,pt.bg=c(1,2))
#text(df.spe.bray.Sor,labels=b,col=as.numeric(b))


#permanova
```

```
PERMANOVA<-function(abun,Group,binary,iters=9999){
  Data.Dist<-vegdist(abun,method="bray", binary=binary)
  adonis2(Data.Dist~Group,permutations=iters)
}
PERMDISP<-function(abun,Group,binary,iters=9999){
  Data.Dist<-vegdist(abun,method="bray", binary=binary)
  Data.betadisper<-betadisper(Data.Dist, group=Group)
  permutest(Data.betadisper, group=Group, permutations=iters)
}

PERMANOVA(presence2[,c(28:143)],b,TRUE,9999)
#0.7032
PERMDISP(presence2[,c(28:143)],b,TRUE,9999)
#0.6496

####has baby has ABX since birth
indices$ABX<-abundance$`Has.baby.had.antibiotics.since.birth?`
table(abundance$`Has.baby.had.antibiotics.since.birth?`)
table(indices$ABX)



#stacked box plot
boxplot<-c(indices$RichMGE,indices$Richamino,indices$RichMDR, indices$Richbeta, indices$Richtetra,  indices$RichMLSB, indices$Richsulfo, indices$Richvanco, indices$Richfluoro)
ABX<-rep(indices$ABX,9)
```

```
type<-
rep(c("MGE","aminoglycoside","MDR","betalactamase","tetracycline","MLSB","sulfonamide","
vancomycin","fluoroquinolone"),each=192)

length(boxplot)


#192


boxplot2<-data.frame(boxplot,type,ABX)


ab<-ggplot(boxplot2, aes(x = type, y = boxplot, fill=ABX)) +ylim(0,30)+
geom_boxplot(width=0.4,position=position_dodge(0.4)) + theme_minimal() +
theme(axis.text.x = element_text(angle = 90)) + labs(title = "", y = "Richness", x = "") +
stat_compare_means(aes(group = ABX),label="p.signif") +
scale_fill_manual(values=c("#E69F00", "#56B4E9", "#FC4E07"))+
theme(legend.position="top",)

ab

#ggsave("RichnessClassAll.tiff", units="in", width=7.5, height=5, dpi=300)

#2023APR28_RichnessClass_MatchedPairs_3mos_abxclass_MR


wilcox.test(RichARG~ABX, data=indices)

#p-value =0.6277

wilcox.test(RichMGE~ABX, data=indices)

# p-value = 0.4912

wilcox.test(Richamino~ABX, data=indices)

# p-value = 0.1964


#Richness
```

```
b<-ggboxplot(data=indices, x="ABX", y="RichARG", color="black", fill="ABX",palette="d3",
ylab="Richness", xlab="", outlier.colour = "black", outlier.shape = 1, main="Rich ARG")+
theme_classic() + guides(fill=FALSE) + coord_cartesian(ylim = c(0, 100))+
stat_compare_means(label.x=1.3, label.y=95)

b


a<-ggboxplot(data=indices, x="ABX", y="RichMGE", color="black", fill="ABX",palette="d3",
ylab="Richness", xlab="", outlier.colour = "black", outlier.shape = 1, main="Rich MGE")+
theme_classic() + guides(fill=FALSE) + coord_cartesian(ylim = c(0, 40))+
stat_compare_means(label.x=1.7, label.y=35)

a


#combine these

figure <- ggarrange(a, b,labels = c("A", "B", ncol = 2, nrow = 1))

figure

#ggexport(figure, filename = "RichMGE_ARG_matchedpairs.tiff")


#Shannon

wilcox.test(ShannonMGERA~ABX, data=indices)

#p-value = 0.6109

wilcox.test(ShannonARGRA~ABX, data=indices)

#p-value = 0.763

wilcox.test(ShannonfluoroRA~ABX, data=indices)

#0.0134


#shannon

d<-ggboxplot(data=indices, x="ABX", y="ShannonARGRA", color="black",
fill="ABX",palette="d3", ylab="Shannon Diversity Index", xlab="", outlier.colour = "black",
```

```
outlier.shape = 1, main="Shannon ARG")+ theme_classic() + guides(fill=FALSE) +
coord_cartesian(ylim = c(0, 4.0))+ stat_compare_means(label.x=1.3, label.y=3.8)

d

c<-ggboxplot(data=indices, x="ABX", y="ShannonMGERA", color="black",
fill="ABX",palette="d3", ylab="Shannon Diversity Index", xlab="", outlier.colour = "black",
outlier.shape = 1, main="Shannon MGE")+ theme_classic() + guides(fill=FALSE) +
coord_cartesian(ylim = c(0, 4.0))+ stat_compare_means(label.x=1.3, label.y=3.8)

c


#combine all 4

figure <- ggarrange(a, b, c, d, labels = c("A", "B", "C","D", ncol = 2, nrow = 2))

figure

#ggexport(figure, filename = "ShannonMGE_ARG_matchedpairs.pdf")

##2023APR28_Rich_shan_Class_MatchedPairs_3mos_MR



#stacked box plot

boxplot<-c(indices$ShannonMGERA,indices$ShannonaminoRA,indices$ShannonMDRRA,
indices$ShannonbetaRA, indices$ShannontetraRA,  indices$ShannonMLSBRA,
indices$ShannonsulfoRA, indices$ShannonvancoRA, indices$ShannonfluoroRA)

ABX<-rep(indices$ABX,9)

type<-
rep(c("MGE","aminoglycoside","MDR","betalactamase","tetracycline","MLSB","sulfonamide","
vancomycin","fluoroquinolone"),each=191)


length(boxplot)


boxplot2<-data.frame(boxplot,type,ABX)
```

```
ab<-ggplot(boxplot2, aes(x = type, y = boxplot, fill=ABX)) +ylim(0,4)+
geom_boxplot(width=0.4,position=position_dodge(0.4)) + theme_minimal() +
theme(axis.text.x = element_text(angle = 90)) + labs(title = "", y = "Shannon Diversity Index ",
x = "") + stat_compare_means(aes(group = ABX),label="p.signif") +
scale_fill_manual(values=c("#E69F00", "#56B4E9", "#E69F00"))+
theme(legend.position="top",)

ab

#ggsave("SumShanAbudancesClass_matchedpairs.tiff", units="in", width=7.5, height=5,
dpi=300)

#2023APR28__shan_Class_MatchedPairs_ABXclass_3mos_MR


#stacked bar plot

sumabundance<-aggregate(boxplot~type+ABX, boxplot2,median)

stackplot<-ggplot(data = sumabundance, aes(x = ABX, y=boxplot)) +
geom_bar(stat="identity", aes(fill=type))+theme_classic() +
scale_fill_brewer(palette="Paired")+ labs(title = "", y = "Shannon Index", x = "")+theme
(axis.text = element_text(size=14), axis.title=element_text(size=14))

stackplot

#2023APR28__Stackplot_shan_Class_MatchedPairs_ABXclass_3mos_MR

#ggexport(stackplot, filename = "/Users/madeleinerussell/Desktop/Comstock
Lab/AbxResistance/ABXR_Ranalysis/ShannonDiv_ALL.pdf")


wilcox.test(SimpsonMGERA~ABX, data=indices)

#p-value =0.447

wilcox.test(SimpsonARGRA~ABX, data=indices)

#p-value = 0.969

wilcox.test(SimpsonfluoroRA~ABX, data=indices)

#0.0111
```

```
boxplot<-c(indices$SimpsonMGERA,indices$SimpsonaminoRA,indices$SimpsonMDRRA, indices$SimpsonbetaRA, indices$SimpsontetraRA,  indices$SimpsonMLSBRA, indices$SimpsonsulfoRA, indices$SimpsonvancoRA, indices$SimpsonfluoroRA)

ABX<-rep(indices$ABX,9)

type<-
rep(c("MGE","aminoglycoside","MDR","betalactamase","tetracycline","MLSB","sulfonamide","vancomycin","fluoroquinolone"),each=191)

length(boxplot)

boxplot2<-data.frame(boxplot,type,ABX)

ab<-ggplot(boxplot2, aes(x = type, y = boxplot, fill=ABX)) +ylim(0,12)+ geom_boxplot(width=0.4,position=position_dodge(0.4)) + theme_minimal() + theme(axis.text.x = element_text(size=10, angle = 45)) + labs(title = "", y = "Inverse Simpson Diversity Index", x = "") + stat_compare_means(aes(group = ABX),label="p.signif") + scale_fill_manual(values=c("#9F79EE", "#FF8247"))+ theme(legend.position="top",)

ab

ggsave("2023DEC05_simp_inf_abx_SF10.tiff", units="in", width=7.5, height=5, dpi=300)

#beta diversity

abundance2$ABX<-indices$ABX

abundance2_omit<-abundance2 %>% drop_na(ABX)
```

```
indices_omit<-indices %>% drop_na(ABX)
presence2$ABX<-indices$ABX
presence2_omit<-presence2 %>% drop_na(ABX)

indices<-indices_omit
presence2<-presence2_omit
abundance2<-abundance2_omit

abun<-abundance2[1:27]
head(abun)




#bray curtis
Sor.bray.pcoa<-function(abun,Dim=2,Color=1,binary,pch=16,Title="Bray-Curtis MGE"){
 Data.df<-vegdist(abun,method="bray", binary)
 Data.df.PCoA<-cmdscale(Data.df, k = Dim, eig = FALSE)
 Data.df.PCoA.eig<-cmdscale(Data.df, k = Dim, eig = TRUE)
 eig.Data.df.PCoA<-Data.df.PCoA.eig$eig
 eig.Data.df.PCoA.sum<-sum(eig.Data.df.PCoA)
 a<-(eig.Data.df.PCoA/eig.Data.df.PCoA.sum)*100
 xlab<-paste("PC1","(",round(a[1],1),"%",")",sep="")
 ylab<-paste("PC2","(",round(a[2],1),"%",")",sep="")
 if(binary==TRUE){
  main<-"Sorensen PCoA"
 }else(main<-"Bray-Curtis PCoA")
```

```
  plot(Data.df.PCoA, col=Color,
       main=Title,xlab=xlab,ylab=ylab,pch=c(pch))
  return(Data.df.PCoA)
}

#abun<-abundance2[28:143]
b<-as.factor(indices$ABX)
df.spe.bray.Sor<-Sor.bray.pcoa(abun, Dim = 2, Color = b, binary = FALSE)
ordiellipse(df.spe.bray.Sor,groups=b,col=c(1,2),lwd=1)
legend(-0.4,0.3,c("No","Yes"), pch=21,col=1,pt.bg=c(1,2))
#text(df.spe.bray.Sor,labels=b,col=as.numeric(b))

#permanova
PERMANOVA<-function(abun,Group,binary,iters=9999){
  Data.Dist<-vegdist(abun,method="bray", binary=binary)
  adonis2(Data.Dist~Group,permutations=iters)
}
PERMDISP<-function(abun,Group,binary,iters=9999){
  Data.Dist<-vegdist(abun,method="bray", binary=binary)
  Data.betadisper<-betadisper(Data.Dist, group=Group)
  permutest(Data.betadisper, group=Group, permutations=iters)
}


PERMANOVA(abundance2[,c(1:27)],b,FALSE,9999)
#p-value= 0.0588
```

```
PERMDISP(abundance2[,c(1:27)],b,FALSE,9999)

#0.2198


#Sorensen


abun<-presence2[1:27]

Sor.bray.pcoa<-function(abun,Dim=2,Color=1,binary,pch=16,Title="Sorensen MGE"){
 Data.df<-vegdist(abun,method="bray", binary)
 Data.df.PCoA<-cmdscale(Data.df, k = Dim, eig = FALSE)
 Data.df.PCoA.eig<-cmdscale(Data.df, k = Dim, eig = TRUE)
 eig.Data.df.PCoA<-Data.df.PCoA.eig$eig
 eig.Data.df.PCoA.sum<-sum(eig.Data.df.PCoA)
 a<-(eig.Data.df.PCoA/eig.Data.df.PCoA.sum)*100
 xlab<-paste("PC1","(",round(a[1],1),"%",")",sep="")
 ylab<-paste("PC2","(",round(a[2],1),"%",")",sep="")
 if(binary==TRUE){
  main<-"Sorensen PCoA"
 }else(main<-"Bray-Curtis PCoA")
 plot(Data.df.PCoA, col=Color,
     main=Title,xlab=xlab,ylab=ylab,pch=c(pch))
 return(Data.df.PCoA)
}
#sor
b<-as.factor(indices$ABX)
df.spe.bray.Sor<-Sor.bray.pcoa(abun, Dim = 2, Color = b, binary = TRUE)
ordiellipse(df.spe.bray.Sor,groups=b,col=c(1,2),lwd=1)
```

```
legend(-0.2,-0.2,c("No","Yes"), pch=21,col=1,pt.bg=c(1,2))
#text(df.spe.bray.Sor,labels=b,col=as.numeric(b))


#permanova
PERMANOVA<-function(abun,Group,binary,iters=9999){
  Data.Dist<-vegdist(abun,method="bray", binary=binary)
  adonis2(Data.Dist~Group,permutations=iters)
}
PERMDISP<-function(abun,Group,binary,iters=9999){
  Data.Dist<-vegdist(abun,method="bray", binary=binary)
  Data.betadisper<-betadisper(Data.Dist, group=Group)
  permutest(Data.betadisper, group=Group, permutations=iters)
}

indices$ABX

PERMANOVA(presence2[,c(1:27)],b,TRUE,9999)
#0.0267
PERMDISP(presence2[,c(1:27)],b,TRUE,9999)
#0.9551
summary(bcdata$MOM_AGE)

table21<-(table(bcdata$ID, bcdata$BMI))
table21

table(bcdata$BMI)
```

```
summary(bcdata$GRAMS)

#doing this for matched pair babies

#2023APR18_abundance3mos_plusmoms_MR.xlsx

#need to do it with only matched data

abundance3mos.matched<-
read.xlsx("2023APR18_AMR_3mos_matchedmoms_bbys.xlsx")
presence3mos.matched<-read.xlsx("2023APR18_pres_3mos_matchedmoms_bbys.xlsx")
abundance3mos<-abundance3mos.matched
presence3mos<-presence3mos.matched

bcdata<-read.xlsx("2023APR21_AMR_3mosBCData_plusabun.xlsx")
meta<-read.xlsx("2023APR17_AMR_3mosMetaData.xlsx")
table(meta$total_inf_treated)

#make matching presence
df6<-bcdata[,c(1,150:172)]
pres.bcdata<-inner_join(presence3mos, df6, by='ID')
abun.bcdata<-inner_join(abundance3mos, df6, by='ID')
```

```
abundance<-abun.bcdata

presence<-pres.bcdata


presence2<- presence[,4:146]

presence2


abundance2<- abundance[,4:146]





#did this in case you skip the medium stuff above and need to make variable names

abundance$Race<-as.factor(ifelse(abundance$BRIDGEMOMRACE== '1', 'white',

                        ifelse(abundance$BRIDGEMOMRACE== '2', 'black',

                            ifelse(abundance$BRIDGEMOMRACE== '24', 'asian',

                                ifelse(abundance$BRIDGEMOMRACE== '10', 'asian',

                                    ifelse(abundance$BRIDGEMOMRACE== '22', 'black',

                                        ifelse(abundance$BRIDGEMOMRACE== '8', 'asian',
'other')))))))




abundance$Race2<-as.factor(ifelse(abundance$Race== 'white', 'white', 'non-white'))


abundance$MOD<-as.factor(ifelse(abundance$MD_FINAL_ROUTE== '1','vaginal',

                        ifelse(abundance$MD_FINAL_ROUTE== '2', 'vaginal',
```

```
                    ifelse(abundance$MD_FINAL_ROUTE== '3', 'vaginal',
                        ifelse(abundance$MD_FINAL_ROUTE== '4', 'cesarean', 'other')))))
abundance$MOD

table(abundance$pair, abundance$MOD)



names(sharedpie)[names(sharedpie) == 'pair.y'] <- 'pair'
dataframe<-sharedpie
dataframe$pair <- lapply(dataframe$pair, as.character)
dataframe$pair<-as.character(dataframe$pair)
is.character(dataframe$pair)
sharedgenes<-inner_join(abundance, dataframe, by='pair')



sharedgenes$percentage
table(sharedgenes$percentage, sharedgenes$MOD)

sharedgenes$cate_percent_shared<-as.factor(ifelse(sharedgenes$percentage >=
0.5,'>50%',
                        ifelse(sharedgenes$percentage <= 0.5, '<50%', 'other')))
sharedgenes$cate_percent_shared

#prop tests
table(sharedgenes$cate_percent_shared, sharedgenes$MOD)
```

```
table1<-as.table(table(sharedgenes$cate_percent_shared, sharedgenes$MOD))

table1

prop.table(table1,1)


#kind of a pretty plot

dt <- as.table(as.matrix(table1))


#balloonplot(t(dt), main ="Infections", xlab ="", ylab="",

#         label = FALSE, show.margins = FALSE)



chisq.test(table1)

#p-value = 5.584e-16


FUN = function(i,j){

  chisq.test(matrix(c(table1[i,1], table1[i,2],

                      table1[j,1], table1[j,2]),

                    nrow=2,

                    byrow=TRUE))$ p.value

}


pairwise.table(FUN,

               rownames(table1),

               p.adjust.method="none")


sharedgenes[c(1:33), c(1,3,179,175)]
```

```
### food status any vs. some

SIF<-read.xlsx("MARCH3mos_SIF_MR.xlsx")

names(SIF)[names(SIF) == 'Study.ID'] <- 'match'

head(SIF)

SIF<-SIF[,-c(11:12)]


SIFabun2<-inner_join(sharedgenes, SIF, by="match")

df1 <- SIFabun2 %>% distinct(match, .keep_all = TRUE)

df1


df1$foodstatus2<-gsub(".*100% breast.*", "breastfed",

             gsub(".*100% formula.*", "formula",

               gsub(".*20-50%.*", "breastfed",

                 gsub(".*50%.*", "breastfed",

                   gsub(".*20% breast milk.*", "breastfed",

                     gsub(".*80% breast milk.*", "breastfed",
df1$` During.the.past.week,.my.baby.ate:` ))))))

df1$foodstatus2

table(df1$foodstatus2)


#prop tests

table(df1$cate_percent_shared, df1$MOD)


table1<-as.table(table(df1$foodstatus2, df1$MOD))

table1
```

```
prop.table(table1,1)

#kind of a pretty plot
dt <- as.table(as.matrix(table1))

#balloonplot(t(dt), main ="Infections", xlab ="", ylab="",
#           label = FALSE, show.margins = FALSE)



chisq.test(table1)
#p-value = 5.584e-16

FUN = function(i,j){
  chisq.test(matrix(c(table1[i,1], table1[i,2],
                      table1[j,1], table1[j,2]),
                    nrow=2,
                    byrow=TRUE))$ p.value
}

pairwise.table(FUN,
               rownames(table1),
               p.adjust.method="none")

df1[c(1:33), c(1,3,180,175,216)]

df1[c(1:33), c(1,3,216)]
```

```
#creating complete dataset

smartchip.1<-read.xlsx('Smartchip data_Avg CT_part1.xlsx')
smartchip.2<-read.xlsx('Smartchip data_Avg CT_part2.xlsx')

smartchip.combined<-rbind(smartchip.1, smartchip.2) #combine smartchip files first

#abundance and pres data
abundance3mos<-read.xlsx("2023APR18_abundance3mos_plusmoms_MR.xlsx")
presence3mos<-read.xlsx("2023APR18_presence3mos_plusmoms_MR.xlsx")

abundance3mos$ID

smartchip.combined$Assay_IDnum
names(smartchip.combined)[names(smartchip.combined) == 'Assay_IDnum'] <- 'ID'
abun3mos<-left_join(abundance3mos, presence3mos, by = 'ID')
abun3mos2<-right_join(smartchip.combined, abun3mos, by = 'ID')



abun3mos2 <- abun3mos2 %>% distinct(match.y, .keep_all = TRUE)
abun3mos2

SIF<-read.xlsx("MARCH3mos_SIF_MR.xlsx")
names(SIF)[names(SIF) == 'Study.ID'] <- 'match.y'
SIF2<-SIF[,-c(2:6,8:9,31:38)] # only included relevant columns
```

```
names(SIF2)[names(SIF2) == 'match'] <- 'match.y'

SIFabun2<-left_join(abun3mos2, SIF2, by="match.y")
df1 <- SIFabun2 %>% distinct(match.y, .keep_all = TRUE)
df1

crossmeta<-read.xlsx("2023_3mos_Crosswalk_4_MAdi.xlsx")
crossmeta$Specimen.ID
names(crossmeta)[names(crossmeta) == 'Specimen.ID'] <- 'match.y'

df1.cross<-left_join(df1, crossmeta, by="match.y")
df2 <- df1.cross %>% distinct(match.y, .keep_all = TRUE)

bcdata<-read.xlsx("2023APR21_AMR_3mosBCData_plusabun.xlsx")
bcdata2<-bcdata[,c(148:172)] # only included relevant columns
names(bcdata2)[names(bcdata2) == 'match'] <- 'match.y'

df2.bc<-left_join(df2, bcdata2, by="match.y") # add in BC data
df2.bc$MARCHID.x
names(df2.bc)[names(df2.bc) == 'MARCHID.x'] <- 'MARCH.ID'

metadata<-read.xlsx("20221031_AMR_MetaData.xlsx")
metadata$ID
names(metadata)[names(metadata) == 'ID'] <- 'MARCH.ID'
df3<-left_join(df2.bc, metadata, by="MARCH.ID") # add in BC data
```

```
df4<-df3[,-c(473:475, 480:481)]

write.csv(df4, "/Users/madeleinerussell/Library/Mobile
Documents/com~apple~CloudDocs/Desktop/Desktop - Madeleine's MacBook -
1/Comstock
Lab/AbxResistance/ABXR_Ranalysis/AMR_Data/2024MAR01_AMR_complete_data.csv",
row.names = FALSE)

getwd()

complete_data<-read.csv("2024MAR01_AMR_complete_data.csv")

mom_data<-read.xlsx("2024MAY29_Mom_complete_data.xlsx")

print("Original DataFrame : ")
print(mom_data)

print("Original col names")
print(colnames(mom_data))

# adding suffix to column names
colnames(mom_data) <- paste(colnames(mom_data),"new",sep="_")

print("New DataFrame : ")
print(mom_data)

print("New col names")
```

```
print(colnames(mom_data)) #added this code so can left_join with complete_data but have the columns have different names so when we submit for review, no one yells at me

mom_data$ID_new

names(mom_data)[names(mom_data) == 'ID_new'] <- 'ID'

complete_data2<-left_join(complete_data, mom_data, by = 'ID')

write.csv(complete_data2, "/Users/madeleinerussell/Library/Mobile Documents/com~apple~CloudDocs/Desktop/Desktop - Madeleine’s MacBook - 1/Comstock Lab/AbxResistance/ABXR_Ranalysis/AMR_Data/2024MAY29_AMR_complete_data.csv", row.names = FALSE)

df5 <- tibble::rownames_to_column(a, "Genename")

df5$Genename<-gsub('X',"",df5$Genename)

names(df5)[names(df5) == 'Genename'] <- 'Assay'

df5$Included_In_Analysis<-"Yes"

genenames<-read.xlsx("2024MAY15_gene_names_3mos_AMR_MR.xlsx")

genenames$Assay<-as.factor(as.character(genenames$Assay))

gene.list.full<-left_join(genenames, df5, by='Assay')

gene.list.full<-gene.list.full[,-c(5)]
```

```
gene.list.full2<-inner_join(genenames, df5, by='Assay')

gene.list.full2<-gene.list.full2[,-c(5)]



write.xlsx(gene.list.full2, "2024MAY24_genelist_supplementary_table1.xlsx", quote=FALSE, rowNames=FALSE)

write.xlsx(gene.list.full, "2024MAY24_genelist_full.xlsx", quote=FALSE, rowNames=FALSE)



###Testing HC clustering

###July 7th, 2023


library("gplots")

library("ggpubr")

library(dplyr)

library(RColorBrewer)

library(ggplot2)

library(viridis)

library(dplyr)

library(forcats)

require(openxlsx)

require(tidyr)

library(gridExtra)

library(vegan)

library(Hmisc)

library(plyr)
```

```
library("plotrix")

library(tidyverse)

library(ggpubr)

library(fossil)

#install.packages("fossil")


#set your working directory

setwd("/Users/madeleinerussell/Library/Mobile Documents/com~apple~CloudDocs/Desktop/Desktop - Madeleine’s MacBook - 1/Comstock Lab/AbxResistance/ABXR_Ranalysis/AMR_Data")


abundance3mos<-read.xlsx("2023APR18_abundance3mos_plusmoms_MR.xlsx")

presence3mos<-read.xlsx("2023APR18_presence3mos_plusmoms_MR.xlsx")



head(presence3mos)


#look at data first

library(ggplot2)



is.character(new_df2$cluster)

new_df2$cluster <- as.character(new_df2$cluster)

#ggplot(abundance3mos, aes(abundance3mos$`6`, abundance3mos$`201`, color = abundance3mos$class)) + geom_point()

ggplot(new_df2, aes(new_df2$`201`, new_df2$`412`, color = new_df2$cluster)) + geom_point()
```

```
#exploring clustering

# Since we know that there are 3 species involved, we ask the algorithm to group the data

# into 3 clusters, and since the starting assignments are random, we specify nstart = 20.

# This means that R will try 20 different random starting assignments and then select

# the one with the lowest within cluster variation.


set.seed(20)

hcCluster <- kmeans(new_df2[, 3:145], 2, nstart = 20)

hcCluster


# We can see the cluster centroids, the clusters that each data point was assigned to

# and the within cluster variation.

#Let us compare the clusters with the species.

table(hcCluster$cluster, new_df2$cluster)


hcCluster$cluster <- as.factor(hcCluster$cluster)

ggplot(new_df2, aes(new_df2$`201`, new_df2$`412`, color = hcCluster$cluster)) +
geom_point()


## part II

# Finding an interesting number of clusters in a dendrogram is the same as

# finding the largest horizontal space that doesn't have any vertical lines

# (the space with the longest vertical lines). This means that there's more

# separation between the clusters.
```

```
clusters <- hclust(dist(new_df2[, 4:146]))

plot(clusters)


clusterCut <- cutree(clusters, 2)


table(clusterCut, abundance3mos$class)


#mean linkage method

clusters <- hclust(dist(abundance3mos[, 4:5]), method = 'average')

plot(clusters)


#more accurate

clusterCut <- cutree(clusters, 3)

table(clusterCut, abundance3mos$class)


ggplot(abundance3mos, aes(abundance3mos$`26`, abundance3mos$`201`, color =
abundance3mos$class)) +

  geom_point(alpha = 0.4, size = 3.5) + geom_point(col = clusterCut) +

  scale_color_manual(values = c('black', 'red', 'green'))


## Part III: using different methods

disssim<-abundance3mos[, 4:148]

spellman.cor <- dplyr::select(disssim, -class, -match) %>%

  cor(use='pairwise.complete.obs')


spellman.dist <- as.dist(1 - spellman.cor)
```

```
spellman.tree <- hclust(spellman.dist, method="complete")

plot(spellman.tree)
plot(spellman.tree, cex=0.3)

#Part III b: making it prettier
#install.packages('dendextend')
library(dendextend)

spellman.dend <- as.dendrogram(spellman.tree) # create dendrogram object
nleaves(spellman.dend)  # number of leaves in tree
## [1] 143
nnodes(spellman.dend)  # number of nodes (=leaves + joins) in tree
## [1] 285

plot(spellman.dend, leaflab = "none")
clusters <- cutree(spellman.dend, k=4)
table(clusters)
## clusters
##  1   2   3   4
# 18  17   4 104

clusters[1:6]
plot(color_branches(spellman.dend, k=4),leaflab="none")
```

```
plot(color_branches(spellman.dend, k=8),leaflab="none")


clusters <- cutree(spellman.dend, k=8, order_clusters_as_data = FALSE)

table(clusters)

## clusters

# 1  2  3  4  5  6  7  8

# 5  2 11 17  4 15  4 85


clusters.df <- data.frame(gene = names(clusters), cluster = clusters)


# Having created this data frame, it's straightforward to lookup the

# cluster to which a gene belongs:

clusters.df["26",]


# or to get all the names of genes in a given cluster:

cluster3.genes <- filter(clusters.df, cluster == 3)$gene


cat(as.character(cluster3.genes[1:10]), quote=FALSE,sep="\n");


spellman.long <- gather(disssim, gene, expression, -class, -match)

head(spellman.long)

## # A tibble: 6 × 4

# class    match gene   expression

# 1 Infant 25FE8862   26 3.289741e-01

# 2 Infant 25FE8863   26 1.610433e-04

# 3 Infant 25FE8481   26 5.047662e-06
```

```
# 4 Infant 25FE8598   26 6.380541e-01

# 5 Infant 25FE8948   26 4.332441e-01

# 6 Infant 25FE8712   26 1.795996e-04


color.scheme <- rev(brewer.pal(8,"RdBu")) # generate the color scheme to use


disssim$class

spellman.long %>%

  filter(gene %in% cluster3.genes & class == "Infant") %>%

  ggplot(aes(x = match, y = gene)) +

  geom_tile(aes(fill = expression)) +

  scale_fill_gradientn(colors=color.scheme, limits = c(0,0.0001)) +

  theme(axis.text.y = element_text(size = 5))  # set size of y axis labels



# note that I determined the height to cut at by looking at the colored dendrogram

# plot above for 8 clusters

sub.trees <- cut(spellman.dend, h = 1.0)

sub.trees$lower

cluster3.tree <- sub.trees$lower[[3]]

cluster3.tree

nleaves(cluster3.tree)


cluster3.tree  %>%

  set("labels_cex", 0.45) %>%

  set("labels_col", "red") %>%
```

```
  plot(horiz = TRUE)  # plot horizontally

# subset out the alpha factor data
alpha.factor <- filter(disssim, class == "Infant")

# create matrix after dropping time and expt columns
alpha.mtx <- as.matrix(dplyr::select(alpha.factor, -match, -class)) # drop time, expt
columns

# set row names to corresponding time points for nice plotting
row.names(alpha.mtx) <- alpha.factor$class

# transpose the matrix so genes are drawn in rows
transposed.alpha.mtx <- t(alpha.mtx)

# this is a large figure, so if working in RMarkdown document I suggest specifying
# the code block header as so to make the figure large
# {r, fig.width = 8, fig.height = 8}
heatmap.2(transposed.alpha.mtx,
      Rowv = cluster3.tree,  # use the dendrogram previously calculated
      Colv = NULL, # don't mess with my columns! (i.e. keep current ordering )
      dendrogram = "row",   # only draw row dendrograms
      breaks = seq(-2, 2, length.out = 9),  # OPTIONAL: set break points for colors
      col = color.scheme,  # use previously defined colors
      trace = "none", density.info = "none",  # remove distracting elements of plot
      xlab = "Class")
```

```
#another test

### another test thing

#Create random data

###https://www.biostars.org/p/287512/#287518

library(pheatmap)

# data <- replicate(20, rnorm(50))

# rownames(data) <- paste("Gene", c(1:nrow(data)))

# colnames(data) <- paste("Sample", c(1:ncol(data)))


infants1<-abundance3mos[grep("Infant",abundance3mos$class),] #select for infants

infants<-infants1[-grep("25FE8261",infants1$match),] #remove outlier infant

infants

df2 <-infants[,c(4:146)] #select for genes only

# rownames(df2) <- paste("Sample", c(1:nrow(df2)))

rownames(df2) <- infants$match

head(df2)


#put data into log scale

data_log <- log(df2)    # Log transformation of data

data_log


data2<-t(data_log) #switch columns and genes
```

```
dev.off()

out <- pheatmap(data2,
            show_rownames=F, show_colnames=F, main="Infants Heatmap", cluster_cols=T,
cluster_rows=T, scale="row",
            cex=1, clustering_distance_rows="euclidean", cex=1,
            clustering_distance_cols="euclidean", clustering_method="complete",
border_color=FALSE)

out
#grid.text("genes", x=0.3, y=0.1, rot = 270)

#Re-order original data (genes) to match ordering in heatmap (top-to-bottom)
test_col<-rownames(data2[out$tree_row[["order"]],])
test_col
#Re-order original data (samples) to match ordering in heatmap (left-to-right)
test_row<-colnames(data2[,out$tree_col[["order"]]])
test_row

vec<-colnames(data2[,out$tree_col[["order"]]])
vec

#this is done to link the clusters to correct order of IDs
clusters<-sort(cutree(out$tree_col, k=3)) #sorting by the same IDs that are in the
dendogram
df.test<-as.data.frame(clusters)                              # Duplicate example data
df.test2 <- tibble::rownames_to_column(df.test, "ID") # Apply rownames_to_column
df.test2
```

```
df.test3 <- df.test2[match(vec, df.test2$ID), ]  #create matching list

df.test3

sort(cutree(out$tree_row, k=3))


#madi doing things

datatest <- df2                                   # Duplicate example data

datatest <- tibble::rownames_to_column(datatest, "ID") # Apply rownames_to_column

datatest                                          # Print updated data


data_new1 <- datatest[match(vec, datatest$ID), ]       # Reorder data frame

data_new1$ID


data_new3 <- infants[match(vec, infants$match), ]

data_new3$match


data_new2<-data_new1[test_col]

data_new2$ID<-data_new1$ID

new_df <- data_new2 %>% select(ID, everything())

new_df

#now go back and pull the correct cluster order

new_df$cluster<-df.test3$clusters

new_df2<-new_df %>% select(ID, cluster, everything())

new_df2


#2023SEP05_cluster_abund_genes_MR.xlsx
```

```
write.xlsx(new_df2, "/Users/madeleinerussell/Desktop/Comstock Lab/AbxResistance/ABXR_Ranalysis/AMR_Data/2023SEP05_cluster_abund_genes_MR.xlsx
", quote=FALSE, rowNames=FALSE)



# Print updated data frame


test_col

infantdendo<-out$tree_col %>%

  as.dendrogram() %>%

  plot(horiz = FALSE)


infantdendo<-out$tree_row %>%

  as.dendrogram() %>%

  plot(horiz = FALSE)


plot(out$tree_row)

abline(h=21.5, col="red", lty=2, lwd=2)


#getting clusters of genes

genes<-as.data.frame(sort(cutree(out$tree_row, k=5)))

genes


#get order of genes

gene_vec<-rownames(data2[out$tree_row[["order"]],])

gene_vec
```

```
df.genes.test <- tibble::rownames_to_column(genes, "genes") # Apply
rownames_to_column

df.genes.test


df.genes.test2<- df.genes.test[match(gene_vec, df.genes.test$genes), ]

table(df.genes.test2$`sort(cutree(out$tree_row, k = 5))`)


# 1   2   3   4   5

# 11  17 103   3   9


#201 vs. 6




dend1 <- color_branches(infantdendo, k = 3)



#columns

infant_dendo<-plot(out$tree_col, k = 4)


abline(h=2, col="red", lty=2, lwd=2)


#bootstrapping with AU/BP

#https://corpling.hypotheses.org/2675

mat<-data_log
```

```
mat<-na.omit(mat)

mat <- t(mat)


library(pvclust)

fit <- pvclust(mat, method.hclust="complete", method.dist="euclidean")

fit


plot(fit)


summary(fit)


#The plot should be read from bottom to top. There are three numbers around each node. The number below each node specifies the rank of the cluster

#The number on the left indicates an 'approximately unbiased' p-value (AU) and is computed by multiscale bootstrap resampling

#The number on the right indicates a 'bootstrap probability' p-value (BP) and is computed by normal bootstrap resampling.

#left is better


#In either case, the closer the number is to 100 (i.e the closer the p-value is to 1),

#the more valid the cluster. For example, an AU p-value of, say, 90 implies that

#the hypothesis that the cluster is invalid is rejected with a significance level of 0.1


pvrect(fit, alpha=.95, pv="au", type="geq")
```

```
#messing around
#library(dendextend)


dend <- as.dendrogram(hclust(dist(data_log[1:146,])))
dend
plot(dend, main = "Original dend")
labels_colors(dend)
#
dend1 <- color_branches(dend, k = 3)
plot(dend1, main = "Infant Dendogram", horiz= TRUE)
#
plot(dend1,main = "Infant Dendogram", leaflab = "none")
# abline(h=50, col="red", lty=2, lwd=2)
# clusters <- cutree(dend1, k=3)
# table(clusters)
# clusters
# # clusters
# #  1  2  3
# # 90 42 14
#
#
# clusters[1:3]
# plot(color_branches(dend1, k=4),leaflab="none")
#
# plot(color_branches(dend1, k=8),leaflab="none")
#
```

```
# clusters.df <- data.frame(gene = names(clusters), cluster = clusters)
#
# # Having created this data frame, it's straightforward to lookup the
# # cluster to which a gene belongs:
# clusters.df["25FE8948",]
#
# # or to get all the names of genes in a given cluster:
# cluster3.samples <- filter(clusters.df, cluster == 3)$gene
# cluster2.samples <- filter(clusters.df, cluster == 2)$gene
# cluster1.samples <- filter(clusters.df, cluster == 1)$gene
#
# #list of genes names
# #df3<-infants2 %>% select(one_of(cluster1.samples))
# resistome_mom<-df3
#
#
# #selecting all of the genes that are in this cluster
# cat(as.character(cluster3.samples[1:10]), quote=FALSE,sep="\n");
#
# spellman.long <- gather(disssim, gene, expression, -class, -match)
# head(spellman.long)
```

```
## # A tibble: 6 × 4
# class    match gene   expression
# 1 Infant 25FE8862   26 3.289741e-01
# 2 Infant 25FE8863   26 1.610433e-04
```

```
# 3 Infant 25FE8481   26 5.047662e-06

# 4 Infant 25FE8598   26 6.380541e-01

# 5 Infant 25FE8948   26 4.332441e-01

# 6 Infant 25FE8712   26 1.795996e-04



#kind of a messy way to get the IDs assigned to a specific cluster but

#I was too lazy to find a better way


#basically, here we're just filtering for specific samples, creating a few data

#frames and binding them back together to get the column

#cluster_dend which we can then map back to our initial data

# cluster1.samples <- filter(clusters.df, cluster == 1)$gene

# cluster1.samples

# length(cluster1.samples)

# is.vector(cluster1.samples) #check to see if vector

# ID<-cluster1.samples

# Cluster_dend<-rep("A", 90) #create vector to assign new variable

# df.test<- data.frame(ID,Cluster_dend) #create data frame

#

# #part 2

# cluster2.samples <- filter(clusters.df, cluster == 2)$gene

# length(cluster2.samples)

# ID<-cluster2.samples

# Cluster_dend<-rep("B", 42) #create vector to assign new variable

# df.test1<- data.frame(ID,Cluster_dend)
```

```
#
# #part 3
# cluster3.samples <- filter(clusters.df, cluster == 3)$gene
# length(cluster3.samples)
# ID<-cluster3.samples
# Cluster_dend<-rep("C", 14) #create vector to assign new variable
# df.test2<- data.frame(ID,Cluster_dend)
#
# # combine three data frames vertically
# updated <- rbind(df.test, df.test1, df.test2)


install.packages('future')
install.packages('shipunov')
library(future)
plan(multisession)
library(shipunov)
library(dplyr)


#best way to do this that i've seen
# https://www.datacamp.com/tutorial/hierarchical-clustering-R
# #take like 5
#method="complete"
dist_mat <- dist(data_log, method = 'euclidean')
hclust_comp <- hclust(dist_mat)


plot(hclust_comp)
```

```
cut_comp <- cutree(hclust_comp, k = 3)


plot(hclust_comp)

rect.hclust(hclust_comp , k = 3, border = 2:6)

abline(h = 72.5, col = 'red')


avg_dend_obj <- as.dendrogram(hclust_comp)

avg_col_dend <- color_branches(avg_dend_obj, k = 3)

plot(avg_col_dend, main = "Infant Dendogram")


data_log_cl <- mutate(data_log, cluster = cut_comp)

count(data_log_cl,cluster)


cluster<-data_log_cl$cluster

RW.cl<-rownames(data_log_cl)

df.test123<- data.frame(RW.cl,cluster)


table(new_df2$cluster) #checking that they're the same


#this is the same groupings as the one above, HOWEVER, this flipped groups 1 and 2.

#Should not be an issue for analysis, as the groups are otherwise the same.


new_df2<-read.xlsx("2023SEP05_cluster_abund_genes_MR.xlsx")



vec<-new_df2$ID
```

```
infants_cluster <- infants[match(vec, infants$match), ]   #here i'm making infants the same order to get cluster group

infants_cluster$match


infants_cluster$cluster<-new_df2$cluster


table(infants_cluster$cluster)


ids<-infants$match


infants_cluster2<-infants_cluster[match(ids, infants_cluster$match), ]

infants_cluster2$ID #make these match again bc itll make madi's life easier


# 1   2   3

# 148  51  12


#now doing some quick analysis


bcdata<-read.xlsx("2023APR21_AMR_3mosBCData_plusabun.xlsx")


#doing this because un even number ids

df3<-infants_cluster2 %>% #first minimize number of infants with cluster to match BCdata

  filter(ID %in% bcdata$ID)

df4<-bcdata %>% #then match bc data to infants

  filter(ID %in% df3$ID)

df4$cluster<-df3$cluster #now they can vibe
```

```
df4$cluster

bcdata<-df4


bcdata$cluster

table(bcdata$cluster)

# 1   2   3

# 146  48  10


#mode of delivery

table(bcdata$MD_FINAL_ROUTE)

bcdata$MD_FINAL_ROUTE


bcdata$mode<-as.factor(ifelse(bcdata$MD_FINAL_ROUTE== '1','vaginal',
                  ifelse(bcdata$MD_FINAL_ROUTE== '2', 'vaginal',
                      ifelse(bcdata$MD_FINAL_ROUTE== '3', 'vaginal',
                          ifelse(bcdata$MD_FINAL_ROUTE== '4', 'cesarean', 'other')))))



#looking at feeding and abx data

SIF<-read.xlsx("MARCH3mos_SIF_MR.xlsx")

names(SIF)[names(SIF) == 'Study.ID'] <- 'match'

head(SIF)

SIF<-SIF[,-c(11:12)]


SIFabun2<-inner_join(bcdata, SIF, by="match")
```

```
df1 <- SIFabun2 %>% distinct(match, .keep_all = TRUE)

df1


table(df1$cluster)

# 1   2   3

# 140  47  10

#mode of delivery

table7 = as.table(table(df1$cluster,df1$mode))

table7

# cesarean vaginal

# 1      46     86

# 2      20     43

# 3       5      4


prop.table(table7,1)

# cesarean   vaginal

# 1 0.3000000 0.7000000

# 2 0.4042553 0.5957447

# 3 0.5000000 0.5000000


dt <- as.table(as.matrix(table7))

balloonplot(t(dt), main ="MOD", xlab ="", ylab="",

          label = FALSE, show.margins = FALSE)

chisq.test(table7)


FUN = function(i,j){
```

```
  chisq.test(matrix(c(table7[i,1], table7[i,2],
                    table7[j,1], table7[j,2]),
                  nrow=3,
                  byrow=TRUE))$ p.value
}

pairwise.table(FUN,
             rownames(table7),
             p.adjust.method="BH")



#checking feeding
table1<-as.data.frame(table(df1$`During.the.past.week,.my.baby.ate:`))

#checking abx
table2<-as.data.frame(table(df1$`Has.baby.had.antibiotics.since.birth?`))

#abx exposure x clustering
table7 = as.table(table(df1$cluster,df1$`Has.baby.had.antibiotics.since.birth?`))
table7
#NO YES
# 1 118  19
# 2  40   7
# 3   8   2
prop.table(table7,1)
# NO      YES
```

```
# 1 0.8480000 0.1520000
# 2 0.8666667 0.1333333
# 3 0.8888889 0.1111111

dt <- as.table(as.matrix(table7))
balloonplot(t(dt), main ="MOD", xlab ="", ylab="",
        label = FALSE, show.margins = FALSE)
chisq.test(table7)

# Pearson's Chi-squared test
#
# data:  table7
# X-squared = 0.19871, df = 2, p-value = 0.9054

FUN = function(i,j){
  chisq.test(matrix(c(table7[i,1], table7[i,2],
                      table7[j,1], table7[j,2]),
                    nrow=3,
                    byrow=TRUE))$ p.value
}

pairwise.table(FUN,
          rownames(table7),
          p.adjust.method="BH")
# 1       2
# 2 0.9698749       NA
```

```
# 3 0.9698749 0.9698749

df1$BRIDGEMOMRACE

df1$Race<-as.factor(ifelse(df1$BRIDGEMOMRACE== '1', 'white',
                    ifelse(df1$BRIDGEMOMRACE== '2', 'black',
                         ifelse(df1$BRIDGEMOMRACE== '24', 'asian',
                              ifelse(df1$BRIDGEMOMRACE== '10', 'asian',
                                   ifelse(df1$BRIDGEMOMRACE== '22', 'black',
                                        ifelse(df1$BRIDGEMOMRACE== '8', 'asian', 'other')))))))

df1$Race2<-as.factor(ifelse(df1$Race== 'white', 'white', 'non-white'))

table(df1$Race2)

# non-white    white

# 50      146

###all races all locations

table20 = as.table(table(df1$cluster,df1$Race2))

table20

prop.table(table20,1)

#(using a 2 would have given you proportion by column)

dt <- as.table(as.matrix(table20))

balloonplot(t(dt), main ="race", xlab ="", ylab="",
```

```
        label = FALSE, show.margins = FALSE)
chisq.test(table20)
FUN = function(i,j){
  chisq.test(matrix(c(table20[i,1], table20[i,2],
                      table20[j,1], table20[j,2]),
                    nrow=2,
                    byrow=TRUE))$ p.value
}

pairwise.table(FUN,
               rownames(table20),
               p.adjust.method="none")



## feeding


df1$foodstatus<-gsub(".*100% breast.*", "100%",
                     gsub(".*100% formula.*", "<20%",
                          gsub(".*20-50%.*", "50-80%",
                               gsub(".*50%.*", "50-80%",
                                    gsub(".*20% breast milk.*", "<20%",
                                         gsub(".*80% breast milk.*", "50-80%",
df1$`During.the.past.week,.my.baby.ate:`))))))


df1$foodstatus
table(df1$foodstatus)
```

```
# <20%   100% 50-80%
# 80    85    29


table20 = as.table(table(df1$cluster,df1$foodstatus))
table20
prop.table(table20,1)


dt <- as.table(as.matrix(table20))
balloonplot(t(dt), main ="race", xlab ="", ylab="",
        label = FALSE, show.margins = FALSE)
chisq.test(table20)
#p-value < 2.2e-16


FUN = function(i,j){
  chisq.test(matrix(c(table20[i,1], table20[i,2],
                  table20[j,1], table20[j,2]),
                 nrow=4,
                 byrow=TRUE))$ p.value
}


pairwise.table(FUN,
           rownames(table20),
           p.adjust.method="none")


### food status any vs. some
# df1$foodstatus2<-gsub(".*100% breast.*", "breastfed",
```

```
#                   gsub(".*100% formula.*", "formula",
#                       gsub(".*20-50%.*", "breastfed",
#                           gsub(".*50%.*", "breastfed",
#                               gsub(".*20% breast milk.*", "breastfed",
#                                   gsub(".*80% breast milk.*", "breastfed",
df1$`During.the.past.week,.my.baby.ate:` ))))))
# df1$foodstatus2
# table(df1$foodstatus2)
#breastfed (any breast) = 121
#formula fed (any formula) = 73


table20 = as.table(table(df1$cluster,df1$foodstatus2))
table20
prop.table(table20,1)


dt <- as.table(as.matrix(table20))
balloonplot(t(dt), main ="food status", xlab ="", ylab="",
        label = FALSE, show.margins = FALSE)
chisq.test(table20)
# Pearson's Chi-squared test
#
# data:  table20
# X-squared = 2.6636, df = 2, p-value = 0.264


FUN = function(i,j){
  chisq.test(matrix(c(table20[i,1], table20[i,2],
```

```
              table20[j,1], table20[j,2]),
             nrow=4,
             byrow=TRUE))$ p.value
}

pairwise.table(FUN,
         rownames(table20),
         p.adjust.method="none")

#grouping towards all FF
df1$foodstatus2<-gsub(".*100% breast.*", "breastfed",
              gsub(".*100% formula.*", "formula",
                gsub(".*20-50%.*", "formula",
                  gsub(".*50%.*", "formula",
                    gsub(".*20% breast milk.*", "formula",
                      gsub(".*80% breast milk.*", "formula",
df1$`During.the.past.week,.my.baby.ate:` ))))))
df1$foodstatus2
table(df1$foodstatus2)
#FF= 109
#BF= 85

table(df1$foodstatus2)
#breastfed (any breast) = 121
#formula fed (any formula) = 73
```

```
table20 = as.table(table(df1$cluster,df1$foodstatus2))

table20

prop.table(table20,1)


dt <- as.table(as.matrix(table20))

balloonplot(t(dt), main ="food status", xlab ="", ylab="",

        label = FALSE, show.margins = FALSE)

chisq.test(table20)

#p-value < 2.2e-16


FUN = function(i,j){

  chisq.test(matrix(c(table20[i,1], table20[i,2],

                    table20[j,1], table20[j,2]),

                  nrow=4,

                  byrow=TRUE))$ p.value

}


pairwise.table(FUN,

          rownames(table20),

          p.adjust.method="none")

### multi-variate analysis

#need to download zip file from online source

#install.packages('https://cran.r-project.org/src/contrib/Archive/dominanceanalysis/dominanceanalysis_2.0.0.tar.gz', repos=NULL, type='source')

#install.packages('lm.beta')

require(lm.beta)
```

```
library(devtools)

#install_github("clbustos/dominanceAnalysis")

library(dominanceanalysis)



#create variables


indices <- df1[,c("class","ID")]


df1$mode

indices$MOD<-df1$mode

indices$Abx.baby.use<-df1$`Has.baby.had.antibiotics.since.birth?`

indices$Race<-df1$Race2

indices$foodstatus2<-df1$foodstatus2


indices1 <- na.omit(indices) #need this to run multi-variate analysis

indices<-indices1


#select IDs from presence

presence<-presence3mos %>%

  filter(ID %in% indices$ID)

presence2<- presence[,4:146]

head(presence2)


abundance<-abundance3mos %>%

  filter(ID %in% indices$ID)
```

```
abundance2<- abundance[,4:146]
head(abundance2)
abundance2[is.na(abundance2)]<- 0


# indices <- abundance[,c("class","pair")]
# head(indices)

##alpha diversity analysis
indices$RichMGE<-rowSums(presence2[,1:27])
ChaoMGE<-t(estimateR(presence2[,1:27]))
chaomge<-ChaoMGE[,2]

indices$Richamino <- rowSums(presence2[,28:48])
summary(indices$Richamino)
ChaoAMINO<-t(estimateR(presence2[,28:48]))
chaoamino<-ChaoAMINO[,2]

indices$RichMDR<-rowSums(presence2[,49:69])
summary(indices$RichMDR)
ChaoMDR<-t(estimateR(presence2[,49:69]))
chaoMDR<-ChaoMDR[,2]

indices$Richbeta<-rowSums(presence2[,70:85])
summary(indices$Richbeta)
```

```
ChaoBETA<-t(estimateR(presence2[,70:85]))
chaobeta<-ChaoBETA[,2]

indices$Richtetra<-rowSums(presence2[,86:98])
summary(indices$Richtetra)
ChaoTETRA<-t(estimateR(presence2[,86:98]))
chaoTETRA<-ChaoTETRA[,2]

indices$RichMLSB<-rowSums(presence2[,99:116])
summary(indices$RichMLSB)
ChaoMLSB<-t(estimateR(presence2[,99:116]))
chaoMLSB<-ChaoMLSB[,2]

indices$Richsulfo<-rowSums(presence2[,117:121])
summary(indices$Richsulfo)
ChaoSulfo<-t(estimateR(presence2[,117:121]))
chaosulfo<-ChaoSulfo[,2]

indices$Richvanco<-rowSums(presence2[,122:129])
summary(indices$Richvanco)
Chaovanco<-t(estimateR(presence2[,122:129]))
chaovanco<-Chaovanco[,2]

indices$Richfluoro<-rowSums(presence2[,130:133])
summary(indices$Richfluoro)
Chaofluoro<-t(estimateR(presence2[,130:133]))
```

```
chaofluoro<-Chaofluoro[,2]

indices$Richother<-rowSums(presence2[,134:143])
summary(indices$Richother)
Chaoother<-t(estimateR(presence2[,134:143]))
chaoother<-Chaoother[,2]

#that was so long
Chao<-t(estimateR(presence2[,28:143]))
ChaoARG<-Chao[,2]

indices$ShannonMGERA<-diversity(abundance2[,1:27])
indices$ShannonaminoRA <- diversity(abundance2[,28:48])
indices$ShannonMDRRA<-diversity(abundance2[,49:69])
indices$ShannonbetaRA<-diversity(abundance2[,70:85])
indices$ShannontetraRA<-diversity(abundance2[,86:98])
indices$ShannonMLSBRA<-diversity(abundance2[,99:116])
indices$ShannonsulfoRA<-diversity(abundance2[,117:121])
indices$ShannonvancoRA<-diversity(abundance2[,122:129])
indices$ShannonfluoroRA<-diversity(abundance2[,130:133])
indices$ShannonotherRA<-diversity(abundance2[,134:143])
indices$ShannonARGRA<-diversity(abundance2[,28:143])
indices$RichARG<-rowSums(presence2[,28:143])

indices$SimpsonMGERA<-diversity(abundance2[,1:27], index="invsimpson")
indices$SimpsonaminoRA<-diversity(abundance2[,28:48], index="invsimpson")
```

```
indices$SimpsonMDRRA<-diversity(abundance2[,49:69], index="invsimpson")
indices$SimpsonbetaRA<-diversity(abundance2[,70:85], index="invsimpson")
indices$SimpsontetraRA<-diversity(abundance2[,86:98], index="invsimpson")
indices$SimpsonMLSBRA<-diversity(abundance2[,99:116], index="invsimpson")
indices$SimpsonsulfoRA<-diversity(abundance2[,117:121], index="invsimpson")
indices$SimpsonvancoRA<-diversity(abundance2[,122:129], index="invsimpson")
indices$SimpsonfluoroRA<-diversity(abundance2[,130:133], index="invsimpson")
indices$SimpsonotherRA<-diversity(abundance2[,134:143], index="invsimpson")
indices$SimpsonARGRA<-diversity(abundance2[,28:143], index="invsimpson")

# ChaoMGE<-t(estimateR(presence2[,1:27]))
# chaomge<-ChaoMGE[,2]



head(ChaoARG)


table(indices$RichMGE)


indices$Abx.baby.use
table(indices$Abx.baby.use)
# NO YES
# 164  28


table(indices$mod)
# cesarean  vaginal
```

```
# 65     127

table(indices$Race)
# non-white    white
# 48      144
table(indices$foodstatus2)
# breastfed   formula
# 85      109

#make variables
MOD<-indices$MOD
Abx.baby.use<-indices$Abx.baby.use
Race<-indices$Race
foodstatus2<-indices$foodstatus2



# need to make chao, shan, etc.
shan<-indices$ShannonARGRA
#Chao-- ARG
model1<-lm(shan~foodstatus2)
model2<-lm(shan~MOD+Abx.baby.use+Race+foodstatus2)
summary(model1)
coef.lm.beta(model1,standardized = F) #This gives effect size
summary(model2)
coef.lm.beta(model2,standardized = F)
```

```
#This gives standardized coefficient: #bigger coefficent means more influential for multivariable analysis

test<-lm.beta(model2)

print(test)


anova(model1,model2,test="Chisq") #This tests whether the complex model is better than the univariate

aov(model1,type = "II") #This gives F values

aov(model2,type = "II")


summary(dominanceAnalysis(model2)) #This gives R2



###chao

# need to make chao, shan, etc. for each abx type

chao<-ChaoARG # change based on what you need (ie RA vs. amino)


#Chao-- ARG


model1<-lm(chao~MOD)

model2<-lm(chao~MOD+Abx.baby.use+Race+foodstatus2)

summary(model1)

coef.lm.beta(model1,standardized = F) #This gives effect size

summary(model2)

coef.lm.beta(model2,standardized = F)
```

```
#This gives standardized coefficient: #bigger coefficent means more influential for multivariable analysis

test<-lm.beta(model2)

print(test)


anova(model1,model2,test="Chisq") #This tests whether the complex model is better than the univariate

aov(model1,type = "II") #This gives F values

aov(model2,type = "II")


summary(dominanceAnalysis(model2)) #This gives R2


#invsimp

invsimp<-indices$RichARG

model1<-lm(invsimp~foodstatus2)

model2<-lm(invsimp~MOD+Abx.baby.use+Race+foodstatus2)

summary(model1)

coef.lm.beta(model1,standardized = F) #This gives effect size

summary(model2)

coef.lm.beta(model2,standardized = F)


#This gives standardized coefficient: #bigger coefficent means more influential for multivariable analysis

test<-lm.beta(model2)

print(test)
```

```
anova(model1,model2,test="Chisq") #This tests whether the complex model is better than the univariate

aov(model1,type = "II") #This gives F values

aov(model2,type = "II")

summary(dominanceAnalysis(model2)) #This gives R2

#shan

shan<-indices$ShannonotherRA

#Chao-- ARG

model1<-lm(shan~Abx.baby.use)

model2<-lm(shan~MOD+Abx.baby.use+Race+foodstatus2)

summary(model1)

coef.lm.beta(model1,standardized = F) #This gives effect size

summary(model2)

coef.lm.beta(model2,standardized = F)

#This gives standardized coefficient: #bigger coefficent means more influential for multivariable analysis

test<-lm.beta(model2)

print(test)

anova(model1,model2,test="Chisq") #This tests whether the complex model is better than the univariate

aov(model1,type = "II") #This gives F values
```

```
aov(model2,type = "II")

summary(dominanceAnalysis(model2)) #This gives R2

#finding out M & SD for variables of interest

indices$ShannonARGRA
mean(indices$ShannonARGRA)
#1.985283
sd(indices$ShannonARGRA)
#0.5336458

shan_vaginal <- subset(indices, (MOD == "vaginal")) # subset
shan_cs <- subset(indices, (MOD == "cesarean")) # subset

mean(shan_cs$ShannonARGRA)
#1.928606 -- v
#c -- c
sd(shan_cs$ShannonARGRA)
#0.5111029
#0.5628196

indices$chaoarg<-ChaoARG
mean(indices$chaoarg)
sd(indices$chaoarg)
```

```
indices$foodstatus2

chao_FF <- subset(indices, (foodstatus2 == "formula")) # subset

chao_BF <- subset(indices, (foodstatus2 == "breastfed")) # subset


mean(chao_FF$chaoarg)

# [1] 1138.944

sd(chao_FF$chaoarg)

#1193.49

summary(chao_FF$chaoarg)


mean(chao_BF$chaoarg)

#788.6353

sd(chao_BF$chaoarg)

#1097.752


#inv simp

indices$SimpsonARGRA

mean(indices$SimpsonARGRA)

sd(indices$SimpsonARGRA)

indices$MOD

simpson_FF <- subset(indices, (foodstatus2 == "formula")) # subset

simpson_BF <- subset(indices, (foodstatus2 == "breastfed")) # subset


mean(simpson_FF$SimpsonARGRA)

# 6.060943

sd(simpson_FF$SimpsonARGRA)
```

```
#3.628709
mean(simpson_BF$SimpsonARGRA)
#5.557687
sd(simpson_BF$SimpsonARGRA)
#2.646787

indices$MOD
simpson_vaginal <- subset(indices, (MOD == "vaginal")) # subset
simpson_CS <- subset(indices, (MOD == "cesarean")) # subset

mean(simpson_vaginal$SimpsonARGRA)
# 5.37558
sd(simpson_vaginal$SimpsonARGRA)
#2.846687
mean(simpson_CS$SimpsonARGRA)
#6.741933
sd(simpson_CS$SimpsonARGRA)
#3.739341

indices$Race

simpson_white <- subset(indices, (Race == "white")) # subset
simpson_nonwhite <- subset(indices, (Race == "non-white")) # subset

mean(simpson_white$SimpsonARGRA)
# 5.850323
```

```
sd(simpson_white$SimpsonARGRA)

#3.078292

mean(simpson_nonwhite$SimpsonARGRA)

#5.80162

sd(simpson_nonwhite$SimpsonARGRA)

#3.693235


### Beta Diversity

#Sorensen

a<-vegdist(presence2[,28:143],method="bray",binary=TRUE)


#Overall significance of model

adonis2(a~MOD+Abx.baby.use+Race+foodstatus2,by=NULL,permutations=9999) #Sig

#Significance of terms in model

adonis2(a~MOD+Abx.baby.use+Race+foodstatus2,by="margin",permutations=9999)


#Univariate

adonis2(a~foodstatus2,by=NULL,permutations=9999)


#Bray-Curtis

a<-vegdist(abundance2[,28:143],method="bray",binary=F)


#Overall significance of model

adonis2(a~MOD+Abx.baby.use+Race+foodstatus2,by=NULL,permutations=9999) #Sig

#Significance of terms in model

adonis2(a~MOD+Abx.baby.use+Race+foodstatus2,by="margin",permutations=9999)
```

```
#Univariate

adonis2(a~MOD,by=NULL,permutations=9999)

adonis2(a~foodstatus2,by=NULL,permutations=9999)


####### Multinomal Logistic Regression #####

require(foreign)

require(nnet)

require(ggplot2)

require(reshape2)

#https://stats.oarc.ucla.edu/r/dae/multinomial-logistic-regression/

infants2<-infants[infants$cluster==3,]

infants2[,c(148)]

df1$cluster

df1$cluster


table(df1$cluster)

#clusters is dependent variable (unordered)

df1$cluster3<-as.factor(ifelse(df1$cluster== '1','clusterone',

                        ifelse(df1$cluster== '2', 'clustertwo', 'clusterthree')))

table(df1$cluster3)

df1$cluster3 <- relevel(df1$cluster3, ref = "clusterone")

levels(df1$cluster3)


df1$cluster3<-factor(df1$cluster3,levels(df1$cluster3)[c(1,3,2)]) # to get 2 in the middle
```

```
MOD<-df1$mode

table(df1$cluster3)

Abx.baby.use<-df1$Has.baby.had.antibiotics.since.birth

Race<-df1$Race2

Foodstatus2<-df1$foodstatus2

cluster3<-df1$cluster3

cluster3

df1$Race2

bcdata

as.factor(Abx.baby.use<-df1$Has.baby.had.antibiotics.since.birth)

#ces is reference group

#non-white is reference group

#breast fed is ref group

#abx: no is reference group


#multi-nomial regression

test <- multinom(cluster3 ~ MOD + Abx.baby.use + Race2 + foodstatus2, data = df1)

test


confint(test)

summary(test)


#z-score

z <- summary(test)$coefficients/summary(test)$standard.errors

z
```

```
#p-value

p <- (1 - pnorm(abs(z), 0, 1))*2

p


exp(coef(test))


#df2<-as.data.frame(df1$cluster2, df1$Race2,
df1$`Has.baby.had.antibiotics.since.birth?`, df1$mode, df1$foodstatus2)


ci <- confint(test, level=0.95)

ci


#install.packages('stargazer')

# library('stargazer')

# stargazer(test, type="text", ci = T)


confint(test)


#stargazer(test, ci = T, single.row = T, type = "text")


#to get RR

multi1.rrr = exp(coef(test))

multi1.rrr


# to get confidence intervals for RR

CI.vector <- exp(confint(test))
```

```
CI.vector

#https://stackoverflow.com/questions/46046173/stargazer-confidence-interval-incorrect


# p.values <- summary(test)$coefficients[, 3]

# p.values

#

# p.values


library(stargazer)

#not exponentiated

# stargazer(test, coef=list(multi1.rrr), ci= T, p = list(p.values), type = "text")

#

# #exponentiated to get odds ratios

# exponentiate <- function(x) exp(x)

# stargazer(test, ci=T, apply.coef=exponentiate, apply.ci=exponentiate,

#         single.row = T, type="text", p = list(p))


#to get a confidence interval

require(effects)

#install.packages("effects")

#"Abx.baby.use" = mean(df1$Has.baby.had.antibiotics.since.birth), "foodstatus2"  = mean(df1$foodstatus2), "race" = mean(df1$Race2)))

#MOD + Abx.baby.use + Race2 + foodstatus2

#2023SEP11_cluster_confidence_intvl_abxuse

# fit.eff <- Effect("foodstatus2", test, given.values = c("cluster2" = mean(df1$cluster2)))

# data.frame(fit.eff$prob, fit.eff$lower.prob, fit.eff$upper.prob)
```

plot(fit.eff)

take 2 --pretty sure this is multiple logistic regression

#examine effect of race (predictor) on cluster (outcome)

model = glm(Race2 ~ cluster2, family = 'binomial', data = df1)

summary(model)

#A multiple logistic regression analysis can be performed using the "glm"

#function in R (general linear models). "glm" includes different procedures so

#we need to add the code at the end "family=binomial (link=logit)" to indicate

#logistic regression. We can conduct the logistic analysis using the code below:

#https://sphweb.bumc.bu.edu/otlt/MPH-Modules/PH717-QuantCore/PH717-Module12-MultipleRegression/PH717-Module12-MultipleRegression8.html

log.out <-glm(cluster2 ~ MOD + Abx.baby.use + Race + Foodstatus2, family=binomial (link=logit))

summary(log.out) #The default output gives the regression slopes which can be used to judge the direction of associations and their statistical significance.

exp(log.out$coeff) #However, since we used log (odds of cluster) as the outcome, we need to exponentiate the coefficients in order to get the odds ratios.

exp(confint(log.out)) #R will also generate the 95% confidence limits for each of these.

```
#towards data science

install.packages('ordinal')

install.packages('readxl')

install.packages('rcompanion')

library(ordinal)

#library(readxl)

library(rcompanion)


model_glm <- glm(cluster2 ~ MOD + Abx.baby.use + Race2 + foodstatus2, family =
binomial, data = df1)

summary(model_glm)


###abx.baby.current

abx.since.birth<-df1$`If.yes,.what.medicine(s)?`

table(df1$`If.yes,.what.medicine(s)?`)

df1$`If.yes,.what.medicine(s)?`

df1$abx.since.birth<-
gsub(".*Antibiotic.*|.*antibiotic.*|.*amoxicillin.*|.*Amp.*|.*Augmentin.*|.*CEPH.*|.*Tobramyci
n.*|.*ANTI.*|.*AMOX.*", "yes", df1$`If.yes,.what.medicine(s)?`)

df1$abx.since.birth



#2023SEP05_cluster_abund_genes_MR.xlsx

write.xlsx(new_df2, "/Users/madeleinerussell/Desktop/Comstock
Lab/AbxResistance/ABXR_Ranalysis/AMR_Data/2023SEP05_cluster_abund_genes_MR.xlsx
", quote=FALSE, rowNames=FALSE)

moms_alldata7
```

```
write.xlsx(moms_alldata7, "/Users/madeleinerussell/Desktop/Comstock Lab/AbxResistance/ABXR_Ranalysis/AMR_Data/2023OCT08_all_data_moms_MR.xlsx", quote=FALSE, rowNames=FALSE)


write.xlsx(moms_alldata3, "/Users/madeleinerussell/Desktop/Comstock Lab/AbxResistance/ABXR_Ranalysis/AMR_Data/2023OCT08_all_data_moms_short_MR.xlsx", quote=FALSE, rowNames=FALSE)


library(tidyverse)

Data.complete<-read.xlsx("2023OCT08_all_data_moms_MR.xlsx")

Data.complete$BMI_all<-as.numeric(Data.complete$BMI_all)

mean(Data.complete$BMI_all, na.rm = TRUE)

Data.complete$BMI_all

BMI_all<-Data.complete$BMI_all

sd(Data.complete$BMI_all, na.rm = TRUE)


sd(Data.complete$MOM_AGE, na.rm = TRUE)


new_df3<-new_df2[new_df2$cluster==3,]


colSums (new_df3[c(3:145)], na.rm = FALSE, dims = 1)



#Trying to get mom info

mom_data<-read.csv("MARCH+SoC+Enrollment+Form_September+8,+2023_15.csv", header = TRUE)

mom_enrollment_data<-mom_data[-c(2), -c(1:17,21:22)]
```

```
view(mom_enrollment_data)

#install.packages('janitor')
require(janitor)
mom_enrollment_data2<-mom_enrollment_data %>%
  row_to_names(row_number = 1)

header.true <- function(df) {
  names(df) <- as.character(unlist(df[1,]))
  df[-1,]
}
mom_enrollment_data2<-header.true(mom_enrollment_data)

mom_enrollment_data2$`MARCH MICROBE ID (MM###)`
mom_enrollment_data2$`Kit #`


moms1<-abundance3mos[grep("Mother",abundance3mos$class),] #select for infants
view(moms1)

mom_enrollment_data2$pair
moms1$pair

names(mom_enrollment_data2)[names(mom_enrollment_data2) == 'Kit #'] <- 'pair'
mom_enrollment_data2$pair
```

```
#birthcert for moms

mom_enroll_abun<-inner_join(moms1, mom_enrollment_data2, by="pair")

mom_enroll_abun2 <- mom_enroll_abun %>% distinct(pair, .keep_all = TRUE)

#


#make cross meta file


crossmeta<-read.xlsx("2023_3mos_Crosswalk_4_MAdi.xlsx")

names(crossmeta)[names(crossmeta) == 'MM_Kit_ID'] <- 'pair'

crossmeta


abundancemoms<-abundance3mos[grep("M", abundance3mos$Timepoint), ]

abunmoms.cross<-left_join(abundancemoms, crossmeta, by="pair")


abunmoms.cross2 <- abunmoms.cross %>% distinct(pair, .keep_all = TRUE)

abunmoms<-abunmoms.cross2


abunmoms$MARCHID


abunmoms$MARCHID


# add meta data



metadata<-read.xlsx("2023FEB03_AMR_MetaData.xlsx")

metadata$MARCHID
```

```
names(metadata)[names(metadata) == 'ID'] <- 'MARCHID'

metadata3mos<-inner_join(abunmoms, metadata, by="MARCHID")

#n= 22

birthcert<-read.xlsx("2022JAN26_data4Madi_set2_bc_info.xlsx")

names(birthcert)[names(birthcert) == 'ID'] <- 'MARCHID'


birthcert_abun_moms<-inner_join(abunmoms, birthcert, by="MARCHID")

birthcert_abun_moms2 <- birthcert_abun_moms %>% distinct(pair, .keep_all = TRUE)

#n=48


birthcert_abun_moms3<-left_join(abunmoms, birthcert, by="MARCHID")

birthcert_abun_moms3 <- birthcert_abun_moms3 %>% distinct(pair, .keep_all = TRUE)

#n=48

view(crossmeta)

mom_ids<-birthcert_abun_moms3[,c(1:3, 148:151)] #written for Dr. C


BCmeta_moms<-birthcert_abun_moms2[,-c(4:146)]

mom_ids


view(moms_alldata3)


#BRIDGEMOMRACE

#MOM_AGE

#SMOKING

#PLURALITY

#MOM_HEIGHT_FT
```

```
#MOM_HEIGHT_IN

#PRE_PREG_WT



#write.xlsx(mom_ids, "/Users/madeleinerussell/Desktop/Comstock Lab/AbxResistance/ABXR_Ranalysis/AMR_Data/2023SEP18_mom_id_list_MR.xlsx", quote=FALSE, rowNames=FALSE)


##Real name of genes-- gathering up to genes 246 which we identified as being the last of the main abundance ones


realname<-read.csv("gene_realname.csv")

cluster_genes<-read.xlsx("2023SEP05_cluster_abund_genes_MR.xlsx")


cluster_genes2<-cluster_genes[c(1:12), c(1:83)]

cluster_genes2

#81 genes associated


x<-colnames(cluster_genes2[-c(1:2)])

x #list of 50 genes

realname$gene<-paste("X", realname$Assay, sep="")


df.gene.test <- realname[match(x, realname$Assay), ]  #create matching list

df.gene.test
```

```
write.xlsx(df.gene.test, "/Users/madeleinerussell/Desktop/Comstock Lab/AbxResistance/ABXR_Ranalysis/AMR_Data/2023SEP18_gene_list_cluster3_MR.xlsx", quote=FALSE, rowNames=FALSE)

#testing things on gene level

arch_bcdata<-read.csv("ARCH+GUT+BC+Data_September+27,+2023_14.16.csv", header = TRUE)

arch_bcdata<-arch_bcdata[-c(2),-c(1:17)]

#n=35

header.true <- function(df) {

  names(df) <- as.character(unlist(df[1,]))

  df[-1,]

}

arch_bcdata2<-header.true(arch_bcdata)

arch_bcdata2$`ARCH Study ID` # arch study ID data

abundance3mos$class

abundance.moms <- filter(abundance3mos, class == "Mother")

abundance.moms$`ARCH Study ID`<-abundance.moms$ID

#names(SIF)[names(SIF) == 'Study.ID'] <- 'match'

abundance.moms$`ARCH Study ID`

abundance.moms$ID<-gsub('.*2012-23.*',"2021-23",abundance.moms$ID)

abundance.moms$ID<-gsub('.*2807-52.*',"2087-52",abundance.moms$ID)
```

```
#2807-52 2087-52

abundance.moms$`ARCH Study ID`<-gsub('-.*',"",abundance.moms$ID)
temp<-left_join(abundance.moms, arch_bcdata2, by="ARCH Study ID")
temp$`ARCH Study ID`
temparch<-inner_join(abundance.moms, arch_bcdata2, by="ARCH Study ID")

temp$ID # this is our arch + BC baby file

#baby gut (study) bc data
babygut_demoq<-
read.csv("BABY+GUT+Demographic+Questionnaire_September+27,+2023_14.12.csv",
header=TRUE)
babygut_demoq

babygut_demoq<-babygut_demoq[-c(2),-c(1:17)]

header.true <- function(df) {
  names(df) <- as.character(unlist(df[1,]))
  df[-1,]
}
babygut_demoq2<-header.true(babygut_demoq)
babygut_demoq2$`Study ID`
names(babygut_demoq2)[names(babygut_demoq2) == 'Study ID'] <- 'ARCH Study ID'

#baby gut (study) bc data
```

```
archgut_demoq<-
read.csv("ARCH+GUT+Demographic+Survey_September+27,+2023_14.18.csv",
header=TRUE)

archgut_demoq


archgut_demoq<-archgut_demoq[-c(2),-c(1:17)]


header.true <- function(df) {
  names(df) <- as.character(unlist(df[1,]))
  df[-1,]
}
archgut_demoq2<-header.true(archgut_demoq) # fix header
archgut_demoq2$`ARCH Study ID`


archgut_demoq3<-archgut_demoq2[-c(2:5,7)] #the demographic that we can match with
baby gut
archgut_demoq4<-archgut_demoq2[c(1,2:5,7)] #remove columns that are not in both
demographic files so we can merge them
archgut_demoq3$`Are you (choose all that apply)? - Selected Choice - White/Caucasian`
babygut_demoq2$`Are you (choose all that apply)? - Other - Text`
names(archgut_demoq3)[names(archgut_demoq3) == 'Are you (choose all that apply)? -
Selected Choice - White/Caucasian'] <- 'Are you (choose all that apply)? - Selected Choice'
df<-archgut_demoq3 #do this so don't mess it up
df2<-babygut_demoq2


#rename columns bc otherwise they won't match
```

```
#ARCH Study ID

#Are you (choose all that apply)? - Selected Choice

#Are you (choose all that apply)? - Other - Text

#Are you Hispanic or Latino?

#What is the highest level of education you have completed?

#What is your current marital status?

#What is your annual household income?

#Do you own: - A home?

#Do you own: - A car?

#Do you own: - Any stocks or bonds?


colnames(df) <- c("ARCH Study ID", "race1","race2","ethnicity","education","marital","income","own_home","own_car","own_stocks")

print(df)


colnames(df2) <- c("ARCH Study ID", "race1","race2","ethnicity","education","marital","income","own_home","own_car","own_stocks")

print(df2)


demographic_data<-rbind(df, df2)


df3<-archgut_demoq4

colnames(df3) <- c("ARCH Study ID", "b","c","d","e","f") #rename columns to make easier to merge

colnames(df3)
```

```
data <- df3 %>%
  mutate(across(everything(), ~ifelse(.=="", NA, as.character(.)))) #need to make na work

df3$`ARCH Study ID`
data<-data %>% mutate(race3 = coalesce(b, c, d, e, f)) %>%
  select('ARCH Study ID', race3)
data

demograph_2$race2
demograph_2<-left_join(demographic_data, data, by= "ARCH Study ID") #left join keeps everything in demographic data

data_test <- demograph_2 %>%
  mutate(across(everything(), ~ifelse(.=="", NA, as.character(.)))) #need to make na work

demograph_3<-data_test %>% mutate(race4 = coalesce(race1, race2, race3)) %>%
  select("ARCH Study ID", "race4","ethnicity","education","marital","income","own_home","own_car","own_stocks")

#now combine
temp2<-temp[grep("B",temp$`ARCH Study ID`),]
temp2$`ARCH Study ID`

babygut_temp<-left_join(temp2, babygut_demoq2, by= 'ARCH Study ID')
babygut_temp #29 BG babies
```

```
temp3<-left_join(temp, demograph_3, by="ARCH Study ID")

temp4<- temp3 %>% distinct(pair, .keep_all = TRUE)


temp5<-temp4[,-c(4:146)]

temp5 # only interesting info


#25 babies for who we have bc data


bc_data_only<-bcdata[,-c(4:146)]

bcdata$pair


#BCmeta_moms

bc_data_only2<-bc_data_only[,-c(5,30:31)] #getting rid of non-matching columns

head(bc_data_only2)

BCmeta_moms2<-BCmeta_moms[,-c(6:8)] #getting rid of non-matching columns

#names(BCmeta_moms)[names(BCmeta_moms) == ''] <- 'pair'


bc_meta_data_moms <- rbind(bc_data_only2, BCmeta_moms2)

bc_meta_data_moms2 <- bc_meta_data_moms %>% distinct(match, .keep_all = TRUE)


moms_alldata<-left_join(temp5, bc_meta_data_moms2, by= 'match')


write.xlsx(moms_alldata, "/Users/madeleinerussell/Desktop/Comstock
Lab/AbxResistance/ABXR_Ranalysis/AMR_Data/2023OCT06_moms_alldata_MR.xlsx",
quote=FALSE, rowNames=FALSE)
```

```
#yay you don't have to remake it


moms_alldata2<-moms_alldata[,c(1:6)]

moms_alldata2


moms_alldata$BRIDGEMOMRACE

moms_alldata2$Race<-as.factor(ifelse(moms_alldata$BRIDGEMOMRACE== '1', 'white',
                        ifelse(moms_alldata$BRIDGEMOMRACE== '2', 'black',
                            ifelse(moms_alldata$BRIDGEMOMRACE== '24', 'asian',
                                ifelse(moms_alldata$BRIDGEMOMRACE== '10', 'asian',
                                    ifelse(moms_alldata$BRIDGEMOMRACE== '22', 'black',
                                        ifelse(moms_alldata$BRIDGEMOMRACE== '8', 'asian',
'other')))))))

moms_alldata2$Race

moms_alldata2$race4<-moms_alldata$race4

moms_alldata2$` Race of the Mother (RACEMOM):` <-moms_alldata$` Race of the Mother
(RACEMOM):`

moms_alldata3<-unite(moms_alldata2, col='race_all', c('Race', 'race4','Race of the Mother
(RACEMOM):'), sep='-')


moms_alldata3$race3<-gsub(".*white.*", "white",
              gsub(".*Pacific.*", "Pacific Islander",
                 gsub(".*Black.*", "black",
                    gsub(".*Asian.*", "asian",
                       gsub(".*black.*", "black", moms_alldata3$race_all)))))

moms_alldata3$race5<-gsub(".*White.*", "white", moms_alldata3$race3)

moms_alldata3$race6<-gsub(".*other.*", "other", moms_alldata3$race5)
```

```
moms_alldata3$race6<-gsub(".*NA.*", NA, moms_alldata3$race6)

moms_alldata3$RACE<-as.factor(ifelse(moms_alldata3$race6== 'white', 'white', 'non-
white'))


table(moms_alldata3$race6)

table(moms_alldata3$RACE)

moms_alldata3


#mom age at time of birth

moms_alldata3$Mom_DOB

moms_alldata3$Mom_DOB<-moms_alldata$`Mother's Date of Birth (MOM_BIRTH):`

moms_alldata3$child_DOB<-moms_alldata$`Child's Date of Birth (BXYEAR):`


moms_alldata3$Mom_DOB2<-gsub("/8", '/198',

                    gsub("/7", '/197',

                      gsub("/9", "/199", moms_alldata3$Mom_DOB)))

moms_alldata3$Mom_DOB2<-gsub("4/198/1982", "4/8/1982",
moms_alldata3$Mom_DOB2) # this date got wonky

moms_alldata3$Mom_DOB2


moms_alldata3$Mom_DOB3<-
as.Date(moms_alldata3$Mom_DOB2,format="%m/%d/%Y")

moms_alldata3$Mom_DOB3 # moms bday


moms_alldata3$child_DOB

moms_alldata3$child_DOB2<-gsub("/16", '/2016',

                     gsub("/17", '/2017',
```

```
                gsub("/15", "/2015", moms_alldata3$child_DOB)))
moms_alldata3$child_DOB2


moms_alldata3$child_DOB3<-
as.Date(moms_alldata3$child_DOB2,format="%m/%d/%Y")
moms_alldata3$child_DOB3 # babies bday


moms_alldata3$Date_difference_in_days <-difftime(moms_alldata3$child_DOB3,
moms_alldata3$Mom_DOB3, units=c("days"))
moms_alldata3$Date_difference_in_days
moms_alldata3$Date_difference_in_days2<-
(moms_alldata3$Date_difference_in_days)/365
moms_alldata3$Date_difference_in_days2



moms_alldata3$MOM_AGE<-moms_alldata$MOM_AGE


moms_alldata3<-unite(moms_alldata3, col='all_mom_age', c('Date_difference_in_days2',
'MOM_AGE'), sep='-')
moms_alldata3$all_mom_age
moms_alldata3$all_mom_age<-gsub("NA-", "", moms_alldata3$all_mom_age)
moms_alldata3$all_mom_age<-gsub("-NA", "", moms_alldata3$all_mom_age)
moms_alldata3$all_mom_age<-gsub("NA", NA, moms_alldata3$all_mom_age)
table(moms_alldata3$all_mom_age)


moms_alldata3$all_mom_age<-as.numeric(moms_alldata3$all_mom_age)
summary(moms_alldata3$all_mom_age)
```

```
#smoking
moms_alldata$MOMSMOKE
moms_alldata$smoking<-as.factor(ifelse(moms_alldata$MOMSMOKE== '1','smoking',
                          ifelse(moms_alldata$MOMSMOKE== '2', 'non-smoking','non-smoking')))
table(moms_alldata$smoking)

moms_alldata$`Did Mother Smoke Before of During Pregnancy (TOBACCO)?`

#plurality
moms_alldata$`Plurality (PLURALITY):`
table(moms_alldata$PLURALITY)

#*plurality  1  2
#47  1
pluarality<-moms_alldata[,c(1,117)]
table(moms_alldata$`Number of Previous Live Births Now Living (NOWLIVE):`)
# 0  1  2  4  5
# 9 11  3  1  2
moms_alldata$`Number of Previous Live Births Now Dead (NOWDEAD):` # none


#add bg data
#baby gut enrollment
arch_enroll_question<-
read.csv("ARCH+GUT+Enrollment+Questionnaire_September+27,+2023_14.26.csv",
header=TRUE)
```

```
arch_enroll_question2<-arch_enroll_question[c(-1),-c(1:18, 21:57)]

arch_enroll_question2$pair.x

names(arch_enroll_question2)[names(arch_enroll_question2) == 'Gut.Kit.ID'] <- 'pair.x'


moms_alldata7<-left_join(moms_alldata, arch_enroll_question2, by= 'pair.x')


####BMI######


moms_alldata7$MOM_HEIGHT_IN

moms_alldata7$MOM_HEIGHT_FT

moms_alldata7$inches_height<-moms_alldata7$`Mother's Height (MOM_HEIGHT) in
Inches:` #need to combine this with the other two


ft<-moms_alldata7$MOM_HEIGHT_FT

ft

ft_to_in<-ft*12

ft_to_in



inches<-moms_alldata7$MOM_HEIGHT_IN

height_in_inches<-ft_to_in + inches

moms_alldata7$height_in_inches<-height_in_inches


moms_alldata7 <- moms_alldata7 %>% mutate(height_in_inches =
as.character(height_in_inches))

is.character(moms_alldata7$height_in_inches)
```

```
data<-moms_alldata7 %>% mutate(height = coalesce(height_in_inches, inches_height))
%>%
  select('ARCH Study ID', height)
data$height


moms_alldata7$height<-data$height
moms_alldata7$height
height<-moms_alldata7$height



moms_alldata7$PRE_PREG_WT #now combine weight
moms_alldata7$`Mother's Pre-pregnancy Weight (PWGT) in lbs:`


moms_alldata7$`Mother's Pre-pregnancy Weight (PWGT) in lbs:`


data<-moms_alldata7 %>% mutate(weight = coalesce(PRE_PREG_WT, `Mother's Pre-
pregnancy Weight (PWGT) in lbs:`)) %>%
  select('ARCH Study ID', weight)
data$weight


weight<-data$weight
weight
a<-as.factor(moms_alldata7$height)
a


moms_alldata7$weight<-data$weight
moms_alldata7$weight<-gsub('999',"",moms_alldata7$weight)
```

```
b<-as.factor(moms_alldata7$weight)

b

#make it a numeric factor

height<-as.numeric(as.character(a))

height<-height*0.0254

height #height in meters


weight<-as.numeric(as.character(b))

weight<-weight/2.205 #weight in kg


BMI<-weight/(height*height)

moms_alldata7$BMI<-BMI

moms_alldata7$weight<-weight

moms_alldata7$height_in_meters<-height


moms_alldata7$BMI


table(moms_alldata7$BMIcategory)


moms_alldata7$Calculated.pre.pregnancy.BMI


moms_alldata7 <- moms_alldata7 %>% mutate(BMI = as.character(BMI))

is.character(moms_alldata7$BMI)


data<-moms_alldata7 %>% mutate(BMI_all = coalesce(BMI,
Calculated.pre.pregnancy.BMI)) %>%
```

```
  select('ARCH Study ID', BMI_all)
data$BMI_all


moms_alldata7$BMI_all<-data$BMI_all
#creating BMI categories
moms_alldata7$BMIcategory<-as.factor(ifelse(moms_alldata7$BMI_all <= 18.5, 'normal',
                                ifelse((moms_alldata7$BMI_all >= 25) & (moms_alldata7$BMI_all <=
29.9), 'overweight',
                                    ifelse(moms_alldata7$BMI_all >= 30, 'obese', 'normal'))))




table(moms_alldata7$BMIcategory)


#BRIDGEMOMRACE-- done
#MOM_AGE-- done
#SMOKING-- done
#PLURALITY-- done
#MOM_HEIGHT_FT
#MOM_HEIGHT_IN
###


new_df2<-read.xlsx("2023SEP05_cluster_abund_genes_MR.xlsx")
data<-new_df2[c(1:2)]


bcdata<-read.xlsx("2023APR21_AMR_3mosBCData_plusabun.xlsx")
```

```
#doing this because un even number ids

data$ID

names(data)[names(data) == 'ID'] <- 'match'

bcdata_cluster<-left_join(data, bcdata, by="match")


SIF<-read.xlsx("MARCH3mos_SIF_MR.xlsx")

names(SIF)[names(SIF) == 'Study.ID'] <- 'match'

head(SIF)

SIF<-SIF[,-c(11:12)]


SIFabun2<-left_join(bcdata_cluster, SIF, by="match")

df1 <- SIFabun2 %>% distinct(match, .keep_all = TRUE)

df1


df1.cluster3<-df1[grep("3",df1$cluster),]
```
