# Supplementary material for: Associations of diet, race, and other environmental factors with antimicrobial resistance genes in the gut bacterial communities of pregnant women and 3-month-old infants
Source: mSphere. 2025 Nov 24;10(12):e00445-25. doi: 10.1128/msphere.00445-25 (PMC12724134; doi:10.1128/msphere.00445-25)
Supplement: Data Directory — Information to understand the Data File in the supplemental material. [file msphere.00445-25-s0005.docx]

Data Directory

All of the following files have been uploaded as supplemental data.

- For participant data, this is reported in the following csv document: **Data_File.csv**
  - For columns H-EU, these are the raw CT counts for each participant for each gene assay. Numbers in the column IDs in this group refer to the gene assay used. Gene assay ID and information can be found in the following document: **Table S1**
  - For columns EX-KJ, these are abundance data, calculated as described in the manuscript. The numerical column heading is the gene assay followed by .x. Genes were arranged based on functional classification for data analysis. Function of each gene target can be found in: **Table S1**
  - For columns KO-QA, these are “presence or absence of a gene” data, calculated as described in the manuscript. If an individual had any detection of a particular gene, this was denoted with a 1, and if they had no detection of the gene, this was denoted with a 0. The numerical column heading is the gene assay followed by .y. Genes were arranged based on functional classification for data analysis. Function of each gene target can be found in: **Table S1**
  - Columns QC-VA are compiled participant data from sample info forms, birth certificate, and medical record abstraction. For privacy reasons, private health information and identifiers have been removed.
- Complete R script and analysis is available in the **Code.pdf** file**.**
