# Supplementary material for: Associations of diet, race, and other environmental factors with antimicrobial resistance genes in the gut bacterial communities of pregnant women and 3-month-old infants
Source: mSphere. 2025 Nov 24;10(12):e00445-25. doi: 10.1128/msphere.00445-25 (PMC12724134; doi:10.1128/msphere.00445-25)
Supplement: Table S1 — Genes assayed and their functional classification. [file msphere.00445-25-s0006.pdf]

Table S1. List of genes assayed and their functional classification.

Assay	Name	Functional.classification	Target.antibiotics.(major)
6	aacA/aphD	deactivate	Aminoglycoside
14	aphA3	deactivate	Aminoglycoside
49	sat4	deactivate	Aminoglycoside
104	aph(2')-Id	deactivate	Aminoglycoside
174	aadE	deactivate	Aminoglycoside
402	aph4ib	aph4ib	Aminoglycoside
404	aph6ic	aph6ic	Aminoglycoside
406	spcN	deactivate	Aminoglycoside
410	aac(3)-iid_iii_i	aac(3)-iid_iii_iif_iia_iie	Aminoglycoside
412	Aac6-Aph2	Aac6-Aph2	Aminoglycoside
417	aac(6)-im	aac(6)-im	Aminoglycoside
425	aadA7	aadA7	Aminoglycoside
428	aadA17	aadA17	Aminoglycoside
429	aadB	aadB	Aminoglycoside
431	ant6-ia	ant6-ia	Aminoglycoside
432	aph3-ib	aph3-ib	Aminoglycoside
438	acc3-iva	deactivate	Aminoglycoside
1503	aph6ia	deactivate	Aminoglycoside
1540	aph3-III	aph3-III	Aminoglycoside
1541	ant6-ib	aph3-III	Aminoglycoside
1545	aac(3)-Xa	aac(3)-Xa	Aminoglycoside
53	ceoA	efflux	Amphenicol
42	blaOXY-2	deactivate	Beta Lactam
46	cphA	deactivate	Beta Lactam
106	cfxA	deactivate	Beta Lactam
107	cepA	deactivate	Beta Lactam
108	blaCMY	deactivate	beta Lactam
121	blaSFO	deactivate	Beta Lactam
153	Pbp5	protection	Beta Lactam
162	blaCTX-M	deactivate	Beta Lactam
236	penA	protection	Beta Lactam
362	NDM new	deactivate	Beta Lactam
1108	blaCTX-M-1,3,	blaCTX-M-1,3,15	Beta Lactam
1118	blaOXY-1	deactivate	Beta Lactam
1123	blaMIR	bla_MIR	Beta Lactam
1505	ampC	deactivate	Beta Lactam
1512	blaTEM	deactivate	Beta Lactam
1544	bla-ACT	bla-ACT	Beta Lactam
328	qnrB-bob_red	efflux	Fluoroquinolone
1200	norA	norA	Fluoroquinolone
1201	qepA_1_2	qepA_1_2	Fluoroquinolone
1577	oqxA		Fluoroquinolone
366	orf39-IS26	MGE	Insertional sequence
370	ISSm2-Xantho	MGE	Insertional sequence
372	ISEfm1-Enterc	MGE	Insertional sequence
376	IS1111	MGE	Insertional sequence
336	int1-a-marko	MGE	Integrase
338	intl2	MGE	Integrase

359	intI1F165_clin	MGE	Integrase
1522	intl3	MGE	Integrase
9	acrB	efflux	MDR
11	acrF	efflux	MDR
64	emrD	efflux	MDR
81	mdtE/yhiU	efflux	MDR
89	mexA	efflux	MDR
234	oprD	efflux	MDR
245	mepA	efflux	MDR
246	mexE	efflux	MDR
298	tolC	efflux	MDR
331	merA-marko	unknown	MDR
355	marR	regulator	MDR
1504	bexA/norM	efflux	MDR
1509	mdtA	efflux	MDR
1300	mdth	mdth	MDR-chromosomal
1302	mdtg	mdtg	MDR-mobile
1303	pcoA	pcoA	MDR-mobile
1305	arsA	arsA	MDR-mobile
1536	czcA	czcA	MDR-mobile
1549	sugE	sugE	MDR-mobile
1572	terW		MDR-mobile
1573	pbrT		MDR-mobile
1528	ISCR1		MGE
1546	IS26	IS26	MGE
1547	IS3	IS3	MGE
1548	IS256	IS256	MGE
1551	IS200_1	IS200_1	MGE
1552	IS1247	IS1247	MGE
1553	IS630	IS630	MGE
1556	TN5403		MGE
1557	IS200		MGE
1558	IS21-ISAs29		MGE
1559	Tn3		MGE
1565	IncI1_repI1		MGE
1567	IS91		MGE
91	erm(36)	protection	MLSB
137	ermT	protection	MLSB
138	msr(C)	msr(C)	MLSB
209	ermX	protection	MLSB
227	vgaB	efflux	MLSB
229	pica	protection	MLSB
283	ermA/ermTR	protection	MLSB
285	oleC	efflux	MLSB
801	ere(A)	ere(A)	MLSB
804	erm(B)	erm(B)	MLSB
806	erm(E)	erm(E)	MLSB
809	erm(Q)	erm(Q)	MLSB
812	mphA	deactivate	MLSB
815	erm(35)	erm(35)	MLSB

817	erm(F)	erm(F)	MLSB
819	lsa(C)	lsa(C)	MLSB
1511	mefA	efflux	MLSB
1519	lnuC	deactivate	MLSB
239	pmrA	deactivate	Other
700	fosb	fosb	Other
704	mcr-1	mcr-1	Other
1500	bacA_F	bacA_F (bacitracin)	Other
1520	fabK	protection	Other
910	catQ	catQ	Phenicol
911	cmlV	cmlV	Phenicol
340	IncN_rep	MGE	Plasmid incompatibility
342	IncP_oriT	MGE	Plasmid incompatibility
133	sul2	protection	Sulfonamide
177	strB	protection	Sulfonamide
208	folA	protection	Sulfonamide
280	sulA/folP	protection	Sulfonamide
363	sul1 NEW	protection	Sulfonamide
54	tet(32)	protection	Tetracycline
180	tetA	efflux	Tetracycline
181	tetB	efflux	Tetracycline
185	tetQ	protection	Tetracycline
191	tetW	protection	Tetracycline
196	tetX	deactivate	Tetracycline
200	tetS	protection	Tetracycline
294	tetbP	efflux	Tetracycline
506	tetR	tetR	Tetracycline
507	tetG_F	tetG_F	Tetracycline
1507	tetPA	efflux	Tetracycline
1513	tetM	protection	Tetracycline
1539	tet44	tet44	Tetracycline
26	IS613	MGE	Transposase
201	tnpA	MGE	Transposase
202	tnpA	MGE	Transposase
206	tnpA	MGE	Transposase
603	dfra21	dfra21	trimethoprim
610	dfrA22	dfrA22	trimethoprim
211	VanB	VanB	Vancomycin
213	vanD	protection	Vancomycin
214	vanHD	protection	Vancomycin
215	vanHB	protection	Vancomycin
306	vanRB	protection	Vancomycin
309	vanRD	protection	Vancomycin
316	vanTG	protection	Vancomycin
318	vanYD	protection	Vancomycin
